# *cis*-Selective Direct Halocyclopropanation of Alkenes Mediated by Nucleophilic Cobalt Photocatalysis

**DOI:** 10.1021/jacsau.6c00381

**Published:** 2026-06-01

**Authors:** John Hayford G. Teye-Kau, Martin Pauze, Spencer P. Pitre

**Affiliations:** Department of Chemistry, Oklahoma State University, Stillwater, Oklahoma 74078, United States

**Keywords:** cyclopropanes, vitamin B_12_, alkenes, haloforms, cis-selectivity

## Abstract

The cyclopropyl group is an invaluable motif in both medicinal and synthetic chemistry, with 1,2-disubstituted halocyclopropanes serving as important building blocks for the preparation of more complex cyclopropane-containing structures. Herein, we report a Vitamin B_12_-photocatalyzed approach for the direct synthesis of 1,2-disubstituted halocyclopropanes, starting from alkenes and simple haloforms under visible-light irradiation. Our method yields a diverse range of 1,2-disubstituted halocyclopropanes with high *cis*-selectivity, providing efficient access to these traditionally difficult-to-prepare stereoisomers. Mechanistic studies highlight the crucial role of Vitamin B_12_ in influencing the stereochemical outcome of the reaction.

## Graphical abstract

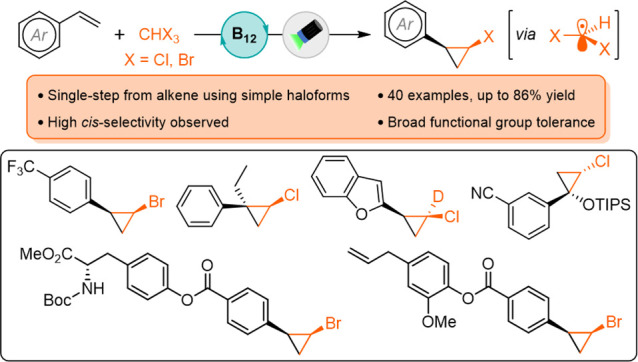

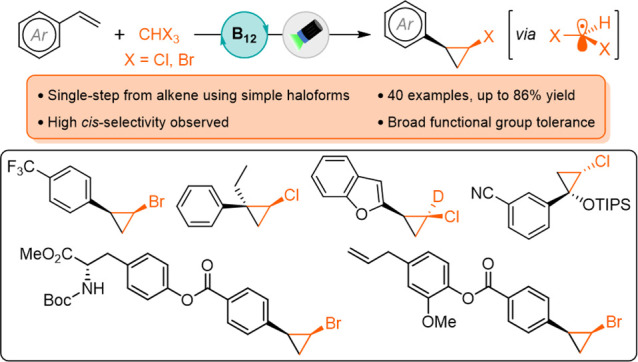

## Introduction

Cyclopropane rings are invaluable to both the medicinal and synthetic chemistry communities, owing to their ability to modulate key pharmacological properties^1–3^ and their unique chemical properties that can be exploited in chemical synthesis.^4–6^ Given their importance, reliable and efficient strategies for the synthesis and derivatization of these scaffolds are of great value. In this context, 1,2-disubstituted halocyclopropanes have been shown to be key building blocks for the construction of more complex cyclopropane-containing structures.^7–13^ Furthermore, monochlorinated cyclopropanes can also be found in natural products with promising bioactivities,^14–16^ further emphasizing the importance of these motifs in potential drug discovery and development. Among the synthetic strategies for the synthesis of halocyclopropanes, common approaches are the Simmons–Smith cyclopropanation reaction^17–21^ and a two-step process involving the preparation of a dihalocyclopropane followed by a second monodehalogenation step (see Scheme 1A). The latter, and most widely used, approach involves the [2 + 1] cycloaddition of a dihalocarbene, generated from a haloform under basic conditions, with an alkene, which generally proceeds in good yields for a broad range of alkene substrates.^22–24^ However, current methods for the selective monodehalogenation step, which often relies on organometallic reagents or strongly reducing metals, suffer from a myriad of drawbacks, including harsh reaction conditions, low yields, low functional group tolerance, and poor diastereoselectivity of the resulting 1,2-disubstituted halocyclopropane.^10,25–30^ More recently, a range of one-step methods that address many of these shortcomings have been developed, which include leveraging techniques such as transition-metal catalysis,^31–33^ electrochemistry,^34^ and photoredox catalysis.^35,36^ Of note, Tambar and coworkers have reported the photomediated isomerization of cinnamyl chlorides to 1,2-substituted chlorocyclopropanes, which proceeds with remarkable selectivity for the *trans*-isomer (>95:5).^35^ More recently, Fasan and coworkers disclosed an enzyme-catalyzed carbene transfer protocol to generate bromo- and chlorocyclopropanes from β-halostyrenes in high diastereo- and enantioselectivity.^37^ In other recent developments, Nagib and coworkers reported an iron catalyzed direct halocyclopropanation of 1,1-diphenylethylene, which was proposed to proceed via iron carbene intermediates.^38^ Despite these elegant advances, reports of direct approaches for the synthesis of *cis*-halocyclopropanes are limited to iodocyclopropanes.^39^ For *cis*-bromo- and chlorocyclopropyl analogs, their preparation and isolation often relies on the separation of *cis*- and *trans*-isomers from unselective reactions. Given prior observations from both our group^40^ and from Chen and Zhang^41^ that Vitamin B_12_-catalyzed cyclopropanation reactions showed slight preference for the formation of the *cis*-isomer, we hypothesized that we could leverage this catalytic platform and our prior expertise to develop a one-step, *cis*-selective halocyclopropanation of alkenes directly from haloforms (CHX_3_).

**Scheme 1:**
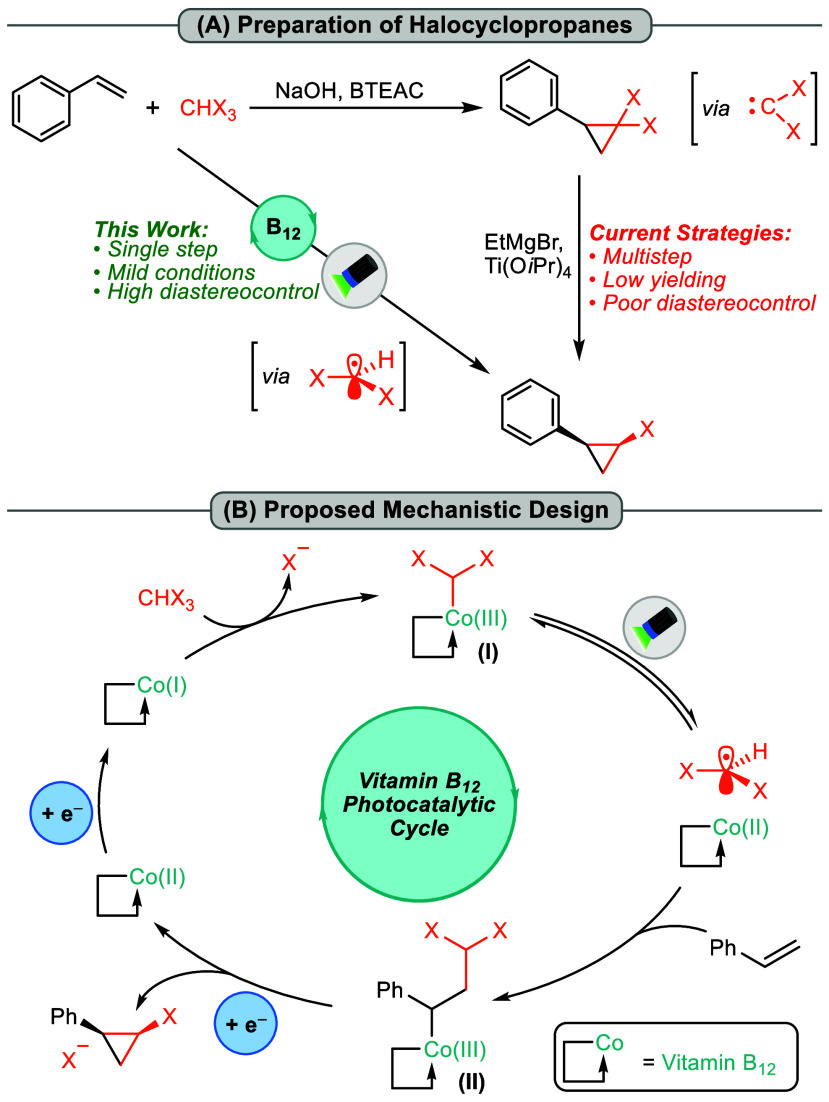
(A) Preparation of *cis*-1,2-Disubstituted Halocyclopropanes; (B) Proposed Mechanistic Design for the Direct Halocyclopropanation of Alkenes Mediated by Nucleophilic Cobalt Photocatalysis

Our proposed mechanistic design for our *cis*-selective direct halocyclopropanation of alkenes mediated by nucleophilic cobalt photocatalysis is outlined in Scheme 1B. Based on prior art using Vitamin B_12_ and other macrocyclic tetradentate cobalt complexes,^42–45^ including our recent contribution for the activation of CH_2_Cl_2_ as a radical precursor and methylene source in cyclopropanation reactions,^40^ we anticipate that generation of the highly nucleophilic Co­(I) oxidation state of Vitamin B_12_ would enable an S_N_2-type nucleophilic substitution with CHX_3_,^46^ generating Co­(III)–CHX_2_ intermediate **I**.^47^ Intermediate **I** could then undergo photolysis upon exposure to green (525 nm) LED irradiation to form a ^•^CHX_2_ radical and a persistent Co­(II) radical.^48^ Addition of the electrophilic ^•^CHX_2_ radical to an activated alkene, such as styrene, followed by trapping of the carbon radical intermediate by Co­(II) would yield intermediate **II**, which we tentatively propose gets reduced to liberate the carbanion that would ultimately yield the halocyclopropane after a polar 3-*exo*-*tet* cyclization.

## Results and Discussion

We began our investigation of the direct halocyclopropanation of alkenes using styrene (**1**) as the model substrate (Table 1). After optimization of each of the key reaction parameters, we found that chlorocyclopropanation of **1** proceeded efficiently using 4 equiv CHCl_3_, 5 mol % Vitamin B_12_, 5 equiv of Zn as the stoichiometric reductant and 1 equiv NH_4_Cl in DMA under 525 nm LED irradiation for 8 h, generating cyclopropyl adduct **2** in 64% NMR yield (entry 1). Importantly, high selectivity for the *cis*-**2** adduct (*cis:trans* 86:14) was observed under these optimized reaction conditions. The reaction was also compatible with CHBr_3_, generating **3** in 58% yield, albeit with a slightly decreased *cis:trans* ratio of 78:22 (entry 2). Switching to CHI_3_ did provide the corresponding cycloadduct **4** with good *cis*-selectivity (84:16); however, a low yield was observed, with the remaining mass balance being recovered starting material (entry 3). We hypothesize the low yield stems from side reactivity between Zn and CH_3_I. DMF was also effective as a solvent, albeit with a decreased yield (entry 4). The use of Mn as a stoichiometric reductant led to a low yield of **2** and a sharp decrease in *cis:trans* selectivity, likely owing to off-cycle reactivity between Mn and CHCl_3_ (entry 5). Switching to a 456 nm LED also led to a decrease in reaction efficiency (entry 6), likely owing to the weaker absorption of the Co­(III)–CHX_2_ ligand-to-metal charge-transfer (LMCT) band under 500 nm (see SI). Control reactions indicated degassed conditions were optimal and Zn was crucial for product formation (entries 7–8). Removing Vitamin B_12_ resulted in only 8% yield for CHCl_3_ (entry 9) and no observable product for CHBr_3_ (entry 10). Furthermore, the observed product formed in the absence of Vitamin B_12_ was found to have minimal *cis:trans* selectivity (55:45). These results highlight that Vitamin B_12_ plays a critical role in generating the corresponding halocyclopropanes with high *cis*-selectivity, with minimal competing Simmons–Smith reactivity being observed under our standard conditions. These findings are consistent with a recent report from Nagib and coworkers, who observed that Zn was unable to efficiently activate CHCl_3_ in the absence of LiI.^38^ While removal of NH_4_Cl only resulted in a slight reduction in yield (entry 11), we have observed greater reaction reproducibility using this acid additive, removing any variation that could be observed related to inconsistent Zn activation. Consistent with our prior findings,^40^ 30% yield of **2** could be obtained in the absence of LED irradiation, likely resulting from inefficient thermolysis of the weak Co­(III)–C bond at room temperature (entry 12). Increasing the LED intensity, while resulting in the highest *cis*-selectivity, was found to negatively impact the yield (entry 13). Finally, adding TEMPO to the reaction resulted in a decreased yield of **2**, highlighting the potential involvement of radical intermediates (entry 14).

**Table 1:**
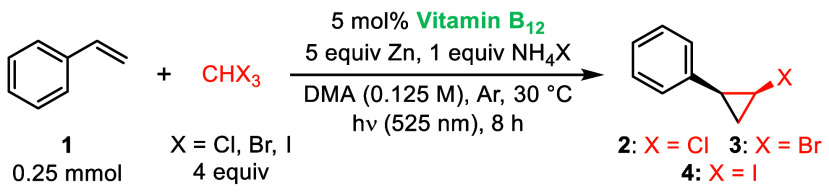
Optimization and Control Reactions for the Halocyclopropanation of Styrene^a^

Entry	X	Deviations from standard conditions	Product: yield (%)	*cis*:*trans*
1	Cl	none	**2**: 64	86:14
2	Br	none	**3**: 58	78:22
3	I	none	**4:** 14	84:16
4	Cl	DMF instead of DMA	**2:** 50	85:15
5	Cl	Mn instead of Zn	**2:** 24	56:44
6	Cl	456 nm LED	**2:** 45	86:14
7	Cl	not degassed	**2:** 50	87:13
8	Cl	no Zn	**2:** N.D.	-
9	Cl	no Vitamin B_12_	**2:** 8	55:45
10	Br	no Vitamin B_12_	**3:** N.D.	-
11	Cl	no NH_4_Cl	**2:** 53	87:13
12	Cl	no hν, rt	**2:** 30	79:21
13	Cl	100% LED Intensity	**2:** 47	91:9
14	Cl	2 equiv TEMPO	**2:** 33	85:15

a Standard conditions: **1** (0.25 mmol, 1 equiv), CHX_3_ (4 equiv), Vitamin B_12_ (5 mol %), Zn (5 equiv), NH_4_X (1 equiv) in DMA (2 mL) were irradiated under Ar with a Kessil PR-160L 525 nm LED (75% intensity) at 30 °C for 8 h. Yields and dr ratios were determined by ^1^H NMR analysis of the crude reaction mixture using 1,3,5-trimethoxybenzene as an external standard. N.D.: not detected.

With the optimized reaction conditions in hand, we then examined the scope of the halocyclopropanation reaction (Scheme 2). Using either CHCl_3_ or CHBr_3_, a series of *cis*-selective halocyclopropyl adducts could be generated in moderate to good yields and good *cis*:*trans* selectivity (>80:20) from a range of styrenes (**2**–**15**). In most of these cases, the minor *trans*-isomer could be removed during purification, leading to highly pure *cis*-halocyclopropanes. The reaction proceeded equally efficiently regardless of whether the styrene contained electron donating (**6**–**10**) or electron withdrawing (**11**–**14**) functional groups. The reaction could tolerate aryl halides (**14**) and aryl boronic esters (**15**), which can serve as functional handles for further cross-couplings and derivatizations. Styrenyl analogues of benzoic acid and aniline were not compatible with our method. While 1,1-disubstituted alkenes were well tolerated (**16**–**19**), albeit with decreased *cis*-selectivity, 1,2-disubstituted styrenes were found to be unreactive. Indene (**20**) also underwent chlorocyclopropanation in 46% yield, and *trans*-1-phenyl-1,3-butadiene (**21**) was also compatible, with chlorocyclopropanation taking place exclusively at the terminal alkene of the diene. Styrenes derived from eugenol (**22**), naproxen (**23**), *L*-tyrosine (**24**), and probenecid (**25**) all provided the desired *cis*-selective halocyclopropyl adducts in moderate to good yields, highlighting the potential of our method for the late-stage functionalization of drugs and natural products. Vinyl heterocycles derived from pyridine (**26**), indole (**27**), benzofuran (**28**) and benzothiophene (**29**) all reacted smoothly under our optimized conditions to give the corresponding chlorocyclopropyl adducts in moderate to good yields. Electron-deficient alkenes, while still compatible owing to the mild nucleophilicity of the ^•^CHX_2_ radical,^49^ gave lower yields of the desired *cis*-halocyclopropane (see SI). On the other hand, unactivated alkenes did not yield any of the desired halocyclopropyl adducts. Reactions could also be directly scaled up to 1.0 mmol scale with no loss in reactivity (**12**, **19**). Given the recent interest in deuterium incorporation in small molecule pharmaceutics,^50–52^ we also investigated the direct halodeuterocyclopropanation of alkenes using readily available CDCl_3_. In all cases, good to excellent yields and remarkable diastereoselectivity of the desired chlorodeuterocyclopropyl adducts were observed (**30**–**34**). Finally, a series of silyl enol ethers were also examined as suitable alkene substrates for our halocyclopropanation protocol. In all cases, good to excellent yields of the desired trisubstituted cyclopropanes were observed (**35**–**40**). Furthermore, subsequent TMS-deprotection following the photomediated halocyclopropanation reaction led to successful isolation of the trisubstituted cyclopropanols **41** and **42**, which have been shown to be useful building blocks in organic synthesis.^53–56^

**Scheme 2:**
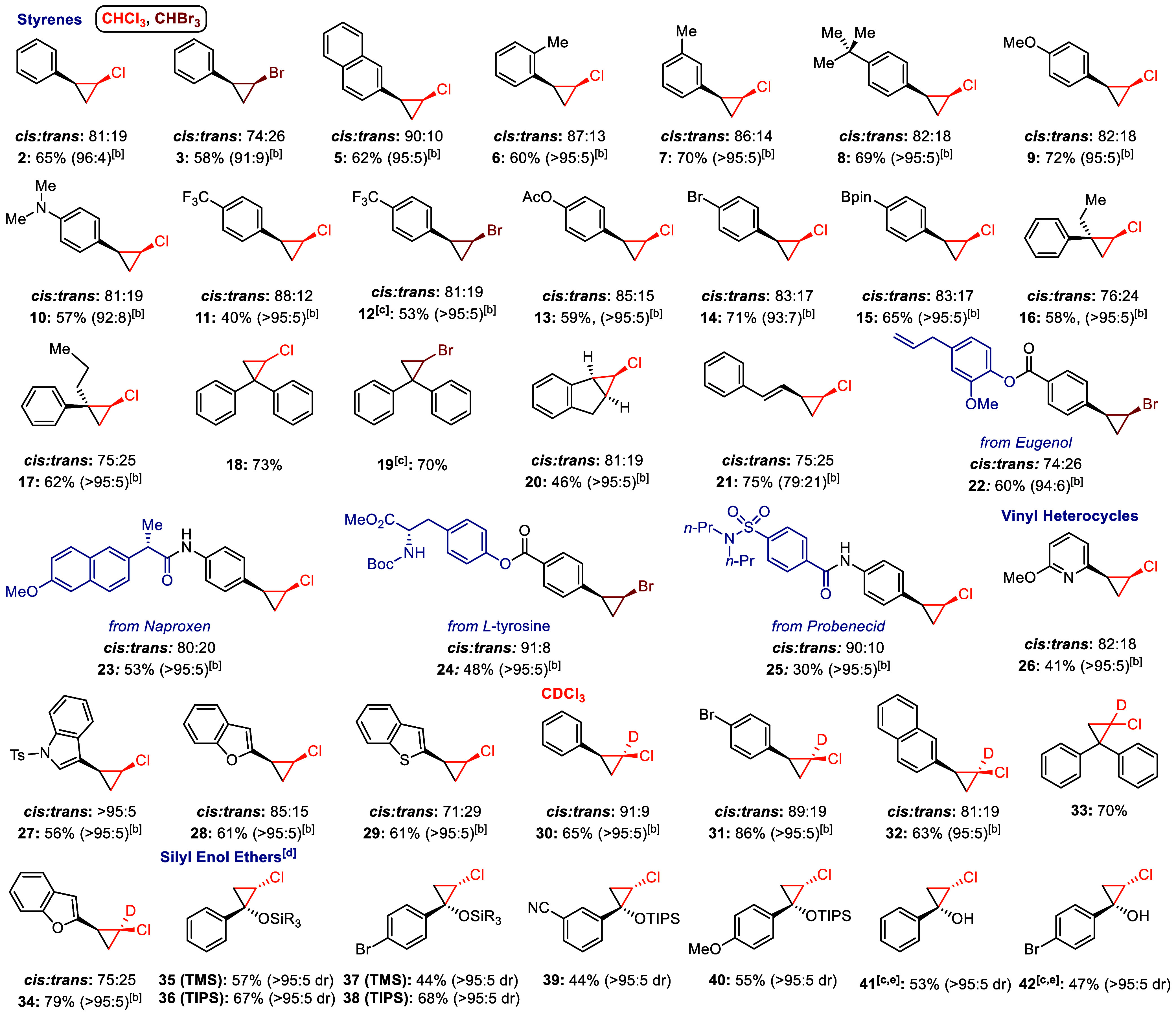
Substrate Scope for the Direct *cis*-Selective Halocyclopropanation of Alkenes^a^

a Yields of isolated, purified products after 8 h of irradiation using the optimized conditions (see General Procedures C1 and C2 in the Supporting Information). Cis:trans ratios were determined by ^1^H NMR analysis of the crude reaction mixture. ^b^Represents cis:trans ratio of the isolated, purified products. ^c^Reaction performed at 1.0 mmol scale (see General Procedure C3 in the Supporting Information). ^d^Diastereomeric ratios for silyl enol ether examples were determined by ^1^H NMR analysis of the crude reaction mixture. ^e^Deprotection conditions: cat. TMSCl, MeOH, 0°C, 10 min. Yields reported as isolated yields over two steps.

Next, we sought to determine the synthetic potential of this *cis*-selective halocyclopropanation of alkenes (Scheme 3). First, the reaction was optimized for a more synthetically relevant scale. Using 5 mmol of styrene (**1**) with CHBr_3_, 66% yield of **3** was obtained in high diastereoselectivity (*cis:trans* 83:17, Scheme 3A). Notably, the loading of both Vitamin B_12_ and Zn could be lowered without sacrificing reaction efficiency, and the amount of CHBr_3_ could be decreased to only 2 equiv by adding it dropwise over the course of the reaction. Next, we explored a series of synthetic manipulations of the *cis*-bromocyclopropane products. Using *in situ* generated lithium morpholide, bromocycloadduct **12** underwent efficient nucleophilic amination, giving the *trans*-2-arylcyclopropylamine **43**, a class of compounds known for their rich biological activity,^57^ in 62% yield and 92:8 dr (Scheme 3B). In the presence of excess potassium *tert*-butoxide, bromocyclopropane **19** could be converted into the corresponding cyclopropene (**44**), an important class of building blocks in organic synthesis (Scheme 3C).^58–60^ *cis*-Bromocyclopropane **3** readily underwent magnesium-bromide exchange with retention of configuration,^11^ which upon trapping with DMF yielded aldehyde **45** (Scheme 3D). Subsequently, the aldehyde was further elaborated to the alkene using a Wittig reaction to yield a common radical clock (**46**) in 42% yield over two steps.^61–63^ Finally, **3** could also be employed as a radical precursor under nucleophilic Vitamin B_12_-photocatalyzed conditions, and upon reaction with methyl acrylate was converted to Giese adduct **47** in 49% yield with good selectivity for the *trans*-isomer (dr 94:6).

**Scheme 3:**
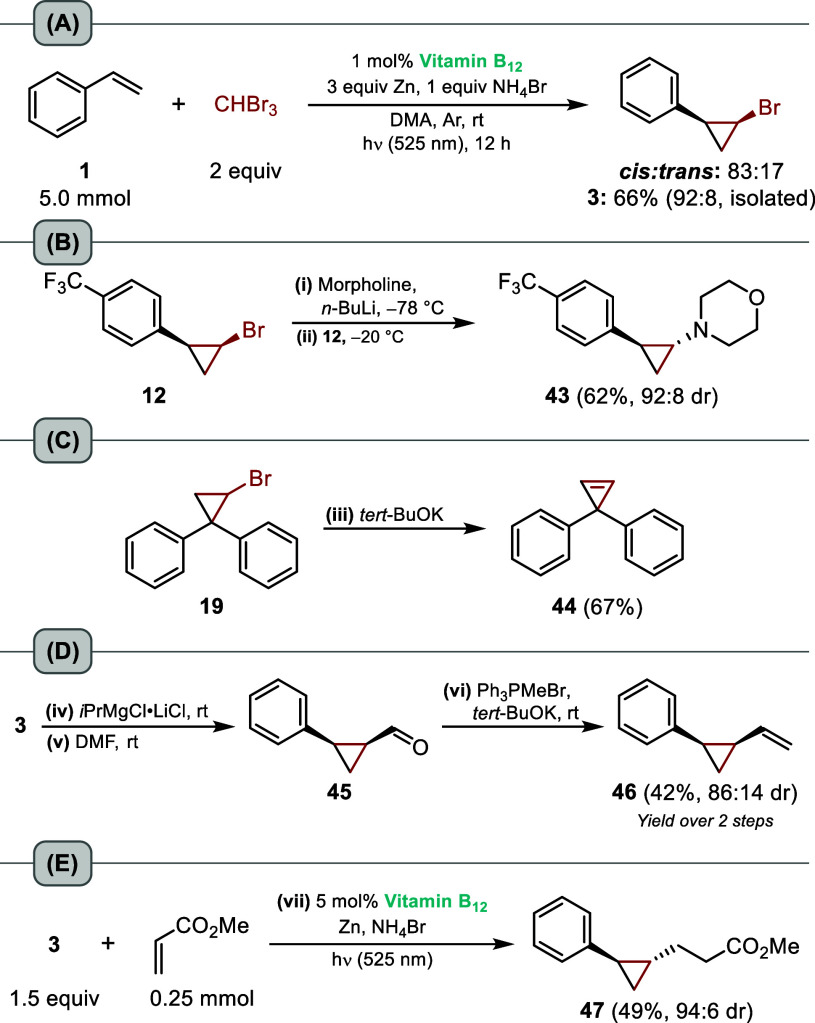
Synthetic Elaborations of Halocyclopropanes[Fn sch3-fn2]

a (A) 5 mmol scale halocyclopropantion. (B) Nucleophilic amination. (C) Cyclopropene synthesis. (D) Synthesis of radical clock. (E) Giese reaction. Conditions: (i) 1.1 equiv morpholine, 1.2 equiv *n*-BuLi, THF, – 78 °C to −20 °C. (ii) 11, THF, −20 °C. (iii) 4 equiv *tert*-BuOK, DMSO, rt, 24 h. (iv) 1.1 equiv *i*PrMgCl·LiCl, 1,4-dioxane, rt, 8 h. (v) DMF, rt, 4 h. (vi) 1.1 equiv Ph_3_PMeBr, 1.1 equiv *tert*-BuOK, THF, rt, 16 h. (vii) 5 mol % Vitamin B_12_, 3 equiv Zn, 1 equiv NH_4_Br, DMF, Ar, hν (525 nm), 30 °C, 16 h.

Having explored the reaction scope and synthetic utility of halocyclopropanes, we investigated the key steps of the reaction mechanism. Using UV–vis techniques,^40^ we were able to confirm the feasibility of both the generation of the catalytically active Co­(I) oxidation state and the subsequent S_N_2 reaction with both CHCl_3_ and CHBr_3_ (see SI). Next, we sought to probe the involvement of ^•^CHX_2_ radical intermediates. Upon subjecting radical clock **48** to our standard reaction conditions with CHCl_3_, the anticipated ring-opened/cyclized product **49** was observed in 7% yield and confirmed by HRMS analysis, supporting the intermediacy of ^•^CHCl_2_ radicals (Scheme 4A). Interestingly, the non-ring-opened chlorocyclopropyl adduct **50** was observed in 58% yield, which was also confirmed by HRMS analysis. It is also important to note that the corresponding dichlorocyclopropane adduct was not observed in the reaction, and a control study for the dehalogenation of **51** under standard conditions yielded only 12% yield of **2** with a low *cis*:*trans* selectivity (58:42) that was inconsistent our model reaction (Scheme 4B). Considering these data, dihalocyclopropanes are unlikely to be key reaction intermediates, ruling out the formation of carbenes under our standard conditions. To explain the formation of **50**, an analysis of the relevant rate constants is required, which are summarized in Scheme 4C. Upon ^•^CHCl_2_ radical generation, addition to radical clock **48** results in the formation of stabilized benzyl radical intermediate **III**. Given that ring-opening leads to the formation of unstabilized primary radical **IV**, the unimolecular rate constant for this reaction is estimated to be quite slow, on the order of 10^5^ s^–1^ (*k*_1_).^64^ Conversely, the Co­(II) oxidation state of Vitamin B_12_ and other related model cobalt complexes, which can be described as a persistent free radical,^65^ is known to react with other carbon-centered radicals to form Co­(III)–R species at bimolecular rate constants in the range of 10^7^ to 10^9^ M^–1^ s^–1^ (*k*_2_).^66,67^ Therefore, trapping of benzyl radical intermediate **III** by Co­(II) is proposed to lead to the formation of Co­(III)-bound intermediate **V**, which upon single-electron reduction leads to dicyclopropyladduct **50** through a polar 3-*exo*-*tet*. In good agreement, the involvement of intermediates of type **II** or **V** have long been invoked in Vitamin B_12_-photocatalyzed Giese reactions.^68,69^ Taken together, these data highlight both the radical-polar crossover nature of the halocyclopropanation, and the intimate involvement of Vitamin B_12_ leading up to the key 3-*exo*-*tet* step. We hypothesize that the formation and reduction of intermediate **II** plays a crucial role in setting the observed *cis*-selectivity of our Vitamin B_12_-photocatalyzed cyclopropanation reaction (Scheme 4D), as 3-*exo*-*tet* cyclizations occurring in solution (in the absence of Vitamin B_12_) are expected to be unselective based on previously reported radical-polar crossover approaches.^70–72^ Our group is actively working on developing a stereochemical model to rationalize the selectivity observed in Vitamin B_12_-catalyzed cyclopropanation reactions, and these results will be reported in due course.

**Scheme 4:**
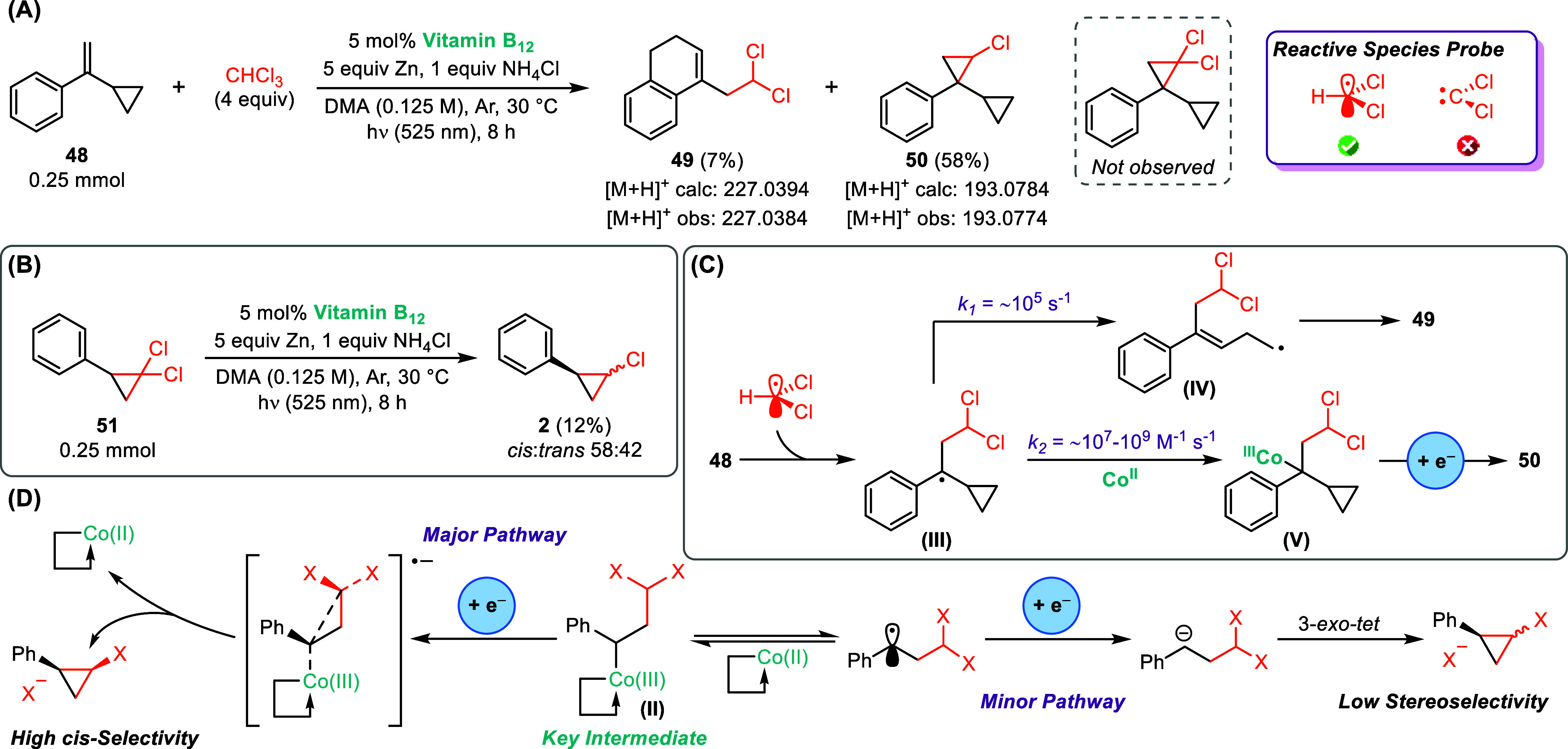
Mechanistic Studies and Proposed Halocyclopropanation Mechanism[Fn sch4-fn3]

a (A) Radical clock experiment. (B) Monodehalogenation control. (C) Proposed mechanistic pathway. (D) Proposed origin of stereoselectivity.

## Conclusions

In summary, we have developed a Vitamin B_12_-photocatalyzed approach for the direct halocyclopropanation of electron-rich alkenes using haloforms. The reaction proceeds with high *cis*-selectivity, providing efficient access to these traditionally difficult to prepare stereoisomers. Our protocol was compatible with a broad range of styrenes, vinyl heterocycles, and silyl enol ethers, and the method could also be applied for the chlorodeuterocyclopropanation of alkenes using readily available CDCl_3_. The reaction could be performed on 5 mmol scale, and the bromocyclopropyl adducts could be further derivatized into a range of high value products. Mechanistic studies suggested that Vitamin B_12_ plays a key role in the radical-polar crossover step, ultimately influencing the stereoselectivity. We anticipate our Vitamin B_12_-photocatalysis platform will find immediate applications in the synthesis of 1,2-disubstituted halocyclopropanes, as it provides a direct approach for the preparation of these valuable motifs that proceeds with complementary selectivity compared to known methods.

## Supplementary Material



## Data Availability

The data underlying this study are available in the published article and its Supporting Information.

## Notes

Experimental procedures, reaction optimization, compound characterization, and NMR spectra (PDF)
