# Supplementary material for: cis-Selective Direct Halocyclopropanation of Alkenes Mediated by Nucleophilic Cobalt Photocatalysis
Source: JACS Au. 2026 Jun 1;6(6):3356–62. doi: 10.1021/jacsau.6c00381 (PMC13291968; doi:10.1021/jacsau.6c00381)

Supporting Information

cis-Selective Direct Halocyclopropanation of Alkenes Mediated by Nucleophilic Cobalt Photocatalysis

John Hayford G. Teye-Kau, Martin Pauze and Spencer P. Pitre*

[a]Department of Chemistry, Oklahoma State University, Stillwater, OK 74078, United States

*Email: spencer.p.pitre@okstate.edu

Table of Contents

A. General Information 2
B. Pictures of Photochemistry Setup 3
C. Experimental Procedures 3
   1. General Procedure for Halocyclopropanations using $CHCl_3$ 3
   2. General Procedure for Halocyclopropanations using $CHBr_3$ 4
   3. General Procedure for 1.0 mmol scale Halocyclopropanations using $CHBr_3$ 4
   4. General Procedure for Activation of Zn Powder 5
   5. Synthesis of Starting Materials 5
      a. 1-Tosyl-1H-indole-3-carbaldehyde1 5
      b. Procedure for Preparation of Alkene Starting Materials 6
      c. Procedure for Preparation of Trimethylsilyl Enol Ethers 7
      d. Procedure for Preparation of Triisopropylsilyl Enol Ethers 8
      e. (2,2-Dichlorocyclopropyl)benzene (51)[10] 9
D. Full Reaction Optimization 10
E. Characterization of Reaction Products 11
F. Halocyclopropanation of Phenyl Vinyl Sulfone 37
G. Unsuccessful Substrates 37
H. Synthetic Elaborations 38
   1. 5 mmol Scale Halocyclopropanation 38
   2. Nucleophilic Amination 39
   3. Cyclopropene Synthesis 40
   4. Synthesis of Radical Clock 41
   5. Giese Reaction 42
I. Mechanistic Studies 44
   1. Radical Trap Experiment with TEMPO 44
   2. Radical Clock Experiment 45
   3. Monodehalogenation Control Reaction 46
   4. UV-Vis Studies 46
   5. Initial Rate Studies 48
J. References 50
K. NMR Spectra 52

A. General Information

All glassware used for the reactions were oven-dried and unless otherwise stated reactions were performed under argon atmosphere. All reagents and solvents were purchased from commercial suppliers (Sigma Aldrich, Thermo Fisher Scientific, Oakwood Chemicals, Ambeed, TCI America, Combi-Blocks Inc.) and were used as received unless otherwise noted. Vitamin B_{12} stands for cyanocobalamin. All photochemistry experiments were performed using a 40W Kessil PR160L 525 nm LED at 75% intensity in an EvoluChem Photobox from Hepatochem at 30 °C, unless otherwise stated. Thin-layer chromatography (TLC) was conducted with silica gel 60 F254 pre-coated plates (0.25 μm) and visualized by exposure to UV-light (254 nm) or staining with potassium permanganate ($KMnO_4$), thiocyanate, or Hanessian's stain. Flash column chromatography was performed using a Biotage Isolera Four equipped with Sorbtech Purity flash column cartridges (60 Å porosity, 40-75 μm). ^{1}H NMR spectra were recorded at 400 MHz or 800 MHz and reported relative to deuterated solvent signals. Data for ^{1}H NMR spectra are reported as follows: chemical shift (δ ppm), multiplicity, coupling constant (Hz), and integration. ^{13}C NMR spectra were recorded at 101 or 201 MHz. Data for ^{13}C NMR spectra are reported in terms of chemical shift. High-resolution mass spectra were obtained with a quadrupole-Orbitrap hybrid mass spectrometer at Oklahoma State University. IR spectra were recorded on a Shimadzu IRAffinity-1S FT-IR spectrophotometer equipped with a QATR 10 single reflectance ATR accessory and reported in terms of absorption frequency (cm^{-1}). UV-vis spectra were recorded on a Shimadzu UV-2600 UV-vis spectrophotometer.

B. Pictures of Photochemistry Setup

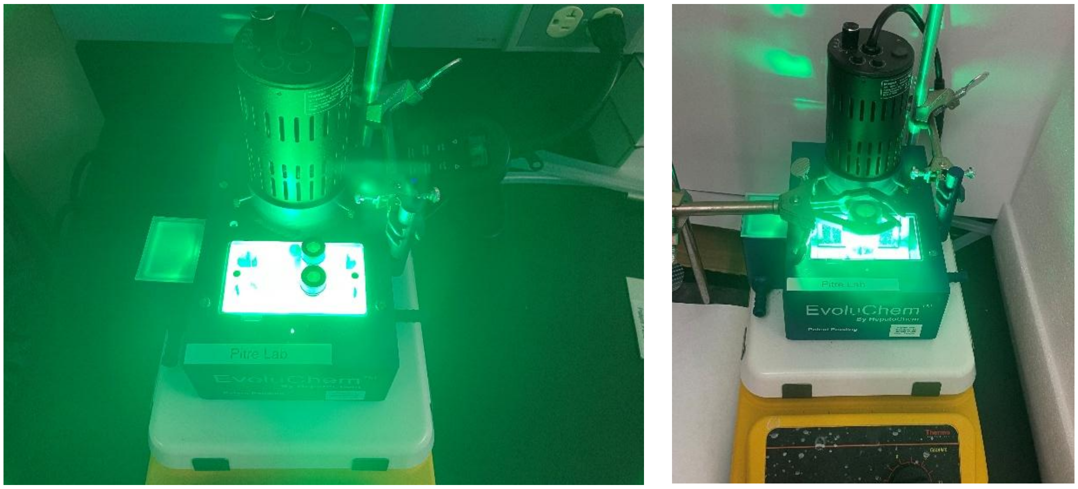

Figure S1. Picture of the photochemistry setup employed in this work for both the 0.25 mmol scale reactions (left) and 1.0 mmol scale reactions (right).

C. Experimental Procedures

1. General Procedure for Halocyclopropanations using $CHCl_3$

An oven-dried 1 dram vial with a pressure relief cap equipped with a magnetic stir bar was charged with Vitamin B_{12} (17 mg, 0.0125 mmol, 5 mol%), activated Zn powder (82 mg, 1.25 mmol, 5 equiv), and NH_4Cl (13 mg, 0.25 mmol, 1 equiv). Dry DMA (2 mL, 0.125 M, dried over 4 Å molecular sieves) was added, and the reaction mixture was degassed by sparging with argon for 10 minutes while stirring. $CHCl_3$ (80 µL, 1.0 mmol, 4 equiv) was added under argon, followed by the alkene (0.25 mmol, 1 equiv). The reaction mixture was then sonicated and irradiated with a Kessil PR160L 525 nm LED at 75% intensity in an EvoluChem Photobox for 8 h. The reaction mixture was filtered through a cotton plug, transferred into a separatory funnel with 20 mL of anhydrous Et_2O, and washed with saturated aqueous NH_4Cl (20 mL) and brine (3 x 20 mL). The combined aqueous phases were extracted with anhydrous Et_2O (20 mL), and the combined organic phases were dried over $MgSO_4$ and concentrated. The purified product was obtained by flash

column chromatography using a Biotage Isolera Four. Yields were reported as isolated yields of purified products.

2. General Procedure for Halocyclopropanations using $CHBr_3$

An oven-dried 1 dram vial with pressure relief cap equipped with a magnetic stir bar was charged with Vitamin B_{12} (17 mg, 0.0125 mmol, 5 mol%), activated Zn powder (82 mg, 1.25 mmol, 5 equiv), and NH_4Br (25 mg, 0.25 mmol, 1 equiv). Dry DMA (2 mL, 0.125 M, dried over 4 Å molecular sieves) was added, and the reaction mixture was degassed by sparging with argon for 10 minutes while stirring. $CHBr_3$ (87 µL, 1.0 mmol, 4 equiv) was added under argon, followed by the alkene (0.25 mmol, 1 equiv). The reaction mixture was then sonicated and irradiated with a Kessil PR160L 525 nm LED at 75% intensity in an EvoluChem Photobox for 8 h. The reaction mixture was filtered through a cotton plug, transferred into a separatory funnel with 20 mL of anhydrous Et_2O, and washed with saturated aqueous NH_4Cl (20 mL) and brine (3 x 20 mL). The combined aqueous phases were extracted with anhydrous Et_2O (20 mL), and the combined organic phases were dried over $MgSO_4$ and concentrated. The purified product was obtained by flash column chromatography using a Biotage Isolera Four. Yields were reported as isolated yields of purified products.

3. General Procedure for 1.0 mmol scale Halocyclopropanations using $CHBr_3$

An oven-dried 20 mL vial with a pressure relief cap equipped with a magnetic stir bar was charged with Vitamin B_{12} (68 mg, 0.05 mmol, 5 mol%), activated Zn powder (327 mg, 5.0 mmol, 5 equiv), and NH_4Br (98 mg, 1.0 mmol, 1 equiv). Dry DMA (8 mL, 0.125 M, dried over 4 Å molecular sieves) was added and the reaction mixture was degassed by sparging with argon for 10 minutes while stirring. $CHBr_3$ (350 µL, 4.0 mmol, 4 equiv) was added under argon, followed by the alkene (1.0 mmol, 1 equiv). The reaction mixture was then sonicated and irradiated with a Kessil PR160L

525 nm LED at 75% intensity in an EvoluChem Photobox for 8 h. The reaction mixture was filtered through a cotton plug, transferred into a separatory funnel with 60 mL of anhydrous Et_2O, and washed with saturated aqueous NH_4Cl (60 mL) and brine (3 x 60 mL). The combined aqueous phases were extracted with anhydrous Et_2O (60 mL), and the combined organic phases were dried over $MgSO_4$ and concentrated. The purified product was obtained by flash column chromatography using a Biotage Isolera Four. Yields were reported as isolated yields of the purified products.

4. General Procedure for Activation of Zn Powder

To 5 g of Zn powder in a round-bottom flask equipped with a magnetic stir bar, 10% aqueous HCl (50 mL) was added. This was stirred vigorously for 5 minutes, and the suspension was filtered and washed successively with water (50 mL), acetone (50 mL), methanol (50 mL), and then ether (50 mL). The collected Zn powder was ground in a mortar and pestle, transferred into a glass vial, and dried in vacuo. The activated Zn powder was stored in a dry box and could be used without any loss in reactivity for around 1 month.

5. Synthesis of Starting Materials

a. 1-Tosyl-1H-indole-3-carbaldehyde1

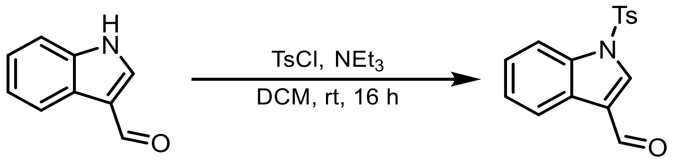

An oven-dried round-bottom flask equipped with a magnetic stir bar was charged with 1*H*-indole-3-carbaldehyde (1.45 g 10 mmol, 1 equiv), triethylamine (2.80 mL, 20 mmol, 2 equiv), and DCM (20 mL, 0.50 M). The mixture was cooled to 0 °C and stirred for 15 minutes. 4-Toluenesulfonyl chloride (2.10 g, 11 mmol, 1.1 equiv) was added to the mixture and stirred at room temperature

overnight (16 h). The crude was diluted with 20 mL DCM and washed saturated aqueous NH_4Cl (50 mL), saturated aqueous $NaHCO_3$ (50 mL) and brine (50 mL). The organic phase was dried with $MgSO_4$, filtered and concentrated under vacuum to afford the title compound as a purple solid (2.77g, 93%). Spectral data matched that previously reported.[1] This aldehyde product was used to synthesize the corresponding alkene without further purification.

b. Procedure for Preparation of Alkene Starting Materials

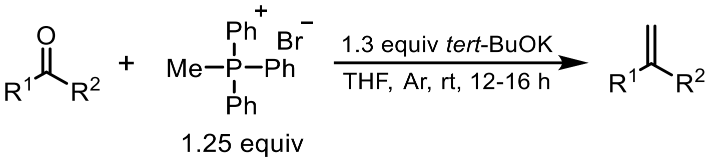

An oven-dried 100 mL round-bottom flask equipped with a magnetic stir bar was charged with methyltriphenylphosphonium bromide (1.79 g, 5 mmol, 1.25 equiv), and potassium *tert*-butoxide (0.58 g, 5 mmol, 1.3 equiv) and capped with a septum. The component of the flask was evacuated and backfilled with Ar, and anhydrous THF (11 mL) was added, and the mixture was stirred at room temperature for 1 hour under Ar. The ketone/aldehyde (4.00 mmol, 1 equiv) in 5 mL anhydrous THF was added dropwise over 5 minutes with a syringe. The reaction was monitored by TLC until full conversion was achieved (12-16 h). The reaction mixture was quenched with saturated aqueous NH_4Cl (35 mL) and then extracted with Et_2O (3 x 40 mL). The combined organic phase was washed with brine (2 x 50 mL), dried with $MgSO_4$, filtered and concentrated under vacuum. The purified product was obtained by flash column chromatography using a Biotage Isolera Four to afford the corresponding alkene product. Spectral data matched those previously reported.[1–6]

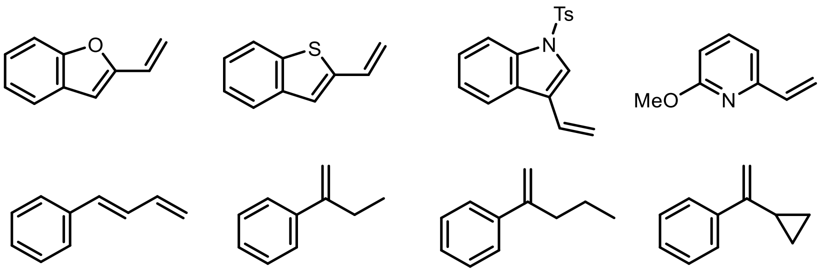

Figure S2. Alkenes synthesized for this work.

c. Procedure for Preparation of Trimethylsilyl Enol Ethers

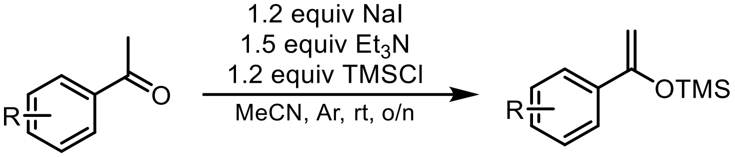

An oven-dried 100 mL round-bottom flask equipped with a stir bar was charged with ketone (10 mmol, 1 equiv) and sodium iodide (1.8 g, 12 mmol, 1.2 equiv) and capped with a septum. The flask was then evacuated and backfilled with Ar, and 15 mL of dry MeCN was added, and the reaction was stirred at room temperature for 5 minutes. Et_3N (2.1 mL, 15 mmol, 1.5 equiv) and TMSCl (1.5 mL, 12 mmol, 1.2 equiv) were added, and the mixture was stirred overnight under argon at room temperature. The reaction mixture was cooled to 0 °C and quenched with a mixture of petroleum ether (50 mL) and saturated $NH_4Cl_{(aq)}$ (50 mL) previously cooled to 0 °C. The organic phase was separated, and the aqueous phase was extracted twice with 30 mL petroleum ether and the combined organic phase was washed with 50 mL ice water, 50 mL saturated $NH_4Cl_{(aq)}$ and dried over $MgSO_4$ and concentrated under vacuum.[7] The purified product was obtained by flash column chromatography using a Biotage Isolera Four to afford the corresponding silyl enol ether. Spectral data matched those previously reported.[7]

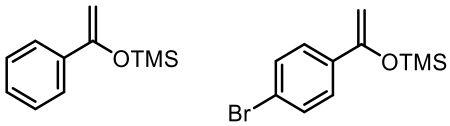

Figure S3. Trimethylsilyl enol ethers synthesized for this work.

d. Procedure for Preparation of Triisopropylsilyl Enol Ethers

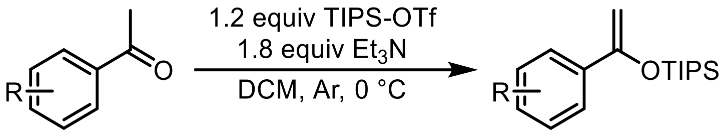

An oven-dried 100 mL round-bottom flask equipped with a stir bar was charged with ketone (5 mmol, 1 equiv), Et_3N (1.3 mL, 9 mmol, 1.8 equiv) and capped with a septum. The component of the flask was evacuated and backfilled with Ar, and 20 mL dry DCM was added and the reaction mixture stirred at room temperature for 15 minutes. The reaction mixture then cooled to 0 °C and TIPS-OTf (1.7 mL, 9.00 mmol, 1.2 equiv) was added dropwise over 3 minutes under Ar. The reaction mixture was allowed to stir at 0 °C for 50 minutes until full conversion was observed by TLC. The mixture was quenched with saturated aqueous $NaHCO_3$ (20 mL) and diluted with a cooled DCM (10 mL). The organic layer was washed twice with cooled saturated aqueous $NaHCO_3$ (20 mL), dried over $MgSO_4$ and concentrated under vacuum.[8]The purified product was obtained by flash column chromatography using a Biotage Isolera Four and a Et_3N-treated flash column cartridge to afford the corresponding silyl enol ether. Spectral data matched those previously reported.[7–9]

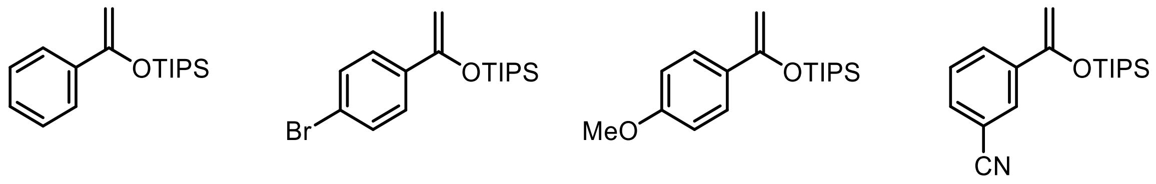

Figure S4. Triisopropylsilyl enol ethers synthesized for this work.

e. (2,2-Dichlorocyclopropyl)benzene (51)[10]

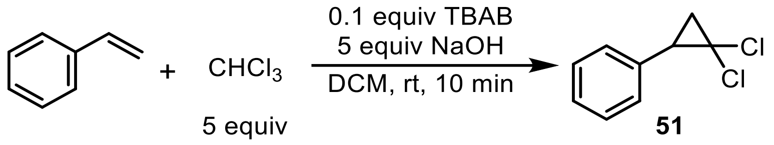

An oven-dried 50 mL round-bottom flask equipped with a magnetic stir bar was charged with $CHCl_3$ (4.8 mL, 60 mmol, 5 equiv), tetrabutylammonium bromide (387 mg, 1.2 mmol, 0.1 equiv), NaOH (2.4 mg, 60 mmol, 5 equiv), and DCM (6 mL). The reaction mixture was stirred at room temperature, and styrene (1.4 mL, 12 mmol, 1 equiv) was added while stirring. After 10 minutes, the reaction mixture was quenched with saturated aqueous NH_4Cl (15 mL) and extracted with EtOAc (2 x 15 mL). The combined organic layers were dried over $MgSO_4$ and concentrated. The purified product was obtained by flash column chromatography using a Biotage Isolera Four using pentane as the eluent, affording the title compound as a colorless oil in 18% yield (410 mg). The spectral data matched that previously reported.[10]

D. Full Reaction Optimization

Table S1. Optimization reactions for the chlorocyclopropanation of styrene.[a]

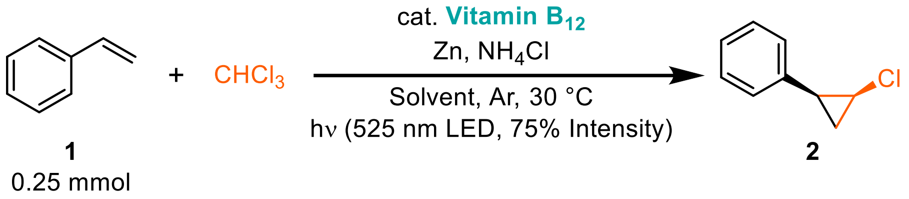

equiv. $CHCl_3$	mol % VB_{12}	equiv. Zn	equiv. NH_4Cl	Solvent (Conc.)	Time	% Yield of 2	*cis*:*trans*
1.5	5.0	2.0	1.0	DMF (0.125 M)	O/N	45%	76:24
1.5	5.0	2.0	2.0	DMF (0.125 M)	O/N	33%	71:29
1.5	5.0	3.0	1.0	DMF (0.125 M)	O/N	42%	81:19
1.5	5.0	3.0	1.0	DMA (0.125 M)	O/N	46%	83:17
1.5	5.0	3.0	1.0	MeCN (0.125 M)	O/N	29%	63:37
1.5	5.0	3.0	1.0	3:1 MeCN:H_2O (0.125 M)	O/N	36%	77:23
1.5	5.0	3.0	2.0	DMF (0.125 M)	O/N	30%	77:23
1.5	5.0	4.0	1.0	DMF (0.125 M)	O/N	48%	82:18
1.5	5.0	5.0	1.0	DMF (0.125 M)	O/N	52%	86:14
3.0	5.0	3.0	1.0	DMF (0.125 M)	O/N	45%	74:26
3.0	5.0	5.0	1.0	DMF (0.125 M)	O/N	57%	79:21
3.0	5.0	5.0	2.0	DMF (0.125 M)	O/N	32%	78:22
4.0	5.0	5.0	1.0	DMF (0.125 M)	O/N	50%	80:20
4.0	5.0	5.0	1.0	DMA (0.125 M)	O/N	64%[b]	84:16
4.0	4.0	5.0	1.0	DMA (0.125 M)	O/N	41%	79:21
4.0	3.0	5.0	1.0	DMA (0.125 M)	O/N	59%	83:17
4.0	2.0	5.0	1.0	DMA (0.125 M)	O/N	59%	82:18
4.0	1.0	5.0	1.0	DMA (0.125 M)	O/N	39%	77:23
4.0	5.0	5.0	1.0	DMA (0.125 M)	O/N	65%	82:18
4.0	5.0	5.0	1.0	DMA (0.125 M)	1.5 h	56%	80:20
4.0	5.0	5.0	1.0	DMA (0.125 M)	2.0 h	56%	81:19
4.0	5.0	5.0	1.0	DMA (0.125 M)	4.0 h	57%	80:20
4.0	5.0	5.0	1.0	DMA (0.125 M)	6.0 h	64%	86:14

[a]Yields were determined by ^{1}H NMR using 1,3,5-trimethoxybenzene as an external standard. [b]Reaction was run at 100% LED intensity. O/N: overnight.

E. Characterization of Reaction Products

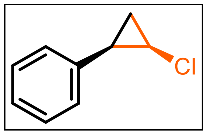

***cis*-(2-Chlorocyclopropyl)benzene (2).** Prepared according to General Procedure C1 using styrene (29 µL, 0.25 mmol, 1 equiv), $CHCl_3$ (80 µL, 1.0 mmol, 4 equiv), Vitamin B_{12} (17 mg, 0.0125 mmol, 5 mol%), activated Zn powder (82 mg, 1.25 mmol, 5 equiv) and NH_4Cl (13 mg, 0.25 mmol, 1 equiv) in 2 mL of dry DMA. The crude reaction mixture was analyzed by ^1H NMR to determine the *cis*:*trans* ratio (81:19). The crude material was purified by flash column chromatography (100% Hex) to afford the title compound as a colorless oil in 65% yield (25 mg) with a dr ratio of 96:4. Spectral data are in accordance with those reported in the literature.[11]

^{1}H NMR: (800 MHz, $CDCl_3$) δ 7.34-7.26 (m, 5H), 3.39 (td, J = 7.4, 4.3 Hz, 1H), 2.36 (dt, J = 9.6, 7.5 Hz, 1H), 1.49-1.46 (m, 1H), 1.26 (m, 1H). **^{13}C{^{1}H} NMR:** (200 MHz, $CDCl_3$) δ 136.1, 129.3, 128.0, 126.8, 34.5, 22.8, 14.2. **Rf:** 0.78 (4:1 Hex:EtOAc).

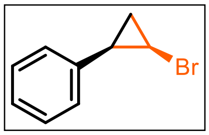

***cis*-(2-Bromocyclopropyl)benzene (3).** Prepared according to the General Procedure C2 using styrene (29 µL, 0.25 mmol, 1 equiv), $CHBr_3$ (87 µL, 1.0 mmol, 4 equiv), Vitamin B_{12} (17 mg, 0.0125 mmol, 5 mol%), activated Zn powder (82 mg, 1.25 mmol, 5 equiv) and NH_4Br (25 mg, 0.25 mmol, 1 equiv) in 2 mL of dry DMA. The crude reaction mixture was analyzed by ^1H NMR to determine the *cis*:*trans* ratio (74:26). The crude material was purified by flash column chromatography (100% Hex) to afford the title compound as a colorless oil in 58% yield (28.5 mg) with a dr ratio of 91:9. Spectral data are in accordance with those reported in the literature.[11]

^{1}H NMR: (800 MHz, $CDCl_3$) δ 7.37-7.35 (m, 2H), 7.31-7.27 (m, 3H), 3.34 (td, J = 7.6, 4.6 Hz, 1H), 2.35 (dt, J = 9.5, 7.6 Hz, 1H), 1.61 (m, 1H), 1.36 (m, 1H). **^{13}C{^{1}H} NMR:** (200 MHz, $CDCl_3$) δ 137.2, 129.2, 128.0, 126.8, 24.1, 22.1, 14.2. **Rf:** 0.83 (4:1 Hex:EtOAc).

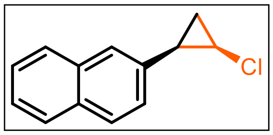

***cis*-2-(2-Chlorocyclopropyl)naphthalene (5).** Prepared according to General Procedure C1 using 2-vinylnaphthalene (39 µL, 0.25 mmol, 1 equiv), $CHCl_3$ (80 µL, 1.0 mmol, 4 equiv), Vitamin B_{12} (17 mg, 0.0125 mmol, 5 mol%), activated Zn powder (82 mg, 1.25 mmol, 5 equiv) and NH_4Cl (13 mg, 0.25 mmol, 1 equiv) in 2 mL of dry DMA. The crude reaction mixture was analyzed by ^{1}H NMR to determine the *cis*:*trans* ratio 90:10). The crude material was purified by flash column chromatography (0 → 25% EtOAc in Hex) to afford the title compound as a colorless oil in 62% yield (31 mg) with a dr ratio of 95:5. Spectral data are in accordance with those reported in the literature.[12]

^{1}H NMR: (400 MHz, $CDCl_3$) δ 7.86-7.78 (m, 3H), 7.70 (s, 1H), 7.50-7.38 (m, 3H), 3.46 (td, J = 7.4, 4.3 Hz, 1H), 2.52 (m, 1H), 1.60-1.50 (m, 1H) 1.41 (ddd, J = 7.6, 6.7, 4.3 Hz, 1H). **^{13}C{^{1}H} NMR:** (101 MHz, $CDCl_3$) δ 133.9, 133.3, 132.6, 127.9, 127.9, 127.8, 127.6, 126.1, 125.7, 34.7, 23.2, 14.4. **Rf:** 0.68 (4:1 Hex:EtOAc).

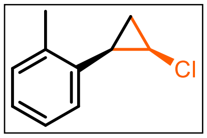

***cis*-1-(2-Chlorocyclopropyl)-2-methylbenzene (6).** Prepared according to General Procedure C1 using 1-methyl-2-vinylbenzene (32 µL, 0.25 mmol, 1 equiv), $CHCl_3$ (80 µL, 1.0 mmol, 4 equiv), Vitamin B_{12} (17 mg, 0.0125 mmol, 5 mol%), activated Zn powder (82 mg, 1.25 mmol, 5 equiv) and NH_4Cl (13 mg, 0.25 mmol, 1 equiv) in 2 mL of dry DMA. The crude reaction mixture was analyzed by ^{1}H NMR to determine the *cis*:*trans* ratio (87:13). The crude material was purified by flash column chromatography (0 → 25% EtOAc in Hex) to afford the title compound as a colorless oil in 60% yield (25 mg) with a dr ration of >95:5.

^{1}H NMR: (400 MHz, $CDCl_3$) δ 7.24-7.12 (m, 4H), 3.46 (td, J = 7.3, 4.0 Hz, 1H), 2.40 (s, 3H), 2.27 (dt, J = 9.5, 7.6 Hz, 1H), 1.52-1.39 (m, 1H), 1.35-1.25 (m, 1H). **^{13}C{^{1}H} NMR:** (101 MHz, $CDCl_3$) δ 139.0, 134.8, 129.7, 129.0, 127.2, 125.7, 33.7, 21.8, 19.8, 13.6. **Rf:** 0.77 (4:1 Hex:EtOAc). **HRMS (ESI) *m/z*:** $[M+H]^+$ calcd for $C_{10}H_{11}Cl$ 167.0627; found 167.0623. **IR (neat, cm^{-1}):** 3017, 2957, 2859, 1490, 1478, 1382, 1259, 1220, 1033, 786, 751, 730, 657.

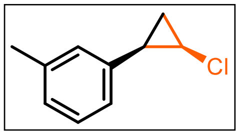

***cis*-1-(2-Chlorocyclopropyl)-3-methylbenzene (7).** Prepared according to General Procedure C1 using 1-methyl-3-vinylbenzene (33 µL, 0.25 mmol, 1 equiv), $CHCl_3$ (80 µL, 1.0 mmol, 4 equiv), Vitamin B_{12} (17 mg, 0.0125 mmol, 5 mol%), activated Zn powder (82 mg, 1.25 mmol, 5 equiv) and NH_4Cl (13 mg, 0.25 mmol, 1 equiv) in 2 mL of dry DMA. The crude reaction mixture was analyzed by ^{1}H NMR to determine the *cis*:*trans* ratio (86:14). The crude material was purified by flash column chromatography (0 → 25% EtOAc in Hex) to afford the title compound as a colorless oil in 70% yield (29 mg) with a dr ratio of >95:5.

^{1}H NMR: (400 MHz, $CDCl_3$) δ 7.19-7.25 (m, 1H), 7.11-7.02 (m, 3H), 3.37 (td, J = 7.4, 4.3 Hz, 1H), 2.61-2.14 (m, 4H), 1.51-1.39 (m, 1H), 1.25 (ddd, J = 7.7, 6.7, 4.3 Hz, 1H). **^{13}C{^{1}H} NMR:** (101 MHz, $CDCl_3$) δ 137.7, 136.1, 130.2, 128.0, 127.7, 126.3, 34.5, 22.9, 21.6, 14.2. **Rf:** 0.8 (4:1 Hex:EtOAc). **HRMS (ESI) *m/z*:** $[M+H]^+$ calcd for $C_{10}H_{11}Cl$ 167.0627; found 167.0623. **IR (neat, cm^{-1}):** 3025, 2958, 2962, 2856, 1607, 1490, 1457, 1434, 1237, 1217, 797, 757, 718.

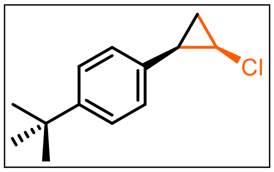

***cis*-1-(*tert*-Butyl)-4-(2-chlorocyclopropyl)benzene (8).** Prepared according to General Procedure C1 using 1-(*tert*-butyl)-4-vinylbenzene (46 µL, 0.25 mmol, 1 equiv), $CHCl_3$ (80 µL, 1.0 mmol, 4 equiv), Vitamin B_{12} (17 mg, 0.0125 mmol, 5 mol%), activated Zn powder (82 mg, 1.25 mmol, 5 equiv) and NH_4Cl (13 mg, 0.25 mmol, 1 equiv) in 2 mL of dry DMA. The crude reaction mixture was analyzed by ^{1}H NMR to determine

the *cis*:*trans* ratio (82:18). The crude material was purified by flash column chromatography (0 → 25% EtOAc in Hex) to afford the title compound as a colorless oil in 69% yield (36 mg) with a dr ratio of >95:5. Spectral data are in accordance with those reported in the literature.[12]

^{1}H NMR: (400 MHz, $CDCl_3$) δ 7.38-7.33 (m, 2H), 7.22-7.17 (m, 2H), 3.38 (td, *J* = 7.4, 4.4 Hz, 1H), 2.32 (m, 1H), 1.51-1.42 (m, 1H), 1.33 (s, 9H), 1.22 (ddd, *J* = 7.6, 6.7, 4.4 Hz, 1H). **^{13}C{^{1}H} NMR:** (101 MHz, $CDCl_3$) δ 149.7, 133.2, 129.0, 125.0, 34.7, 34.6, 31.5, 31.5, 31.4, 22.5, 14.4. **Rf:** 0.89 (4:1 Hex:EtOAc).

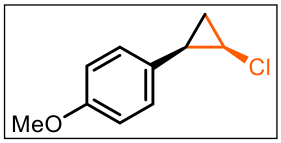

***cis*-1-(2-Chlorocyclopropyl)-4-methoxybenzene (9).** Prepared according to General Procedure C1 using 1-methoxy-4-vinylbenzene (33 µL, 0.25 mmol, 1 equiv), $CHCl_3$ (80 µL, 1.0 mmol, 4 equiv), Vitamin B_{12} (17 mg, 0.0125 mmol, 5 mol%), activated Zn powder (82 mg, 1.25 mmol, 5 equiv) and NH_4Cl (13 mg, 0.25 mmol, 1 equiv) in 2 mL of dry DMA. The crude reaction mixture was analyzed by ^{1}H NMR to determine the *cis*:*trans* ratio (82:18). The crude material was purified by flash column chromatography (0 → 25% EtOAc in Hex) to afford the title compound as a colorless oil in 72% yield (33 mg) with a dr ratio of 95:5. Spectral data are in accordance with those reported in the literature.[12]

^{1}H NMR: (400 MHz, $CDCl_3$) δ 7.24-7.14 (m, 2H), 6.91-6.80 (m, 2H), 3.81 (s, 3H), 3.35 (td, *J* = 7.3, 4.2 Hz, 1H), 2.30 (dt, *J* = 9.6, 7.5 Hz, 1H), 1.44 (dt, *J* = 9.7, 7.0 Hz, 1H), 1.18 (ddd, *J* = 7.5, 6.6, 4.2 Hz, 1H). **^{13}C{^{1}H} NMR:** (101 MHz, $CDCl_3$) δ 158.5, 130.4, 128.2, 113.6, 55.3, 34.5, 22.2, 14.2. **Rf:** 0.63 (4:1 Hex:EtOAc).

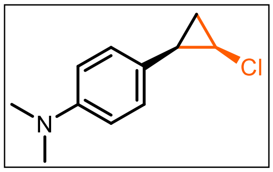

***cis*-4-(2-Chlorocyclopropyl)-*N,N*-dimethylaniline (10).** Prepared according to General Procedure C1 using *N,N*-dimethyl-4-vinylaniline (38 µL, 0.25 mmol, 1 equiv), $CHCl_3$ (80 µL, 1.0 mmol, 4 equiv), Vitamin B_{12} (17 mg, 0.0125 mmol,

5 mol%), activated Zn powder (82 mg, 1.25 mmol, 5 equiv) and NH_4Cl (13 mg, 0.25 mmol, 1 equiv) in 2 mL of dry DMA. The crude reaction mixture was analyzed by ^1H NMR to determine the *cis*:*trans* ratio (81:19). The crude material was purified by flash column chromatography (0 → 25% EtOAc in Hex) to afford the title compound as a pale-yellow oil in 57% yield (28 mg) with a dr ratio of 92:8.

^{1}H NMR: (400 MHz, $CDCl_3$) δ 7.16-7.11 (d, *J* = 8.7, 2H), 6.75-6.69 (d, *J* = 8.7, 2H), 3.33 (td, *J* = 7.3, 4.2 Hz, 1H), 2.94 (s, 6H), 2.27 (dt, *J* = 9.7, 7.4 Hz, 1H), 1.41 (dt, *J* = 9.7, 6.9 Hz, 1H), 1.16 (ddd, *J* = 7.4, 6.6, 4.2 Hz, 1H). **^{13}C{^{1}H} NMR:** (101 MHz, $CDCl_3$) δ 149.6, 130.1, 123.9, 112.4, 40.8, 34.9, 22.1, 14.1. **Rf:** 0.64 (4:1 Hex:EtOAc). **HRMS (ESI) *m/z*:** $[M+H]^+$ calcd for $C_{11}H_{14}ClN$ 196.0893; found 196.0888. **IR (neat, cm^{-1}):** 2987, 2927, 2885, 2848, 2801, 1616, 1522, 1480, 1444, 1349, 1219, 1160, 948, 814, 650.

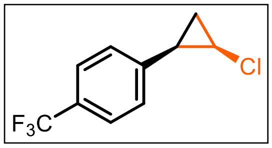

***cis*-1-(2-Chlorocyclopropyl)-4-(trifluoromethyl)benzene (11).** Prepared according to General Procedure C3 using 1-(trifluoromethyl)-4-vinylbenzene (37 μL, 0.25 mmol, 1 equiv), $CHCl_3$ (80 μL, 1.0 mmol, 4 equiv), Vitamin B_{12} (17 mg, 0.0125 mmol, 5 mol%), activated Zn powder (82 mg, 1.25 mmol, 5 equiv) and NH_4Cl (13 mg, 0.25 mmol, 1 equiv) in 2 mL of dry DMA. The crude reaction mixture was analyzed by ^1H NMR to determine the *cis*:*trans* ratio (88:12). The crude material was purified by flash column chromatography (0 → 25% EtOAc in Hex) to afford the title compound as a colorless oil in 40% yield (22 mg) with a dr ratio of >95:5. Spectral data are compared to the *trans*-isomer reported in the literature.[13]

^{1}H NMR: (400 MHz, $CDCl_3$) δ 7.62-7.54 (d, *J* = 8.1, 2H), 7.40-7.31 (d, *J* = 8.1, 2H), 3.43 (td, *J* = 7.4, 4.4 Hz, 1H), 2.40 (dt, *J* = 9.5, 7.6 Hz, 1H), 1.60-1.49 (m, 1H), 1.30 (ddd, *J* = 7.6, 6.9, 4.4 Hz,

1H). **^{13}C{^{1}H} NMR:** (101 MHz, $CDCl_3$) δ 140.4, 129.7, 129.2, 128.9, 125.7, 125.1, 125.0, 125.1, 125.0, 123.1, 34.4, 22.8, 14.8. **Rf:** 0.68 (4:1 Hex:EtOAc).

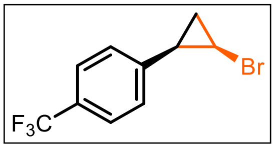

***cis*-1-(2-Bromocyclopropyl)-4-(trifluoromethyl)benzene (12).** Prepared according to General Procedure C2 using 1-(trifluoromethyl)-4-vinylbenzene (148 µL, 1.00 mmol, 1 equiv), $CHBr_3$ (350 µL, 4.0 mmol, 4 equiv), Vitamin B_{12} (68 mg, 0.05 mmol, 5 mol%), activated Zn powder (327 mg, 5.0 mmol, 5 equiv) and NH_4Br (98 mg, 1.0 mmol, 1 equiv) in 2 mL of dry DMA. The crude reaction mixture was analyzed by ^{1}H NMR to determine the *cis*:*trans* ratio (81:19). The crude material was purified by flash column chromatography (0 → 25% EtOAc in Hex) to afford the title compound as a colorless oil in 53% yield (35 mg) with a dr ratio of >95:5. Spectral data are in accordance with those reported in the literature.[14]

^{1}H NMR: (400 MHz, $CDCl_3$) δ 7.59 (d, J = 8.1 Hz, 2H), 7.35 (d, J = 8.1 Hz, 2H), 3.35 (td, J = 7.6, 4.6 Hz, 1H), 2.37 (q, J = 8.0 Hz, 1H), 1.66 (dt, J = 9.4, 7.2 Hz, 1H), 1.37 (td, J = 7.2, 4.6 Hz, 1H). **^{13}C{^{1}H} NMR:** (201 MHz, $CDCl_3$) δ 141.5, 129.6, 129.2, 129.1, 125.1, 125.0, 125.0, 125.0, 23.6, 22.1, 14.8. **Rf:** 0.68 (4:1 Hex:EtOAc).

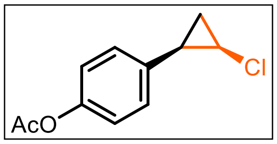

***cis*-4-(2-Chlorocyclopropyl)phenyl acetate (13).** Prepared according to General Procedure C1 using 4-vinylphenyl acetate (33 µL, 0.25 mmol, 1 equiv), $CHCl_3$ (80 µL, 1.0 mmol, 4 equiv), Vitamin B_{12} (17 mg, 0.0125 mmol, 5 mol%), activated Zn powder (82 mg, 1.25 mmol, 5 equiv) and NH_4Cl (13 mg, 0.25 mmol, 1 equiv) in 2 mL of dry DMA. The crude reaction mixture was analyzed by ^{1}H NMR to determine the *cis*:*trans* ratio (85:15). The crude material was purified by flash column chromatography (0 → 25% EtOAc in Hex) to afford the title compound as a colorless oil in 59% yield (31 mg) with a dr ratio of >95:5.

^{1}H NMR: (400 MHz, $CDCl_3$) δ 7.32-7.21 (m, 2H), 7.10-7.00 (m, 2H), 3.38 (td, *J* = 7.4, 4.3 Hz, 1H), 2.30 (m, 4H), 1.49 (ddd, *J* = 9.6, 7.3, 6.7 Hz, 1H), 1.22 (ddd, *J* = 7.6, 6.8, 4.3 Hz, 1H). **$^{13}C\{^1H\}$ NMR:** (101 MHz, $CDCl_3$) δ 169.6, 149.6, 133.8, 130.4, 121.2, 34.4, 22.4, 21.3, 14.6. **Rf:** 0.49 (4:1 Hex:EtOAc). **HRMS (ESI) *m/z*:** $[M+H]^+$ calcd for $C_{11}H_{11}ClO_2$ 211.0526; found 211.0521. **IR (neat, cm^{-1}):** 3051, 3009, 1753, 1715, 1507, 1436, 1367, 1285, 1214, 1188, 1165, 1043, 1054, 912, 845, 807, 655, 623.

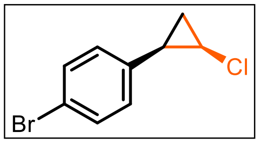

***cis*-1-Bromo-4-(2-chlorocyclopropyl)benzene (14).** Prepared according to General Procedure C1 using 1-bromo-4-vinylbenzene (33 µL, 0.25 mmol, 1 equiv), $CHCl_3$ (80 µL, 1.0 mmol, 4 equiv), Vitamin B_{12} (17 mg, 0.0125 mmol, 5 mol%), activated Zn powder (82 mg, 1.25 mmol, 5 equiv) and NH_4Cl (13 mg, 0.25 mmol, 1 equiv) in 2 mL of dry DMA. The crude reaction mixture was analyzed by ^{1}H NMR to determine the *cis*:*trans* ratio (83:17). The crude material was purified by flash column chromatography (0 → 25% EtOAc in Hex) to afford the title compound as a colorless oil in 71% yield (41 mg) with a dr ratio of 93:7. Spectral data are compared to the *trans*-isomer reported in the literature.[13]

^{1}H NMR: (400 MHz, $CDCl_3$) δ 7.48-7.42 (m, 2H), 7.16-7.10 (m, 2H), 3.38 (td, *J* = 7.4, 4.3 Hz, 1H), 2.30 (dt, *J* = 9.6, 7.5 Hz, 1H), 1.54-1.40 (m, 1H), 1.21 (ddd, *J* = 7.6, 6.8, 4.3 Hz, 1H). **$^{13}C\{^1H\}$ NMR:** (101 MHz, $CDCl_3$) δ 135.3, 131.2, 131.1, 120.8, 34.3, 22.5, 14.5. **Rf:** 0.63 (4:1 Hex:EtOAc).

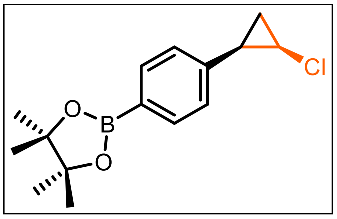

***cis*-2-(4-(2-Chlorocyclopropyl)phenyl)-4,4,5,5-tetramethyl-1,3,2-dioxaborolane (15).** Prepared according to General Procedure C1 using 4,4,5,5-tetramethyl-2-(4-vinylphenyl)-1,3,2-dioxaborolane (58 mg, 0.25 mmol, 1 equiv), $CHCl_3$ (80 µL, 1.0 mmol, 4 equiv), Vitamin B_{12} (17 mg, 0.0125 mmol,

5 mol%), activated Zn powder (82 mg, 1.25 mmol, 5 equiv) and NH_4Cl (13 mg, 0.25 mmol, 1 equiv) in 2 mL of dry DMA. The crude reaction mixture was analyzed by ^{1}H NMR to determine the *cis*:*trans* ratio (83:17). The crude material was purified by flash column chromatography (0 → 25% EtOAc in Hex) to afford the title compound as a white solid in 65% yield (45 mg) with a dr ratio of >95:5.

^{1}H NMR: (400 MHz, $CDCl_3$) δ 7.83-7.73 (d, *J* = 8.0, 2H), 7.30-7.21 (d, *J* = 8.11, 2H), 3.39 (td, *J* = 7.4, 4.3 Hz, 1H), 2.37 (dt, *J* = 9.5, 7.6 Hz, 1H), 1.48 (dt, *J* = 9.5, 7.0 Hz, 1H), 1.34-1.30 (m, 13H). **^{13}C{^{1}H} NMR:** (101 MHz, $CDCl_3$) δ 139.5, 134.6, 128.7, 83.8, 34.7, 25.0, 25.0, 23.2, 14.4. **Rf:** 0.58 (4:1 Hex:EtOAc). **HRMS (ESI) *m/z*:** $[M+H]^+$ calcd for $C_{15}H_{20}BClO_2$ 279.1323; found 279.1320. **IR (neat, cm^{-1}):** 2981, 2931, 1610, 1444, 1397, 1380, 1372, 1352, 1320, 1274, 1214, 1019, 960, 906, 856, 732, 679, 664, 651.

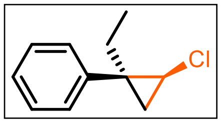

***cis*-(2-Chloro-1-ethylcyclopropyl)benzene (16).** Prepared according to General Procedure C1 using but-1-en-2-ylbenzene[5] (36 µL, 0.25 mmol, 1 equiv), $CHCl_3$ (80 µL, 1.0 mmol, 4 equiv), Vitamin B_{12} (17 mg, 0.0125 mmol, 5 mol%), activated Zn powder (82 mg, 1.25 mmol, 5 equiv) and NH_4Cl (13 mg, 0.25 mmol, 1 equiv) in 2 mL of dry DMA. The crude reaction mixture was analyzed by ^{1}H NMR to determine the *cis*:*trans* ratio (76:24). The crude material was purified by flash column chromatography (0 → 25% EtOAc in Hex) to afford the title compound as a colorless oil in 16% (7 mg) of the *trans*-isomer and 58% (26 mg) of the *cis*-isomer, combining to give a total of 74% yield (33 mg) with a dr ratio of 78:22.

^{1}H NMR, *cis*-isomer: (800 MHz, $CDCl_3$) δ 7.35 (t, *J* = 7.5 Hz, 2H), 7.30 (d, *J* = 7.7 Hz, 2H), 7.27 (d, *J* = 6.6 Hz, 1H), 3.16 (dd, *J* = 7.3, 3.9 Hz, 1H), 1.92 (dq, *J* = 14.5, 7.3 Hz, 1H), 1.24 (q, *J* = 7.0 Hz, 2H), 1.22 – 1.19 (m, 1H), 0.81 (t, *J* = 7.4 Hz, 3H). **^{13}C{^{1}H} NMR, *cis*-isomer:** (201 MHz,

$CDCl_3$) δ 139.5, 130.7, 128.1, 128.1, 127.0, 38.3, 34.3, 33.5, 20.8, 10.7. **Rf, *cis*-isomer:** 0.89 (4:1 Hex:EtOAc). **^{1}H NMR, *trans*-isomer:** (800 MHz, $CDCl_3$) δ 7.30 (t, *J* = 7.6 Hz, 2H), 7.26 (m, 2H), 7.23-7.20 (m, 1H), 3.32 (dd, *J* = 7.7, 4.3 Hz, 1H), 1.94-1.87 (m, 1H), 1.83 (dq, *J* = 14.6, 7.4 Hz, 1H), 1.43 (m, 1H), 0.94 (dd, *J* = 6.1, 4.3 Hz, 1H), 0.89 (t, *J* = 7.4 Hz, 3H). **^{13}C{^{1}H} NMR, *trans*-isomer:** (201 MHz, $CDCl_3$) δ 143.1, 129.1, 128.7, 128.7, 128.5, 127.6, 126.8, 40.7, 32.9, 28.9, 21.6, 11.2. **Rf, *trans*-isomer:** 0.86 (4:1 Hex:EtOAc). **HRMS (ESI) *m/z*:** $[M-H]^+$ calcd for $C_{11}H_{13}Cl$ 179.0628; found 179.0612. **IR (neat, cm^{-1}):** 3060, 3027, 2964, 2923, 2873, 1602, 1497, 1445, 1431, 1377, 1301, 1074, 1039, 1028, 919, 892, 784, 759, 697.

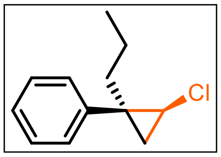

***cis*-(2-chloro-1-propylcyclopropyl)benzene (17).** Prepared according to General Procedure C1 using pent-1-en-2-ylbenzene[5] (40 µL, 0.25 mmol, 1 equiv), $CHCl_3$ (80 µL, 1.0 mmol, 4 equiv) Vitamin B_{12}, (17 mg, 0.0125 mmol, 5 mol%), activated Zn powder (82 mg, 1.25 mmol, 5 equiv), NH_4Cl (13 mg, 0.25 mmol, 1 equiv), in 2 mL dry DMA. The crude reaction mixture was analyzed by ^{1}H NMR to determine the *cis*:*trans* ratio (75:25). The crude material was purified by flash column chromatography (0 → 25% EtOAc in Hex) to afford the title compound as a colorless oil in 12% (6 mg) of the *trans*-isomer and 62% (30 mg) of the *cis*-isomer, combining to give a total 74% yield (36 mg) with a dr ratio of 84:16.

^{1}H NMR, *cis*-isomer: (800 MHz, $CDCl_3$) δ 7.34 (t, *J* = 7.5 Hz, 2H), 7.32-7.29 (m, 2H), 7.27 (d, *J* = 9.2 Hz, 1H), 3.15 (dd, *J* = 7.3, 4.0 Hz, 1H), 1.88 (ddd, *J* = 12.2, 10.5, 4.6 Hz, 1H), 1.28 (dt, *J* = 19.3, 7.1 Hz, 2H), 1.25-1.18 (m, 2H), 1.15 (ddd, *J* = 13.5, 10.7, 5.3 Hz, 1H), 0.81 (t, *J* = 7.3 Hz, 3H). **^{13}C{^{1}H} NMR, *cis*-isomer:** (201 MHz, $CDCl_3$) δ 139.8, 130.6, 128.1, 126.9, 42.8, 38.3, 33.1, 21.2, 19.9, 14.1. **Rf:** 0.87 (4:1 Hex:EtOAc). **^{1}H NMR, *trans*-isomer:** (800 MHz, $CDCl_3$) δ 7.29 (t, *J* = 7.5 Hz, 2H), 7.27-7.24 (m, 2H), 7.21 (t, *J* = 7.2 Hz, 1H), 3.28 (dd, *J* = 7.7, 4.3 Hz, 1H), 1.89-1.84 (m, 1H), 1.76-1.70 (m, 1H), 1.47-1.44 (m, 1H), 1.40-1.33 (m, 1H), 1.28-1.20 (m, 1H),

0.95 (dd, J = 6.2, 4.4 Hz, 1H), 0.86 (t, J = 7.4 Hz, 3H). **^{13}C{^{1}H} NMR, *trans*-isomer:** (201 MHz, $CDCl_3$) δ 143.5, 128.9, 128.5, 126.8, 40.5, 37.9, 31.9, 21.7, 20.1, 14.3. **Rf, *trans*-isomer:** 0.92 (4:1 Hex:EtOAc). **HRMS (ESI) *m/z*:** $[M-H]^+$ calcd for $C_{12}H_{15}Cl$ 193.0784; found 193.0761. **IR (neat, cm^{-1}):** 3061, 3028, 2957, 2930, 2873, 1497, 1465, 1457, 1445, 1431, 1301, 1041, 1028, 766, 759, 698, 680.

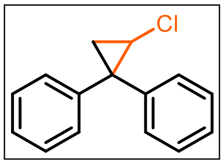

(2-Chlorocyclopropane-1,1-diyl)dibenzene (18). Prepared according to General Procedure C1 using 1,1-diphenylethylene (44 µL, 0.25 mmol, 1 equiv), $CHCl_3$ (80 µL, 1.0 mmol, 4 equiv), Vitamin B_{12} (17 mg, 0.0125 mmol, 5 mol%), activated Zn powder (82 mg, 1.25 mmol, 5 equiv) and NH_4Cl (13 mg, 0.25 mmol, 1 equiv) in 2 mL of dry DMA. The crude material was purified by PTLC (10% EtOAc in Hex) to afford the title compound as a colorless oil in 73% yield (42.6 mg). Spectral data are in accordance with those reported in the literature.[11]

^{1}H NMR: (800 MHz, $CDCl_3$) δ 7.45 -7.43 (m, 2H), 7.39-7.36 (m, 2H), 7.31-7.28 (m, 3H), 7.27-7.25 (m, 2H), 7.22-7.20 (m, 1H), 3.76 (dd, J = 7.6, 4.6 Hz, 1H), 1.83-1.77 (m, 2H). **^{13}C{^{1}H} NMR:** (200 MHz, $CDCl_3$) δ 144.3, 139.7, 130.6, 128.6, 128.3, 127.8, 127.6, 127.1, 126.7, 39.5, 37.0, 23.7. **Rf:** 0.78 (4:1 Hex:EtOAc).

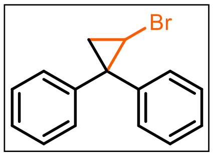

(2-Bromocyclopropane-1,1-diyl)dibenzene (19). Prepared according to General Procedure C3 using 1,1-diphenylethylene (176 µL, 1.0 mmol, 1 equiv), $CHBr_3$ (350 µL, 4.0 mmol, 4 equiv), Vitamin B_{12}, (68 mg, 0.05 mmol, 5 mol%), activated Zn powder (327 mg, 5.0 mmol, 5 equiv) and NH_4Br (98 mg, 1.0 mmol, 1 equiv) in 8 mL of dry DMA. The crude material was purified by flash column chromatography (0 → 25% EtOAc in Hex) to

afford the title compound as a colorless oil in 70% yield (190 mg). Spectral data are in accordance with those reported in the literature.[11]

^{1}H NMR: (400 MHz, $CDCl_3$) δ 7.46-7.34 (m, 4H), 7.32-7.26 (m, 3H), 7.25 (m, 2H), 7.22-7.17 (m, 1H), 3.71 (dd, J = 7.8, 4.8 Hz, 1H), 1.90-1.84 (m, 2H). **^{13}C{^{1}H} NMR:** (101 MHz, $CDCl_3$) δ 144.3, 140.8, 130.6, 128.7, 128.3, 127.9, 127.3, 126.8, 36.4, 28.5, 24.0. **Rf:** 0.83 (4:1 Hex:EtOAc).

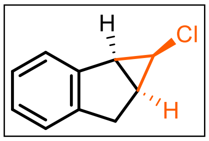

***cis*-1-Chloro-1,1a,6,6a-tetrahydrocyclopropa[*a*]indene (20).** Prepared according to General Procedure C1 using 1*H*-indene (29 µL, 0.25 mmol, 1 equiv), $CHCl_3$ (80 µL, 1.0 mmol, 4 equiv), Vitamin B_{12} (17 mg, 0.0125 mmol, 5 mol%), activated Zn powder (82 mg, 1.25 mmol, 5 equiv) and NH_4Cl (13 mg, 0.25 mmol, 1 equiv) in 2 mL of dry DMA. The crude reaction mixture was analyzed by ^{1}H NMR to determine the *cis*:*trans* ratio (81:19). The crude material was purified by flash column chromatography (0 → 25% EtOAc in Hex) to afford the title compound as a colorless oil in 46% yield (19 mg) with a dr ratio of >95:5.

^{1}H NMR: (400 MHz, $CDCl_3$) δ 7.42-7.36 (m, 1H), 7.35 (s, 1H), 7.30-7.22 (m, 2H), 3.74 (t, J = 7.2 Hz, 1H), 3.34 (dd, J = 17.3, 7.2 Hz, 1H), 3.18-3.07 (d, J = 16.2 Hz, 1H), 2.94 (td, J = 6.9, 1.9 Hz, 1H), 2.30-2.22 (q, J = 7.1 Hz, 1H). **^{13}C{^{1}H} NMR:** (101 MHz, $CDCl_3$) δ 144.5, 140.1, 128.0, 126.8, 126.3, 125.1, 124.0, 40.9, 32.2, 31.0, 23.1. **Rf:** 0.75 (4:1 Hex:EtOAc). **HRMS (ESI) *m/z*:** $[M-H]^+$ calcd for $C_{10}H_9Cl$ 163.0315; found 163.0319. **IR (neat, cm^{-1}):** 2924, 2848, 1559, 1398, 1373, 1357, 1142, 1093, 858, 759, 756, 744, 741, 652.

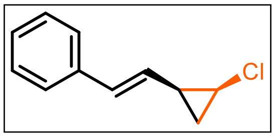

***cis*-(*E*)-2-(2-Chlorocyclopropyl)vinyl)benzene (21).** Prepared according to General Procedure C1 using (*E*)-buta-1,3-dien-1-ylbenzene[3] (34 µL, 0.25 mmol, 1 equiv), $CHCl_3$ (80 µL, 1.0 mmol, 4 equiv), Vitamin B_{12} (17 mg, 0.0125 mmol, 5 mol%), activated Zn powder (82 mg, 1.25 mmol, 5 equiv) and NH_4Cl (13 mg, 0.25 mmol, 1 equiv) in 2

mL of dry DMA. The crude reaction mixture was analyzed by ^{1}H NMR to determine the *cis*:*trans* ratio (75:25). The crude material was purified by PTLC (10% EtOAc in Hex) to afford the title compound as a colorless oil in 75% yield (36 mg) with a dr ratio of 81:19.

^{1}H NMR: (800 MHz, $CDCl_3$) δ ***cis:*** 7.38-7.36 (m, 2H), 7.32-7.29(m, 2H), 7.26-7.21 (m, 1H), 6.60 (d, *J* = 15.9 Hz, 1H), 6.06 (dd, *J* = 15.8, 8.8 Hz, 1H), 3.35 (td, *J* = 7.2, 4.4 Hz, 1H), 1.90 (tt, *J* = 9.1, 7.0 Hz, 1H), 1.42-1.39 (m, 1H), 0.96 (td, *J* = 6.6, 4.4 Hz, 1H), ***trans:*** 7.40 (m, 2H), 6.70 (d, *J* = 15.5 Hz, 1H), 5.71 (dd, *J* = 15.8, 8.3 Hz, 1H), 3.02 (ddd, *J* = 7.3, 4.2, 3.1 Hz, 1H), **^{13}C{^{1}H} NMR:** (200 MHz, $CDCl_3$) δ 137.2, 132.0, 128.8, 127.9, 127.2, 126.7, 126.0, 75.6, 71.1, 51.3, 43.8, 34.8, 21.3, 16.9. **Rf:** 0.76 (4:1 Hex:EtOAc). **HRMS (ESI) *m/z*:** $[M+H]^+$ calcd for $C_{11}H_{11}Cl$ 179.0627; found 179.0622. **IR (neat, cm^{-1}):** 3082, 3025, 2954, 2920, 1493, 1449, 1433, 1283, 1074, 1034, 987, 959, 748, 693, 654.

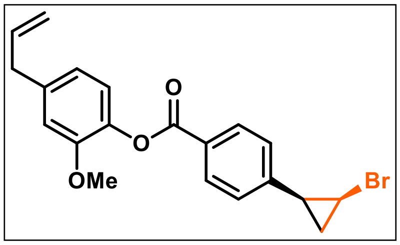

***cis*-4-allyl-2-methoxyphenyl-4-(2-bromocyclopropyl)benzoate (22).** Prepared according to General Procedure C1 using 4-allyl-2-methoxyphenyl 4-vinylbenzoate (73.5 mg, 0.25 mmol, 1 equiv), $CHBr_3$ (87 µL, 1.0 mmol, 4 equiv), Vitamin B_{12} (17 mg, 0.0125 mmol, 5 mol%), activated Zn powder (82 mg, 1.25 mmol, 5 equiv) and NH_4Cl (13 mg, 0.25 mmol, 1 equiv) in 2 mL of dry DMA. The crude reaction mixture was analyzed by ^{1}H NMR to determine the *cis:trans* ratio (74:26). The crude material was purified by flash column chromatography (0 → 25% EtOAc in Hex) to afford the title compound as a colorless oil in 60% yield (58 mg) with a dr ratio of 94:6.

^{1}H NMR: (800 MHz, $CDCl_3$) δ 8.18 (dd, *J* = 8.3, 1.4 Hz, 2H), 7.44 – 7.31 (m, 2H), 7.06 (dd, *J* = 8.0, 1.2 Hz, 1H), 6.84 (s, 1H), 6.83 – 6.75 (m, 1H), 6.07 – 5.87 (m, 1H), 5.26 – 5.01 (m, 2H), 3.81 (d, *J* = 1.2 Hz,

3H), 3.41 (dd, J = 6.7, 1.5 Hz, 2H), 3.37 (tdd, J = 7.7, 4.7, 1.3 Hz, 1H), 2.40 (q, J = 8.1 Hz, 1H), 1.76 – 1.61 (m, 1H), 1.55 (d, J = 1.2 Hz, 1H), 1.42 (tdd, J = 7.2, 5.4, 1.2 Hz, 1H). ^{13}C NMR (201 MHz, $CDCl_3$) δ 164.8, 151.2, 143.2, 139.0, 138.2, 137.2, 129.9, 129.3, 128.0, 122.7, 120.7, 116.2, 112.9, 55.9, 40.2, 23.8, 22.3, 14.8. **Rf:** 0.32 (4:1 Hex:EtOAc). **HRMS (ESI) m/z:** [M+H]+ calcd for $C_{20}H_{20}O_3Br$ 387.0596; found 387.0594. **IR (neat, cm−1):** 1734, 1610, 1506, 1464, 1418, 1265, 1199, 1179, 1150, 1123, 1035, 1017, 904, 725, 649.

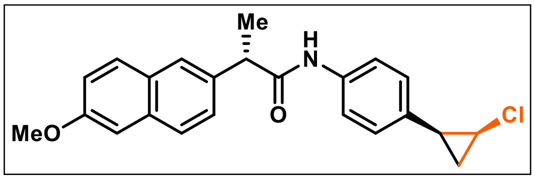

***cis*-(*S*)-*N*-(2-chlorocyclopropyl)phenyl)-2-(6-methoxynaphthalen-2-yl)propanamide (23).** Prepared according to General Procedure C1 using (S)-2-(6-methoxynaphthalen-2-yl)-N-(4-vinylphenyl)propanamide (82.9 mg, 0.25 mmol, 1 equiv), $CHCl_3$ (80 μL, 1.0 mmol, 4 equiv), Vitamin B_{12} (17 mg, 0.0125 mmol, 5 mol%), activated Zn powder (82 mg, 1.25 mmol, 5 equiv) and NH_4Cl (13 mg, 0.25 mmol, 1 equiv) in 2 mL of dry DMA. The crude reaction mixture was analyzed by ^{1}H NMR to determine the *cis:trans* ratio (80:20). The crude material was purified by flash column chromatography (0 → 20% EtOAc in Hex) to afford the title compound as a white solid in 53% yield (50 mg) with a dr ratio > 95:5.

^{1}H NMR: (800 MHz, CDCl3) δ 7.76 (d, J = 8.4 Hz, 1H), 7.75 – 7.69 (m, 2H), 7.42 (dd, J = 8.5, 1.9 Hz, 1H), 7.36 (d, J = 8.4 Hz, 2H), 7.18 (dd, J = 8.9, 2.5 Hz, 1H), 7.18 – 7.08 (m, 3H), 3.93 (s, 3H), 3.85 (q, J = 7.2 Hz, 1H), 3.32 (td, J = 7.4, 4.2 Hz, 1H), 2.28 (dt, J = 9.7, 7.5 Hz, 1H), 1.68 (d, J = 7.1 Hz, 3H), 1.43 (dt, J = 9.7, 7.0 Hz, 1H), 1.17 (td, J = 7.1, 4.2 Hz, 1H). **^{13}C NMR:** (201 MHz, $CDCl_3$) δ 172.3, 157.9, 136.5, 135.9, 133.9, 131.9, 129.7, 129.3, 129.0, 127.9, 126.4, 126.2, 119.4, 119.2, 105.7, 55.4, 48.1, 34.4, 22.3, 18.5, 14.1. **Rf:** 0.46 (7:3 Hex:EtOAc). **HRMS (ESI) m/z:** [M+H]+ calcd for $C_{23}H_{23}ClNO_2$ 380.1412; found 380.1412. **IR (neat, cm−1):** 3298, 2906, 1653, 1603, 1507, 1390, 1262, 1216, 1162, 1028, 906, 826, 732, 651.

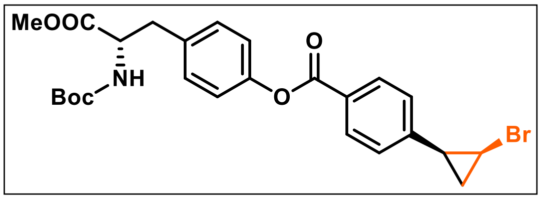

***cis*-4-((S)-2-((*tert*-butoxycarbonyl)amino)-3-methoxy-3-oxopropyl)phenyl 4-(2-bromocyclopropyl)benzoate (24).** Prepared according to General Procedure C1 using (S)-4-(2-((tert-butoxycarbonyl)amino)-3-methoxy-3-oxopropyl)phenyl 4-vinylbenzoate (106.4 mg, 0.25 mmol, 1 equiv), $CHBr_3$ (87 µL, 1.0 mmol, 4 equiv), Vitamin B_{12} (17 mg, 0.0125 mmol, 5 mol%), activated Zn powder (82 mg, 1.25 mmol, 5 equiv) and NH4Cl (13 mg, 0.25 mmol, 1 equiv) in 2 mL of dry DMA. The crude reaction mixture was analyzed by ^{1}H NMR to determine the *cis:trans* ratio (91:8). The crude material was purified by flash column chromatography (0 → 20% EtOAc in Hex) to afford the title compound as a white solid in 48% yield (62 mg) with a dr ratio of >95:5.

^{1}H NMR: (800 MHz, $CDCl_3$) δ 8.15 (d, *J* = 8.0 Hz, 2H), 7.37 (d, *J* = 8.1 Hz, 2H), 7.18 (d, *J* = 8.2 Hz, 2H), 7.15 (d, *J* = 8.3 Hz, 2H), 5.01 (d, *J* = 8.2 Hz, 1H), 4.60 (d, *J* = 7.1 Hz, 1H), 3.73 (s, 3H), 3.38 (td, *J* = 7.6, 4.6 Hz, 1H), 3.22 – 2.98 (m, 2H), 2.41 (dt, *J* = 9.2, 7.6 Hz, 1H), 1.68 (dt, *J* = 9.3, 7.3 Hz, 1H), 1.43 (s, 9H). **^{13}C NMR:** (201 MHz, $CDCl_3$) δ 172.3, 165.0, 155.1, 150.0, 143.5, 133.7, 130.4, 129.8, 129.4, 127.9, 121.8, 54.4, 52.3, 37.8, 28.3, 23.7, 22.3, 14.83. **Rf:** 0.37 (7:3 Hex:EtOAc). **HRMS (ESI) m/z:** [M-Boc+H]+ calcd for $C_{20}H_{21}$BrNO4 418.0654; found 418.0652. **IR (neat, cm−1):** 2979, 1733, 1610, 1506, 1365, 1263, 1170, 1068, 908, 707, 647.

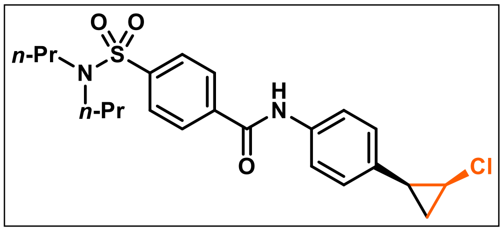

***cis*-N-(4-(2-chlorocyclopropyl)phenyl)-4-(*N*,*N*-dipropylsulfamoyl)benzamide (25).** Prepared according to General Procedure C1 using 4-(*N*,*N*-dipropylsulfamoyl)-*N*-(4-vinylphenyl)benzamide (96.6 mg, 0.25 mmol, 1 equiv), $CHCl_3$ (80 µL, 1.0 mmol, 4 equiv), Vitamin B_{12} (17 mg, 0.0125 mmol, 5 mol%), activated Zn powder (82 mg, 1.25 mmol, 5 equiv) and NH_4Cl (13 mg, 0.25 mmol, 1

equiv) in 2 mL of dry DMA. The crude reaction mixture was analyzed by ^{1}H NMR to determine the *cis:trans* ratio (90:10). The crude material was purified by flash column chromatography (0 → 20% EtOAc in Hex) to afford the title compound as a white solid in 30% yield (33 mg) with a dr ratio > 95:5.

^{1}H NMR: (800 MHz, $CDCl_3$) δ 7.93 (d, J = 8.1 Hz, 2H), 7.82 (d, J = 8.5 Hz, 2H), 7.65 (d, J = 8.0 Hz, 2H), 7.27 (d, J = 8.4 Hz, 2H), 3.39 (td, J = 7.4, 4.2 Hz, 1H), 3.11 – 3.06 (m, 4H), 2.39 – 2.33 (m, 1H), 1.59 (s, 1H), 1.55 (h, J = 7.4 Hz, 4H), 1.49 (dt, J = 9.6, 7.1 Hz, 2H), 1.25 (td, J = 7.2, 4.3 Hz, 1H), 0.87 (t, J = 7.4 Hz, 6H). **^{13}C{^{1}H} NMR:** (201 MHz, $CDCl_3$) δ 164.5, 142.9, 138.7, 136.3, 132.9, 130.0, 127.9, 127.4, 119.9, 50.0, 34.5, 22.4, 22.0, 14.3, 11.2. **Rf:** 0.39 (7:3 Hex:EtOAc). **HRMS (ESI) m/z:** [M+H]+ calcd for $C_{22}H_{28}ClN_2O_3S$ 435.1504; found 435.1499. **IR (neat, cm−1):** 2966, 1653, 1600, 1503, 1405, 1323, 1176, 1085, 92, 833, 732, 603.

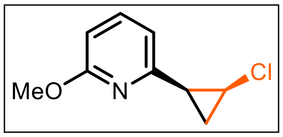

***cis*-(2-Chlorocyclopropyl)-6-methoxypyridine (26).** Prepared according to General Procedure C1 using 2-methoxy-6-vinylpyridine[4] (33 µL, 0.25 mmol, 1 equiv), $CHCl_3$ (80 µL, 1.0 mmol, 4 equiv), Vitamin B_{12} (17 mg, 0.0125 mmol, 5 mol%), activated Zn powder (82 mg, 1.25 mmol, 5 equiv) and NH_4Cl (13 mg, 0.25 mmol, 1 equiv) in 2 mL of dry DMA. The crude reaction mixture was analyzed by ^{1}H NMR to determine the *cis*:*trans* ratio (82:18). The crude material was purified by flash column chromatography (0 → 25% EtOAc in Hex) to afford the title compound as a pale-yellow oil in 41% yield (19 mg) with a dr ratio of >95:5. Spectral data are compared to the *trans*-isomer reported in the literature.[13]

^{1}H NMR: (400 MHz, $CDCl_3$) δ 7.50 (dd, *J* = 8.2, 7.3 Hz, 1H), 6.85 (m, 1H), 6.59 (m, 1H), 3.42 (td, *J* = 7.5, 4.9 Hz, 1H), 2.43 (dt, *J* = 9.3, 7.4 Hz, 1H), 1.79 (ddd, *J* = 7.3, 6.3, 4.9 Hz, 1H), 1.44

(ddd, J = 9.3, 7.4, 6.3 Hz, 1H). **$^{13}C\{^1H\}$ NMR:** (101 MHz, $CDCl_3$) δ 163.3, 153.7, 138.4, 116.7, 108.4, 53.4, 35.4, 24.3, 14.0. **Rf:** 0.76 (4:1 Hex:EtOAc).

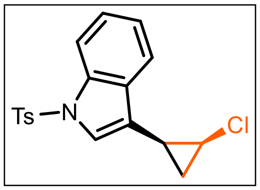

***cis*-3-(-2-Chlorocyclopropyl)-1-tosyl-1*H*-indole (27).** Prepared according to General Procedure C1 using 1-tosyl-3-vinyl-1*H*-indole[1] (74.3 mg, 0.25 mmol, 1 equiv), $CHCl_3$ (80 μL, 1.00 mmol, 4 equiv), Vitamin B_{12} (17 mg, 0.0125 mmol, 5 mol%), activated Zn powder (82 mg, 1.25 mmol, 5 equiv) and NH_4Cl (13 mg, 0.25 mmol, 1 equiv) in 2 mL of dry DMA. The crude reaction mixture was analyzed by ^1H NMR to determine the *cis*:*trans* ratio (>95:5). The crude material was purified by flash column chromatography (0 → 25% EtOAc in Hex) to afford the title compound as a white solid in 56% yield (48 mg) with a dr ratio of >95:5. Spectral data are compared to the *trans*-isomer reported in the literature.[13]

^1H NMR: (400 MHz, $CDCl_3$) δ 7.96 (dt, J = 8.2, 0.9 Hz, 1H), 7.76 -7.67 (m, 2H), 7.58 (d, J = 7.6 Hz, 1H), 7.38 (d, J = 1.2 Hz, 1H), 7.32 (td, J = 8.3, 1.4 Hz, 1H), 7.29-7.22 (m, 1H), 7.21 – 7.13 (m, 2H), 3.45 (td, J = 7.2, 4.3 Hz, 1H), 2.31 (s, 3H), 2.26 (dtd, J = 9.5, 7.3, 1.2 Hz, 1H), 1.60-1.52 (m, 1H), 1.17 (ddd, J = 7.3, 6.5, 4.3 Hz, 1H). **$^{13}C\{^1H\}$ NMR:** (101 MHz, $CDCl_3$) δ 144.9, 135.3, 135.2, 131.8, 129.9, 126.9, 125.0, 124.8, 123.4, 119.6, 119.3, 114.0, 33.7, 21.7, 14.6, 13.7. **Rf:** 0.46 (4:1 Hex:EtOAc).

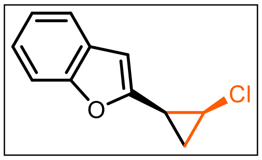

***cis*-2-(2-Chlorocyclopropyl)benzofuran (28).** Prepared according to General Procedure C1 using 3-vinylbenzofuran[2] (39 μL, 0.25 mmol, 1 equiv), $CHCl_3$ (80 μL, 1.0 mmol, 4 equiv), Vitamin B_{12} (17 mg, 0.0125 mmol, 5 mol%), activated Zn powder (82 mg, 1.25 mmol, 5 equiv) and NH_4Cl (13 mg, 0.25 mmol, 1 equiv) in 2 mL of dry DMA. The crude reaction mixture was analyzed by ^1H NMR to determine

the *cis*:*trans* ratio (85:15). The crude material was purified by PTLC (15% EtOAc in Hex) to afford the title compound as a colorless oil in 61% yield corrected by quantitative NMR with 1,2 dichloromethane as internal standard (33 mg isolated, purity 89%) with a dr ratio of >95:5. Spectral data are compared to the *trans*-isomer reported in the literature.[13]

^{1}H NMR: (800 MHz, $CDCl_3$) δ 7.46 (d, 8.2Hz, 1H), 7.35 (d, 8.2Hz, 1H), 7.18 (m, 2H), 6.46 (s, 1H), 3.40cc (td, J = 7.9, 4.0 Hz, 1H), 2.45 (m, 1H), 1.61 (q, 6.8Hz, 1H), 1.45 (m, 1H). **^{13}C{^{1}H} NMR:** (200 MHz, $CDCl_3$) δ 155.7, 154.3, 128.7, 123.6, 122.8, 120.3, 111.8, 102.2, 33.3, 20.6, 17.5 **Rf:** 0.66 (4:1 Hex:EtOAc).

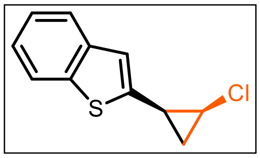

***cis*-2-(2-Chlorocyclopropyl)benzo[*b*]thiophene (29).** Prepared according to General Procedure C1 using 3-vinylbenzo[*b*]thiophene[2] (40.1 mg, 0.25 mmol, 1 equiv), $CHCl_3$ (80 µL, 1.00 mmol, 4 equiv), Vitamin B_{12} (17 mg, 0.0125 mmol, 5 mol%), activated Zn powder (82 mg, 1.25 mmol, 5 equiv) and NH_4Cl (13 mg, 0.25 mmol, 1 equiv) in 2 mL of dry DMA. The crude reaction mixture was analyzed by ^{1}H NMR to determine the *cis*:*trans* ratio (71:29). The crude material was purified by flash column chromatography (0 → 25% EtOAc in Hex) to afford the title compound as a colorless oil in 61% yield (32 mg) with a dr ratio of >95:5. Spectral data are compared to the *trans*-isomer reported in the literature.[13]

^{1}H NMR: (800 MHz, $CDCl_3$) δ 7.78 (m, 1H), 7.73-7.67 (m, 1H), 7.35-7.27 (m, 2H), 7.11 (m, 1H), 3.43 (td, J = 7.2, 4.5 Hz, 1H), 2.51 (m, 1H), 1.65 (dt, J = 9.5, 7.0 Hz, 1H), 1.36 (m, 1H). **^{13}C{^{1}H} NMR:** (200 MHz, $CDCl_3$) δ 141.0, 140.0, 139.8, 124.3, 124.0, 123.3, 123.0, 122.2, 34.8, 18.8, 16.7. **Rf:** 0.65 (4:1 Hex:EtOAc).

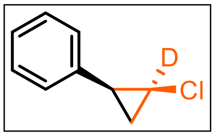

***cis*-(2-Chlorocyclopropyl-2-*d*)benzene (30).** Prepared according to General Procedure C1 using styrene (29 µL, 0.25 mmol, 1 equiv), $CDCl_3$ (80 µL, 1.00 mmol, 4 equiv), Vitamin B_{12} (17 mg, 0.0125 mmol, 5 mol%), activated Zn powder (82 mg, 1.25 mmol, 5 equiv) and NH_4Cl (13 mg, 0.25 mmol, 1 equiv) in 2 mL of dry DMA. The crude reaction mixture was analyzed by ^1H NMR to determine the *cis*:*trans* ratio (91:9). The crude material was purified by flash column chromatography (0 → 25% EtOAc in Hex) to afford the title compound as a colorless oil in 65% yield (25 mg) with a dr ratio of >95:5. Spectral data are in accordance with those reported in the literature.[15]

^{1}H NMR: (400 MHz, $CDCl_3$) δ 7.37-7.30 (m, 2H), 7.27 (m, 3H), 2.36 (t, J = 8.6 Hz, 1H), 1.47 (m, 1H), 1.25 (t, J = 7.1 Hz, 1H). **^{13}C{^{1}H} NMR:** (101 MHz, $CDCl_3$) δ 136.2, 129.4, 128.1, 126.9, 22.9, 14.2. **Rf:** 0.76 (4:1 Hex:EtOAc).

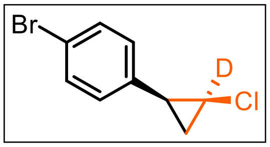

***cis*-1-Bromo-4-(2-chlorocyclopropyl-2-*d*)benzene (31).** Prepared according to General Procedure C1 using 1-bromo-4-vinylbenzene (33 µL, 0.25 mmol, 1 equiv), $CDCl_3$ (80 µL, 1.0 mmol, 4 equiv), Vitamin B_{12} (17 mg, 0.0125 mmol, 5 mol%), activated Zn powder (82 mg, 1.25 mmol, 5 equiv) and NH_4Cl (13 mg, 0.25 mmol, 1 equiv) in 2 mL of dry DMA. The crude reaction mixture was analyzed by ^1H NMR to determine the *cis*:*trans* ratio (89:11). The crude material was purified by flash column chromatography (0 → 25% EtOAc in Hex) to afford the title compound as a colorless oil in 86% yield (50 mg) with a dr ratio of >95:5.

^{1}H NMR: (400 MHz, $CDCl_3$) δ 7.49-7.41 (d, J = 8.4 Hz, 2H), 7.16-7.09 (d, J = 8.4 Hz, 2H), 2.29 (t, J = 8.4 Hz, 1H), 1.48 (dd, J = 9.6, 6.8 Hz, 1H), 1.20 (t, J = 7.2 Hz, 1H). **^{13}C{^{1}H} NMR:** (101 MHz, $CDCl_3$) δ 135.3, 131.2, 131.1, 120.8, 22.4, 14.4. **Rf:** 0.81 (4:1 Hex:EtOAc). **HRMS (ESI)**

***m/z*:** $[M+H]^+$ calcd for C_9H_7DBrCl 231.9639; found 231.9638. **IR (neat, cm^{-1}):** 2927, 2852, 1490, 1436, 1226, 1185, 1106, 1073, 1035, 1009, 837, 811, 713, 695, 641, 601.

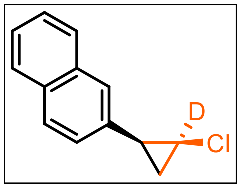

2-(2-chlorocyclopropyl-2-*d*)naphthalene (32). Prepared according to General Procedure C1 using vinylnaphthalene (39 mg, 0.25 mmol, 1 equiv), $CDCl_3$ (80 µL, 1.0 mmol, 4 equiv), Vitamin B_{12} (17 mg, 0.0125 mmol, 5 mol%), activated Zn powder (82 mg, 1.25 mmol, 5 equiv) and NH_4Cl (13 mg, 0.25 mmol, 1 equiv) in 2 mL of dry DMA. The crude reaction mixture was analyzed by ^1H NMR to determine the *cis*:*trans* ratio (81:19). The crude material was purified by flash column chromatography (0 → 10% EtOAc in Hex) to afford the title compound as a colorless oil in 63% yield (32 mg). Spectral data are in accordance with those reported in the literature.[15]

^{1}H NMR: (400 MHz, $CDCl_3$) δ ^1H NMR (400 MHz, $CDCl_3$) δ 7.82 (td, J = 6.5, 3.0 Hz, 3H), 7.70 (d, J = 1.8 Hz, 1H), 7.57 – 7.37 (m, 3H), 2.52 (dd, J = 9.5, 7.6 Hz, 1H), 1.59 – 1.53 (m, 1H), 1.41 (t, J = 7.2 Hz, 1H). **$^{13}C\{^1H\}$ NMR:** (101 MHz, $CDCl_3$) δ 133.8, 133.2, 132.5, 127.8, 127.8, 127.7, 127.5, 126.0, 125.6, 34.6, 34.3, 33.9, 22.9, 14.1. **Rf:** 0.76 (4:1 Hex:EtOAc).

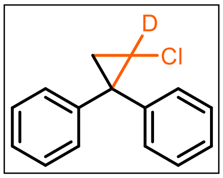

(2-Chlorocyclopropane-1,1-diyl-2-*d*)dibenzene (33). Prepared according to General Procedure C1 using 1,1-diphenylethylene (44 µL, 0.25 mmol, 1 equiv), $CDCl_3$ (80 µL, 1.0 mmol, 4 equiv), Vitamin B_{12} (17 mg, 0.0125 mmol, 5 mol%), activated Zn powder (82 mg, 1.25 mmol, 5 equiv) and NH_4Cl (13 mg, 0.25 mmol, 1 equiv) in 2 mL of dry DMA. The crude material was purified by flash column chromatography (0 → 25% EtOAc in Hex) to afford the title compound as a colorless oil in 70% yield (40 mg). Spectral data are in accordance with those reported in the literature.[15]

^{1}H NMR: (400 MHz, $CDCl_3$) δ 7.47-7.41 (m, 2H), 7.41-7.33 (m, 2H), 7.33-7.23 (m, 5H), 7.23-7.16 (m, 1H), 1.85-1.73 (m, 2H). **$^{13}C\{^1H\}$ NMR:** (101 MHz, $CDCl_3$) δ 144.4, 139.8, 130.7, 129.0, 128.7, 128.4, 127.9, 127.8, 127.2, 126.8, 37.0, 23.7. **Rf:** 0.77 (4:1 Hex:EtOAc).

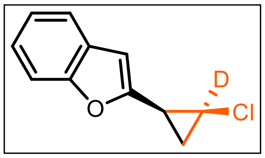

2-(2-chlorocyclopropyl-2-*d*)benzofuran (34). Prepared according to General Procedure C1 using 3-vinylbenzofuran[2] (39 µL, 0.25 mmol, 1 equiv), $CDCl_3$ (80 µL, 1.0 mmol, 4 equiv), Vitamin B_{12} (17 mg, 0.0125 mmol, 5 mol%), activated Zn powder (82 mg, 1.25 mmol, 5 equiv) and NH_4Cl (13 mg, 0.25 mmol, 1 equiv) in 2 mL of dry DMA. The crude reaction mixture was analyzed by ^{1}H NMR to determine the *cis*:*trans* ratio (75:25). The crude material was purified by flash column chromatography (0 → 25% EtOAc in Hex) to afford the title compound as a colorless to pale yellow oil in 79% yield (38 mg) with a dr ratio of >95:5. Spectral data are in accordance with those reported in the literature.[15]

^{1}H NMR: (400 MHz, $CDCl_3$) δ 7.48-7.41 (m, 1H), 7.40-7.34 (m, 1H), 7.21-7.10 (m, 2H), 6.47 (m, 1H), 2.40-2.31 (m, 1H), 1.52 (dd, J = 9.7, 6.7 Hz, 1H), 1.36 (t, J = 7.0 Hz, 1H). **$^{13}C\{^1H\}$ NMR:** (101 MHz, $CDCl_3$) δ 155.0, 154.5, 128.7, 123.8, 122.8, 120.7, 111.0, 104.7, 16.9, 14.7. **Rf:** 0.76 (4:1 Hex:EtOAc).

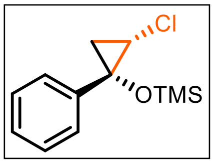

(2-Chloro-1-phenylcyclopropoxy)trimethylsilane (35). Prepared according to General Procedure C1 using trimethyl((1-phenylvinyl)oxy)silane[7] (51 µL, 0.25 mmol, 1 equiv), $CHCl_3$ (80 µL, 1.00 mmol, 4 equiv), Vitamin B_{12} (17 mg, 0.0125 mmol, 5 mol%), activated Zn powder (82 mg, 1.25 mmol, 5 equiv) and NH_4Cl (13 mg, 0.25 mmol, 1 equiv) in 2 mL of dry DMA. The crude reaction mixture was analyzed by ^{1}H NMR to determine the dr ratio (>95:5). The crude material was purified by flash column chromatography (0 → 25% EtOAc in Hex) to afford the title compound as a yellow oil in 57% yield (34 mg) as a single isomer.

^{1}H NMR: (400 MHz, $CDCl_3$) δ 7.39-7.21 (m, 5H), 3.06 (dd, J = 8.5, 5.2 Hz, 1H), 1.79 (dd, J = 8.5, 7.6 Hz, 1H), 1.36 (dd, J = 7.7, 5.2 Hz, 1H), 0.11 (s, 9H). **^{13}C{^{1}H} NMR:** (101 MHz, $CDCl_3$) δ 142.2, 128.5, 127.5, 125.8, 59.8, 39.2, 23.9, 1.1. **Rf:** 0.79 (4:1 Hex:EtOAc). **HRMS (ESI) *m/z*:** $[M-H]^+$ calcd for $C_{12}H_{17}ClOSi$ 239.0659; found 239.0658. **IR (neat, cm^{-1}):** 3063, 3034, 2959, 2900, 1448, 1046, 1104, 1070, 1046, 909, 835, 756, 697, 682.

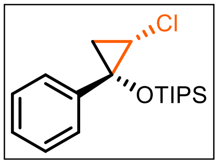

(2-Chloro-1-phenylcyclopropoxy)triisopropylsilane (36). Prepared according to General Procedure C1 using triisopropyl((1-phenylvinyl)oxy)silane[7] (77 µL, 0.25 mmol, 1 equiv), $CHCl_3$ (80 µL, 1.0 mmol, 4 equiv), Vitamin B_{12} (17 mg, 0.0125 mmol, 5 mol%), activated Zn powder (82 mg, 1.25 mmol, 5 equiv) and NH_4Cl (13 mg, 0.25 mmol, 1 equiv) in 2 mL of dry DMA. The crude reaction mixture was analyzed by ^{1}H NMR to determine the dr ratio (>95:5). The crude material was purified by flash column chromatography (0 → 25% EtOAc in Hex) to afford the title compound as a colorless oil in 67% yield (54 mg) as a single isomer.

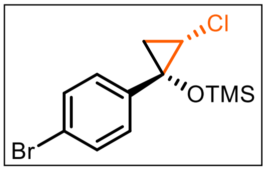

(1-(4-Bromophenyl)-2-chlorocyclopropoxy)trimethylsilane (37). Prepared according to General Procedure C1 using ((1-(4-bromophenyl)vinyl)oxy)trimethylsilane[7] (55 µL, 0.25 mmol, 1 equiv), $CHCl_3$ (80 µL, 1.00 mmol, 4 equiv), Vitamin B_{12} (17 mg, 0.0125 mmol, 5 mol%), activated Zn powder (82 mg, 1.25 mmol, 5 equiv) and NH_4Cl (13 mg, 0.25 mmol, 1 equiv) in 2 mL of dry DMA. The crude reaction mixture was analyzed by ^{1}H NMR to determine the dr ratio (>95:5). The crude material was purified by flash column chromatography (0 → 25% EtOAc in Hex) to afford the title compound as a colorless oil in 44% yield (34 mg) as a single isomer.

^{1}H NMR: (400 MHz, $CDCl_3$) δ 7.47-7.41 (m, 2H), 7.19-7.09 (m, 2H), 3.00 (dd, J = 8.5, 5.3 Hz, 1H), 1.77 (dd, J = 8.5, 7.8 Hz, 1H), 1.37 (dd, J = 7.8, 5.3 Hz, 1H), 0.11 (s, 9H). **^{13}C{^{1}H} NMR:** (101 MHz, $CDCl_3$) δ 141.5, 131.7, 127.4, 121.4, 59.4, 39.2, 23.8, 1.2. **Rf:** 0.81 (4:1 Hex:EtOAc). **HRMS (ESI) *m/z*:** $[M+H]^+$ calcd for $C_{12}H_{16}BrClOSi$ 318.9920; found 318.9922. **IR (neat, cm^{-1}):** 2960, 2898, 1488, 11423, 1394, 1305, 1285, 1252, 1102, 1076, 1044, 1008, 915, 843, 810, 754, 717, 688.

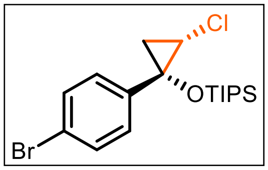

(1-(4-Bromophenyl)-2-chlorocyclopropoxy)triisopropylsilane (38). Prepared according to General Procedure C1 using ((1-(4-bromophenyl)vinyl)oxy)triisopropylsilane[9] (76 μL, 0.25 mmol, 1 equiv), $CHCl_3$ (80 μL, 1.0 mmol, 4 equiv), Vitamin B_{12} (17 mg, 0.0125 mmol, 5 mol%), activated Zn powder (82 mg, 1.25 mmol, 5 equiv) and NH_4Cl (13 mg, 0.25 mmol, 1 equiv) in 2 mL of dry DMA. The crude reaction mixture was analyzed by ^{1}H NMR to determine the dr ratio (>95:5). The crude material was purified by PTLC (15% EtOAc in Hex) to afford the title compound as a yellowish oil in 68% yield corrected by quantitative NMR with 1,2-dichloromethane as internal standard (74.5 mg, purity 91%) as a single isomer.

^{1}H NMR: (800 MHz, $CDCl_3$) δ 7.45-7.43z (d, 8.5Hz, 2H), 7.23-7.22 (d, 8.5Hz, 2H), 2.80 (dd, J = 8.6, 5.4 Hz, 1H), 1.92 (t, J = 8.2 Hz, 1H), 1.36 (dd, J = 7.9, 5.4 Hz, 1H), 1.01 (m, 21H). **^{13}C{^{1}H} NMR:** (200 MHz, $CDCl_3$) δ 141.4, 131.4, 128.0, 121.4, 59.1, 40.4, 22.2, 18.0, 18.0, 12.8. **Rf:** 0.87 (4:1 Hex:EtOAc). **HRMS (ESI) *m/z*:** $[M+H]^+$ calcd for $C_{18}H_{28}BrClOSi$ 403.0859; found 403.0852. **IR (neat, cm^{-1}):** 2994, 2892, 2866, 1688, 1586, 1464, 1257, 1103, 1070, 1009, 998, 912, 881, 812, 718, 674.

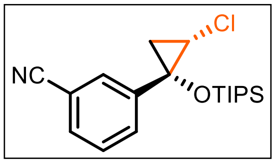

3-(2-chloro-1-((triisopropylsilyl)oxy)cyclopropyl)benzonitrile (39). Prepared according to General Procedure C1 using 3-(1-((triisopropylsilyl)oxy)vinyl)benzonitrile[9] (55.8 µL, 0.25 mmol, 1 equiv), $CHCl_3$ (80 µL, 1.0 mmol, 4 equiv), Vitamin B_{12} (17 mg, 0.0125 mmol, 5 mol%), activated Zn powder (82 mg, 1.25 mmol, 5 equiv) and NH_4Cl (13 mg, 0.25 mmol, 1 equiv) in 2 mL of dry DMA. The crude reaction mixture was analyzed by ^1H NMR to determine the dr ratio (>95:5). The crude material was purified by flash column chromatography (0 → 25% EtOAc in Hex) to afford the title compound as a yellow oil in 44% yield (38 mg) as a single isomer.

^{1}H NMR: (400 MHz, $CDCl_3$) δ 7.64-7.54 (m, 3H), 7.45 (td, *J* = 7.7, 0.8 Hz, 1H), 2.82 (dd, *J* = 8.7, 5.5 Hz, 1H), 1.99 (t, *J* = 8.4 Hz, 1H), 1.46 (dd, *J* = 8.2, 5.5 Hz, 1H), 1.01 (m, 21H). **^{13}C{^{1}H} NMR:** (101 MHz, $CDCl_3$) δ 144.2, 131.3, 130.6, 129.6, 129.4, 118.7, 112.7, 59.0, 40.7, 22.4, 18.3, 18.1, 18.0, 13.0, 12.9. **Rf:** 0.7 (4:1 Hex:EtOAc). **HRMS (ESI) *m/z*:** $[M+H]^+$ calcd for $C_{19}H_{28}ClNOSi$ 350.1707; found 350.1710. **IR (neat, cm^{-1}):** 2945, 2892, 2867, 2230, 1696, 1464, 1427, 1291, 1264, 1054, 988, 882, 795, 743, 699, 678.

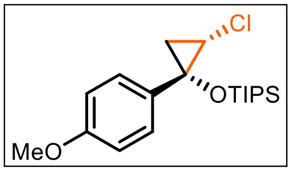

(2-chloro-1-(4-methoxyphenyl)cyclopropoxy)triisopropylsilane (40). Prepared according to General Procedure C1 using triisopropyl((1-(4-methoxyphenyl)vinyl)oxy)silane[9] (74 µL, 0.25 mmol, 1 equiv), $CHCl_3$ (80 µL, 1.0 mmol, 4 equiv), Vitamin B_{12} (17 mg, 0.0125 mmol, 5 mol%), activated Zn powder (82 mg, 1.25 mmol, 5 equiv) and NH_4Cl (13 mg, 0.25 mmol, 1 equiv) in 2 mL of dry DMA. The crude reaction mixture was analyzed by ^1H NMR to determine the dr ratio (>95:5). The crude material was purified by flash column chromatography (0 → 25% EtOAc in Hex) to afford the title compound as a colorless to pale yellow oil in 55% yield (49 mg) as a single isomer.

^{1}H NMR: (400 MHz, $CDCl_3$) δ 7.32-727 (m, 2H), 6.86-6.81 (m, 2H), 3.80 (s, 3H), 2.84 (dd, *J* = 8.5, 5.2 Hz, 1H), 1.87 (t, *J* = 8.2 Hz, 1H), 1.29 (dd, *J* = 7.6, 5.2 Hz, 1H), 0.99 (m, 21H). **$^{13}C\{^1H\}$ NMR:** (101 MHz, $CDCl_3$) δ 159.2, 134.4, 128.3, 113.7, 59.4, 55.4, 40.3, 22.3, 18.1, 12.9. **Rf:** 0.84 (4:1 Hex:EtOAc). **HRMS (ESI) *m/z*:** $[M+H]^+$ calcd for $C_{19}H_{31}ClO2Si$ 355.186; found 355.187. **IR (neat, cm^{-1}):** 3509, 2942, 2893, 2865, 1674, 1599, 1575, 1511, 1464, 1256, 1241, 1208, 1168, 1112, 882, 832, 803, 790, 675.

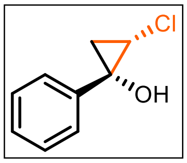

2-Chloro-1-phenylcyclopropan-1-ol (41). An oven-dried 1 dram vial equipped with a magnetic stir bar was charged with Vitamin B_{12} (68 mg, 0.05 mmol, 5 mol%), activated Zn powder (327 mg, 1.25 mmol, 5 equiv), and NH_4Cl (54 mg, 1.0 mmol, 1 equiv). Dry DMA (8 mL, 0.125 M) was added, and the reaction mixture was degassed by sparging with Ar for 10 minutes while stirring. $CHCl_3$ (320 µL, 4.0 mmol, 4 equiv) was added under Ar, followed by trimethyl((1-phenylvinyl)oxy)silane[7] (204 µL, 1.0 mmol, 1 equiv). The reaction mixture was then sonicated and then irradiated with a Kessil PR160L-525 nm LED in an EvoluChem photobox for 8 h. The reaction mixture was diluted with Et_2O (60 mL), filtered through a cotton plug to remove the Zn powder, and washed with saturated aqueous NH_4Cl (60 mL) and brine (3 x 60 mL). The combined aqueous phase was then extracted with Et_2O (60 mL), and the combined organic phase was dried with $MgSO_4$ and concentrated to yield compound **30**. Compound **30** was transferred into a 2-dram vial equipped with a stir bar and purged with Ar for 10 minutes. 2 mL (0.5 M) of dry MeOH (dried over 3Å molecular sieves) was added and purged for 5 minutes. The mixture was cooled to 0 ºC, and 3 drops of chlorotrimethylsilane were added, and the reaction mixture was stirred for 10 minutes. The reaction was concentrated and purified by flash column chromatography (0 → 20% EtOAc in Hex) to afford the title compound as a colorless oil in 53% yield (90 mg) as a single isomer.

^{1}H NMR: (400 MHz, $CDCl_3$) δ 7.39-7.33 (m, 2H), 7.32-7.27 (m, 3H), 3.32 (dd, *J* = 8.4, 5.4 Hz, 1H), 2.67 (bs, 1H), 1.77 (t, *J* = 8.1 Hz, 1H), 1.44 (dd, *J* = 7.9, 5.4 Hz, 1H). **^{13}C{^{1}H} NMR:** (101 MHz, $CDCl_3$) δ 141.3, 128.7, 127.5, 124.8, 57.4, 40.6, 25.5. **Rf:** 0.47 (4:1 Hex:EtOAc). **HRMS (ESI) *m/z*:** $[M+H]^+$ calcd for C_9H_9ClO 169.0420; found 169.0417. **IR (neat, cm^{-1}):** 3294, 3224, 3059, 3045, 3036, 19521605, 1501, 1443, 1422, 1316, 1297, 1264, 1238, 1043, 1033, 1016, 882, 853, 763, 755, 696, 681.

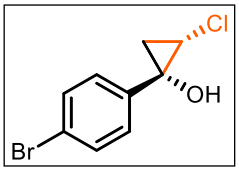

1-(4-Bromophenyl)-2-chlorocyclopropan-1-ol (42). An oven-dried 1 dram vial equipped with a magnetic stir bar was charged with Vitamin B_{12} (68 mg, 0.05 mmol, 5 mol%), activated Zn powder (327 mg, 1.25 mmol, 5 equiv), and NH_4Cl (54 mg, 1.0 mmol, 1 equiv). Dry DMA (8 mL, 0.125 M) was added, and the reaction mixture was degassed by sparging with Ar for 10 minutes while stirring. $CHCl_3$ (320 µL, 4.0 mmol, 4 equiv) was added under Ar, followed by ((1-(4-bromophenyl)vinyl)oxy)trimethylsilane[7] (220 µL, 1.0 mmol, 1 equiv). The reaction mixture was then sonicated and irradiated with a Kessil PR160L-525 nm LED in an EvoluChem photobox for 8 h. The reaction mixture was diluted with Et_2O (60 mL), filtered through a cotton plug to remove the Zn powder, and washed with saturated aqueous NH_4Cl (60 mL) and brine (3 x 60 mL). The combined aqueous phase was then extracted with Et_2O (60 mL), and the combined organic phase was dried with $MgSO_4$ and concentrated to yield compound **32**. Compound **32** was transferred into a 2-dram vial equipped with a stir bar and purged with Ar for 10 minutes. 2 mL (0.5 M) of dry MeOH (dried over 3Å molecular sieves) was added and purged for 5 minutes. The mixture was cooled to 0 ºC, and 3 drops of chlorotrimethylsilane were added and the reaction mixture was stirred for 10 minutes. The reaction mixture was concentrated and purified by flash column chromatography (0 → 20% EtOAc in Hex) to afford the title compound as a colorless oil in 47% yield (90 mg) as a single isomer.

^{1}**H NMR:** (400 MHz, $CDCl_3$) δ 7.50-7.43 (m, 2H), 7.20-7.12 (m, 2H), 3.28 (dd, *J* = 8.4, 5.4 Hz, 1H), 2.68 (s, 1H), 1.74 (t, *J* = 8.2 Hz, 1H), 1.44 (dd, *J* = 8.0, 5.4 Hz, 1H). ^{13}**C{^{1}H} NMR:** (101 MHz, $CDCl_3$) δ 140.4, 131.8, 126.5, 121.4, 57.0, 40.6, 25.7. **Rf:** 0.51 (4:1 Hex:EtOAc). **HRMS (ESI) *m/z*:** $[M+H]^+$ calcd for C_9H_8BrClO 246.9525; found 246.9526. **IR (neat, cm^{-1}):** 3252, 3167, 3046, 2854, 1905, 1488, 1421, 1397, 1308, 1285, 1261, 1101, 1075, 1041, 1019, 1004, 884, 870 809, 713, 641, 626.

F. Halocyclopropanation of Phenyl Vinyl Sulfone

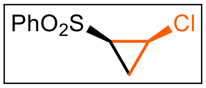

***cis*-((2-chlorocyclopropyl)sulfonyl)benzene.** Prepared according to General Procedure C1 using phenyl vinyl sulfone (42 mg, 0.25 mmol, 1 equiv), $CHCl_3$ (80 μL, 1.00 mmol, 4 equiv), Vitamin B_{12} (17 mg, 0.0125 mmol, 5 mol%), activated Zn powder (82 mg, 1.25 mmol, 5 equiv) and NH_4Cl (13 mg, 0.25 mmol, 1 equiv) in 2 mL of dry DMA. The crude reaction mixture was analyzed by ^{1}H NMR to determine the *cis*:*trans* ratio (73:27) and a yield of 28% (calculated using 1,3,5-trimethoxybenzene as an external standard). Product was not isolated. **GCMS:** calculated 216 m/z, measure 216 m/z.

G. Unsuccessful Substrates

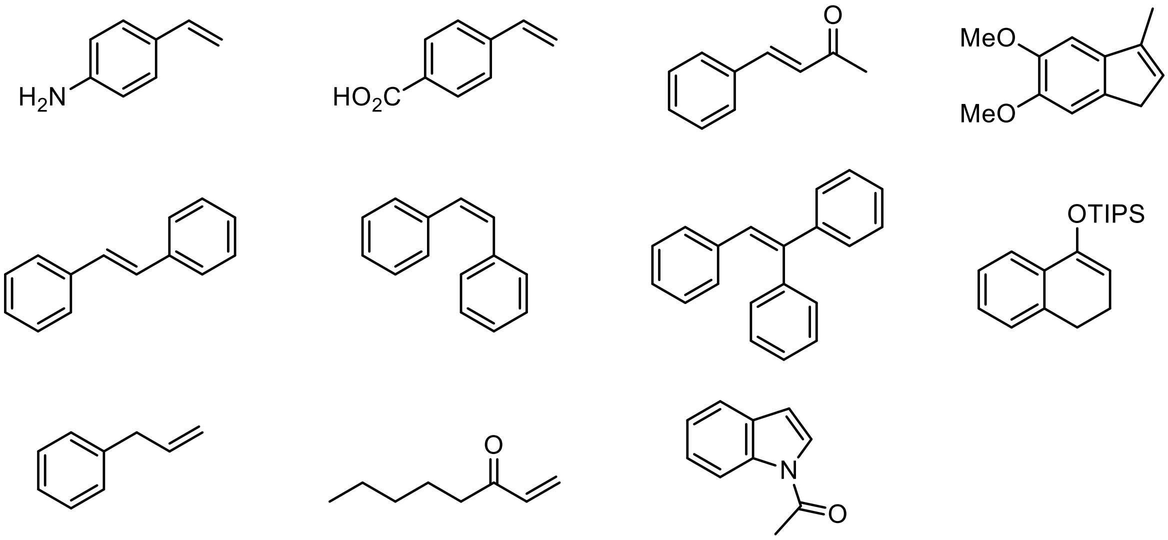

Figure S5. Unsuccessful alkene substrates for the Vitamin B_{12} halocyclopropanation reaction.

H. Synthetic Elaborations

1. 5 mmol Scale Halocyclopropanation

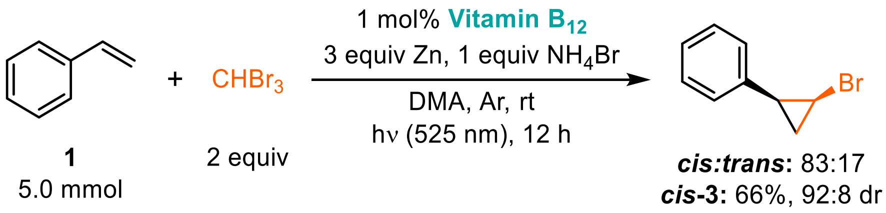

An oven-dried 250 mL round bottom flask equipped with a magnetic stir bar was charged with Vitamin B_{12} (67 mg, 0.1 mmol, 5 mol%) and activated Zn powder (980 mg, 15 mmol, 3 equiv). Dry DMA (100 mL) was added, and the reaction mixture was degassed by sparging with Ar for 10 minutes while stirring. After sparging, styrene (600 µL, 5 mmol, 1 equiv) was added under Ar, and the round bottom flask was equipped with a balloon of Ar gas. In parallel, a solution of NH_4Br (495 mg, 5 mmol, 1 equiv) and $CHBr_3$ (0.87 mL, 10.0 mmol, 4 equiv) was prepared in 40 mL of DMA in 50 mL round bottom flask equipped with magnetic stir bar and sparged with Ar for 10 minutes while stirring. The $CHBr_3$ solution in DMA was then loaded in a 50 mL syringe and mounted on the syringe pump. The syringe and the flask were connected by a long stainless steel needle. The syringe pump was set to inject at a rate of 4.2 mL per hour, and the reaction mixture was irradiated with two Kessil PR160L-525 nm LED lamps for 12 h. The reaction mixture was diluted with Et_2O (200 mL), filtered through a cotton plug to remove the Zn powder, and washed with saturated aqueous LiCl (3 x 100 mL) and brine (100 mL). The combined aqueous phase was extracted with Et_2O (2 x 150 mL), and the combined organic phases were dried with $MgSO_4$ and concentrated under vacuum. The crude reaction mixture was analyzed by ^1H NMR to determine the *cis*:*trans* ratio (83:17). The crude material was purified by flash column chromatography (100% Hex) to afford the title compound as a colorless oil in 66% yield (640 mg) with a dr ratio of 92:8. NMR and GC-MS data were in agreement with the previous synthesis.

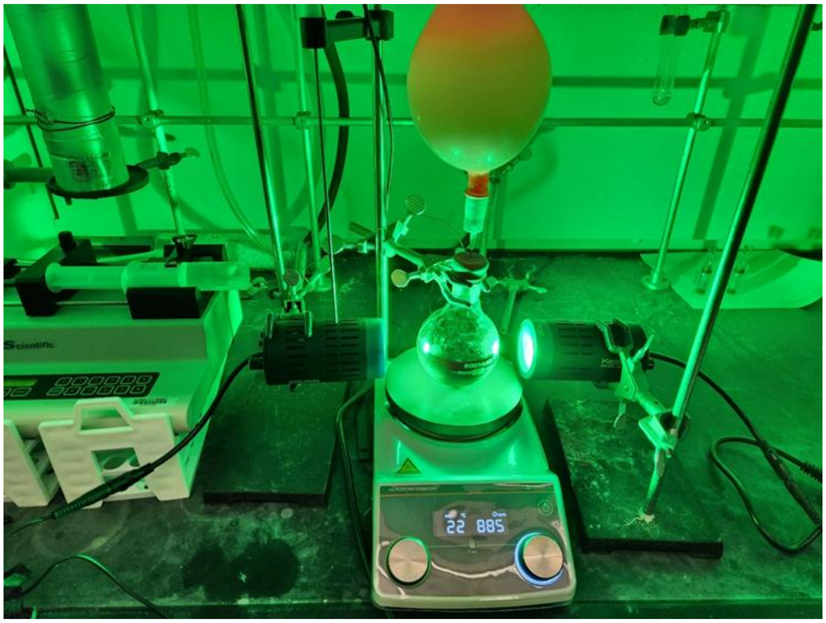

Figure S6. Picture of experimental setup for 5 mmol scale experiment.

2. Nucleophilic Amination

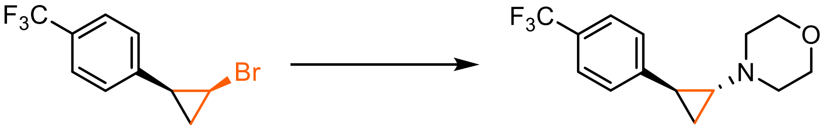

A flame-dried 2-dram vial equipped with a magnetic stir bar was charged with morpholine (39 mg, 0.44 mmol, 1.1 equiv) and THF (2 mL, 0.2 M). The mixture was degassed by sparging with Ar for 10 minutes while stirring and cooled to −78 °C. *n*-BuLi (313 μL, 0.48 mmol, 1.2 equiv) was added, and the reaction mixture was allowed to warm to −20 °C and stirred for 1 h. **12** (66 μL, 0.4 mmol, 1 equiv) was added in 2 mL THF slowly and stirred at −20 °C until full consumption of **12** was observed by TLC (around 8 h). The reaction mixture was carefully quenched with drops of saturated aqueous NH_4Cl, and the crude was concentrated and purified by flash column chromatography (0 → 40% EtOAc in Hex) to afford the title compound **43** as a yellow solid in 62% yield (67 mg) with a dr ratio of 92:8.

^{1}**H NMR:** (800 MHz, $CDCl_3$) δ 7.50 (d, *J* = 7.9 Hz, 2H), 7.13 (d, *J* = 7.9 Hz, 2H), 3.69 (m, 4H), 2.65 (m, 4H), 2.04 (m, 1H), 1.94 (m, 1H), 1.21 (m, 1H), 1.03-0.99 (m, 1H). ^{13}**C{^{1}H} NMR:** (201 MHz, $CDCl_3$) δ 146.4, 128.2, 128.0, 126.2, 125.4, 125.4, 125.3, 125.3, 125.1, 123.8, 67.1, 53.5, 49.7, 24.4, 17.1. ^{19}**F NMR:** (376 MHz, $CDCl_3$) δ −62.29 (s, 3F) **Rf:** 0.32 (1:1 Hex:EtOAc). **HRMS (ESI) m/z:** $[M+H]^+$ calcd for $C_{14}H_{16}F_3NO$ 272.1262; found 272.1259. **IR (neat, cm^{-1}):** 2995, 2932, 2855, 2810, 1619, 1450, 1322, 1275, 1207, 1167, 1111, 1019, 898, 859, 847, 826.

3. Cyclopropene Synthesis

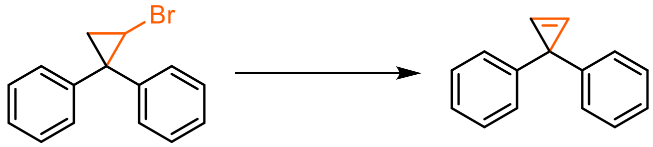

19 → **44** (67%)

An oven-dried 2-dram vial equipped with a magnetic stir bar was charged with potassium *tert*-butoxide (168 mg, 1.50 mmol, 3.00 equiv) in dry DMSO (1 mL), and the mixture was heated at 55 °C for 30 minutes while stirring until homogenous. The mixture was cooled to 25 °C and a solution of **19** (137 mg, 0.50 mmol, 1.00 equiv) dissolved in DMSO (0.5 mL) was then added dropwise over 15 minutes and the reaction mixture was stirred for 20 h. Additional potassium *tert*-butoxide (56 mg, 0.50 mmol, 1.00 equiv) was then added, and the reaction mixture was stirred for another 4 h. The reaction mixture was then quenched with brine (5 mL), and petroleum ether (10 mL) was added. The aqueous layer was washed with petroleum ether (3 x 10 mL), and the combined organic layers were washed with brine (2 x 30 mL), dried with $MgSO_4$, and concentrated. The crude was purified by flash column chromatography (0 → 10% EtOAc in Hex) to afford the title compound **44** as a colorless oil in 67% yield (64 mg). Spectral data are in accordance with those reported in the literature.[16]

^{1}H NMR: (800 MHz, $CDCl_3$) δ 7.49 (m, 2H), 7.31-7.27 (m, 4H), 7.21-7.17 (m, 6H). **^{13}C{^{1}H} NMR:** (201 MHz, $CDCl_3$) δ 147.2, 128.2, 128.2, 125.9, 113.4, 31.9. **Rf:** 0.82 (4:1 Hex:EtOAc)

4. Synthesis of Radical Clock

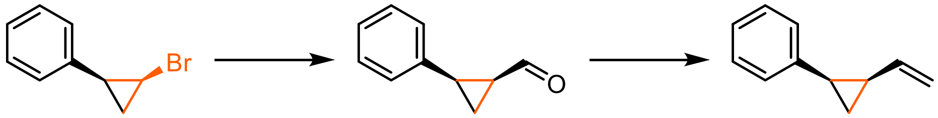

3 **45** **46** (42%, 86:14 dr)
Yield over 2 steps

An oven-dried 50 mL round bottom flask equipped with a magnetic stir bar was charged with *i*PrMgCl•LiCl (0.5 mL, 1.3 M in THF, 0.65 mmol, 1.25 equiv), and Dioxane (100 µL) then stirred for 20 min under Ar at room temperature. Then **3** (100 mg, 0.51 mmol, 1 equiv) was added neat and the reaction mixture was stirred for 8 h at room temperature. Then, DMF (180 µL, 2.5 mmol, 5 equiv) was slowly added at room temperature and the reaction mixture was stirred for an additional 2 h. The reaction was quenched with sat. aqueous NH_4Cl (10 mL), and the aqueous phase was extracted with Et_2O (3 x 20 mL). The organic phases were combined and dried with $MgSO_4$, and the solvent was removed under vacuum to afford compound **45**. The crude material was engaged in the next step without purification.

In a 25 mL round bottom flask was added CH_3PPh_3Br (250 mg, 0.7 mmol, 1.2 equiv), *t*-BuOK (80 mg, 0.7 mmol, 1.2 equiv) and THF (5 mL) and was stirred at 0 ºC for 1 h. Then, unpurified compound **45** from previous step was dissolved in THF (1 mL) and was slowly added to the reaction mixture. The reaction mixture was allowed to warm to room temperature and stirred for 8 h. The reaction was quenched with 1 M aqueous NaOH (10 mL), then neutralized with sat. NH_4Cl (10 mL), then extracted with EtOAc (3 x 20 mL). The organic phases were combined and dried with $MgSO_4$, and the solvent was removed under vacuum. The crude was purified by flash column chromatography (0 → 10% EtOAc in Hex) to afford the title compound **46** as a colorless

oil in 42% yield (36 mg, 86:14 dr) over two steps. Spectral data are in accordance with those reported in the literature.[17]

^{1}H NMR: (800 MHz, $CDCl_3$) **Major**: δ 7.29 – 7.25 (m, 2H), 7.21 (d, J = 7.6 Hz, 2H), 7.18 (t, J = 7.3 Hz, 1H), 5.13 – 5.09 (m, 2H), 4.87 – 4.83 (m, 1H), 2.36 (t, J = 8.6 Hz, 1H), 1.90 – 1.82 (m, 1H), 1.26 (m, 1H), 1.05 (q, J = 5.8 Hz, 1H). **Minor**: δ 5.58 – 5.51 (m, 1H), 4.94 (d, J = 12.6 Hz, 2H), 1.95 – 1.91 (m, 1H), 1.72 – 1.68 (m, 1H), 1.22 – 1.18 (m, 1H), 1.13 – 1.08 (m, 1H). **^{13}C{^{1}H} NMR:** (201 MHz, $CDCl_3$) δ 138.7, 138.1, 129.1, 128.3, 128.0, 125.9, 125.7, 114.1, 23.3, 22.9, 11.7.

5. Giese Reaction

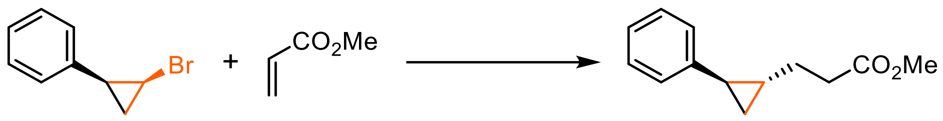

3
1.5 equiv

0.25 mmol

47 (49%, 94:6 dr)

An oven-dried 1 dram vial equipped with a magnetic stir bar was charged with Vitamin B_{12} (17 mg, 0.0125 mmol, 5 mol%), activated Zn powder (49 mg, 0.75 mmol, 3 equiv), and NH_4Br (25 mg, 0.25 mmol, 1 equiv). Dry DMF (2 mL, 0.125 M) was added, and the reaction mixture was degassed by sparging with Ar for 10 minutes while stirring. **3** (50 µL, 0.375 mmol, 1.5 equiv) was added under Ar, followed by methyl acrylate (23 µL, 0.25 mmol, 1 equiv). The reaction mixture was then sonicated and irradiated with a Kessil PR160L-525 nm LED in an EvoluChem photobox for 16 h. The reaction mixture was diluted with Et_2O (20 mL), then filtered through a cotton plug to remove the Zn powder and washed with saturated aqueous NH_4Cl (20 mL) and brine (3 x 20 mL). The combined aqueous phase was then extracted with Et_2O (20 mL), and the combined organic phase was dried with $MgSO_4$ and concentrated. The crude was purified by flash column chromatography (0 → 10% EtOAc in Hex) to afford the title compound **47** as a colorless oil in

49% yield (25 mg) with a dr ratio of 94:6. Spectral data are in accordance with those reported in the literature.[18]

^{1}H NMR: (400 MHz, $CDCl_3$) δ 7.31-7.20 (m, 2H), 7.18-7.08 (m, 1H), 7.08-7.00 (m, 2H), 3.66 (s, 3H), 2.46 (t, J = 7.5 Hz, 2H), 1.79-1.62 (m, 3H), 1.13-1.03 (m, 1H), 0.91 m, 1H), 0.80 (m, 1H). **^{13}C{^{1}H} NMR:** (101 MHz, $CDCl_3$) δ 174.1, 143.5, 128.4, 125.8, 125.5, 51.7, 34.1, 29.9, 23.4, 23.0, 16.0. **Rf:** 0.55 (4:1 Hex:EtOAc)

I. Mechanistic Studies

1. Radical Trap Experiment with TEMPO

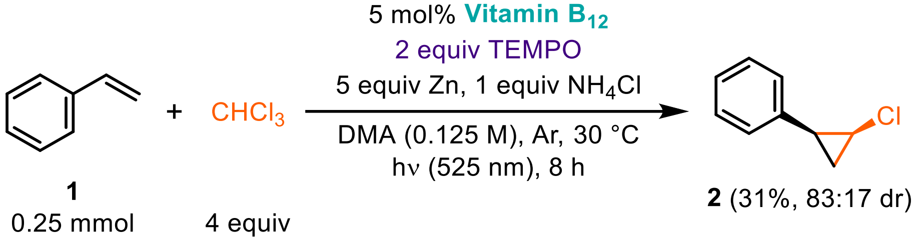

An oven-dried 1 dram vial equipped with a magnetic stir bar was charged with Vitamin B_{12} (17 mg, 0.0125 mmol, 5 mol%), activated Zn powder (82 mg, 1.25 mmol, 5 equiv), and NH_4Cl (13 mg, 0.25 mmol, 1 equiv). Dry DMA (2 mL, 0.125 M) was added, and the reaction mixture was degassed by sparging with Ar for 10 minutes while stirring. $CHCl_3$ (80 µL, 1.0 mmol, 4 equiv) was added under Ar, followed TEMPO (78 mg, 0.50 mmol, 2 equiv) and the mixture was degassed again for 5 minutes while stirring. Styrene (28 µL, 0.25 mmol, 1 equiv) was finally added and the reaction mixture was sonicated and then irradiated with a Kessil PR160L-525 nm LED in an EvoluChem photobox for 8 h. The reaction mixture was diluted with Et_2O (20 mL), filtered through a cotton plug to remove the Zn powder, and washed with saturated aqueous NH_4Cl (20 mL) and brine (3 x 20 mL). The combined aqueous phase was extracted with Et_2O (20 mL), and the combined organic phase was dried with $MgSO_4$. A small sample of the organic phase was taken for GC-MS analysis, and the remainder of the organic phase was concentrated. The yield of the product was determined by ^1H NMR analysis using 1,3,5-trimethoxybenzene as an external standard.

2. Radical Clock Experiment

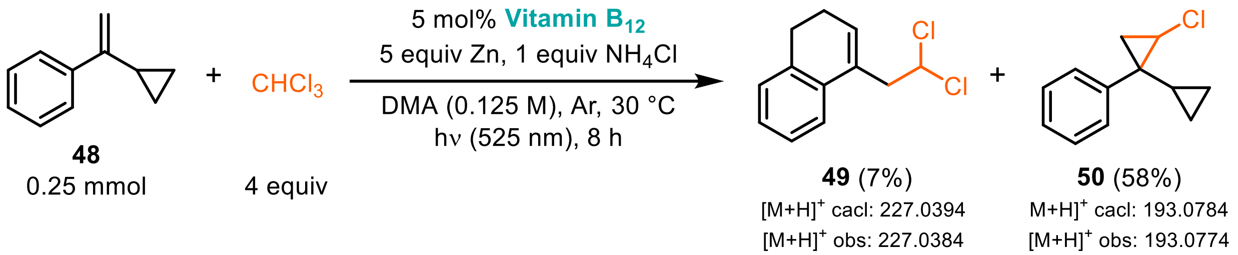

An oven-dried 1 dram vial equipped with a magnetic stir bar was charged with Vitamin B_{12} (17 mg, 0.0125 mmol, 5 mol%), activated Zn powder (82 mg, 1.25 mmol, 5 equiv), and NH_4Cl (13 mg, 0.25 mmol, 1 equiv). Dry DMA (2 mL, 0.125 M) was added, and the reaction mixture was degassed by sparging with Ar for 10 minutes while stirring. $CHCl_3$ (80 µL, 1.0 mmol, 4 equiv) was added under Ar, followed by (1-cyclopropylvinyl)benzene (**48**)[6] (37 µL, 0.25 mmol, 1 equiv). The reaction mixture was then sonicated and irradiated with a Kessil PR160L-525 nm LED in an EvoluChem photobox for 8 h. The reaction mixture was diluted with Et_2O (20 mL), filtered through a cotton plug to remove the Zn powder, and washed with saturated aqueous NH_4Cl (20 mL) and brine (3 x 20 mL). The combined aqueous phases were extracted with Et_2O (20 mL), and the combined organic phases were dried with $MgSO_4$ and concentrated. The yields of the products were determined by ^1H NMR analysis using 1,3,5-trimethoxybenzene as an external standard, and the identity of the products were confirmed by HRMS analysis.

49 – HRMS (ESI) *m/z*: $[M+H]^+$ calcd for $C_{12}H_{12}Cl_2$ 227.0394; found 227.0384.

50 – HRMS (ESI) *m/z*: $[M+H]^+$ calcd for $C_{12}H_{13}Cl$ 193.0784; found 193.0774

3. Monodehalogenation Control Reaction

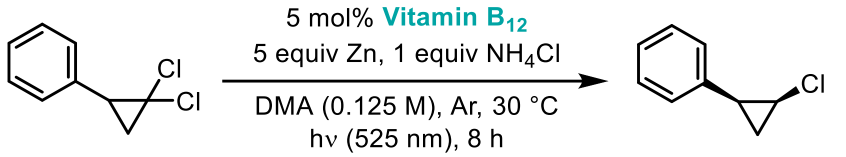

51
0.25 mmol

2 (12%, dr 58:42)

An oven-dried 1 dram vial equipped with a magnetic stir bar was charged with Vitamin B_{12} (17 mg, 0.0125 mmol, 5 mol%), activated Zn powder (82 mg, 1.25 mmol, 5 equiv), and NH_4Cl (13 mg, 0.25 mmol, 1 equiv). Dry DMA (2 mL, 0.125 M) was added, and the reaction mixture was degassed by sparging with Ar for 10 minutes while stirring. (2,2-dichlorocyclopropyl)benzene (**51**)[10] (37 μL, 0.25 mmol, 1 equiv) was added under Ar. The reaction mixture was then sonicated and then irradiated with a Kessil PR160L-525 nm LED in an EvoluChem photobox for 8 h. The reaction mixture was diluted with Et_2O (20 mL), filtered through a cotton plug to remove the Zn powder, and washed with saturated aqueous NH_4Cl (20 mL) and brine (3 x 20 mL). The combined aqueous phases were extracted with Et_2O (20 mL), and the combined organic phases were dried with $MgSO_4$ and concentrated. The yield and dr ratio of product **2** were determined by ^1H NMR analysis using 1,3,5-trimethoxybenzene as an external standard.

4. UV-Vis Studies

To determine the feasibility of the key S_N2 nucleophilic substitution between Vitamin B_{12} and $CHCl_3$, we performed a series of UV-Vis measurements. All absorption spectra were recorded using a Shimadzu UV-2600 UV-Vis spectrophotometer. First, 3 mL of a 0.11 mM solution of Vitamin B_{12} in DMF/DMA was added to a 3.5 mL quartz cuvette equipped with a magnetic stir bar, and the absorption spectrum was recorded. Next, activated Zn powder (25 mg), NH_4Cl (11 mg, [69 mM]), and a magnetic stir bar was added, the cuvette was sealed, and the mixture was degassed with Ar for 30 minutes while stirring. After the Zn powder was allowed to settle, an

absorption spectrum was recorded, revealing a new λ_{max} of ~390 nm, consistent with the formation of Co(I).[19,20] It should be noted that no formation of the Co(I) band was observed unless both Zn powder and NH_4Cl were added prior to degassing. To this same mixture, CHX_3 (10 μL) was then added, and an absorption spectrum was recorded. It was observed that addition of CHX_3 resulted in an immediate loss of the Co(I) absorption band, accompanied by the growth of a new charge-transfer band between 450-600 nm, characteristic of the formation of a Co(III)–R species.[21] These data provide support for the formation of the Co(I) oxidation state of Vitamin B_{12} and the subsequent S_N2-type nucleophilic substitution with CHX_3 to form the key Co(III)–CHX_2 intermediate.

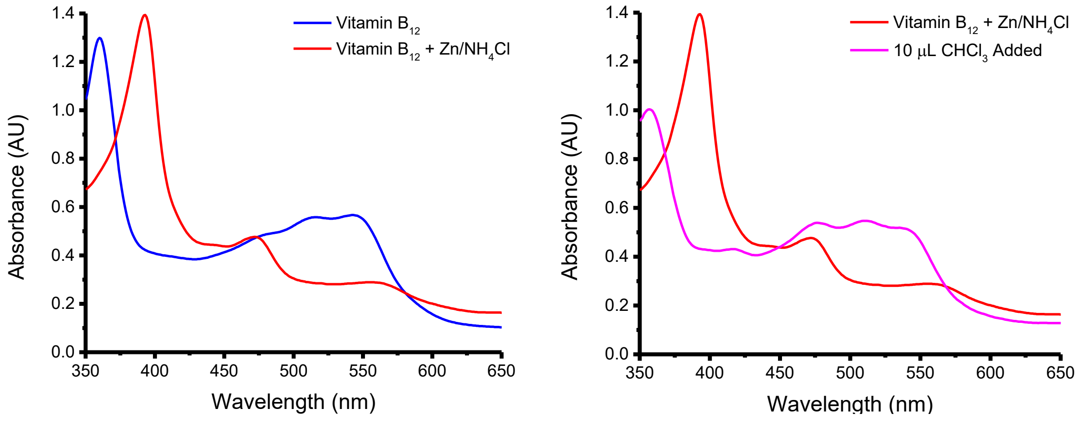

Figure S7. UV-vis studies investigating the feasibility of the S_N2-type nucleophilic substitution step for $CHCl_3$. [Vitamin B_{12}] = 0.11 mM, Zn = 25 mg, [NH_4Cl] = 69 mM, and [$CHCl_3$] = 0.04 mM in 3 mL DMF.

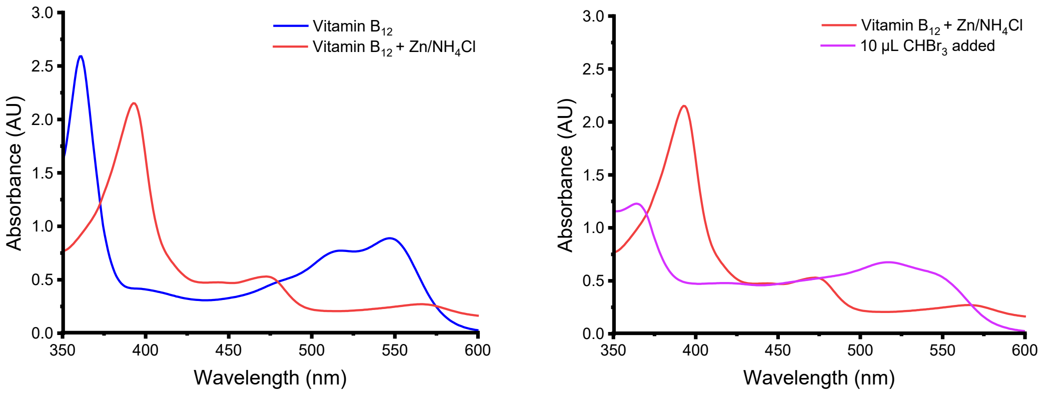

Figure S8. UV-vis studies investigating the feasibility of the S_N2-type nucleophilic substitution step for $CHBr_3$. [Vitamin B_{12}] = 0.11 mM, Zn = 25 mg, [NH_4Cl] = 69 mM, and [$CHBr_3$] = 0.04 mM in 3 mL DMA.

5. Initial Rate Studies

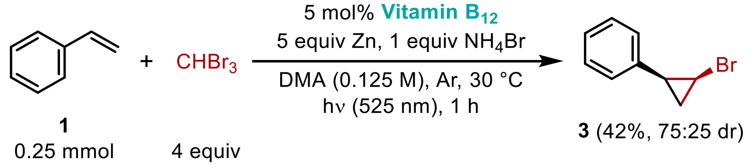

An oven-dried 1 dram vial with pressure relief cap equipped with a magnetic stir bar was charged with Vitamin B_{12} (17 mg, 0.0125 mmol, 5 mol%), activated Zn powder (82 mg, 1.25 mmol, 5 equiv), and NH_4Br (25 mg, 0.25 mmol, 1 equiv). Dry DMA (2 mL, 0.125 M, dried over 4 Å molecular sieves) was added, and the reaction mixture was degassed by sparging with argon for 10 minutes while stirring. $CHBr_3$ (87 µL, 1.0 mmol, 4 equiv) was added under argon, followed by the styrene (29 µL, 0.25 mmol, 1 equiv). The reaction mixture was then sonicated and irradiated with a Kessil PR160L 525 nm LED at 75% intensity in an EvoluChem Photobox for 1 h. The reaction mixture was filtered through a cotton plug, transferred into a separatory funnel with 20 mL of anhydrous Et_2O, and washed with saturated aqueous NH_4Cl (20 mL) and brine (3 x 20 mL). The combined aqueous phases were extracted with anhydrous Et_2O (20 mL), and the combined organic phases were dried over $MgSO_4$ and concentrated. Reaction was analyzed by ^1H NMR using 1,3,5-trimethoxybenzene as an external standard.

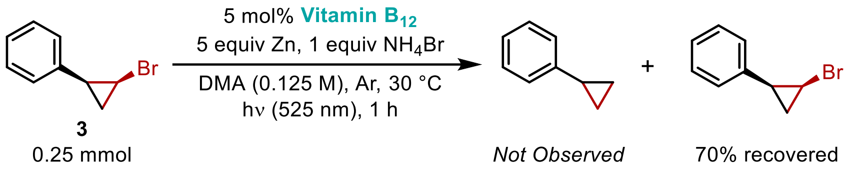

An oven-dried 1 dram vial with pressure relief cap equipped with a magnetic stir bar was charged with Vitamin B_{12} (17 mg, 0.0125 mmol, 5 mol%), activated Zn powder (82 mg, 1.25 mmol, 5 equiv), and NH_4Br (25 mg, 0.25 mmol, 1 equiv). Dry DMA (2 mL, 0.125 M, dried over 4 Å molecular sieves) was added, and the reaction mixture was degassed by sparging with argon for 10 minutes while stirring. **3** (49.3, 0.25 mmol, 1 equiv) was added under argon. The reaction mixture was then sonicated and irradiated with a Kessil PR160L 525 nm LED at 75% intensity in an EvoluChem Photobox for 1 h. The reaction mixture was filtered through a cotton plug, transferred into a separatory funnel with 20 mL of anhydrous Et_2O, and washed with saturated aqueous NH_4Cl (20 mL) and brine (3 x 20 mL). The combined aqueous phases were extracted with anhydrous Et_2O (20 mL), and the combined organic phases were dried over $MgSO_4$ and concentrated. Reaction was analyzed by ^1H NMR using 1,3,5-trimethoxybenzene as an external standard.

J. References

(1) Jérôme Waser, B. G. H. N. and E. M. C. Hydrazines and Azides via the Metal-Catalyzed Hydrohydrazination and Hydroazidation of Olefins. *J Am Chem Soc* **2006**, *128* (35), 11693–11712. https://doi.org/10.1021/ja062355+.

(2) Wang, T.; Wu, C.; Cui, D. Highly Syndioselective Coordination (Co)Polymerization of Vinyl Heteroaromatic Monomers Using Rare-Earth-Metal Complexes. *Polym Chem* **2020**, *11* (48), 7650–7655. https://doi.org/10.1039/d0py01447h.

(3) Wei, X. J.; Yang, D. T.; Wang, L.; Song, T.; Wu, L. Z.; Liu, Q. A Novel Intermolecular Synthesis of γ-Lactones via Visible-Light Photoredox Catalysis. *Org Lett* **2013**, *15* (23), 6054–6057. https://doi.org/10.1021/ol402954t.

(4) Su, M.; Huang, X.; Lei, C.; Jin, J. Nickel-Catalyzed Reductive Cross-Coupling of Aryl Bromides with Vinyl Acetate in Dimethyl Isosorbide as a Sustainable Solvent. *Org Lett* **2022**, *24* (1), 354–358. https://doi.org/10.1021/acs.orglett.1c04018.

(5) Rodríguez-Fernández, L.; Lavandera, I.; Gotor-Fernández, V. Photocatalytic Oxidative Cleavage of Alkenes Followed by Carbonyl Stereoselective Bioreduction for the Synthesis of Enantioenriched Secondary Alcohols. *Adv Synth Catal* **2024**, *366* (4), 900–908. https://doi.org/10.1002/adsc.202301325.

(6) Chatalova-Sazepin, C.; Wang, Q.; Sammis, G. M.; Zhu, J. Copper-Catalyzed Intermolecular Carboetherification of Unactivated Alkenes by Alkyl Nitriles and Alcohols. *Angewandte Chemie* **2015**, *127* (18), 5533–5536. https://doi.org/10.1002/ange.201412357.

(7) Khan, I.; Reed-Berendt, B. G.; Melen, R. L.; Morrill, L. C. FLP-Catalyzed Transfer Hydrogenation of Silyl Enol Ethers. *Angewandte Chemie* **2018**, *130* (38), 12536–12539. https://doi.org/10.1002/ange.201808800.

(8) Zhao, J. F.; Tan, B. H.; Loh, T. P. In(III)-Pybox Complex Catalyzed Enantioselective Mukaiyama Aldol Reactions between Polymeric or Hydrated Glyoxylates and Enolsilanes Derived from Aryl Ketones. *Chem Sci* **2011**, *2* (2), 349–352. https://doi.org/10.1039/c0sc00454e.

(9) Cao, H.; Ma, S.; Feng, Y.; Guo, Y.; Jiao, P. Synthesis of β-Nitro Ketones from Geminal Bromonitroalkanes and Silyl Enol Ethers by Visible Light Photoredox Catalysis. *Chemical Communications* **2022**, *58* (11), 1780–1783. https://doi.org/10.1039/d1cc06529g.

(10) Kano, T.; Aota, Y.; Maruoka, K. Rate Acceleration of Solid-Liquid Phase-Transfer Catalysis by Rotor-Stator Homogenizer. *Adv Synth Catal* **2016**, *358* (18), 2996–2999. https://doi.org/10.1002/adsc.201600425.

(11) Grupe, S.; JacobivonWangelin, A. Iron-Catalyzed Synthesis of Cyclopropyl Halides. *ChemCatChem* **2013**, *5* (3), 706–710. https://doi.org/10.1002/cctc.201200740.

(12) Pan, J.; Qu, H.; Li, Y.; Bu, X. L.; Deng, H. P.; Gong, H.; Ma, M.; Xu, L.; Xue, F. Switchable Divergent Electrochemical Hydrodehalogenation of Gem-Dihalocyclopropanes. *Journal of Organic Chemistry* **2024**. https://doi.org/10.1021/acs.joc.4c01748.

(13) Xu, B.; Troian-Gautier, L.; Dykstra, R.; Martin, R. T.; Gutierrez, O.; Tambar, U. K. Photocatalyzed Diastereoselective Isomerization of Cinnamyl Chlorides to Cyclopropanes. *J Am Chem Soc* **2020**, *142* (13), 6206–6215. https://doi.org/10.1021/jacs.0c00147.

(14) Mendel, M.; Gnägi, L.; Dabranskaya, U.; Schoenebeck, F. Rapid and Modular Access to Vinyl Cyclopropanes Enabled by Air-Stable Palladium(I) Dimer Catalysis. *Angewandte Chemie - International Edition* **2023**, *62* (7). https://doi.org/10.1002/anie.202211167.

(15) Zhang, X.; Cheng, X. Electrochemical Reductive Functionalization of Alkenes with Deuterochloroform as a One-Carbon Deuteration Block. *Org Lett* **2022**, *24* (47), 8645–8650. https://doi.org/10.1021/acs.orglett.2c03443.

(16) Shintani, R.; Iino, R.; Nozaki, K. Rhodium-Catalyzed Polymerization of 3,3-Diarylcyclopropenes Involving a 1,4-Rhodium Migration. *J Am Chem Soc* **2014**, *136* (22), 7849–7852. https://doi.org/10.1021/ja5032002.

(17) Hao, T.; Shen, J.; Wei, Y.; Shi, M. Alkene Difunctionalization Enabled by Photocatalytic Hydrogen Atom Transfer from Haloalkane α-C(Sp3)–H Bonds. *Chem Catalysis* **2023**, *3* (12). https://doi.org/10.1016/j.checat.2023.100807.

(18) He, R.; Deng, M.-Z. *Selective Reduction of Stereodefined Cyclopropyl Substituted Acrylate Esters to the Corresponding Propionate Esters*.

(19) Murakami, Y.; Hisaeda, Y.; Kajihara, A.; Ohno, T. Hydrophobic Vitamin B12. II. Coordination Geometry and Redox Behavior of Heptamethyl Cobyrinate in Nonaqueous Media. *Bull Chem Soc Jpn* **1984**, *57* (2), 405–411. https://doi.org/10.1246/bcsj.57.405.

(20) Shimakoshi, H.; Sakumori, E.; Kaneko, K.; Hisaeda, Y. B 12-TiO 2 Hybrid Catalyst for Dehalogenation of Organic Halides. *Chem Lett* **2009**, *38* (5), 468–469. https://doi.org/10.1246/cl.2009.468.

(21) Schrauzer, G. N.; Lee, L. P.; Sibertlb, J. W.; Sibert, J. W.; Windgassen, R. J.; Amer, J. *Nakajima10 and Boyd and Singer*; UTC, 1968; Vol. 90. https://pubs.acs.org/sharingguidelines.

K. NMR Spectra

2: Crude ^{1}H NMR (400 MHz, $CDCl_3$)

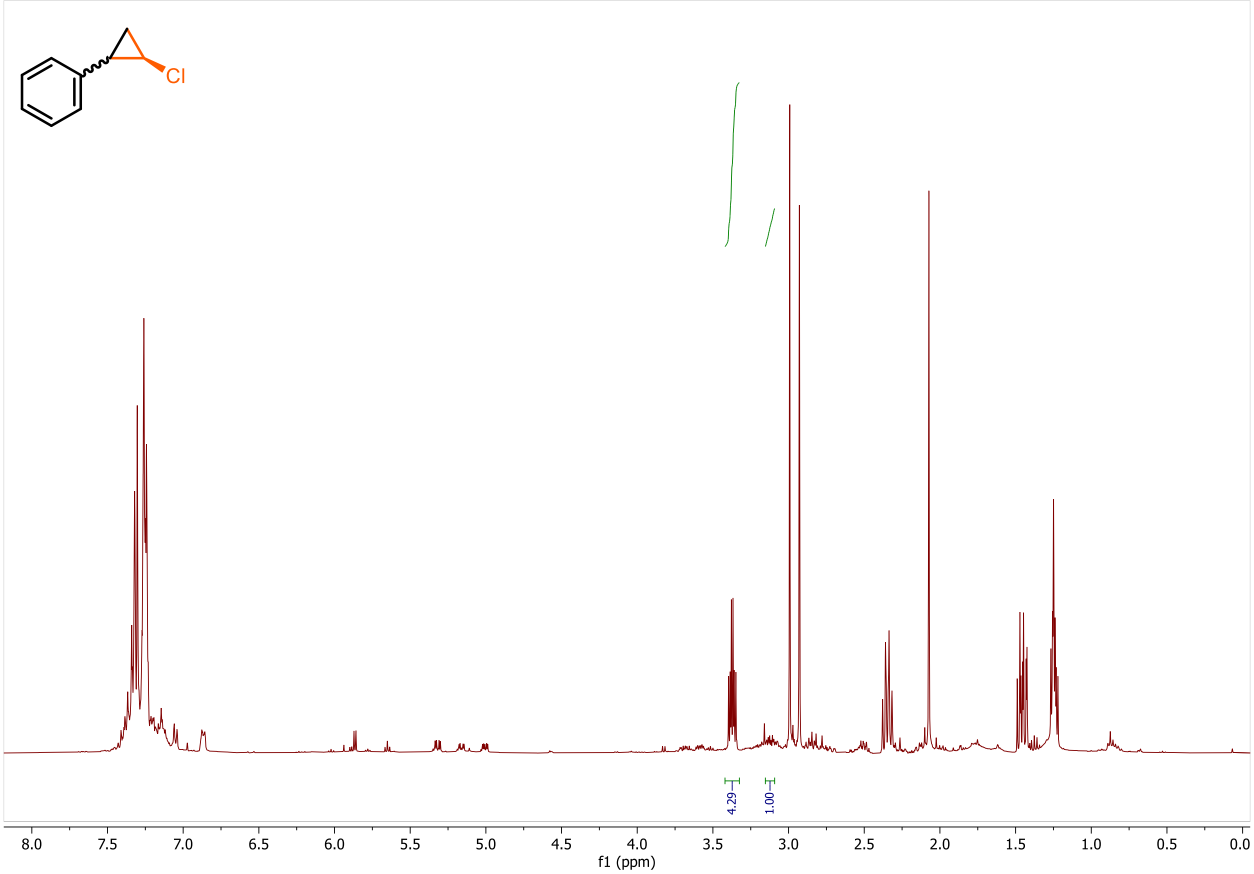

2: **^{1}H NMR** (800 MHz, $CDCl_3$)

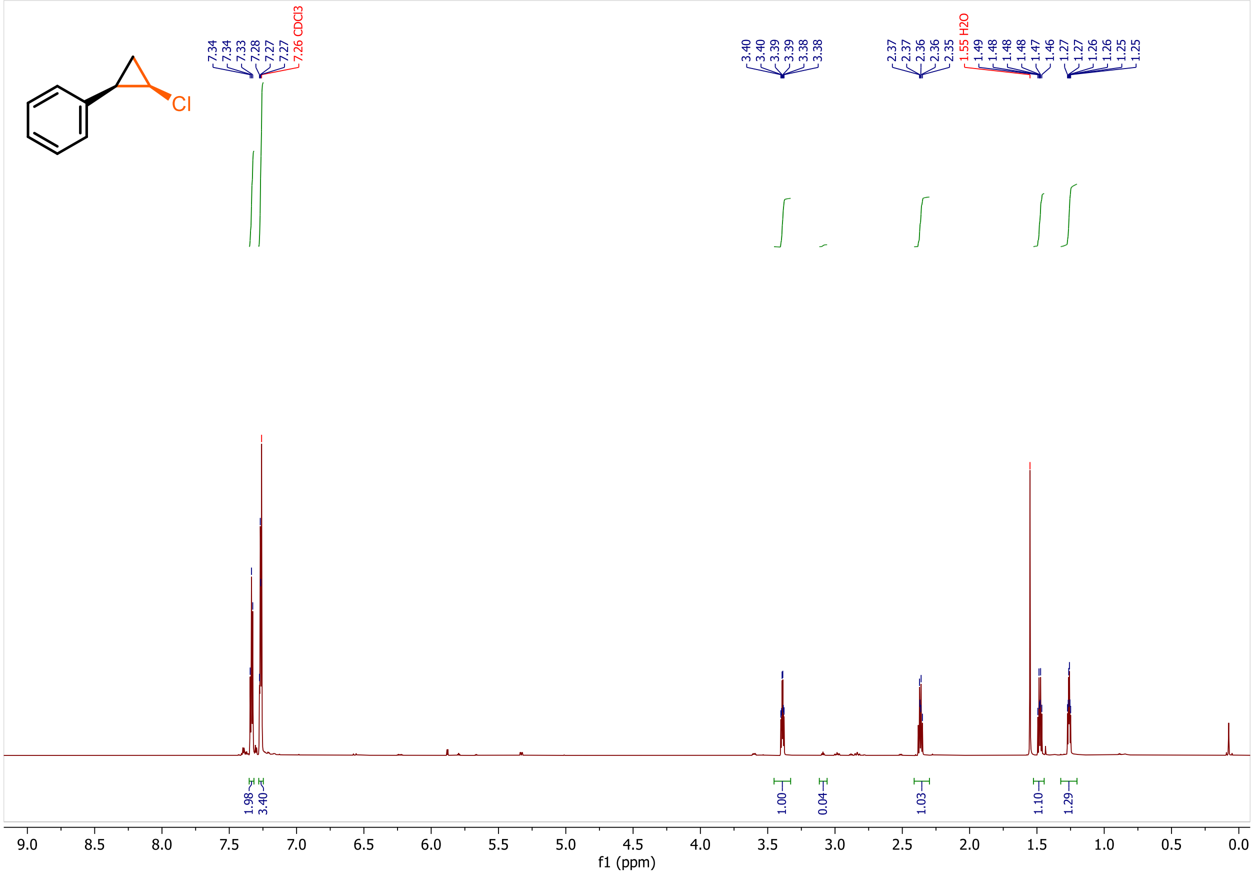

2: ^{13}C NMR (201 MHz, $CDCl_3$)

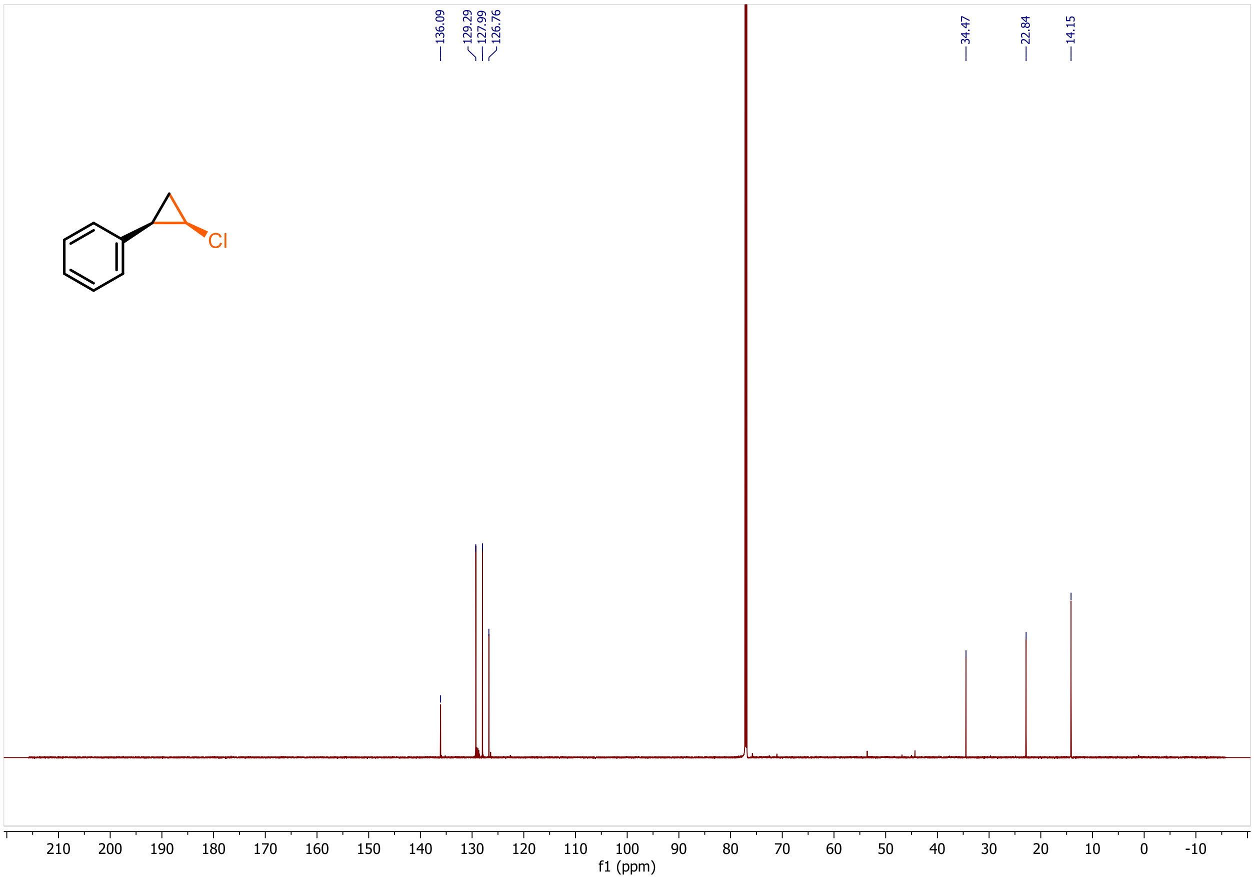

3: Crude ^{1}H NMR (400 MHz, $CDCl_3$)

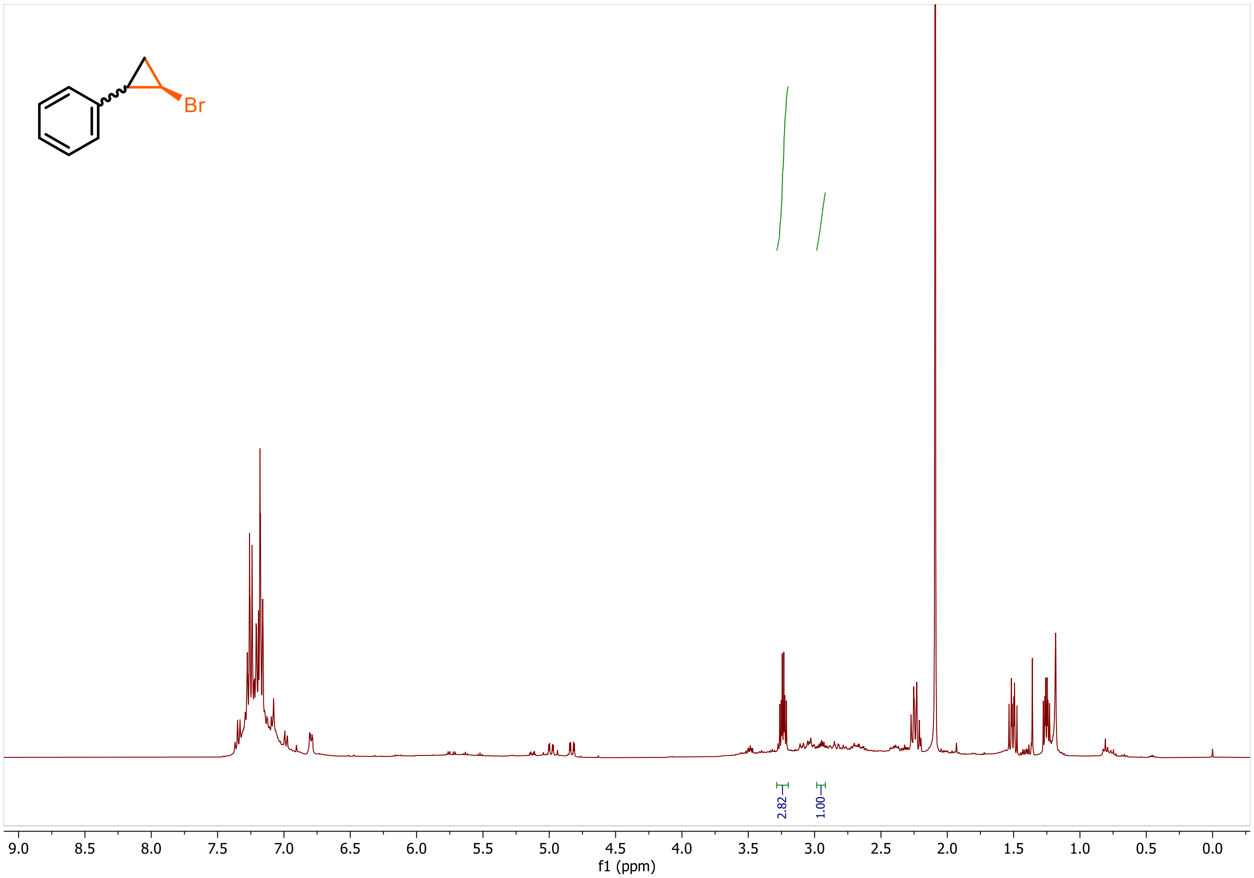

3: ^{1}H NMR (800 MHz, $CDCl_3$)

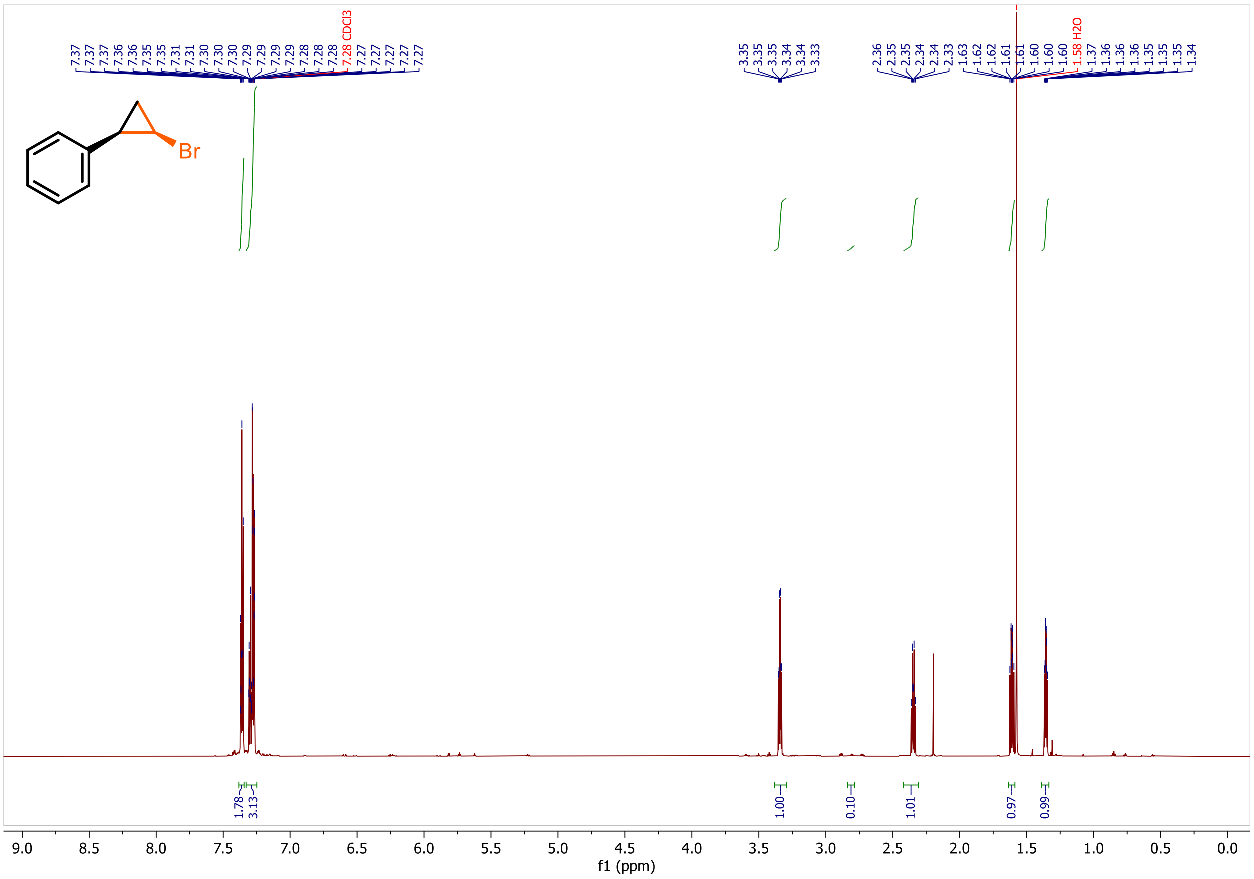

3: ^{13}C NMR (200 MHz, $CDCl_3$)

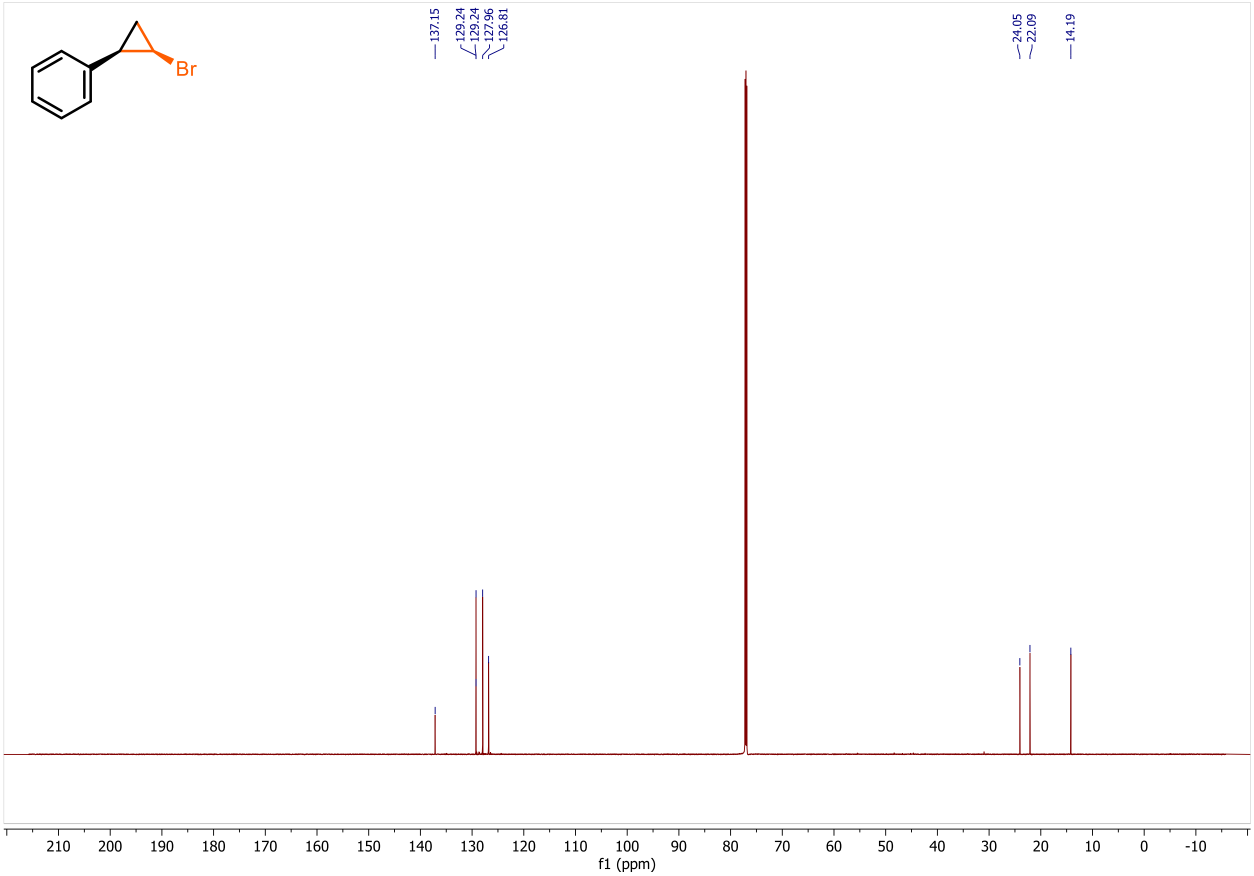

5: Crude ^{1}H NMR (400 MHz, $CDCl_3$)

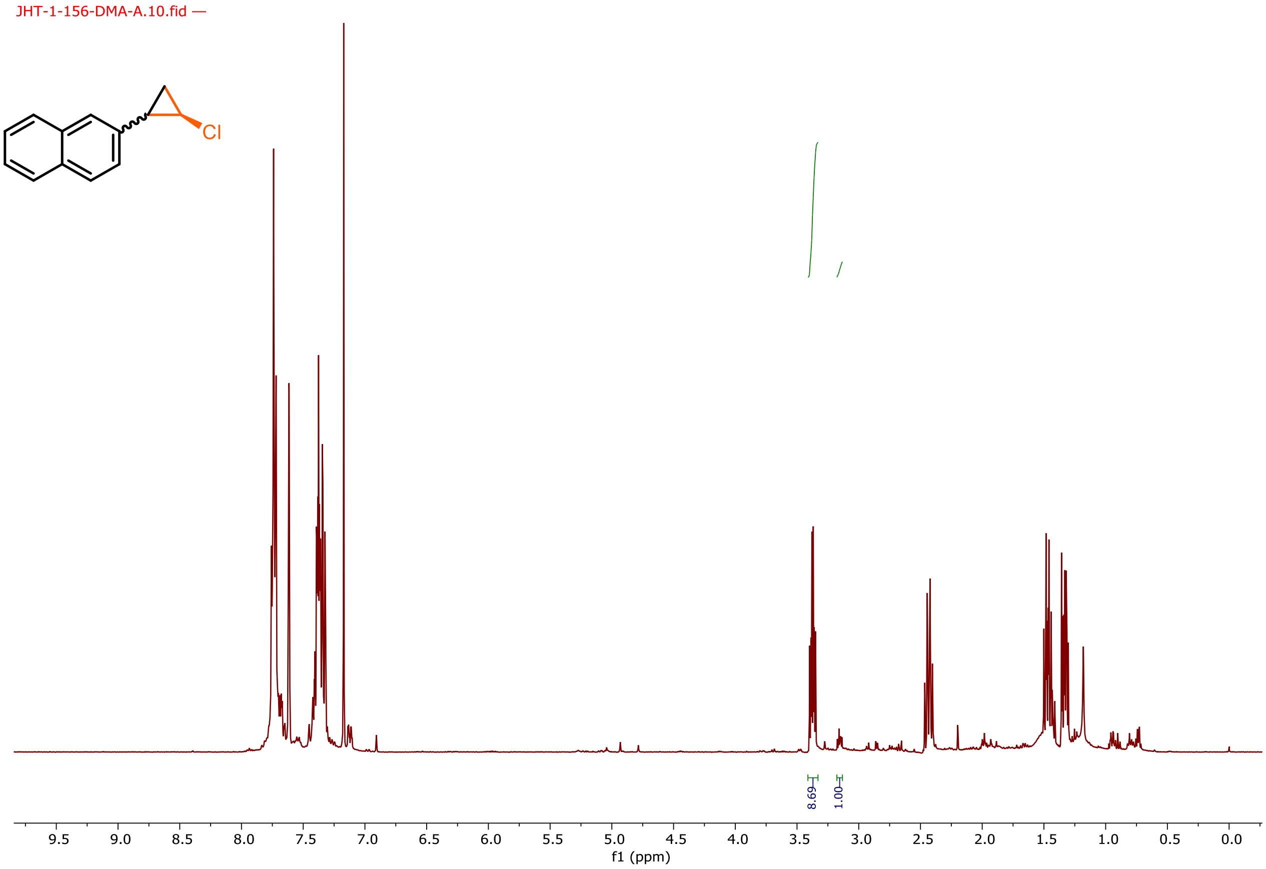

5: ^{1}H NMR (400 MHz, $CDCl_3$)

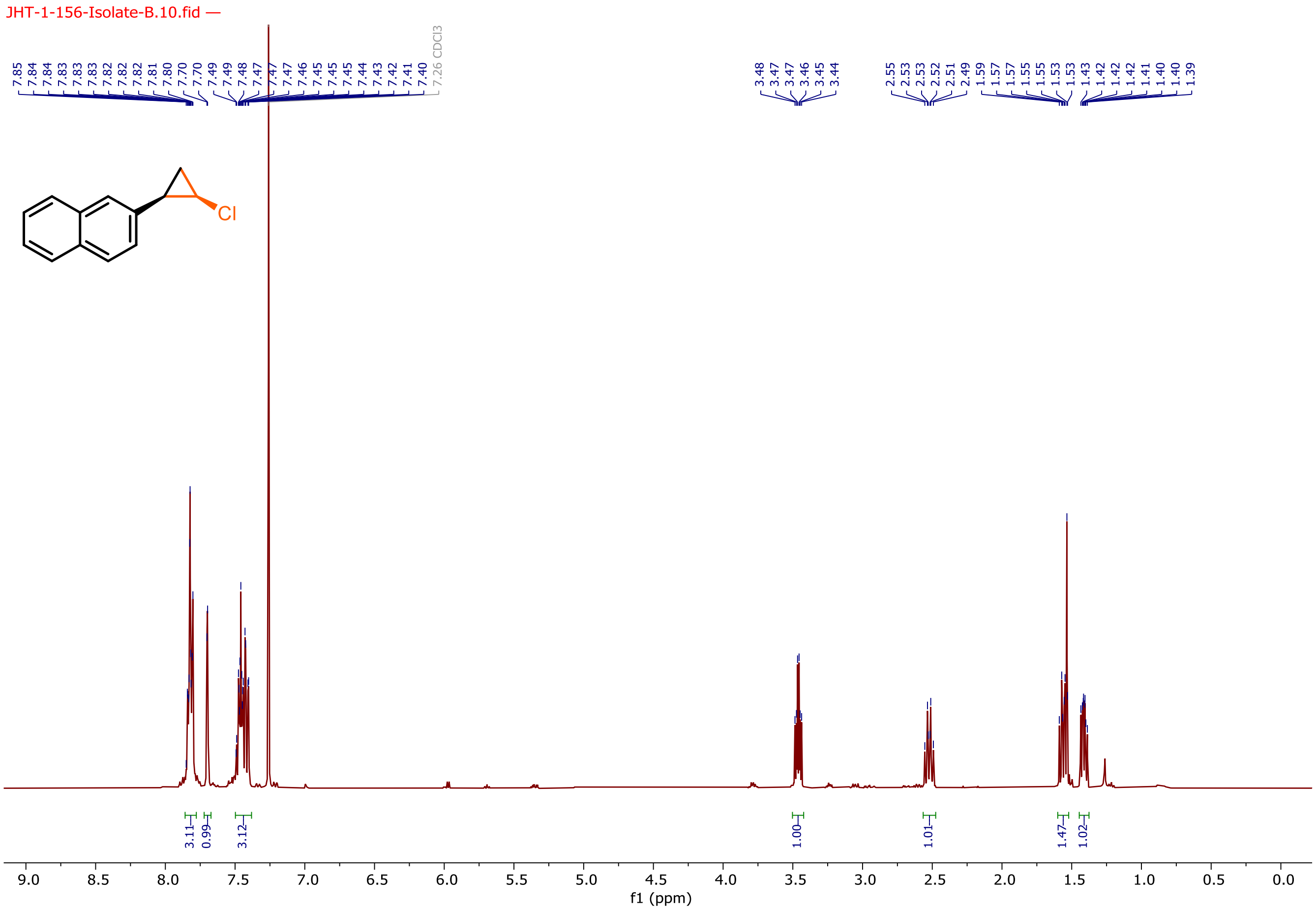

5: ^{13}C NMR (101 MHz, $CDCl_3$)

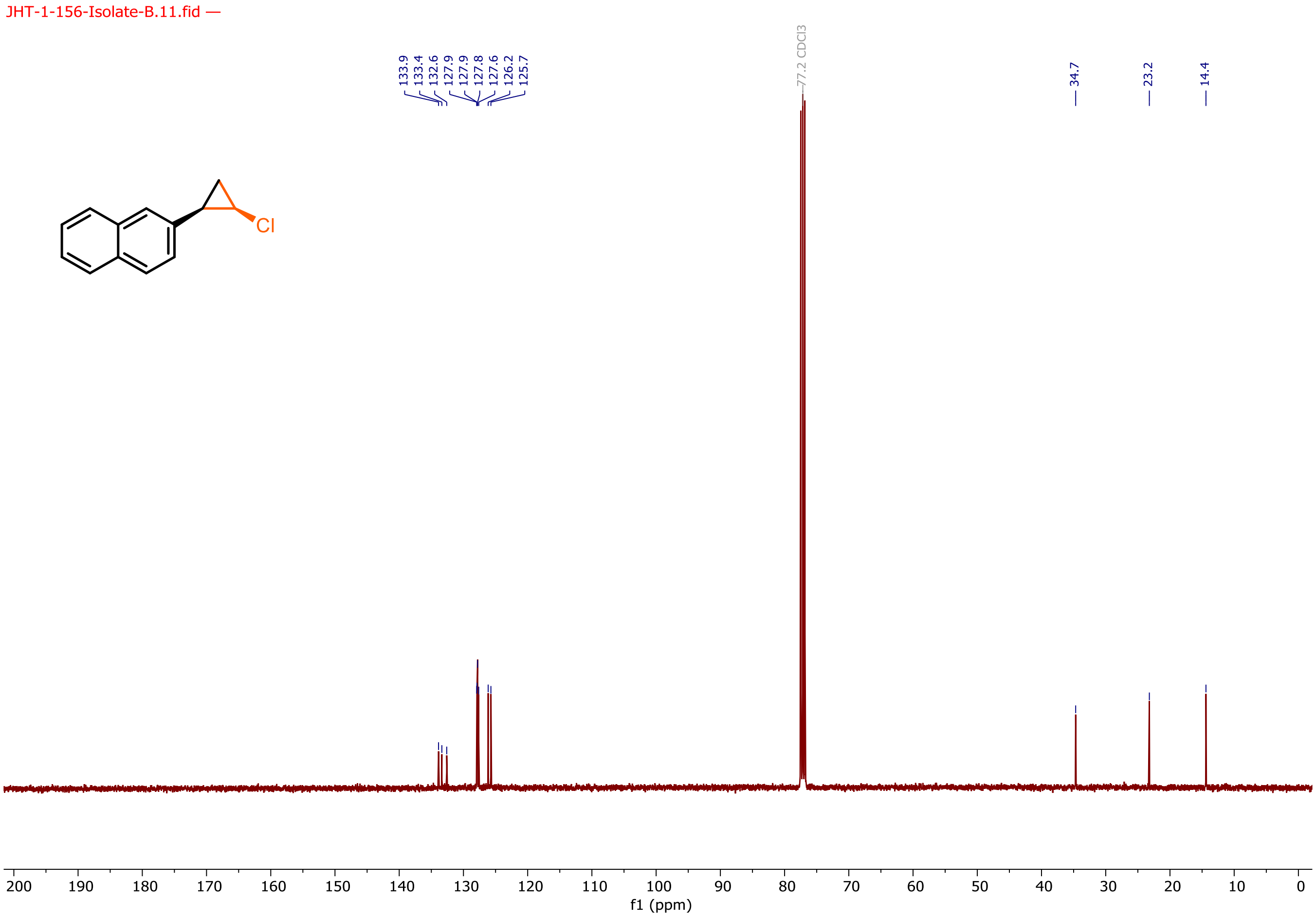

6: Crude ^{1}H NMR (400 MHz, $CDCl_3$)

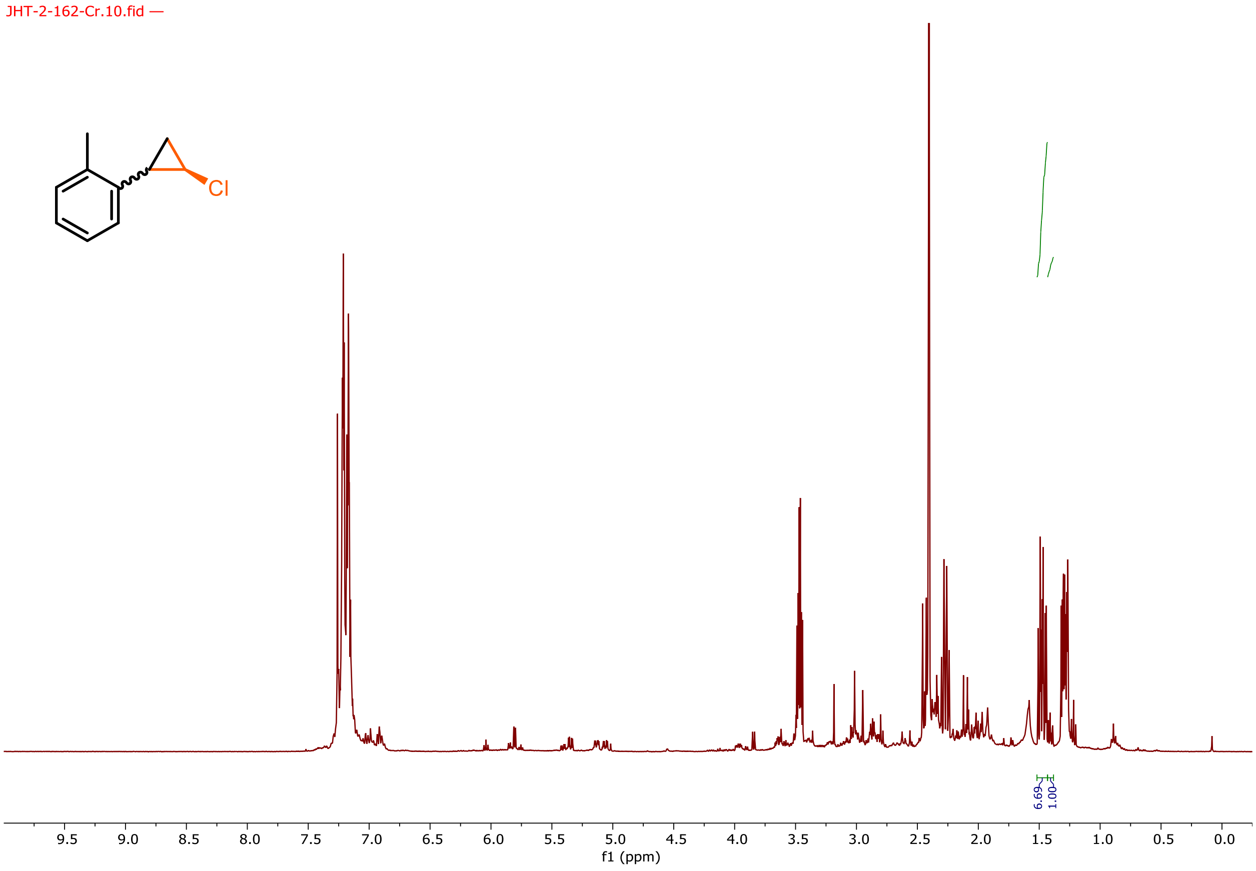

6: ^{1}H NMR (400 MHz, $CDCl_3$)

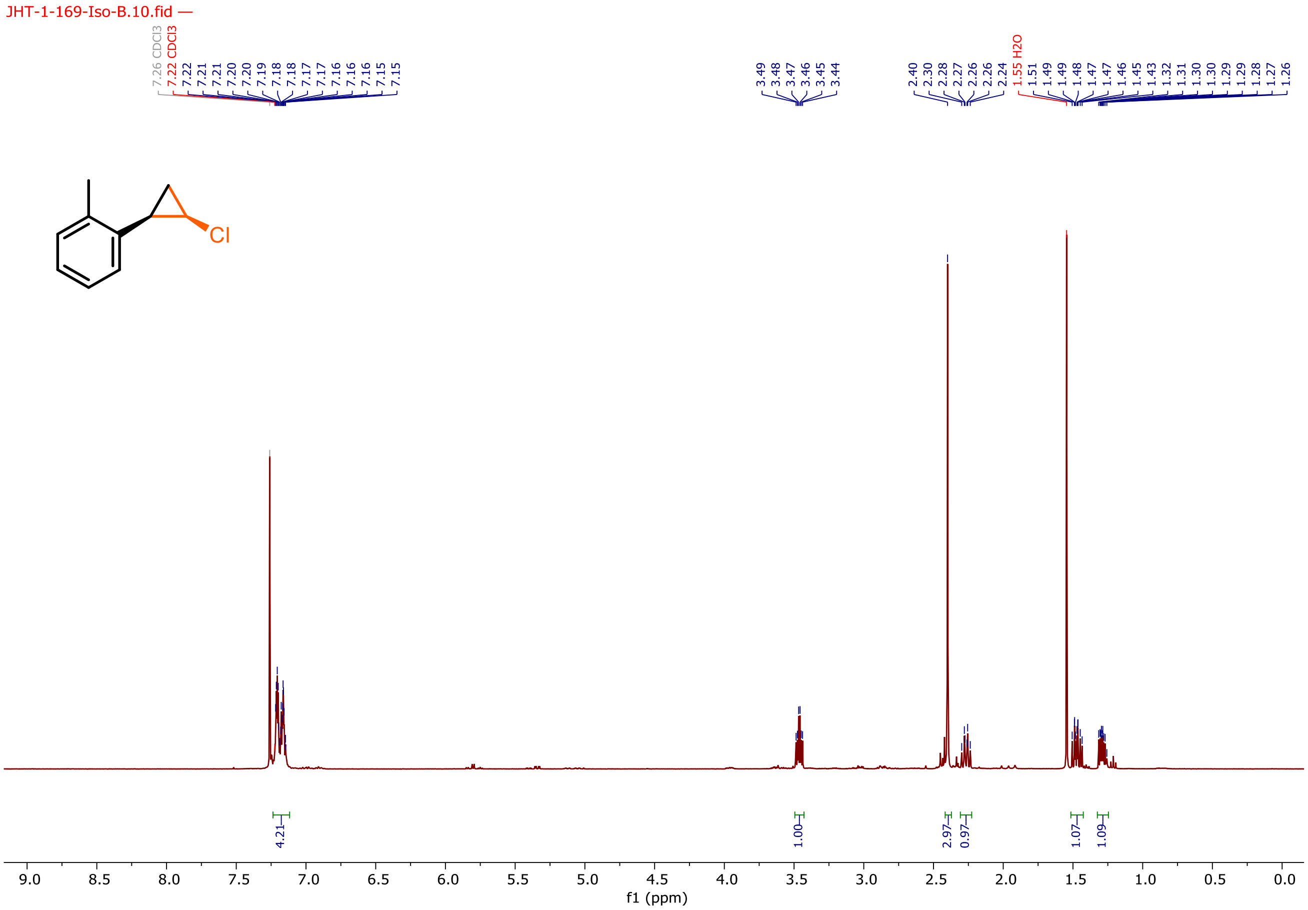

6: ^{13}C NMR (101 MHz, $CDCl_3$)

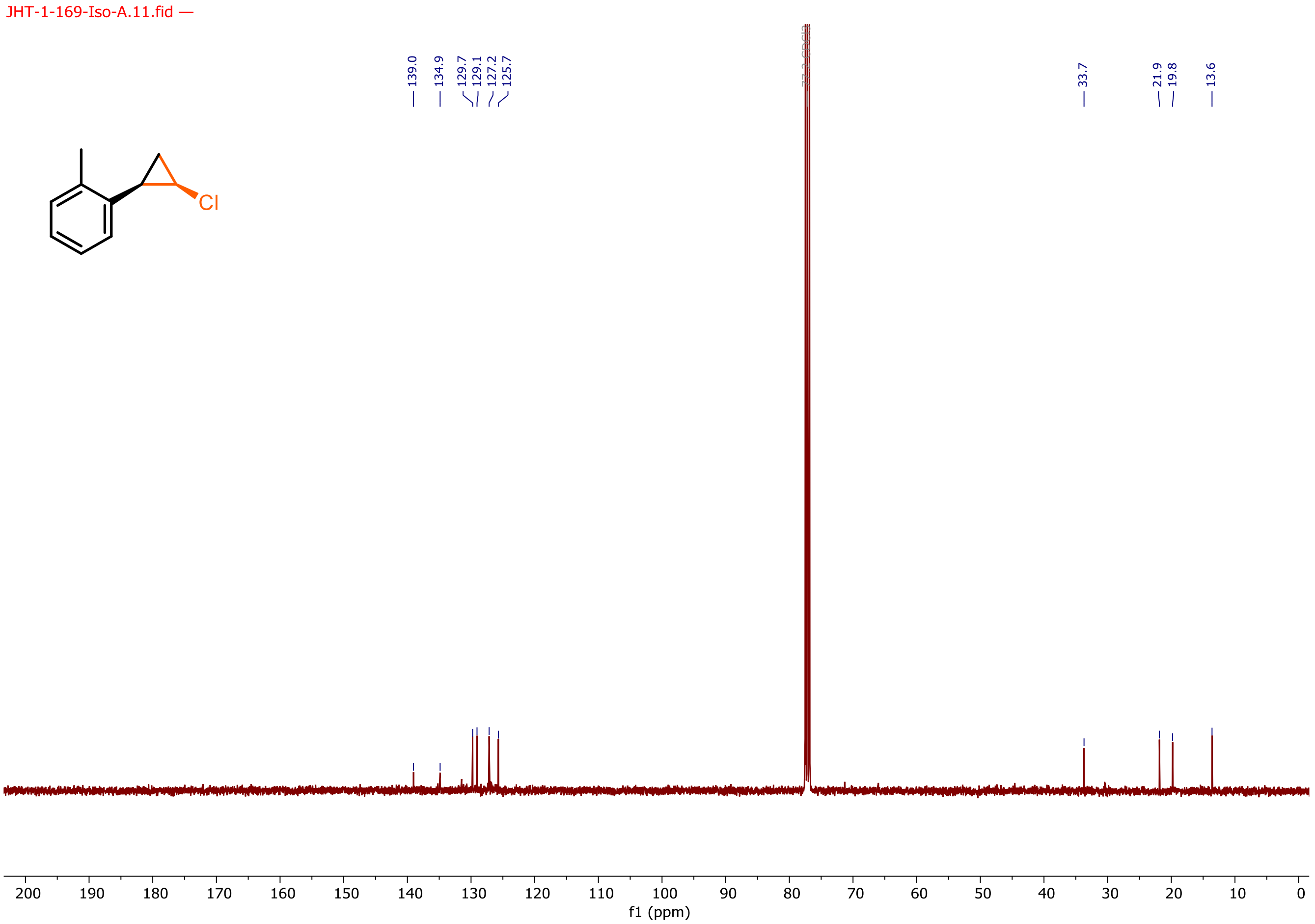

6: 2D NOESY NMR (400 MHz, $CDCl_3$)

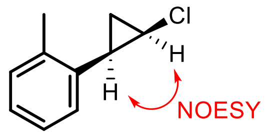

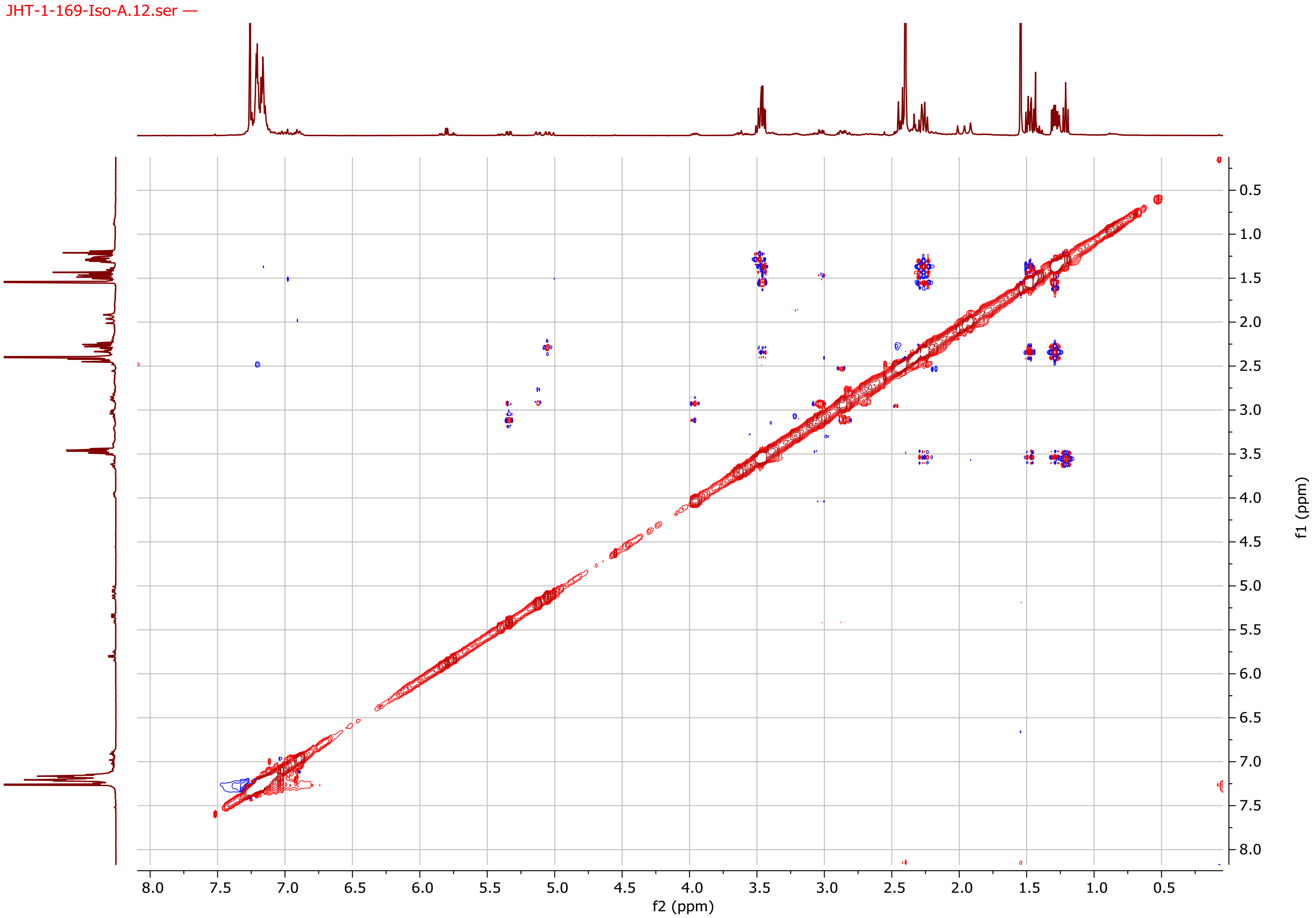

7: Crude ^{1}H NMR (400 MHz, $CDCl_3$)

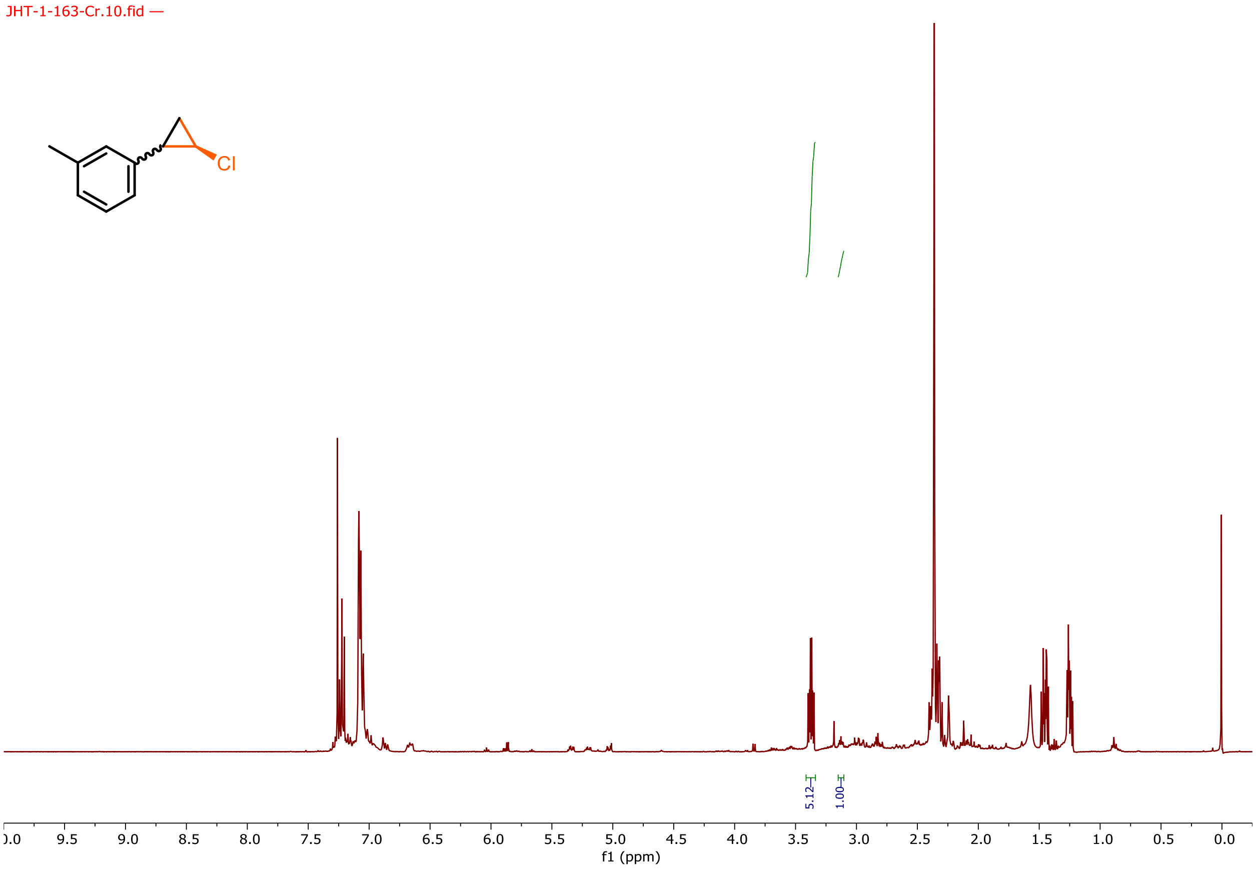

7: ^{1}H NMR (400 MHz, $CDCl_3$)

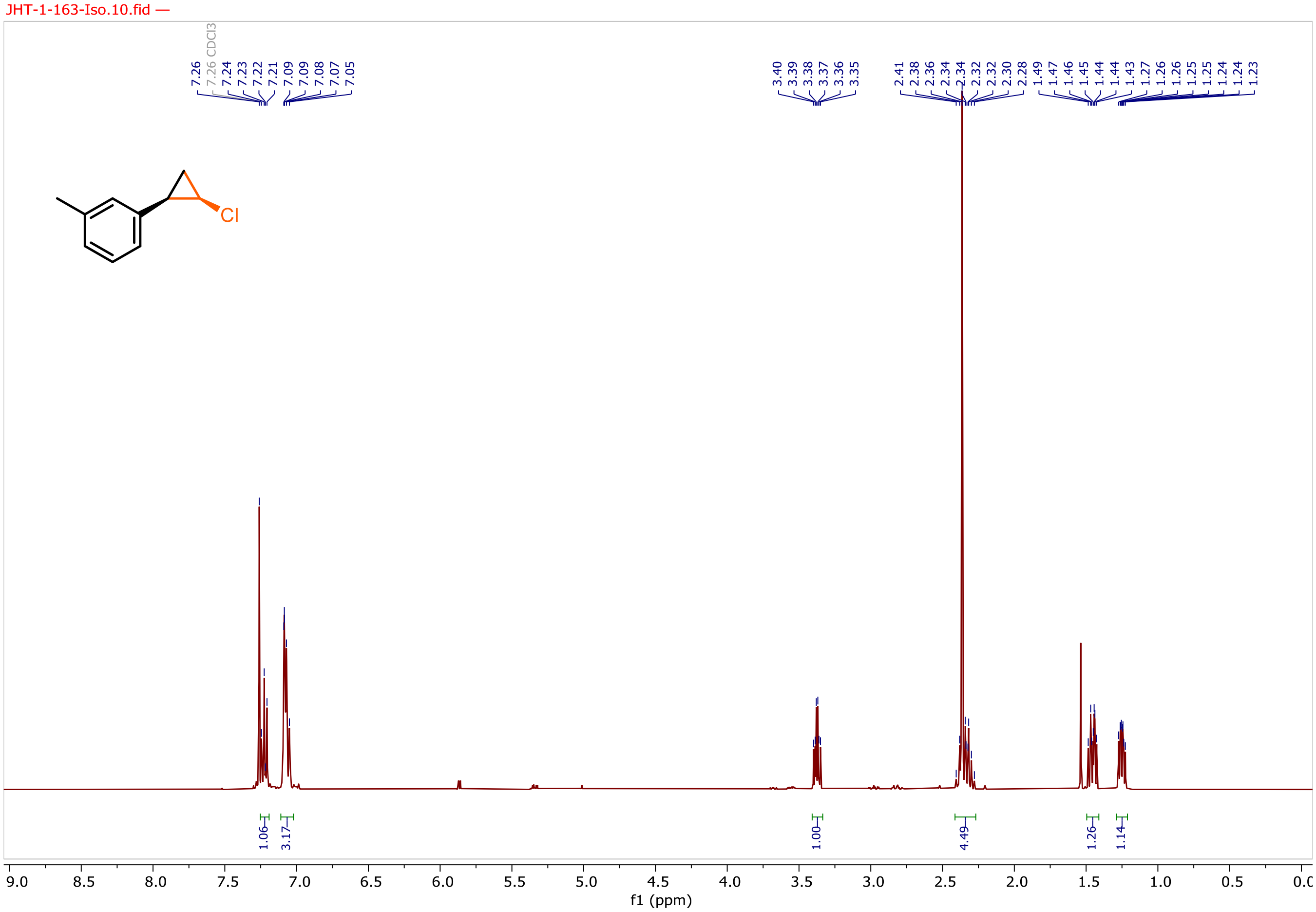

7: NOESY NMR (400 MHz, $CDCl_3$)

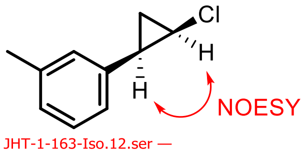

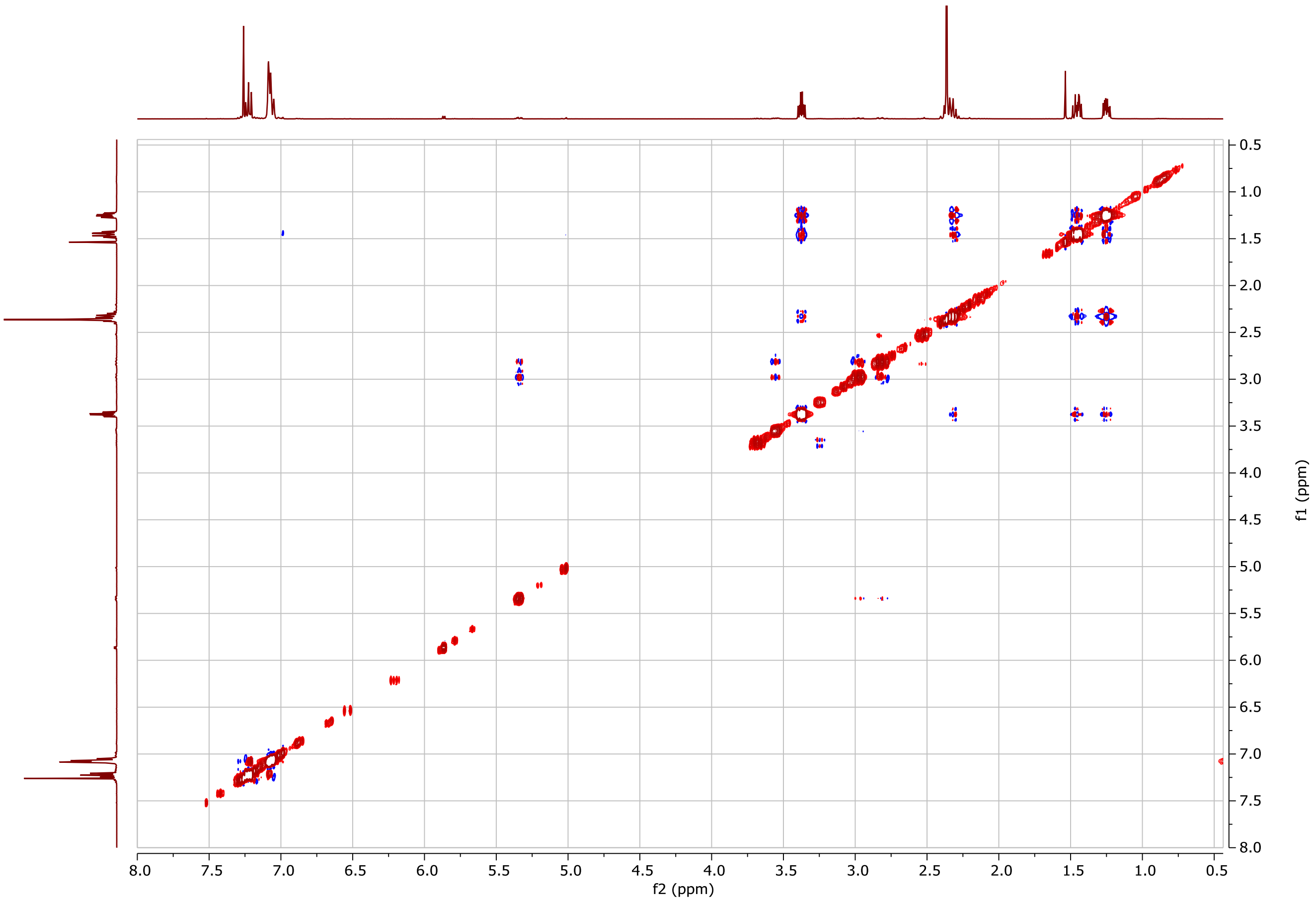

7: ^{13}C NMR (101 MHz, $CDCl_3$)

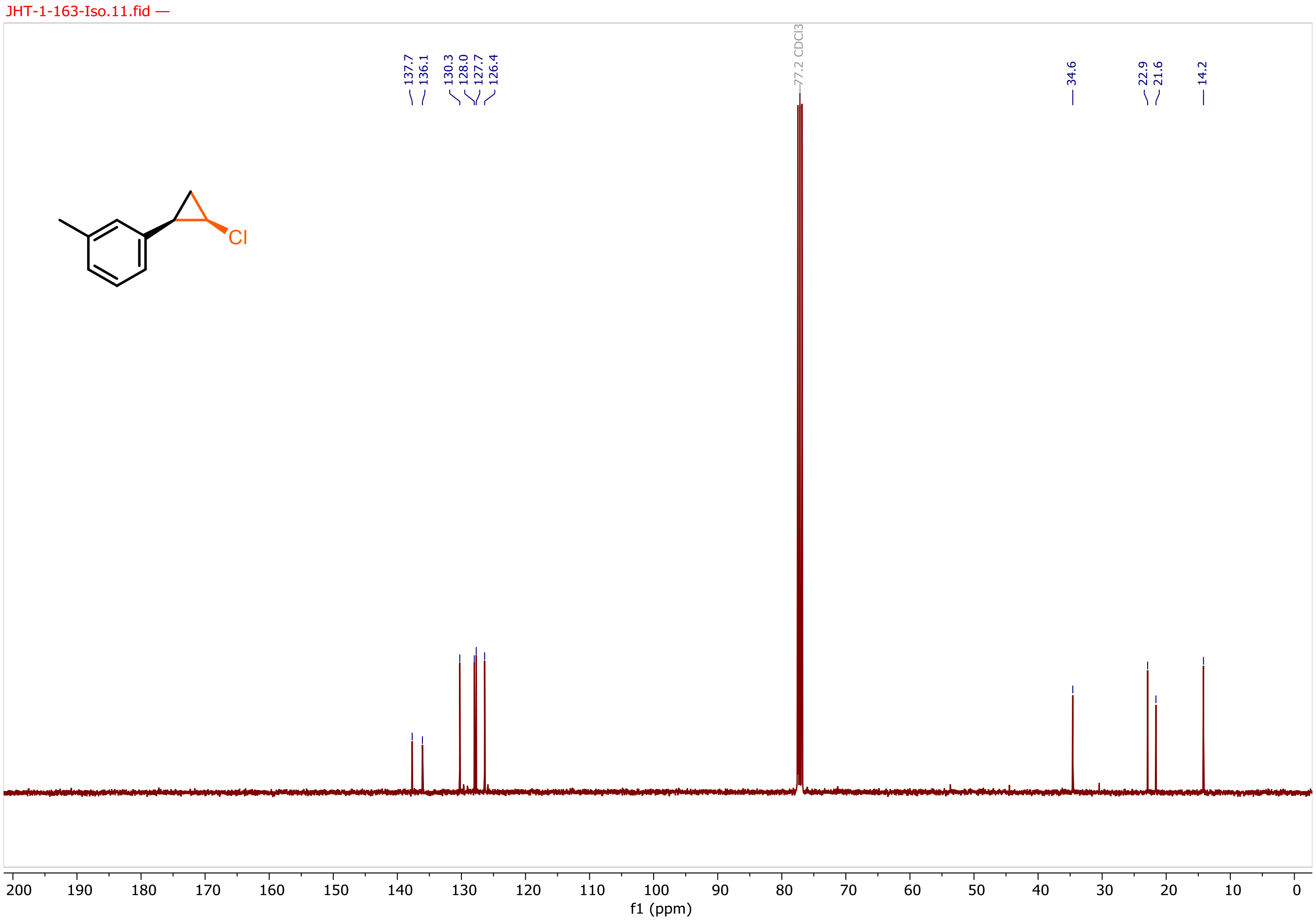

8: Crude ^{1}H NMR (400 MHz, $CDCl_3$)

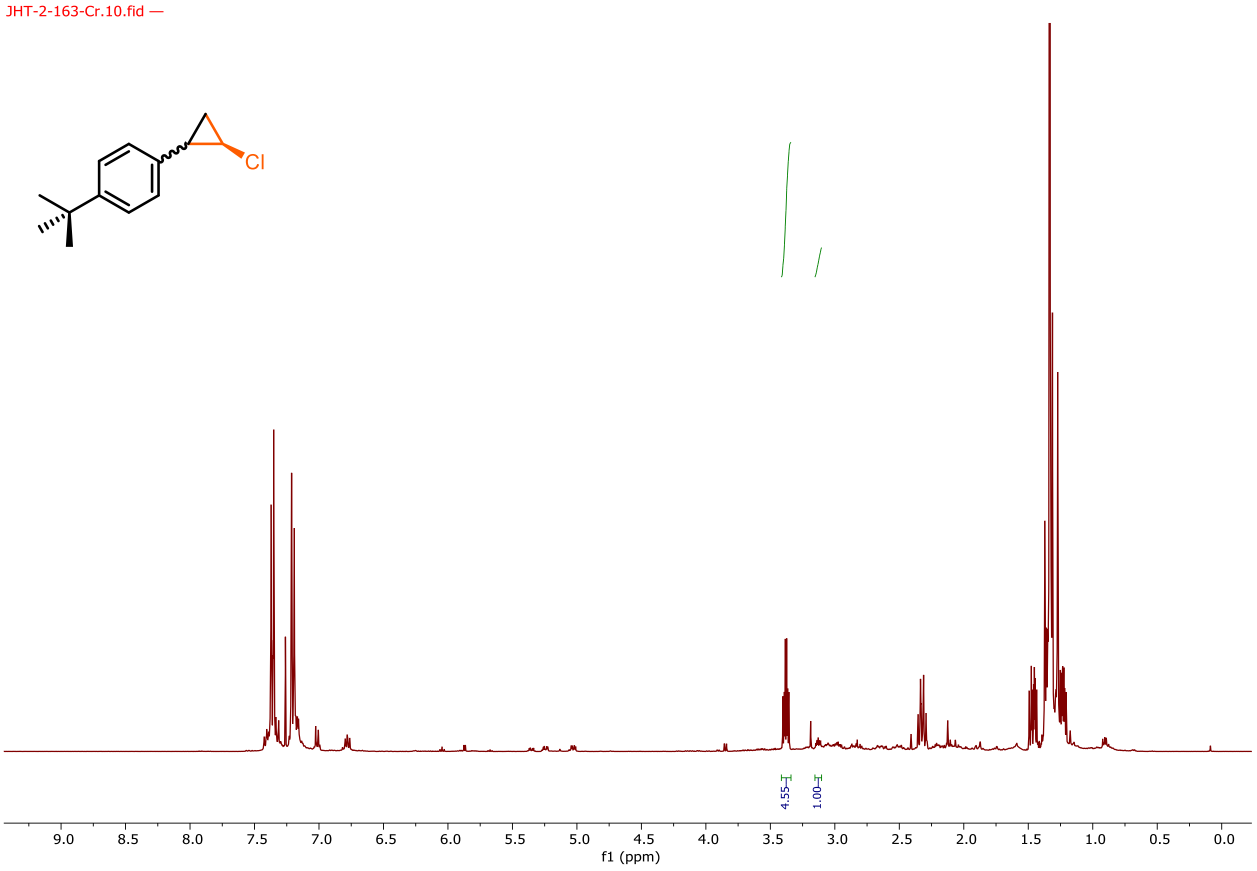

8: ^{1}H NMR (400 MHz, $CDCl_3$)

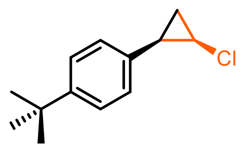

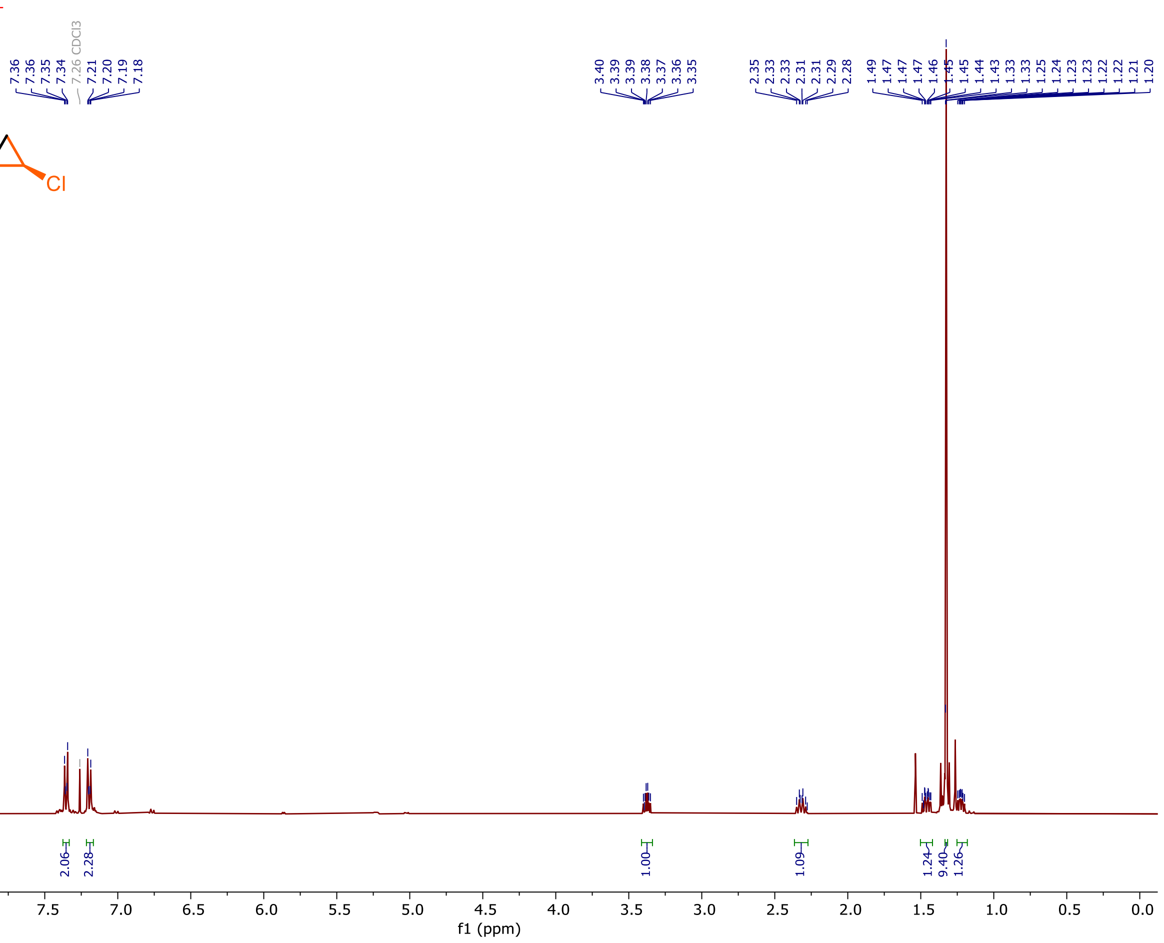

8: ^{13}C NMR (101 MHz, $CDCl_3$)

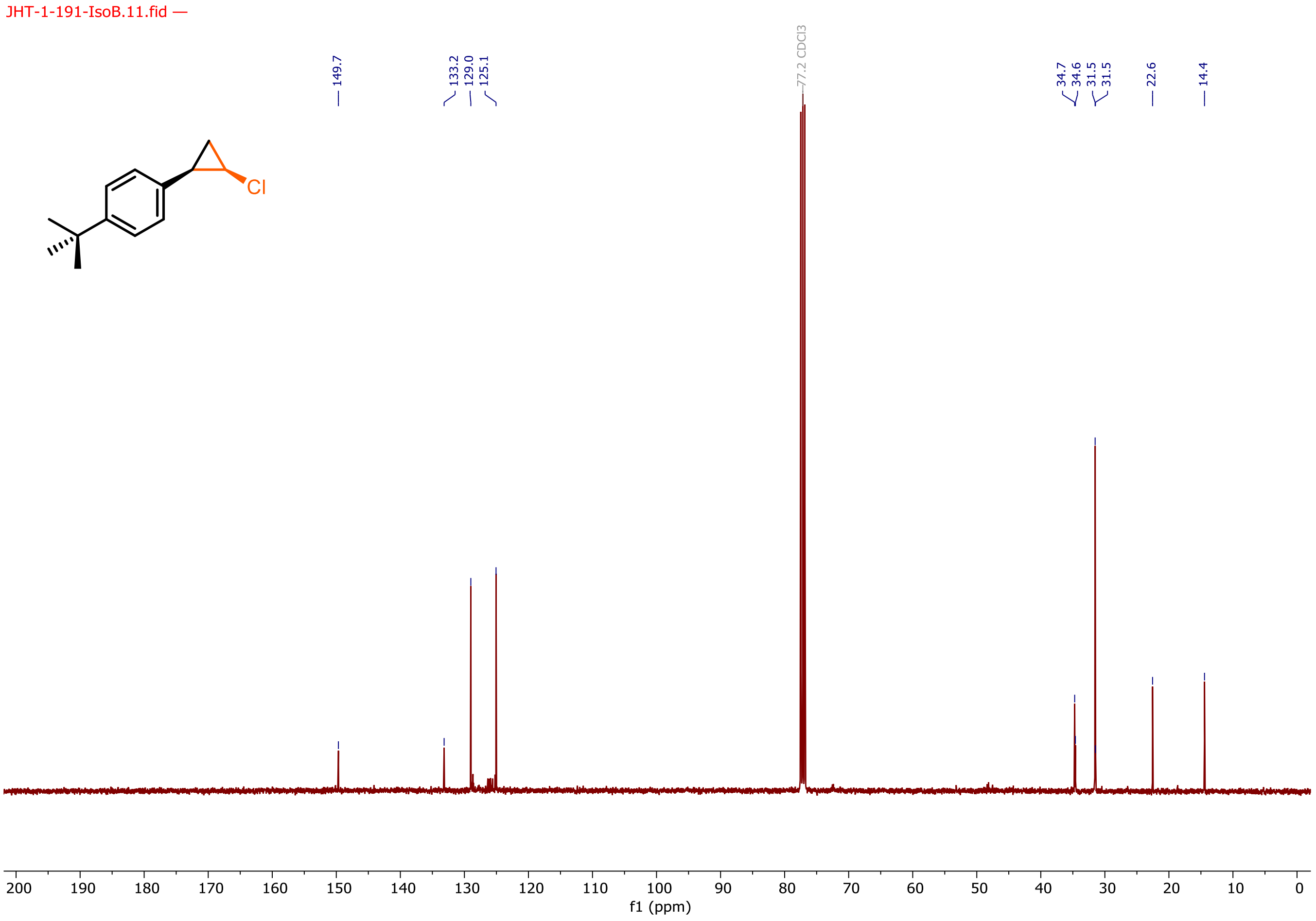

9: Crude ^{1}H NMR (400 MHz, $CDCl_3$)

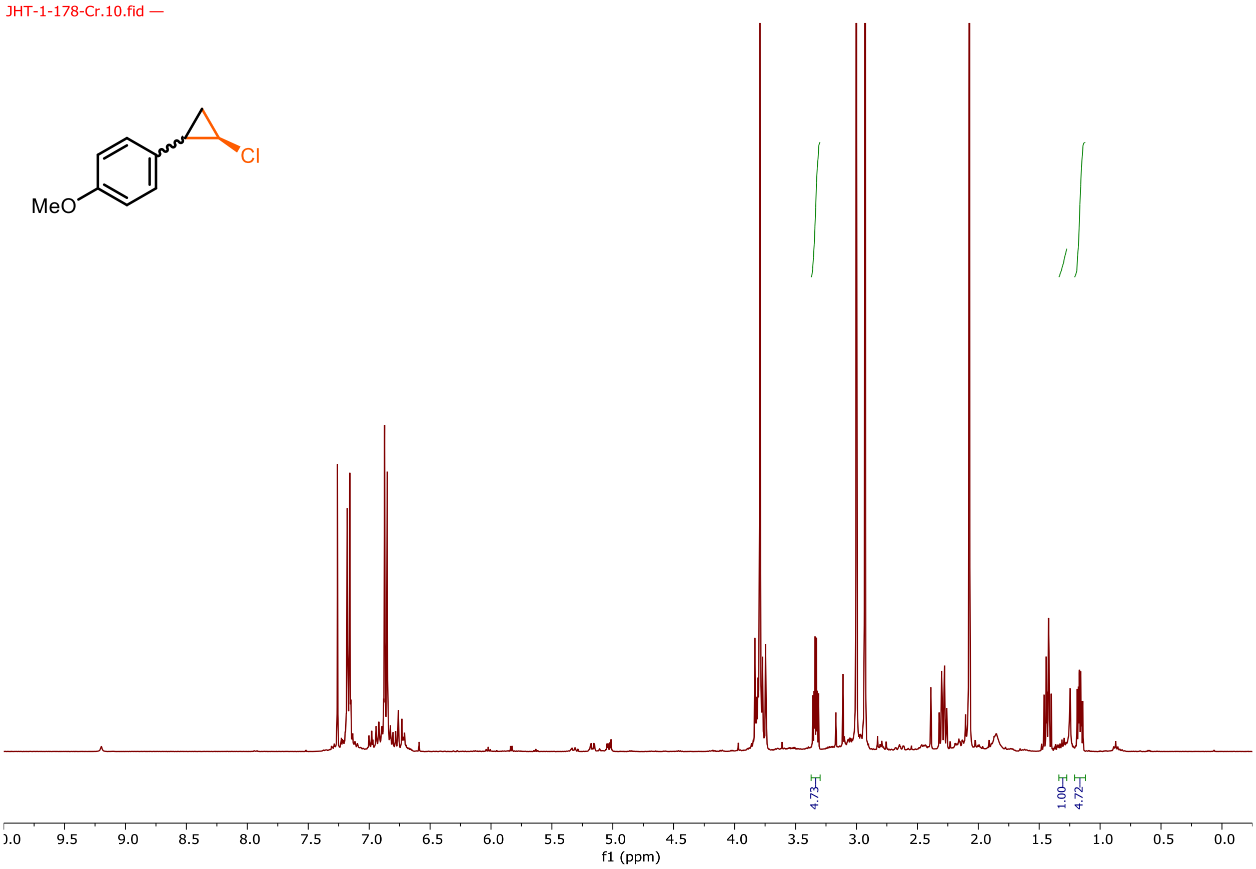

9: ^{1}H NMR (400 MHz, $CDCl_3$)

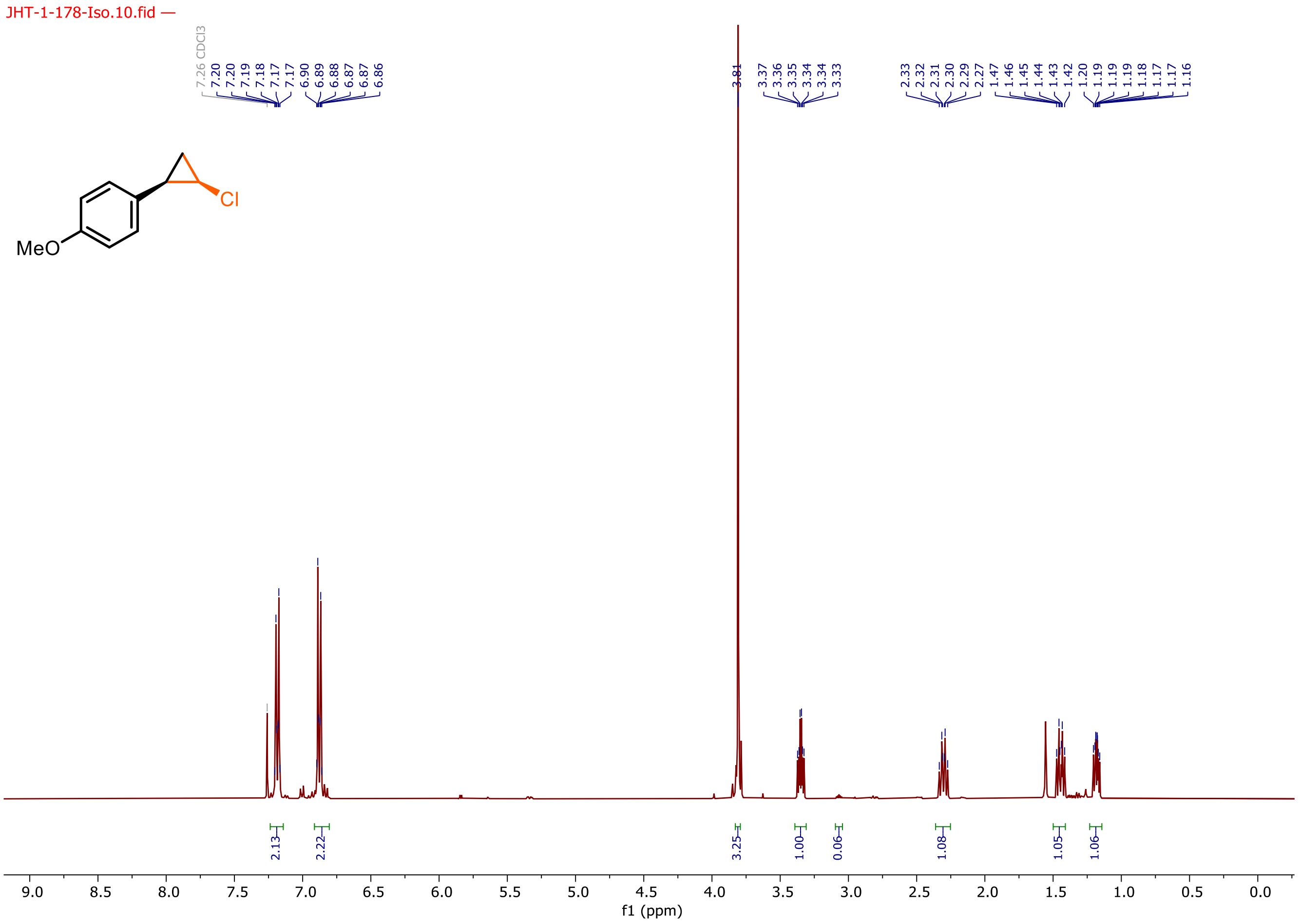

9: ^{13}C NMR (101 MHz, $CDCl_3$)

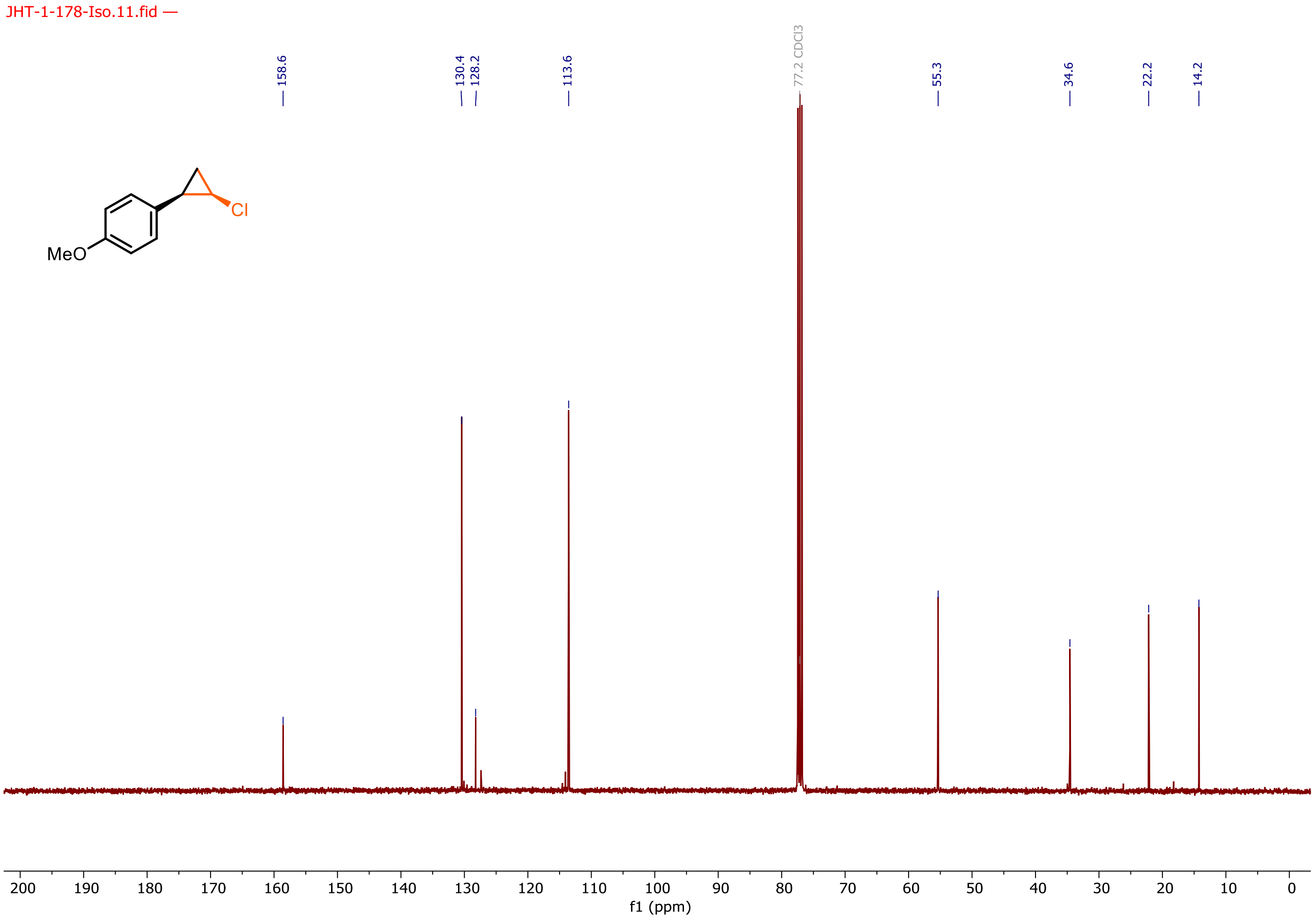

10: Crude ^{1}H NMR (400 MHz, $CDCl_3$)

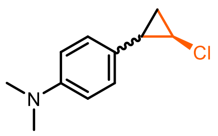

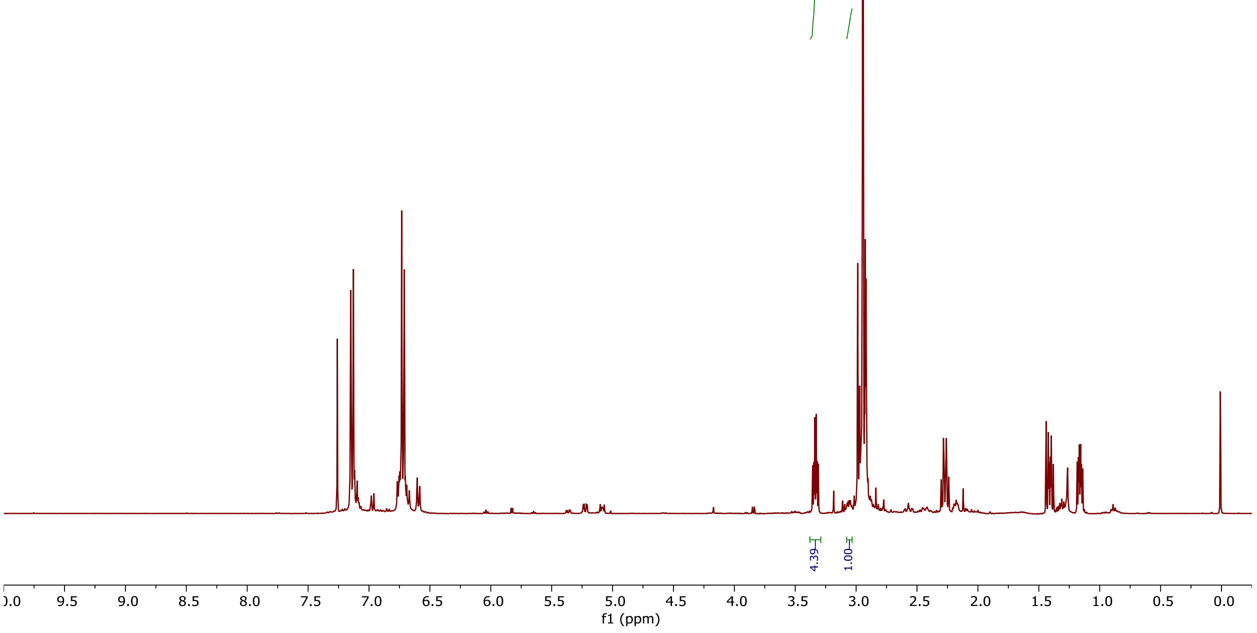

10: ^{1}H NMR (400 MHz, $CDCl_3$)

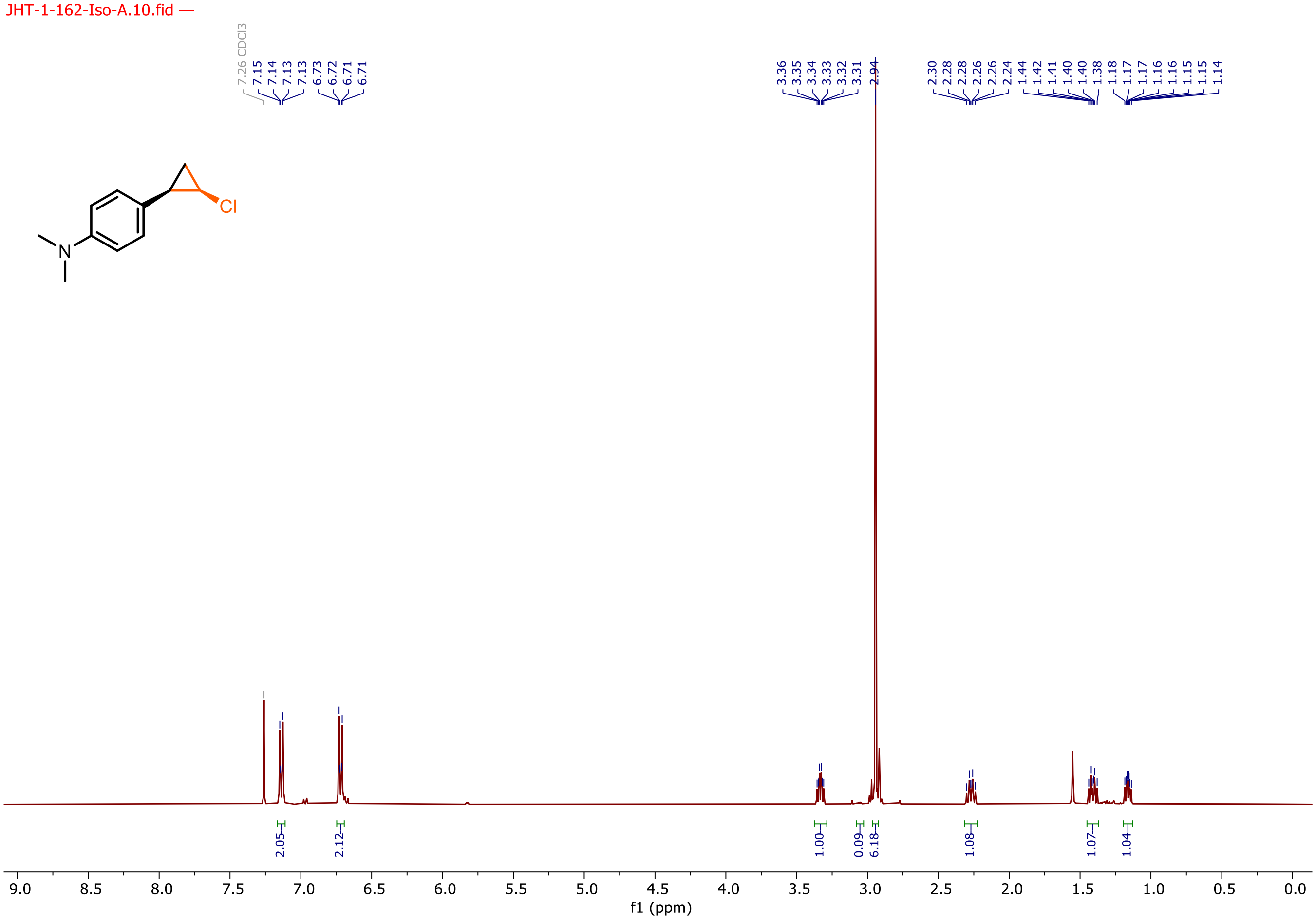

10: ^{13}C NMR (101 MHz, $CDCl_3$)

`

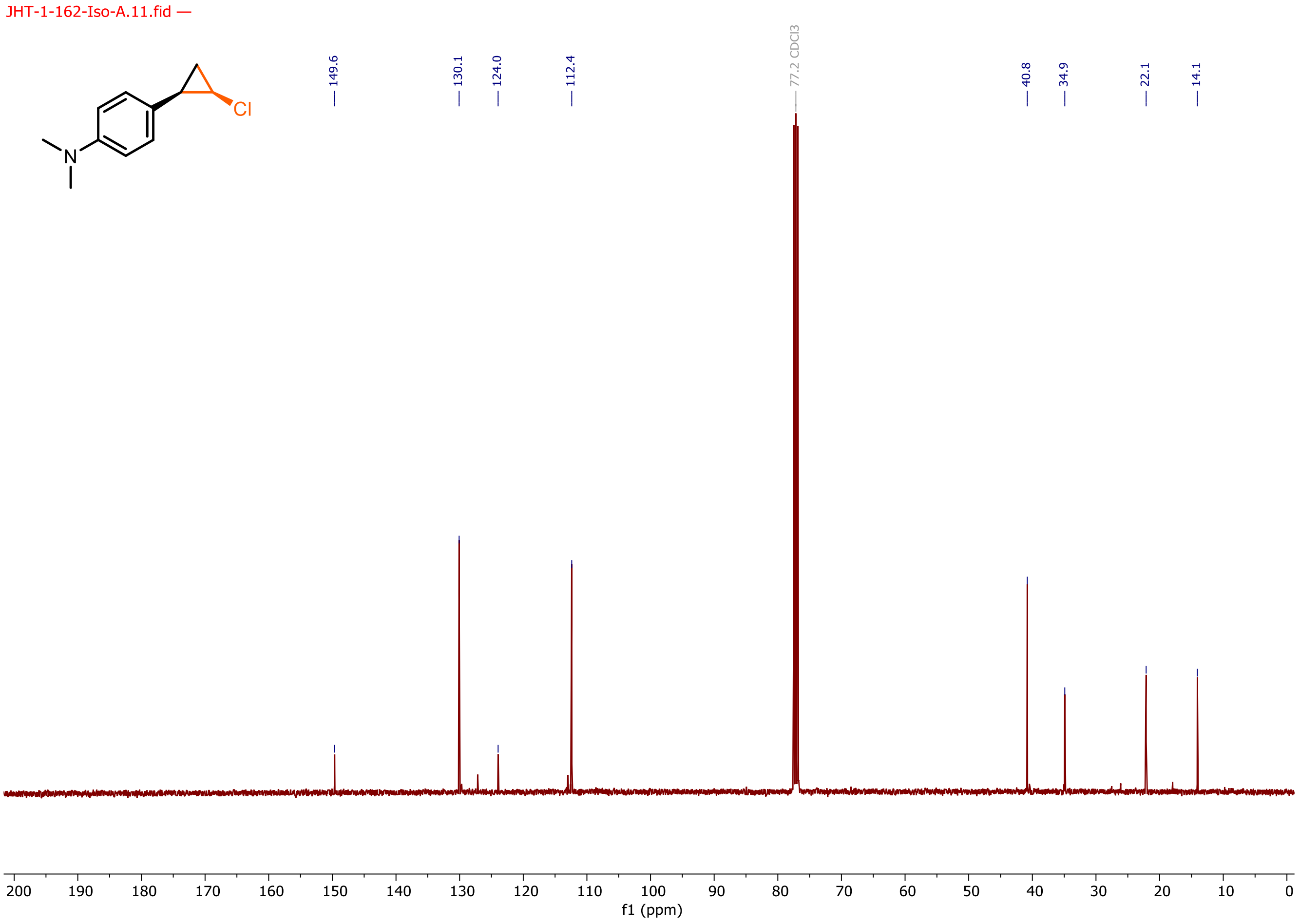

10: NOESY NMR (400 MHz, $CDCl_3$)

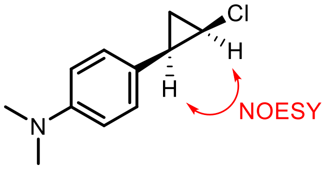

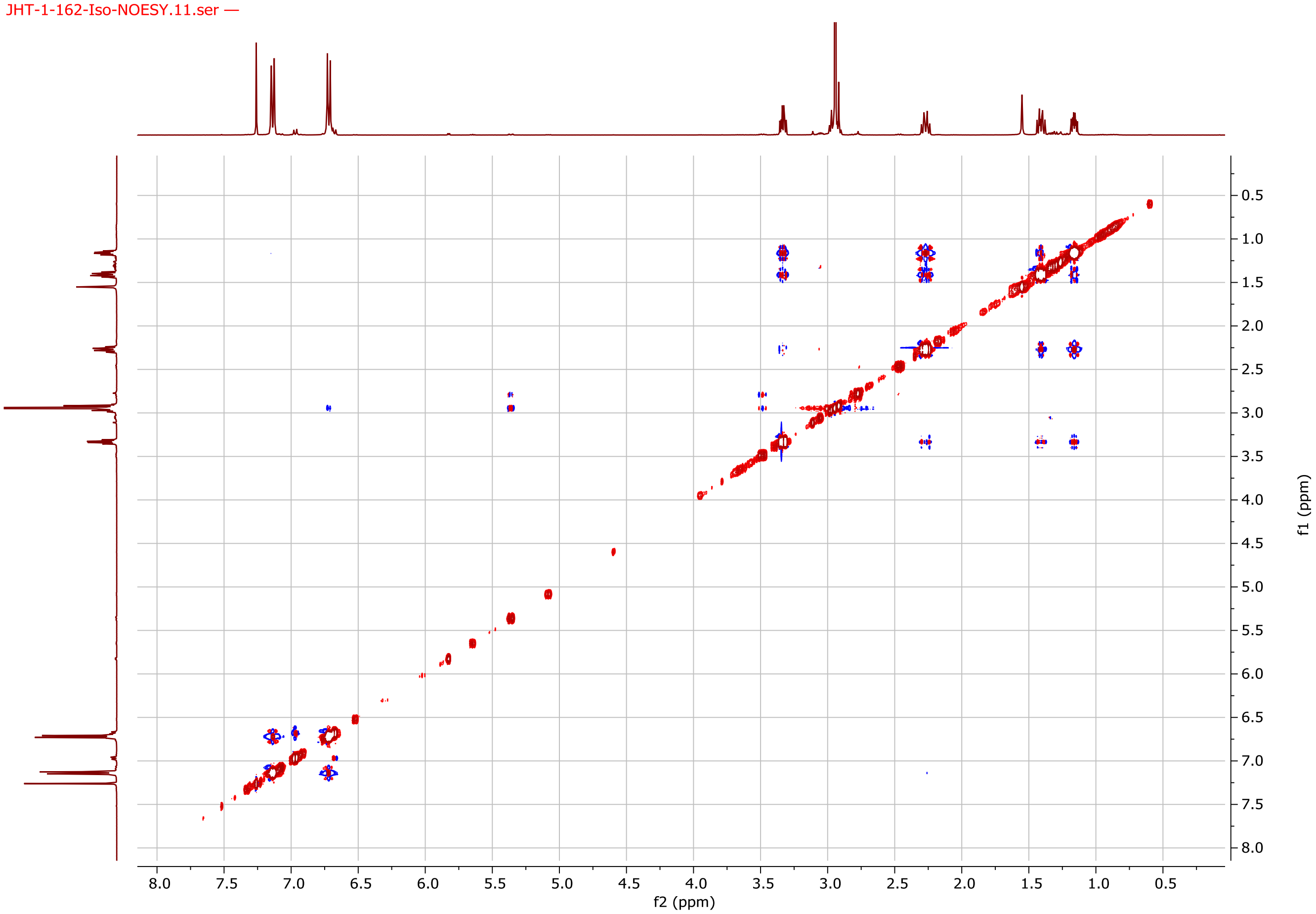

11: Crude ^{1}H NMR (400 MHz, $CDCl_3$)

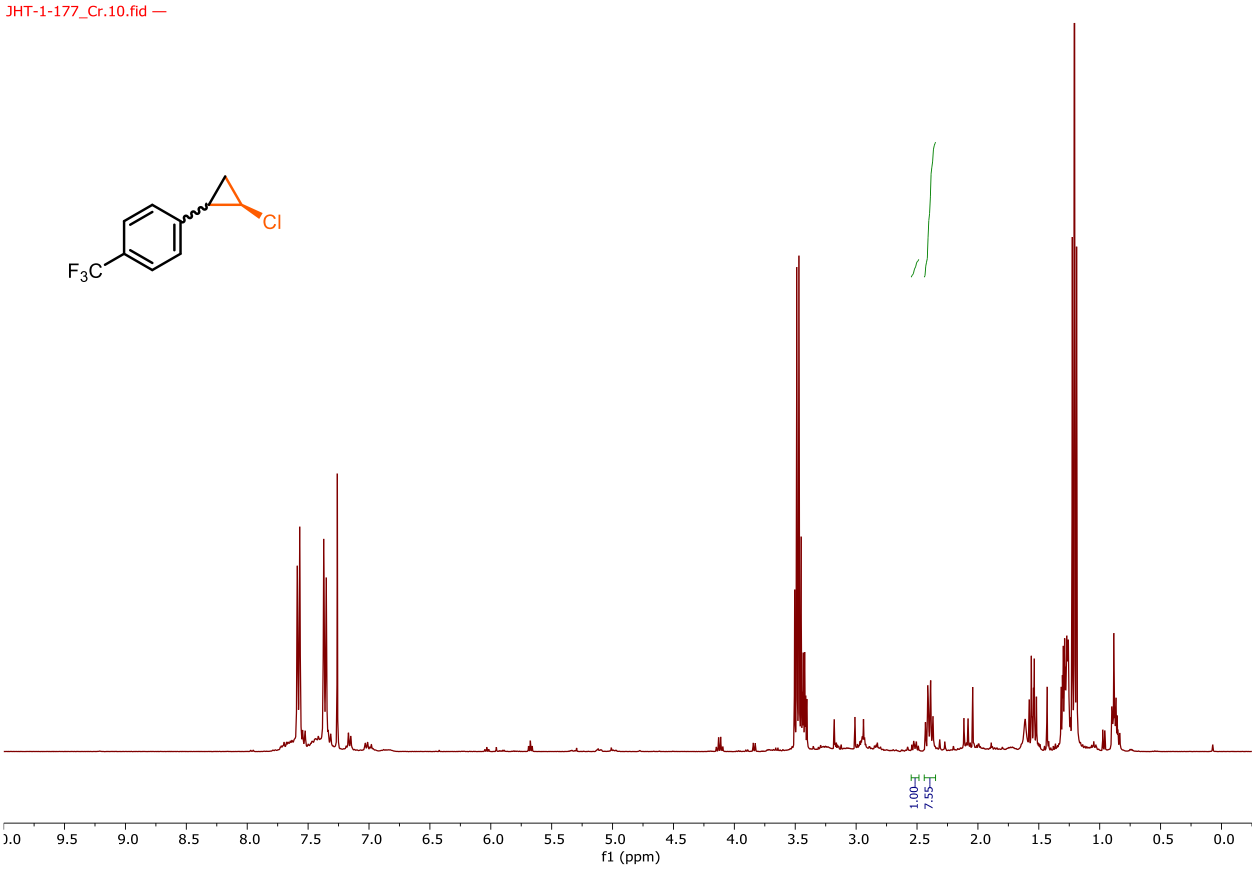

11: ^{1}H NMR (400 MHz, $CDCl_3$)

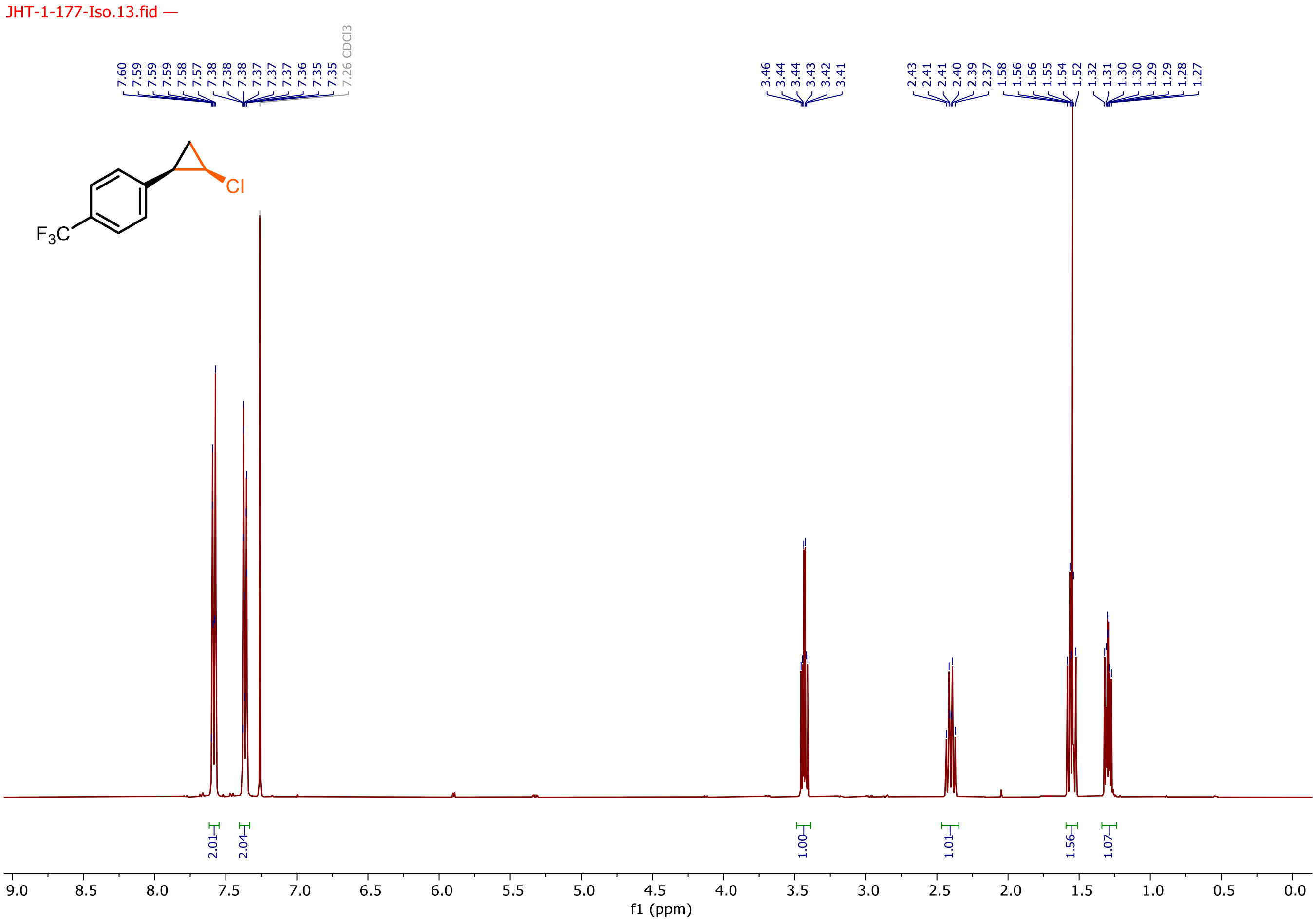

11: ^{13}C NMR (101 MHz, $CDCl_3$)

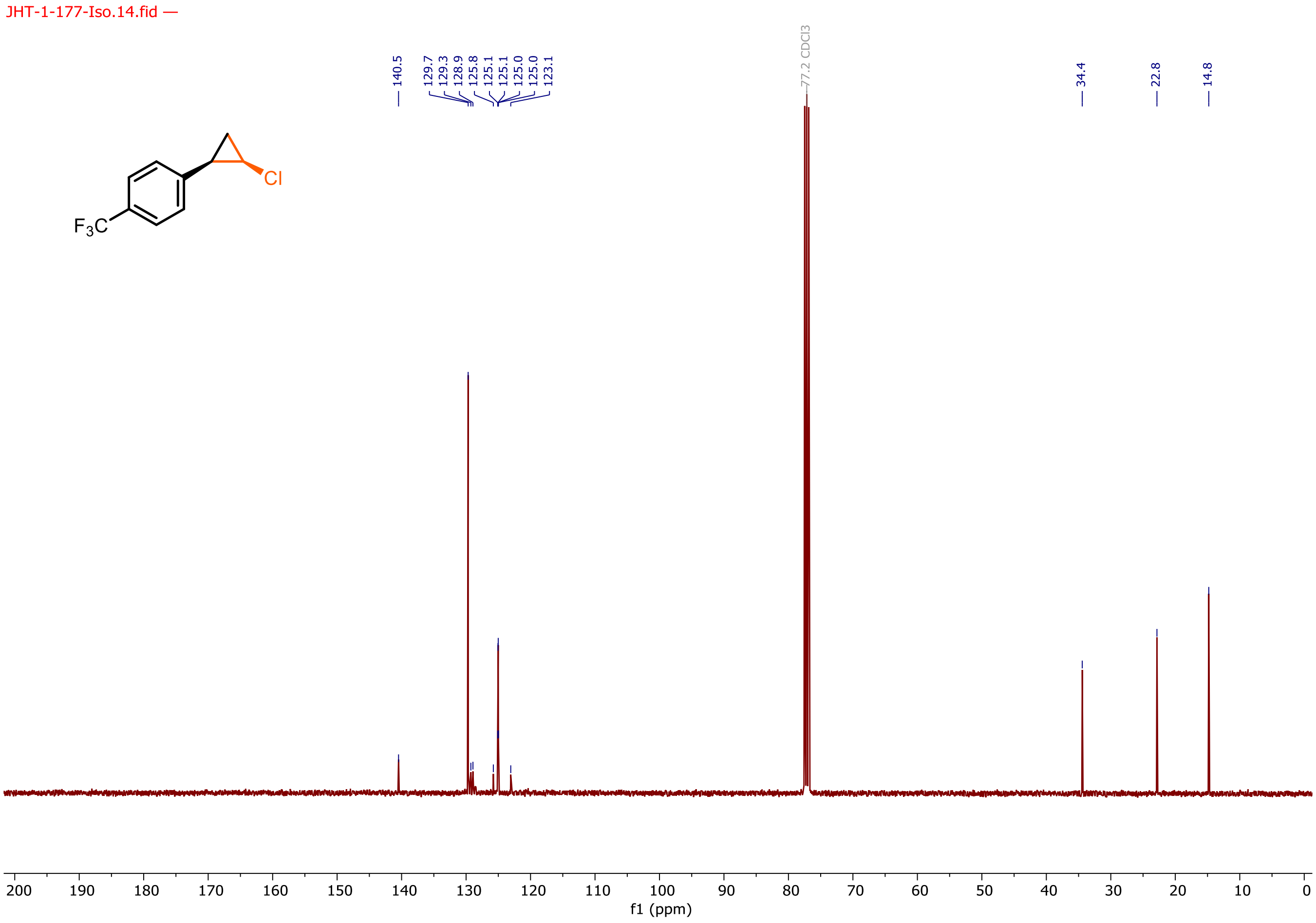

12: Crude ¹H NMR (400 MHz, $CDCl_3$)

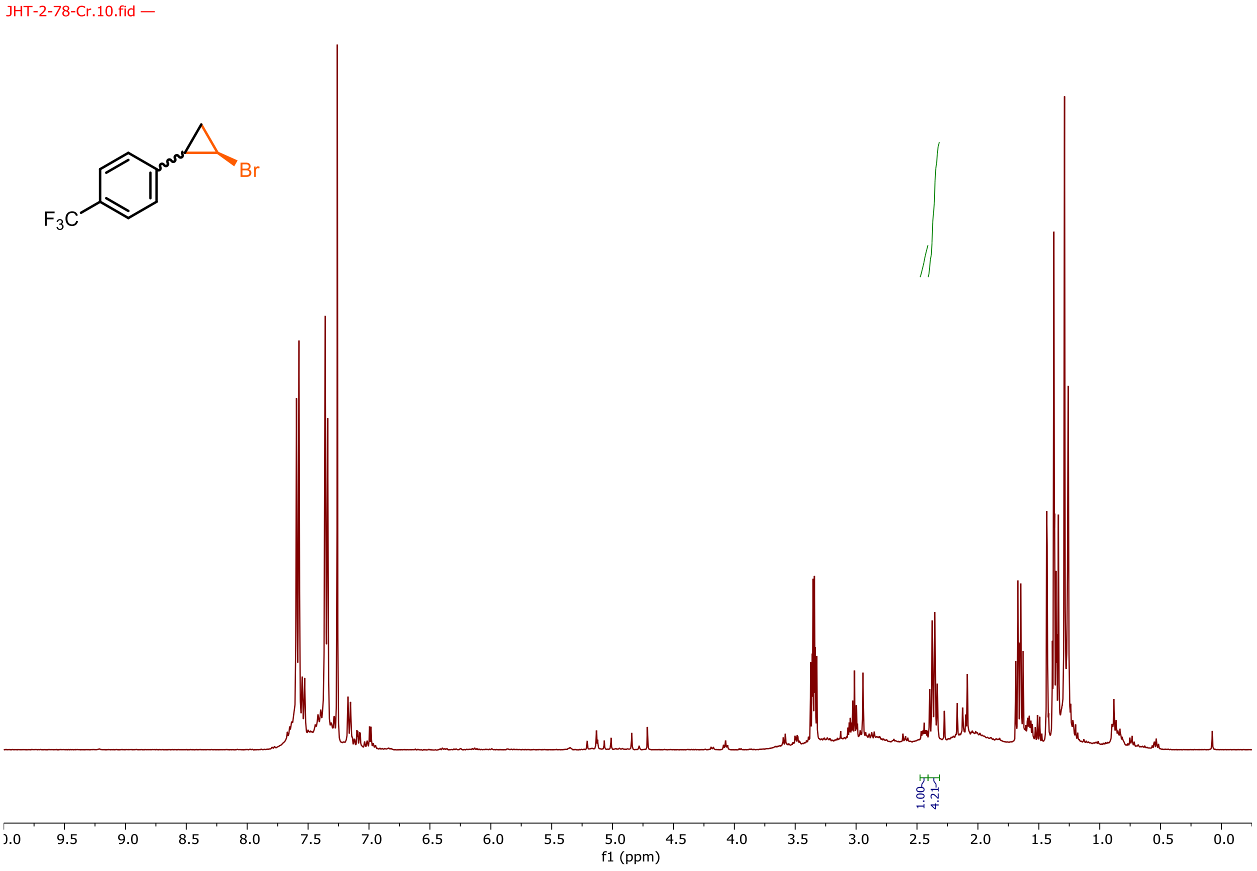

12: ^{1}H NMR (400 MHz, $CDCl_3$)

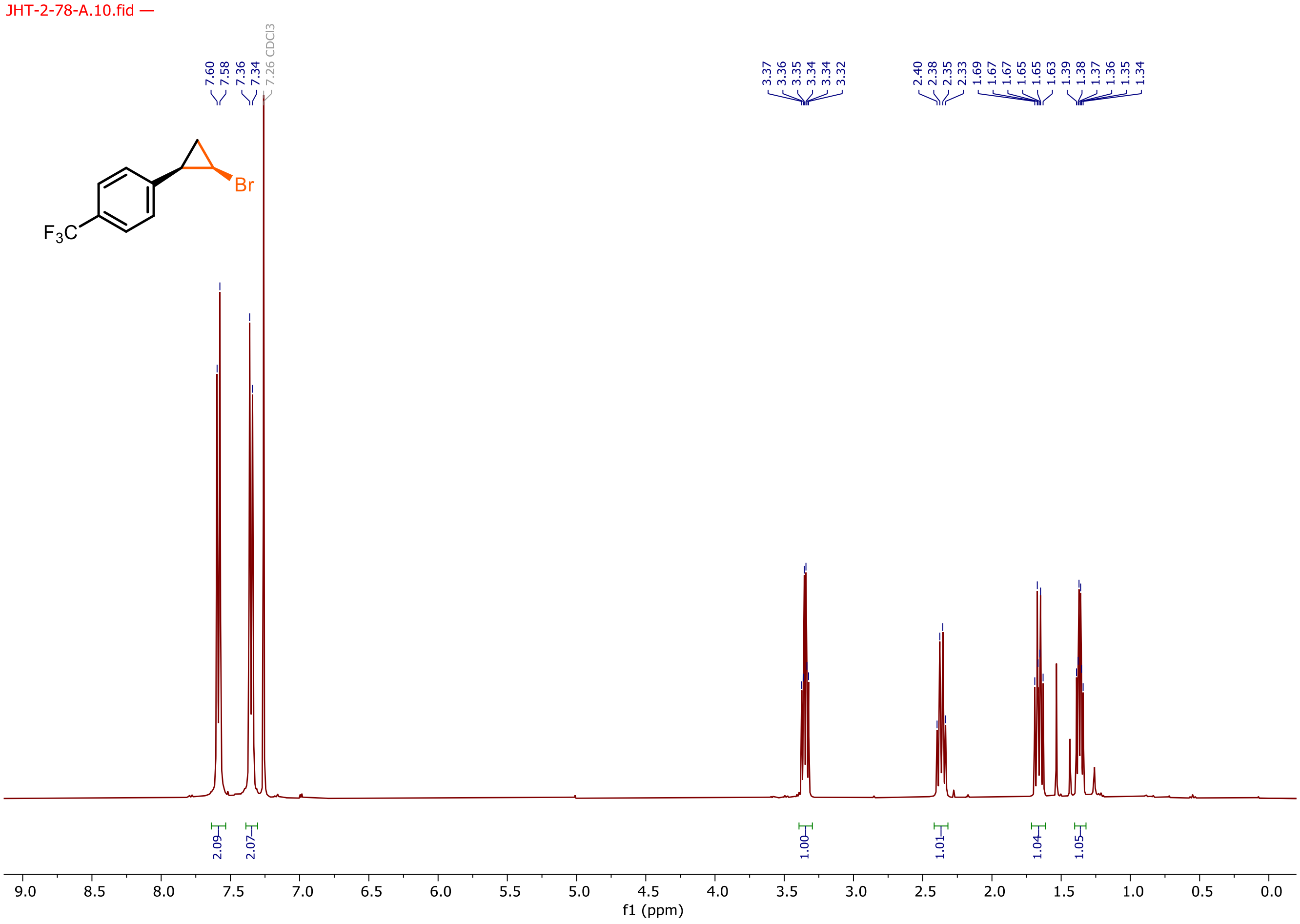

12: ^{13}C NMR (201 MHz, $CDCl_3$)

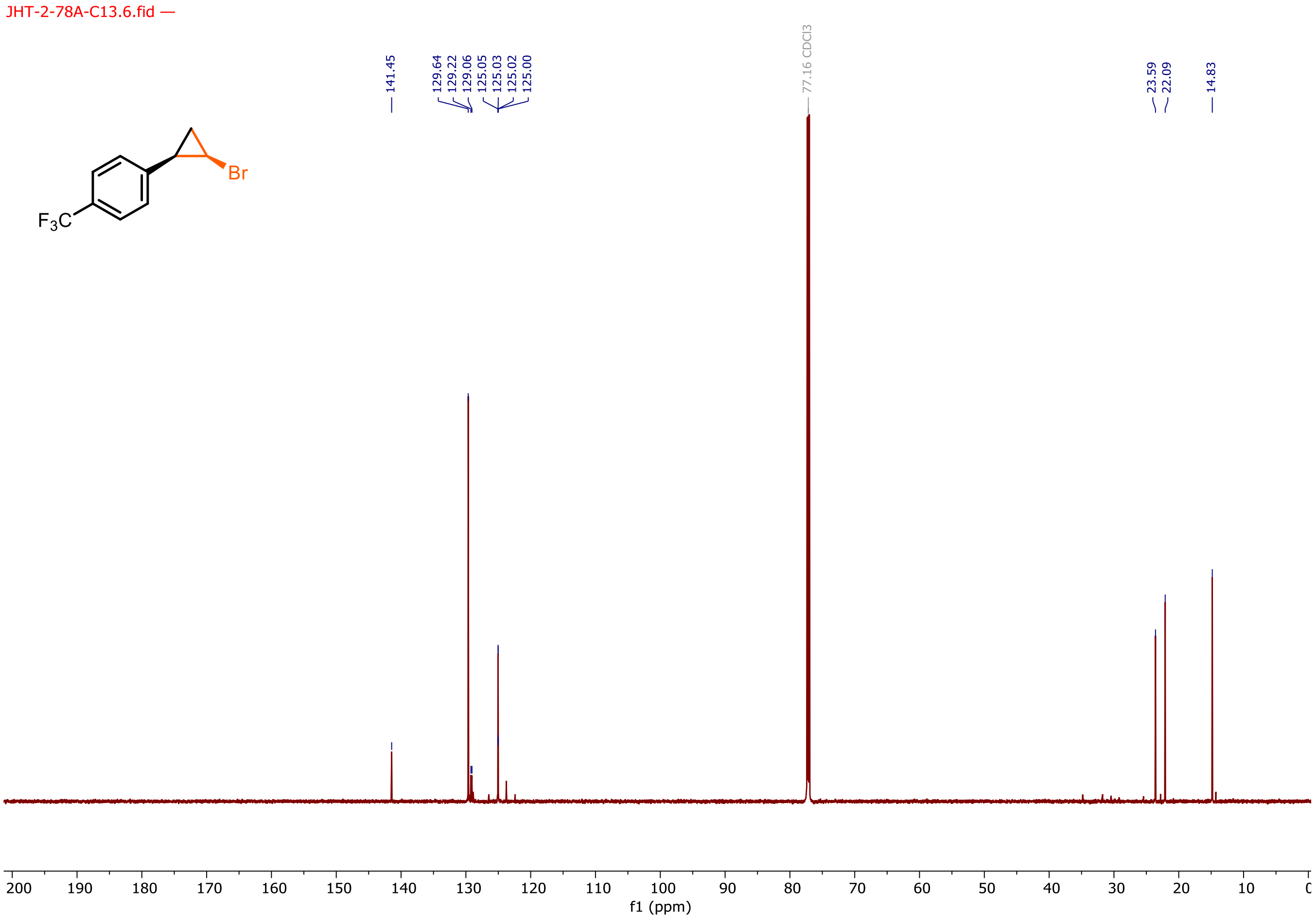

13: Crude ^{1}H NMR (400 MHz, $CDCl_3$)

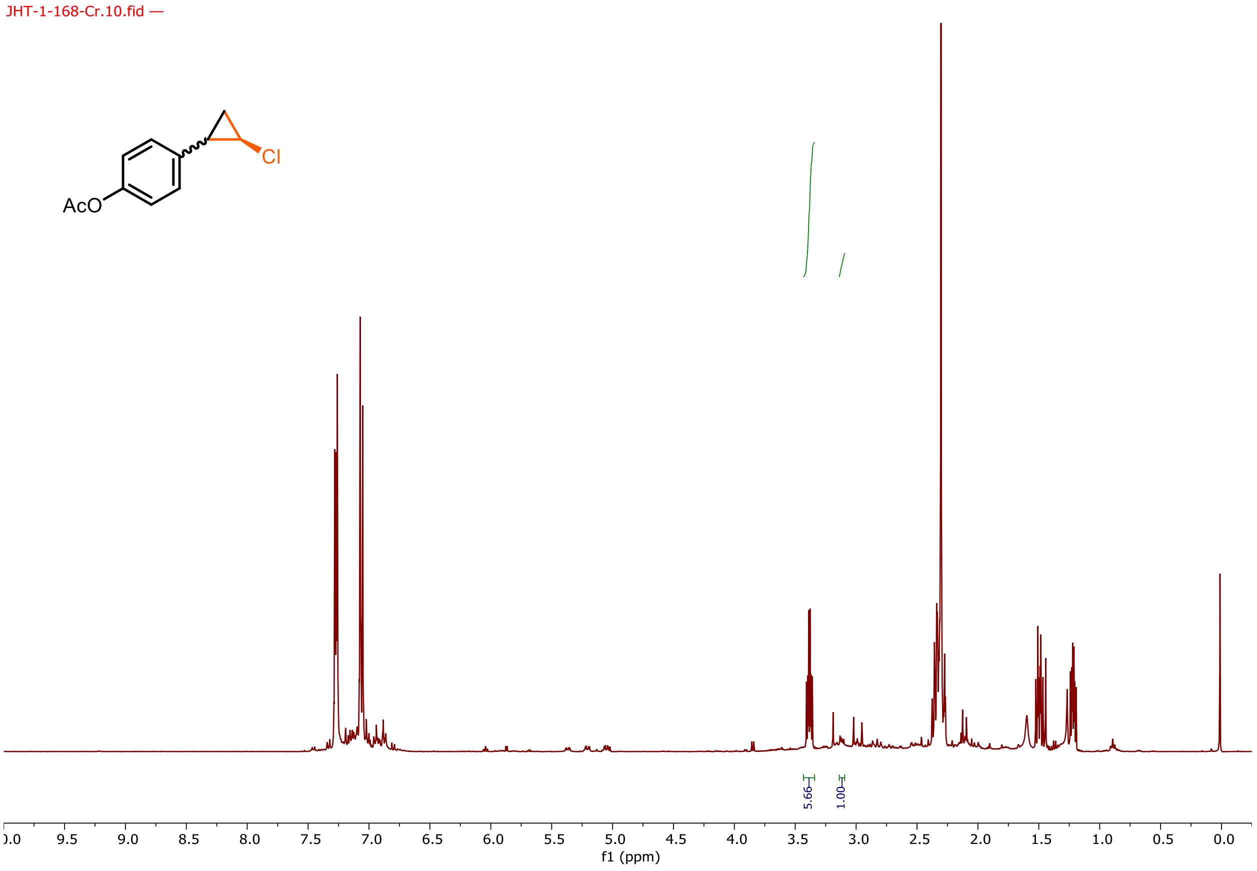

13: ^{1}H NMR (400 MHz, $CDCl_3$)

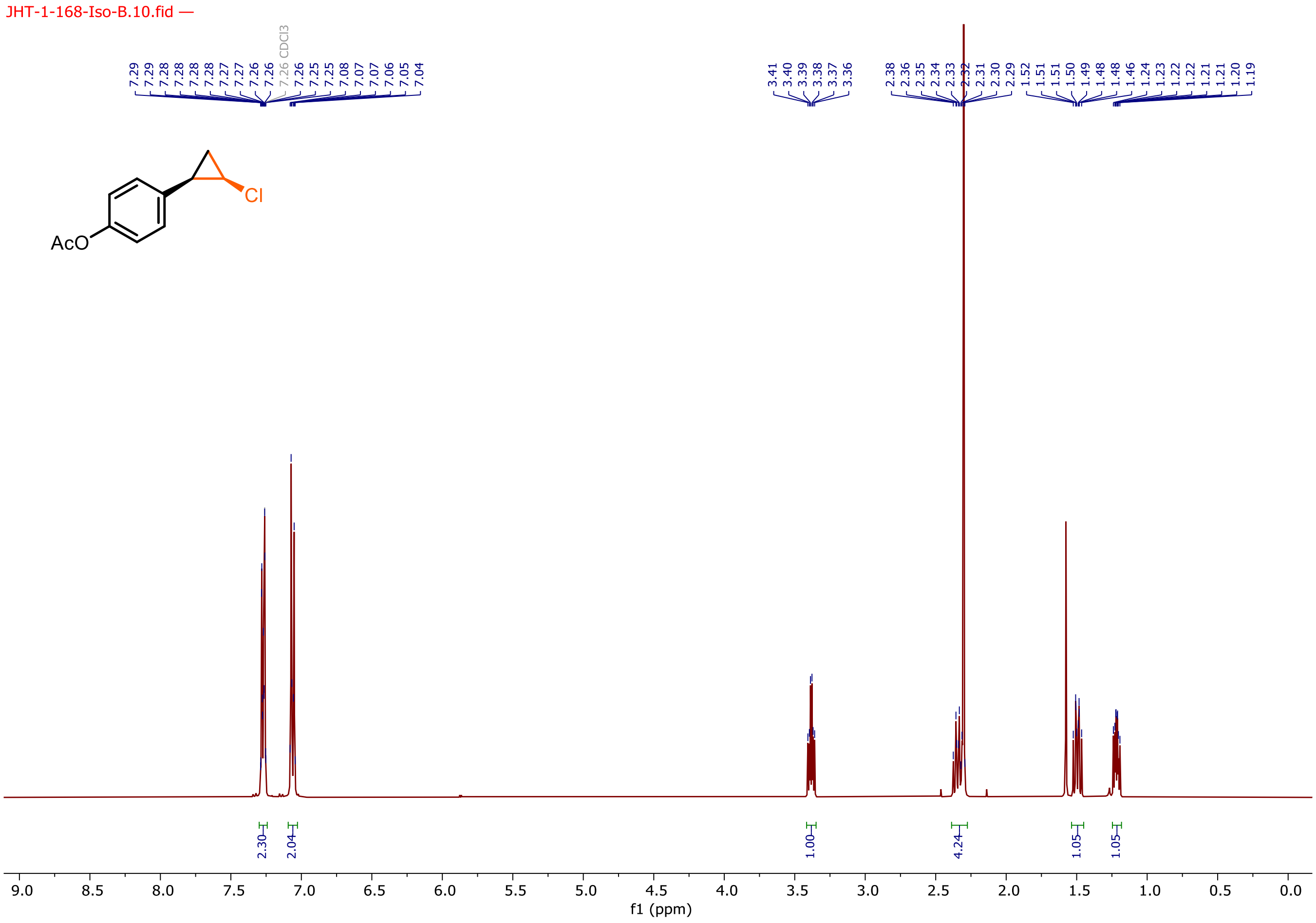

13: ^{13}C NMR (101 MHz, $CDCl_3$)

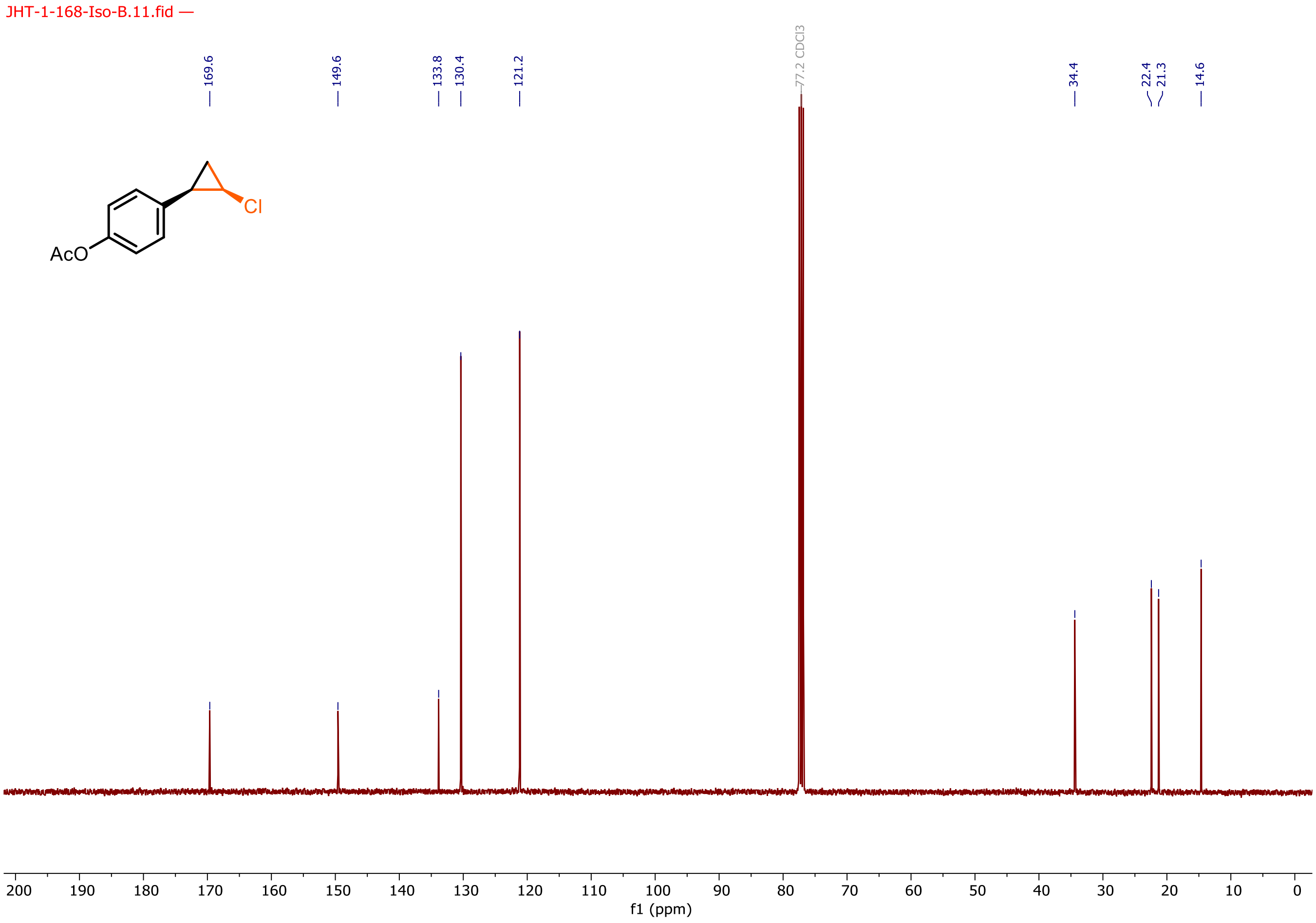

13: NOESY NMR (400 MHz, $CDCl_3$)

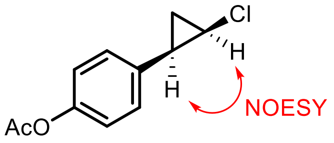

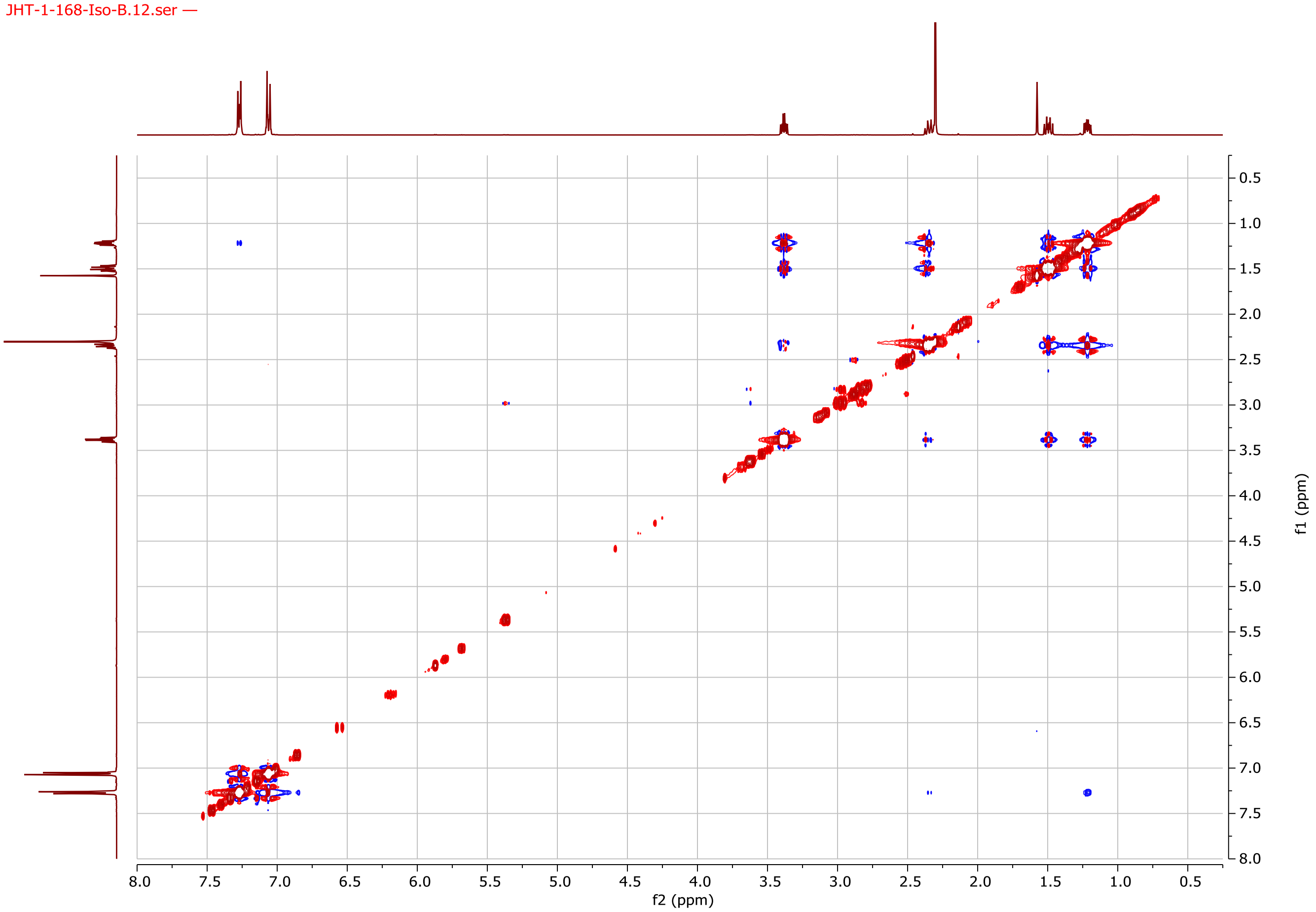

14: Crude ^{1}H NMR (400 MHz, $CDCl_3$)

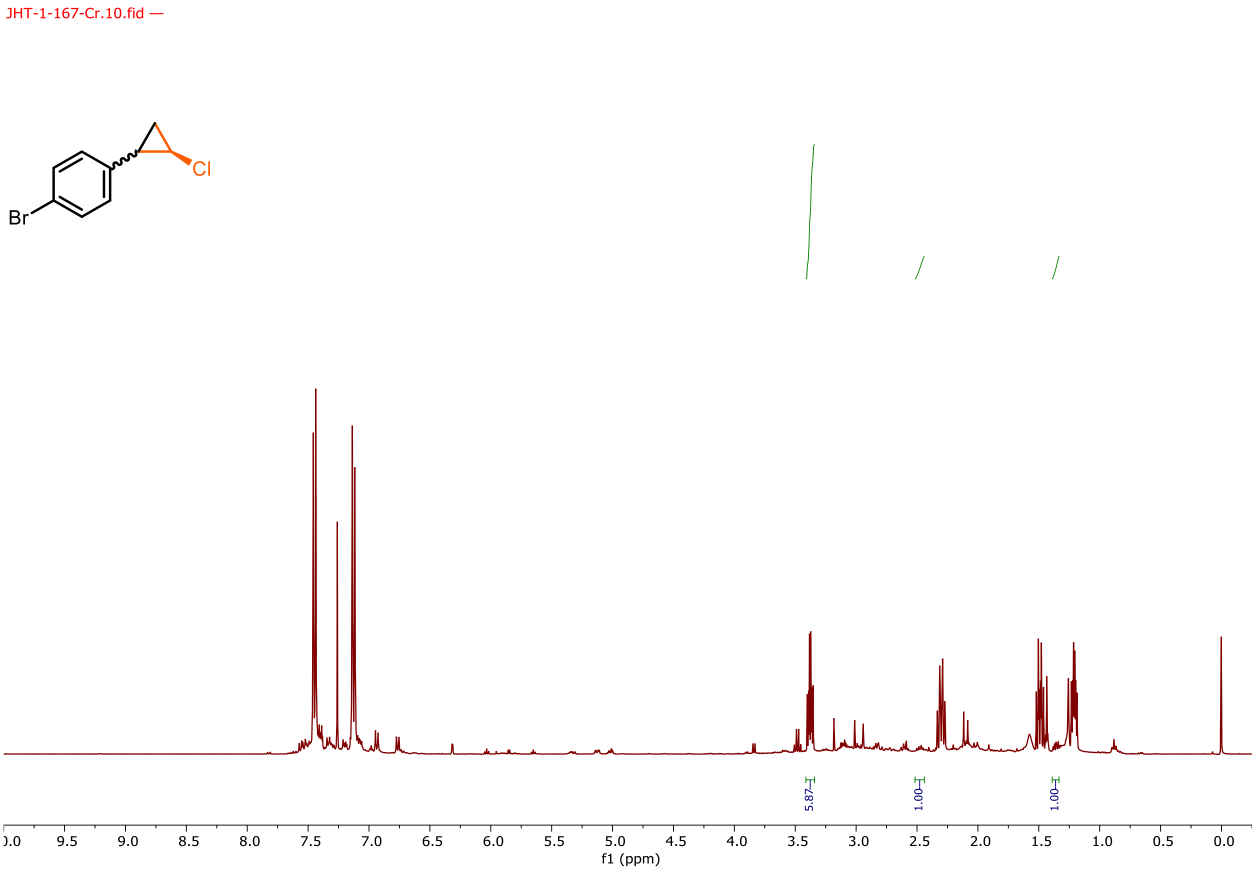

14: ^{1}H NMR (400 MHz, $CDCl_3$)

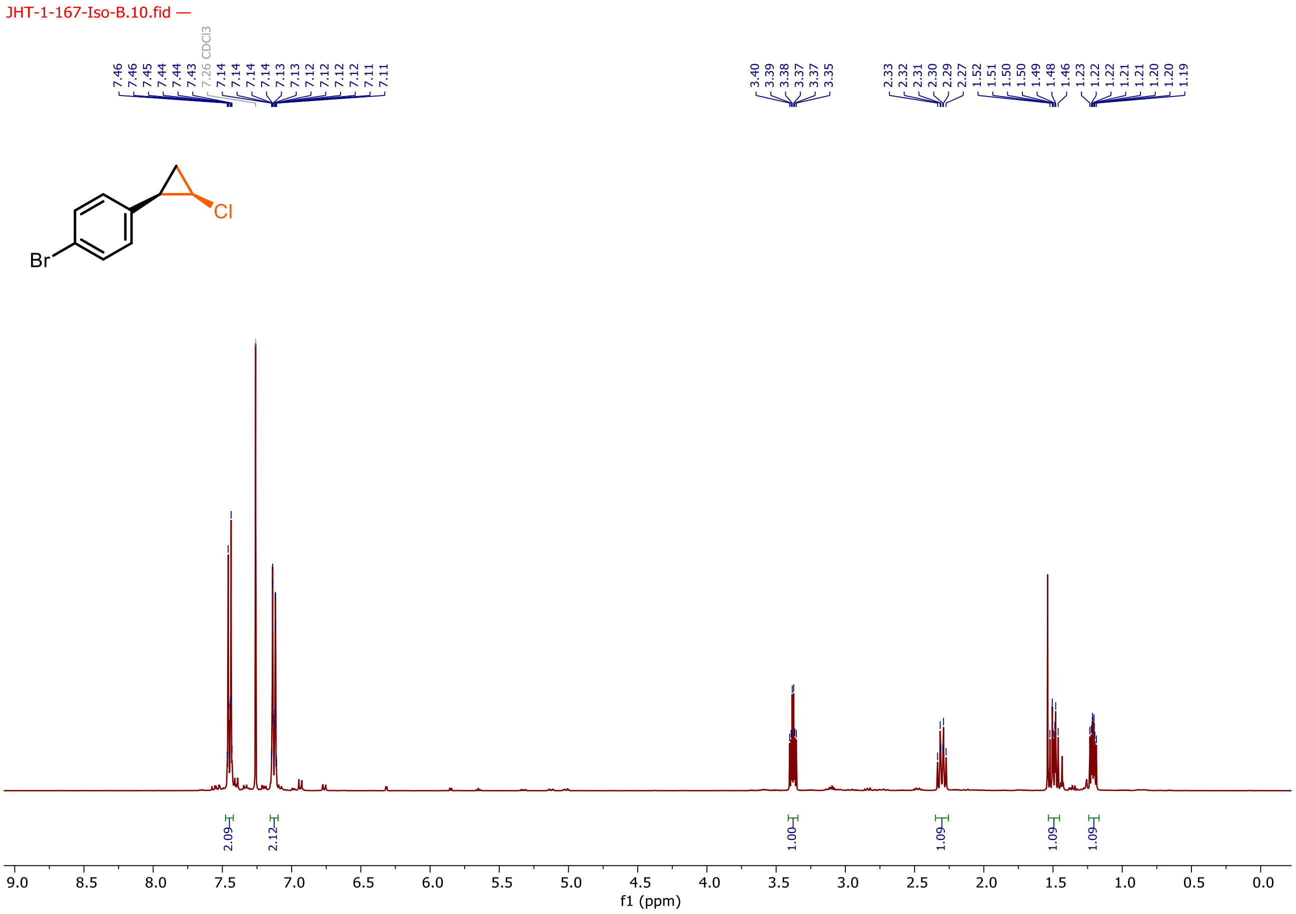

14: ^{13}C NMR (101 MHz, $CDCl_3$)

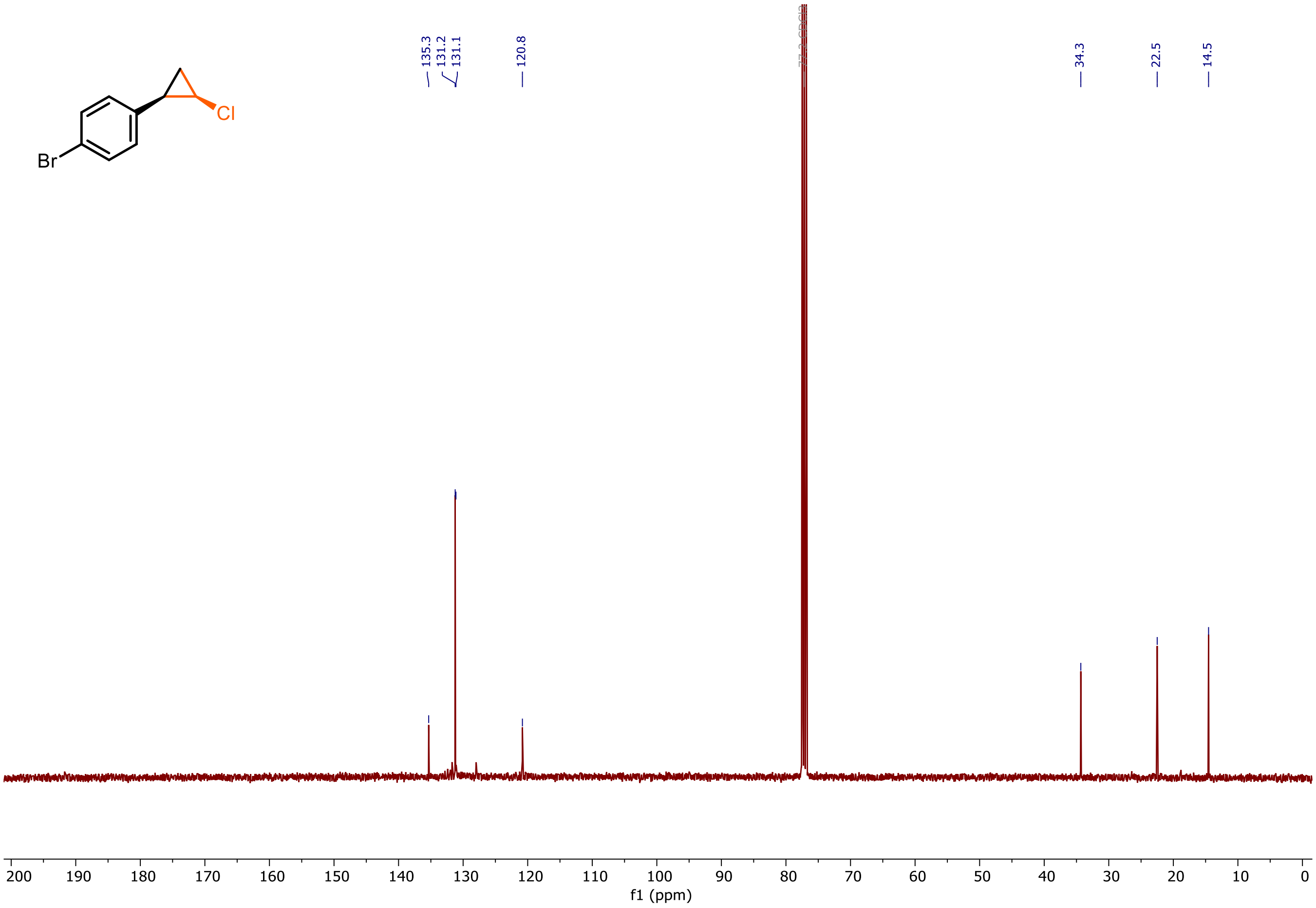

15: Crude ^{1}H NMR (400 MHz, $CDCl_3$)

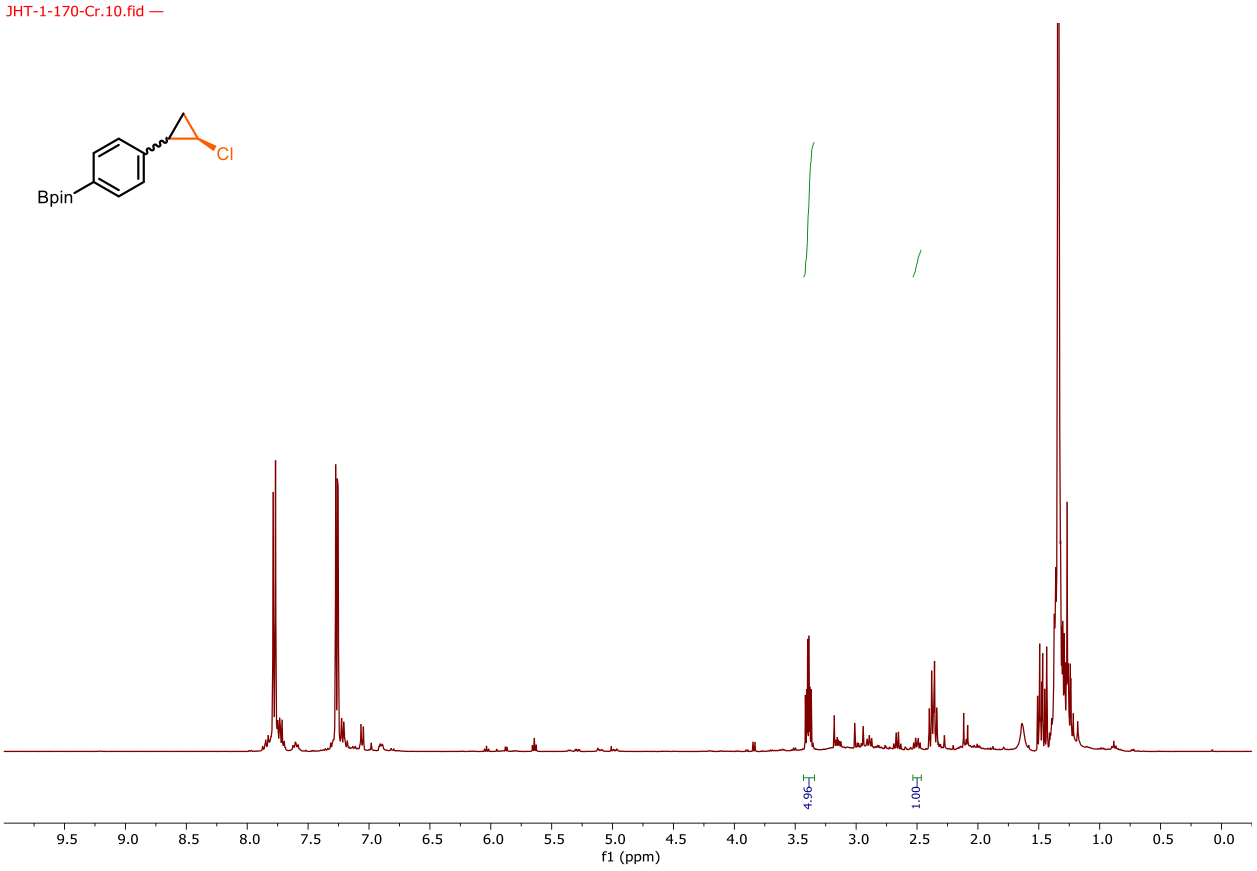

15: ¹H NMR (400 MHz, $CDCl_3$)

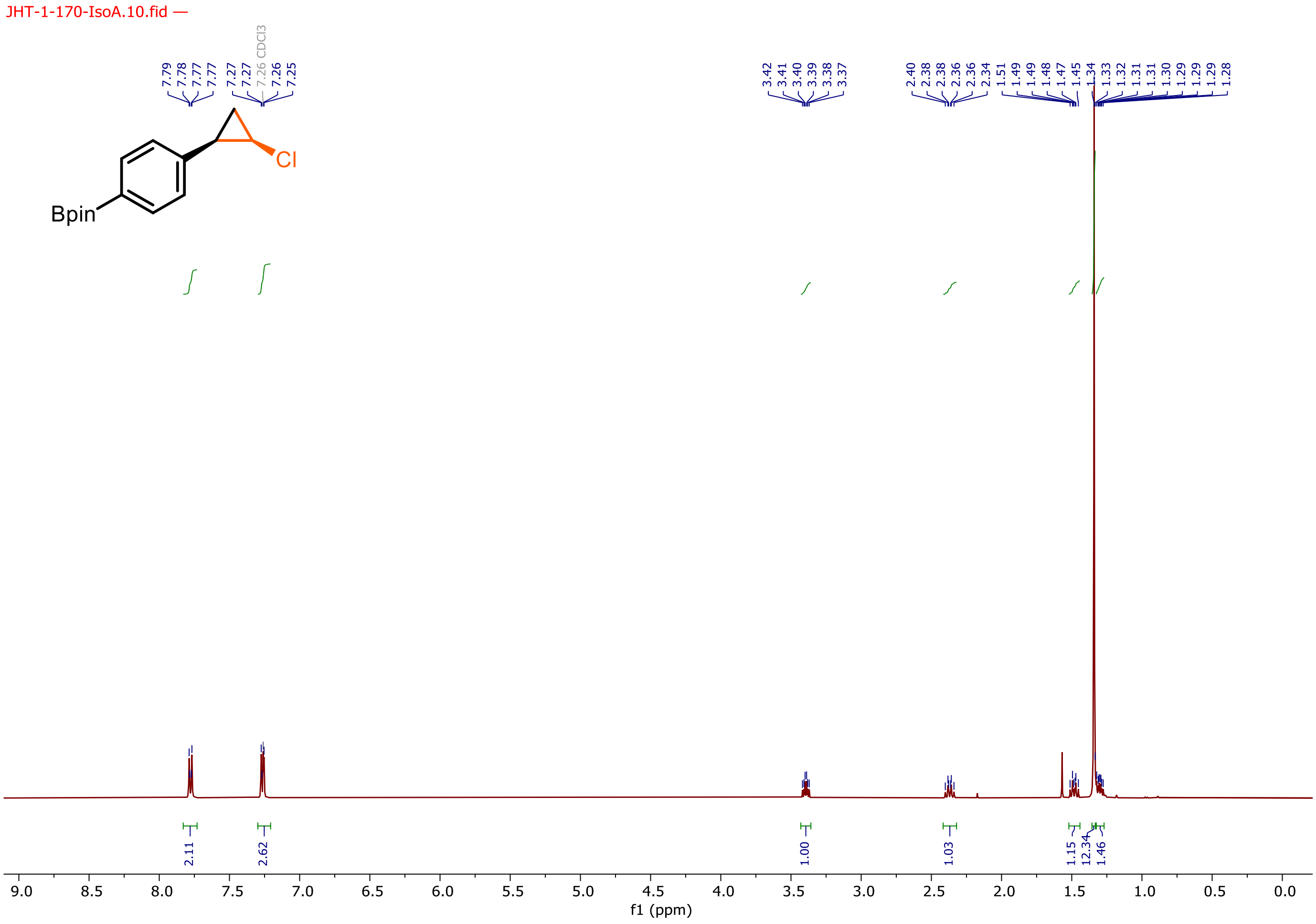

15: ^{13}C NMR (101 MHz, $CDCl_3$)

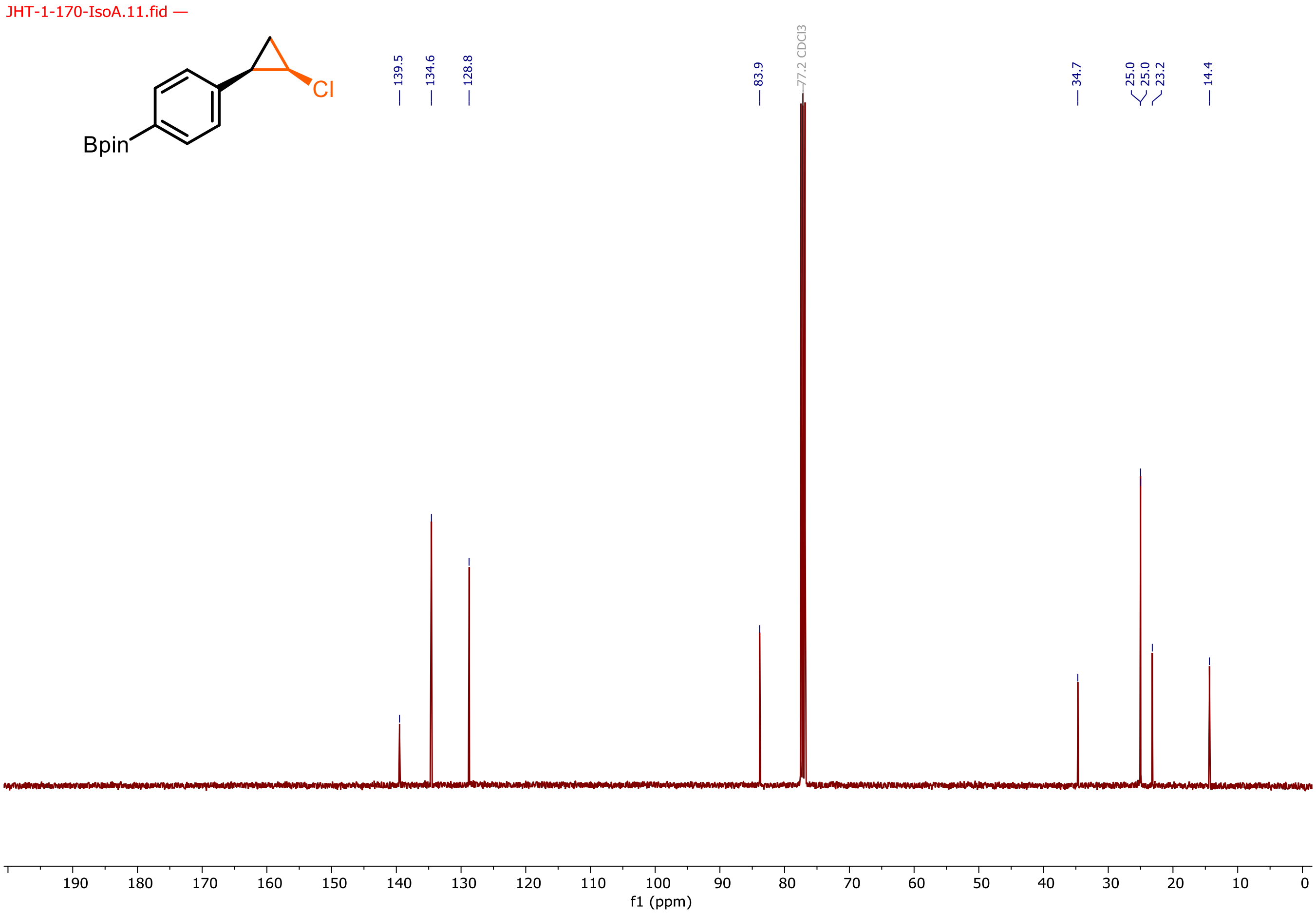

15: NOESY NMR (400 MHz, $CDCl_3$)

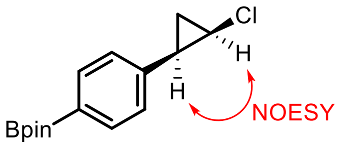

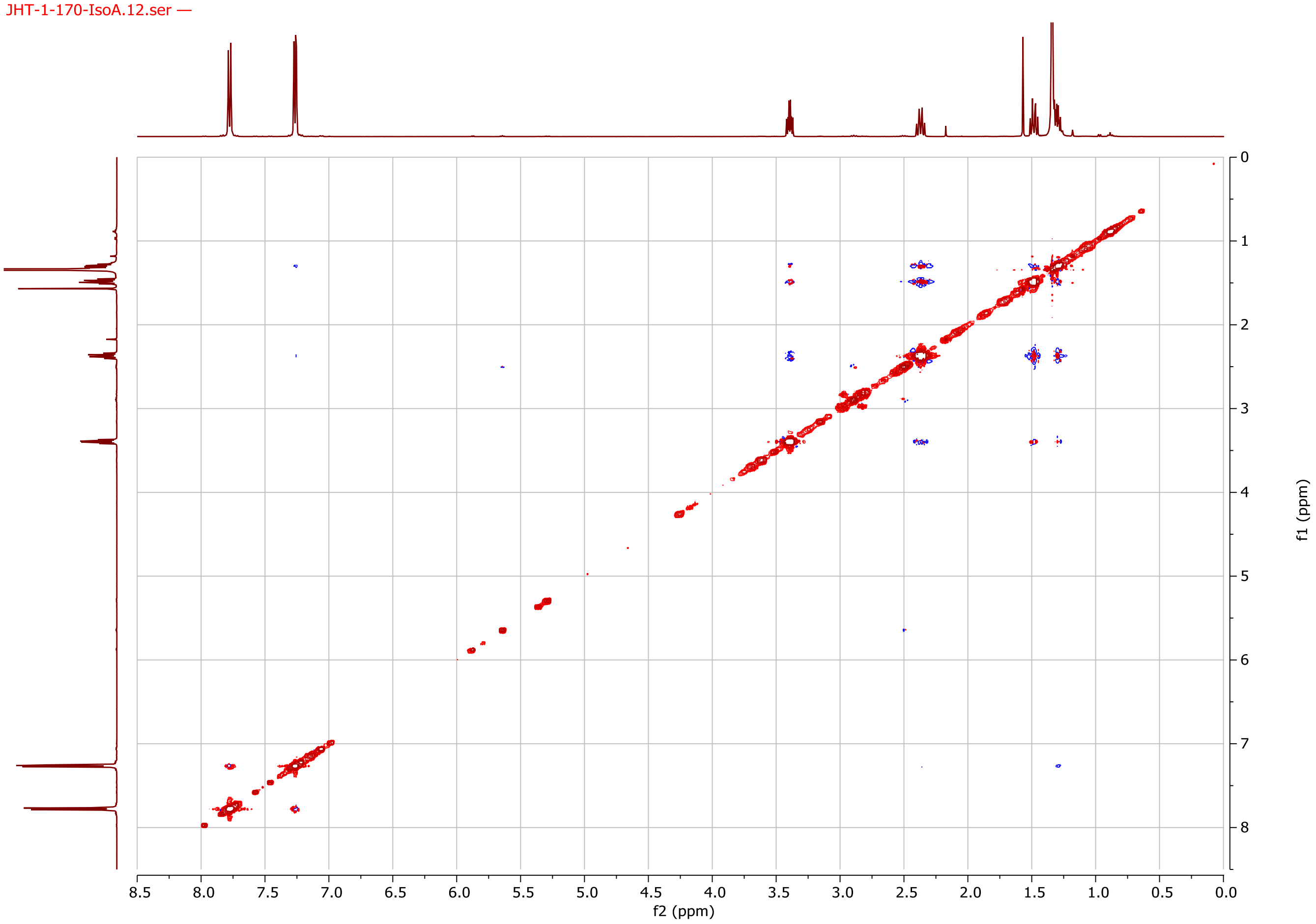

16: Crude ^{1}H NMR (400 MHz, $CDCl_3$)

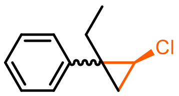

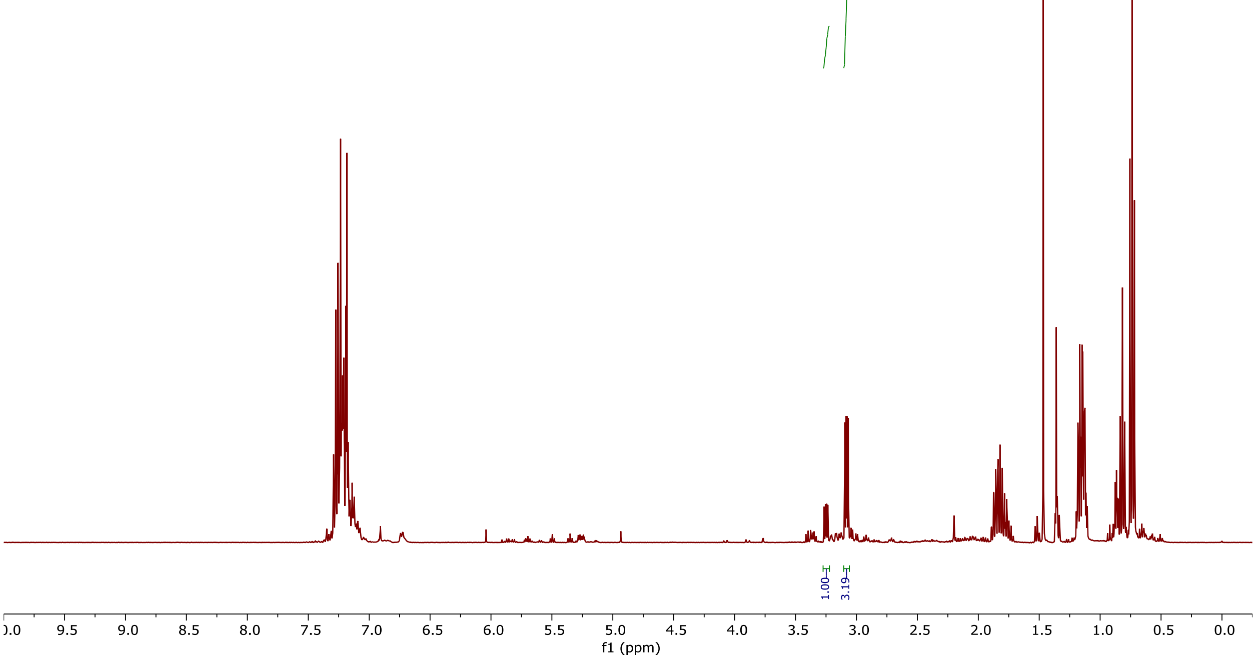

16 (*cis*): ^{1}H NMR (800 MHz, $CDCl_3$)

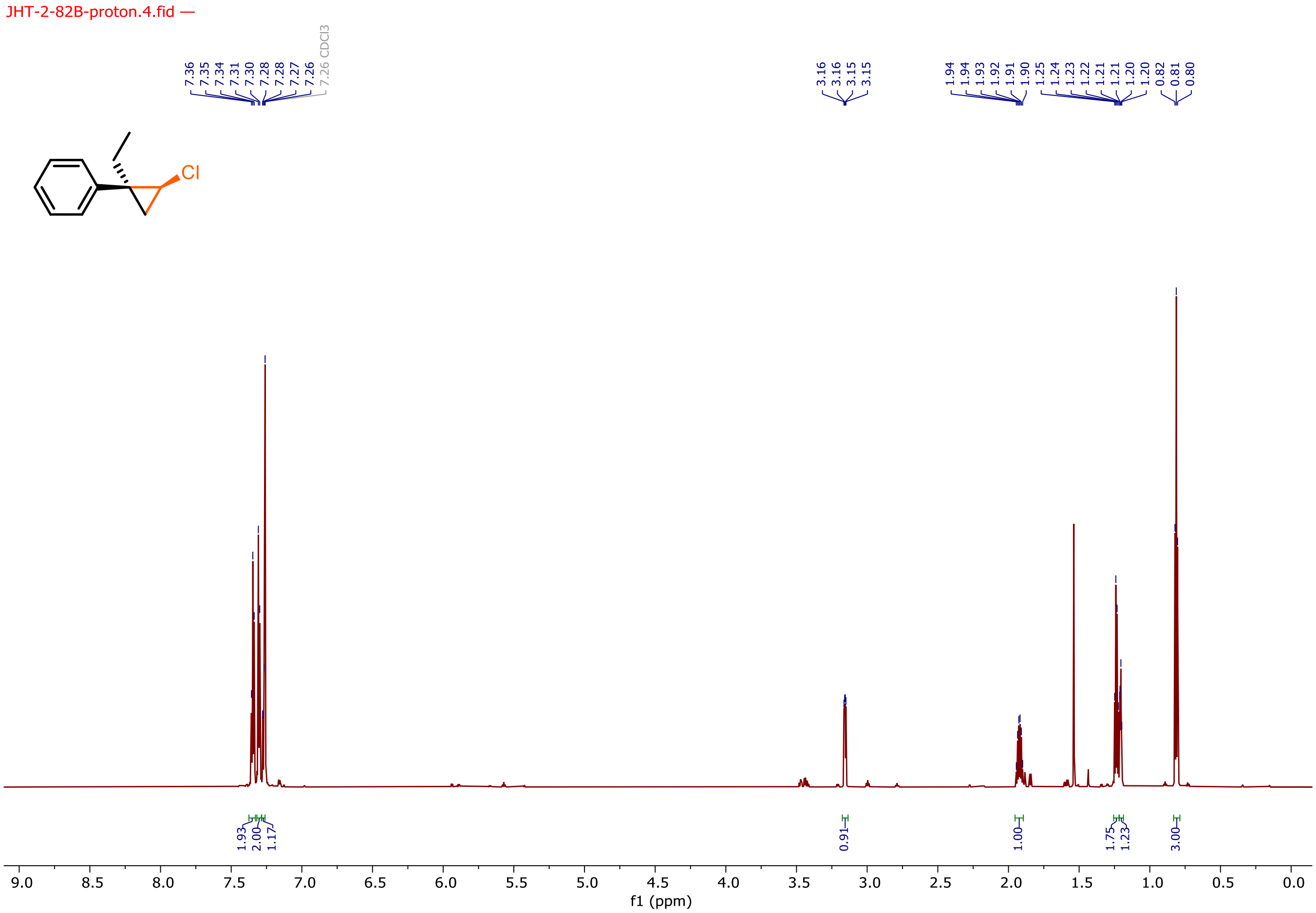

16 (*cis*): ^{13}C NMR (201 MHz, $CDCl_3$)

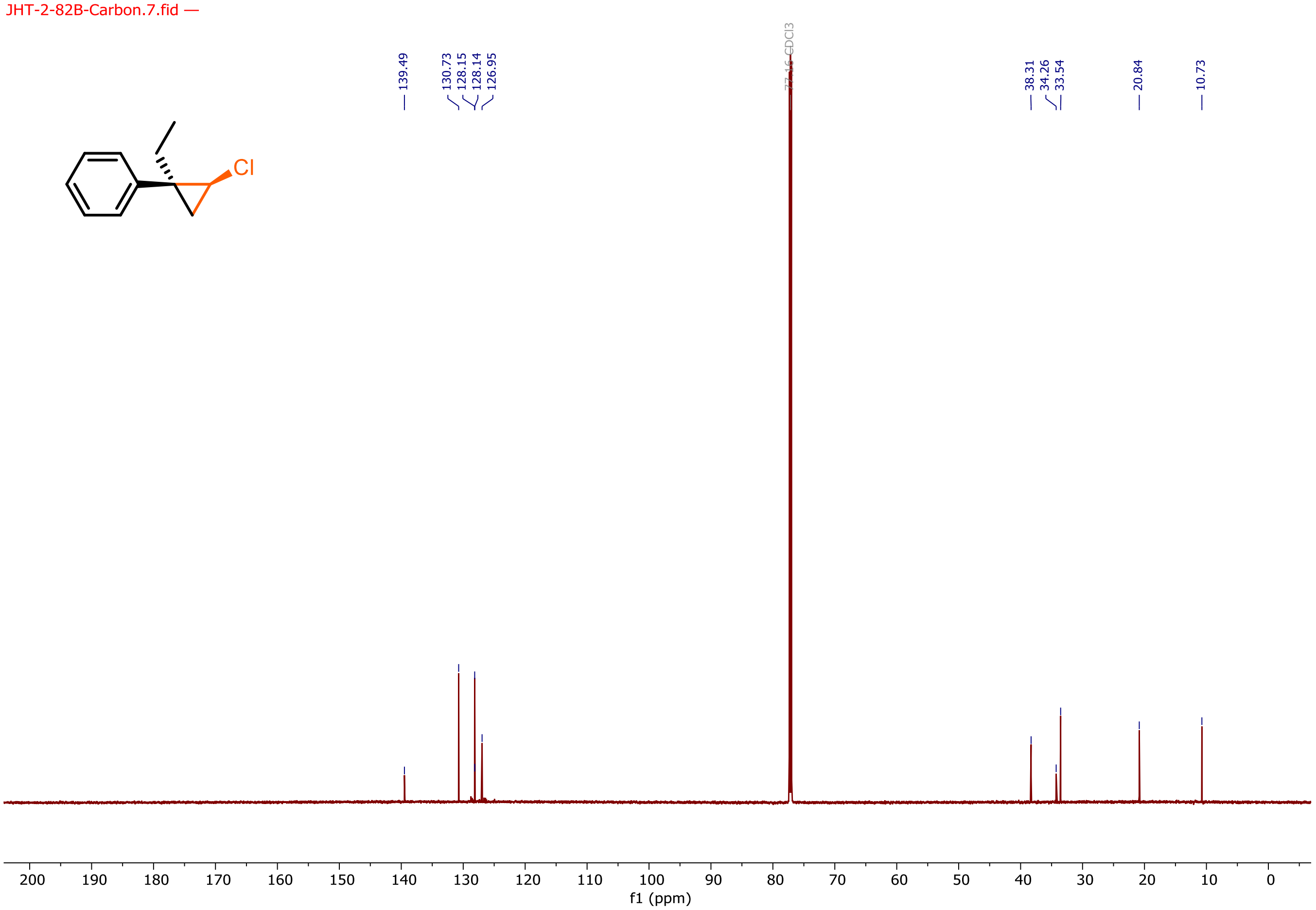

16 *(cis)*: NOESY NMR (800 MHz, $CDCl_3$)

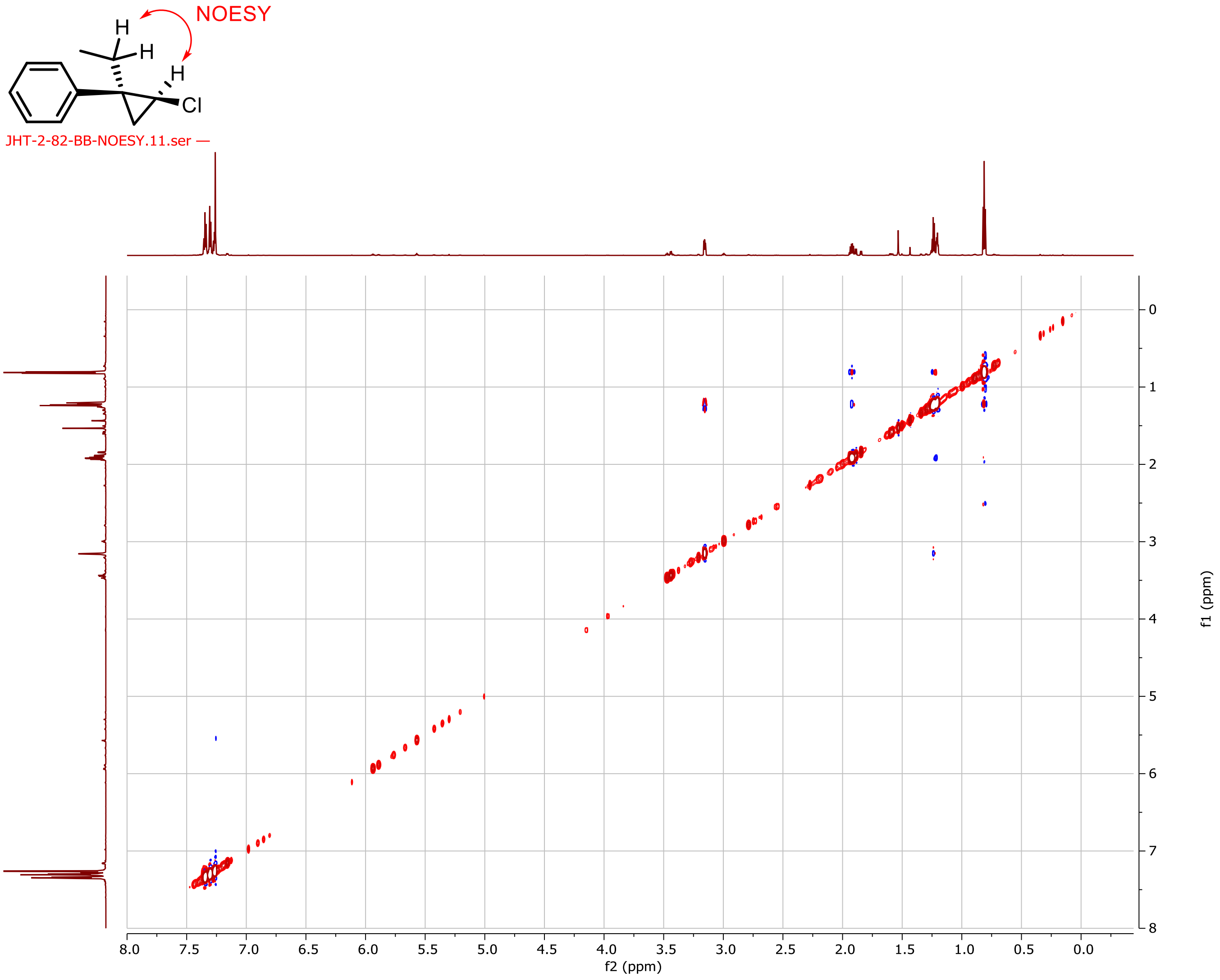

16 (*trans*): ^{1}H NMR (800 MHz, $CDCl_3$)

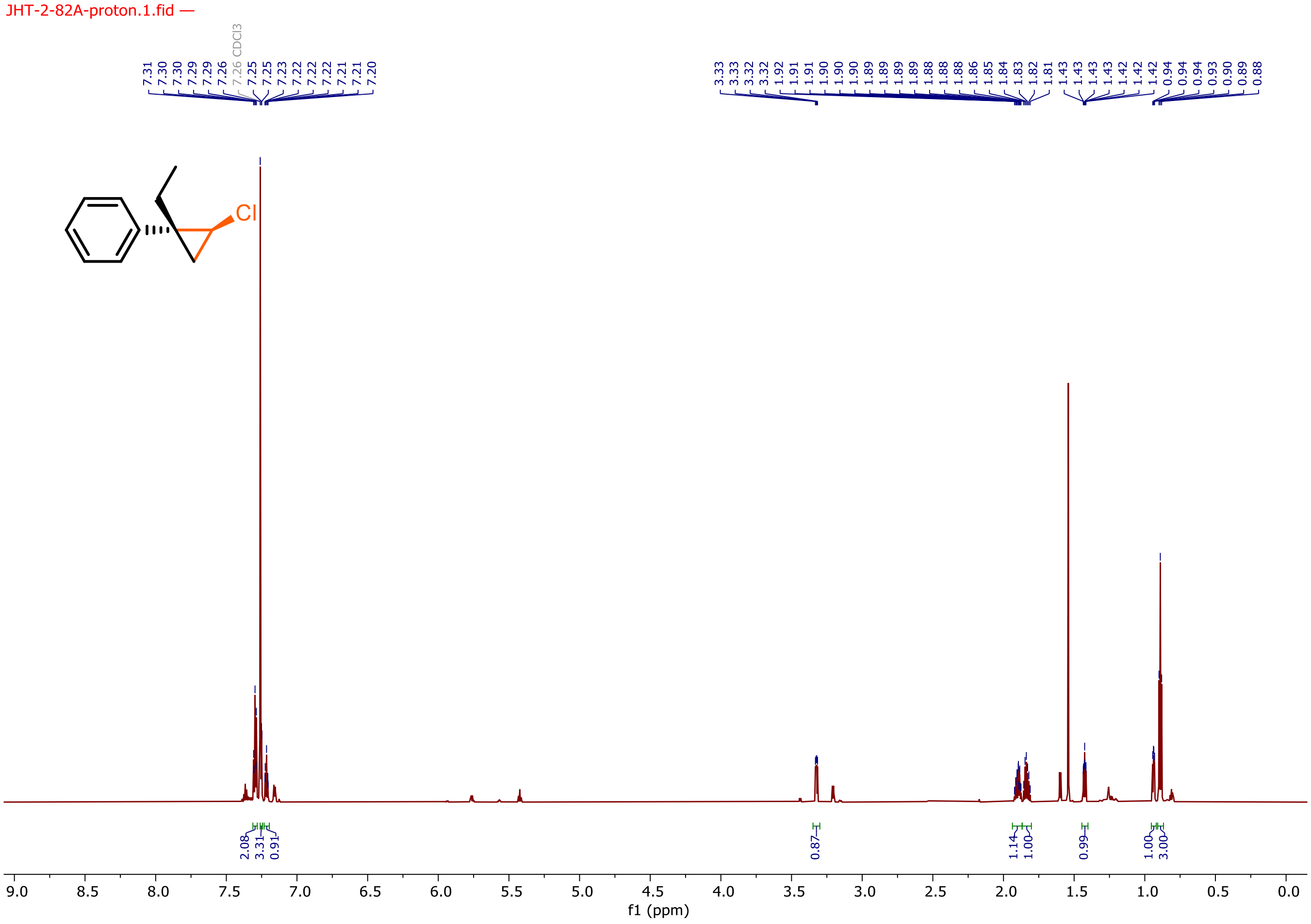

16 (*trans*): ^{13}C NMR (201 MHz, $CDCl_3$)

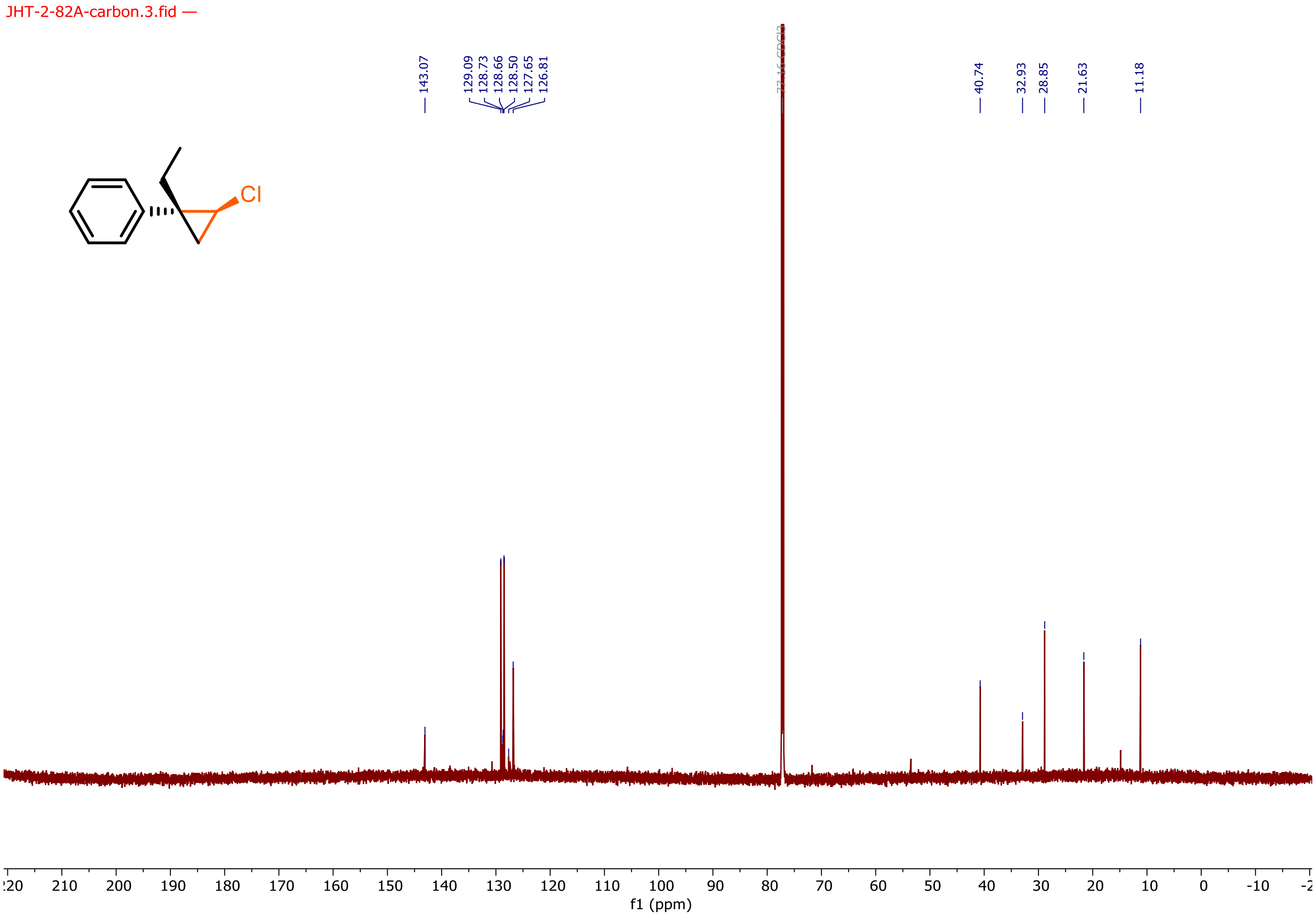

17: Crude ^{1}H NMR (400 MHz, $CDCl_3$)

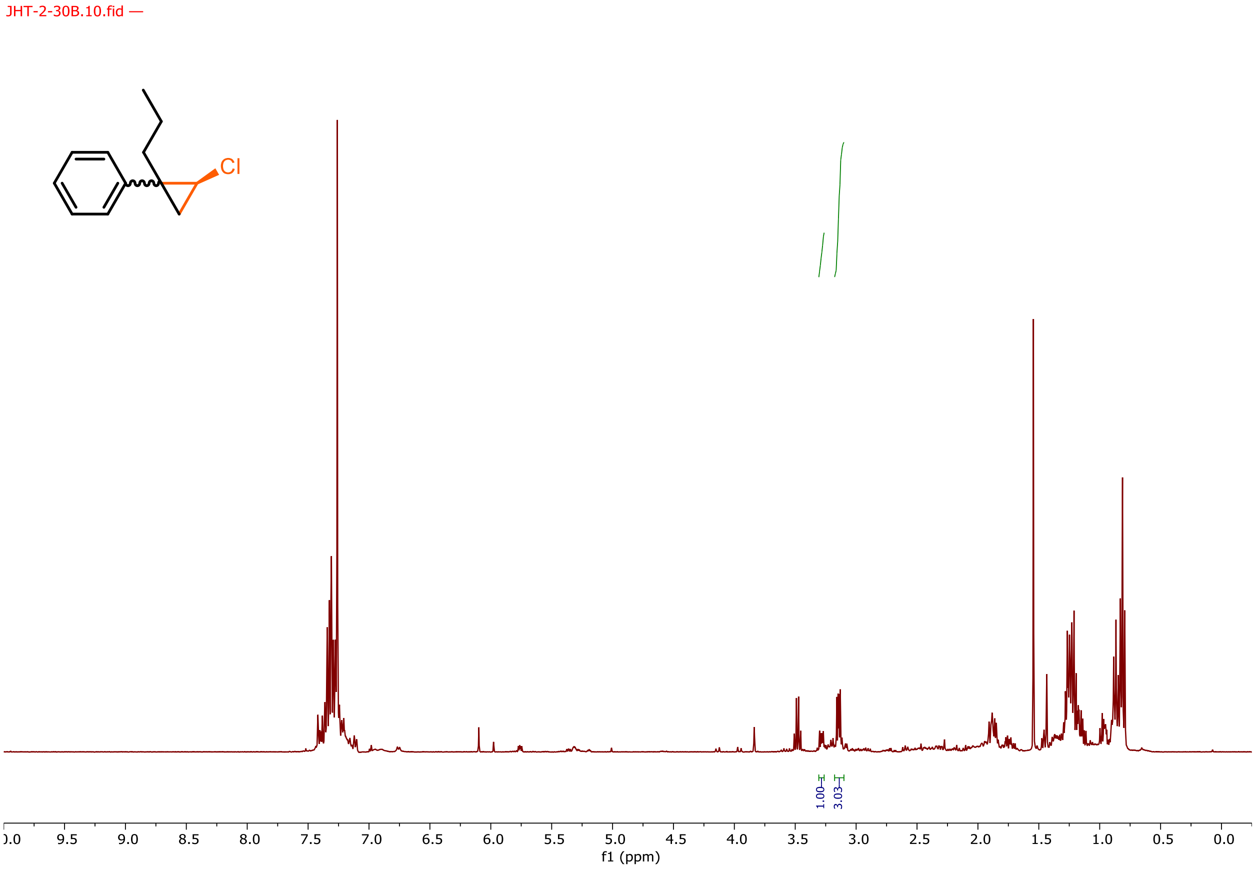

17 (*cis*): ^{1}H NMR (800 MHz, $CDCl_3$)

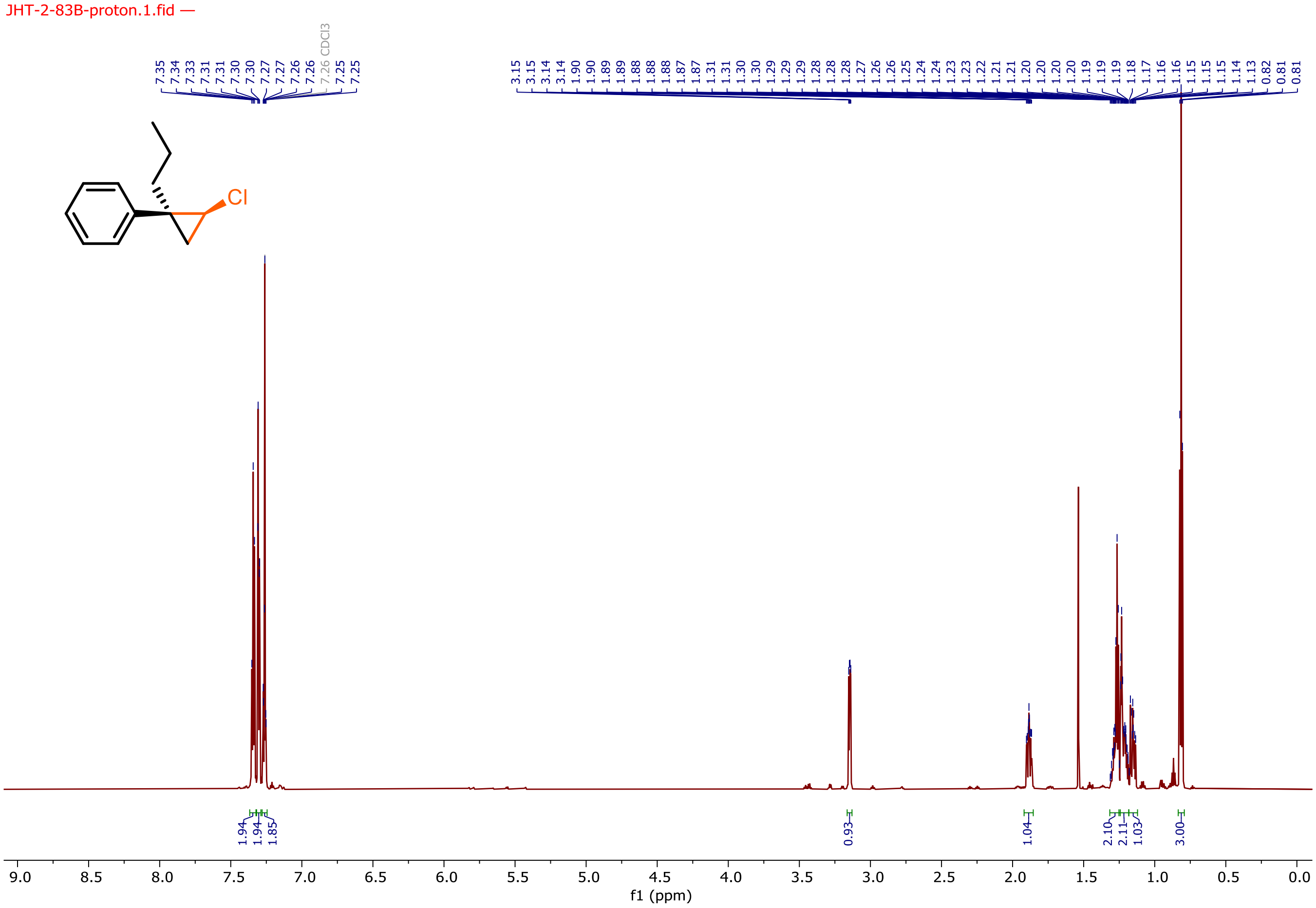

17 (*cis*): ^{13}C NMR (201 MHz, $CDCl_3$)

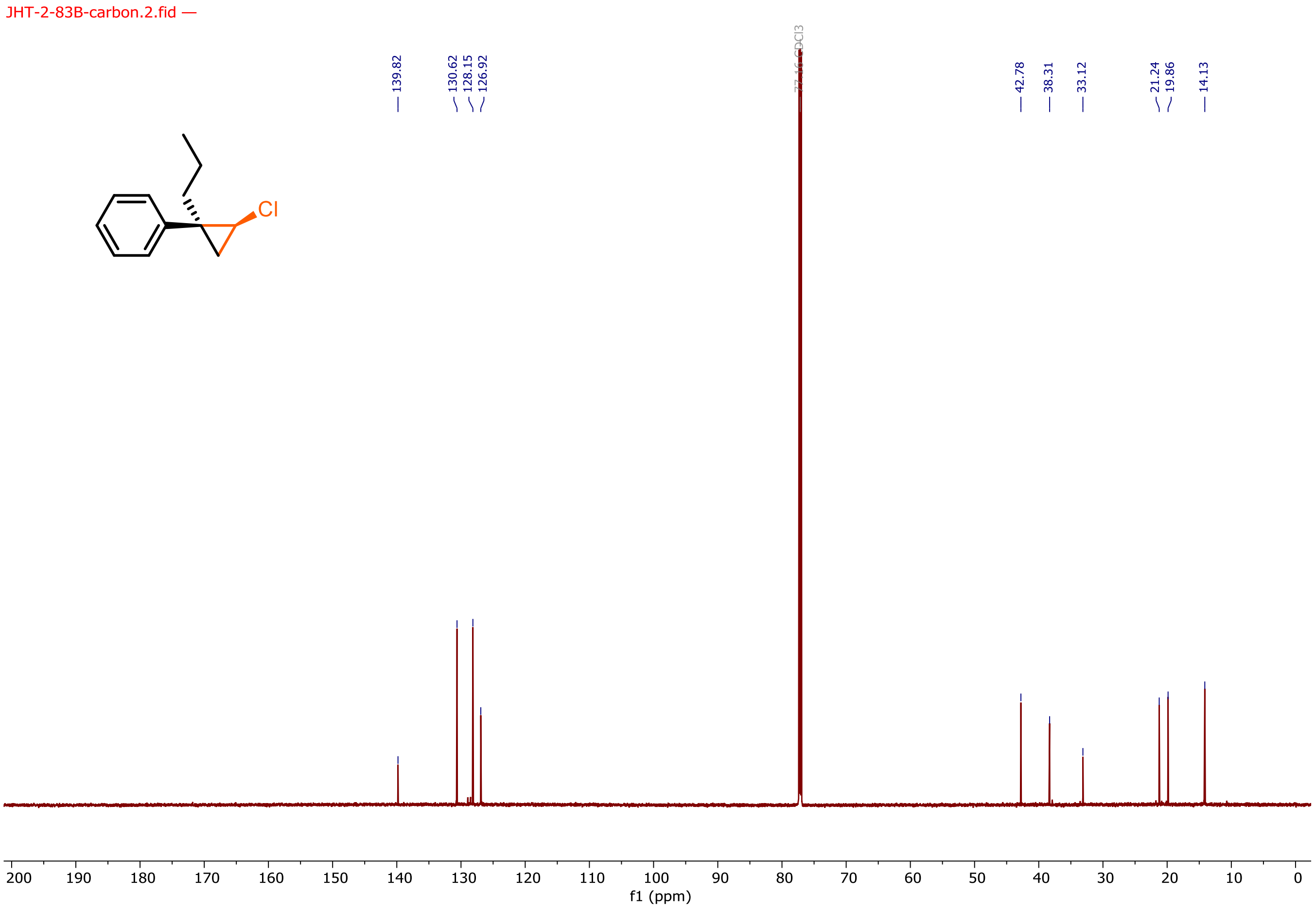

17 (*cis*): NOESY (800 MHz, $CDCl_3$)

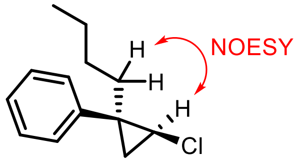

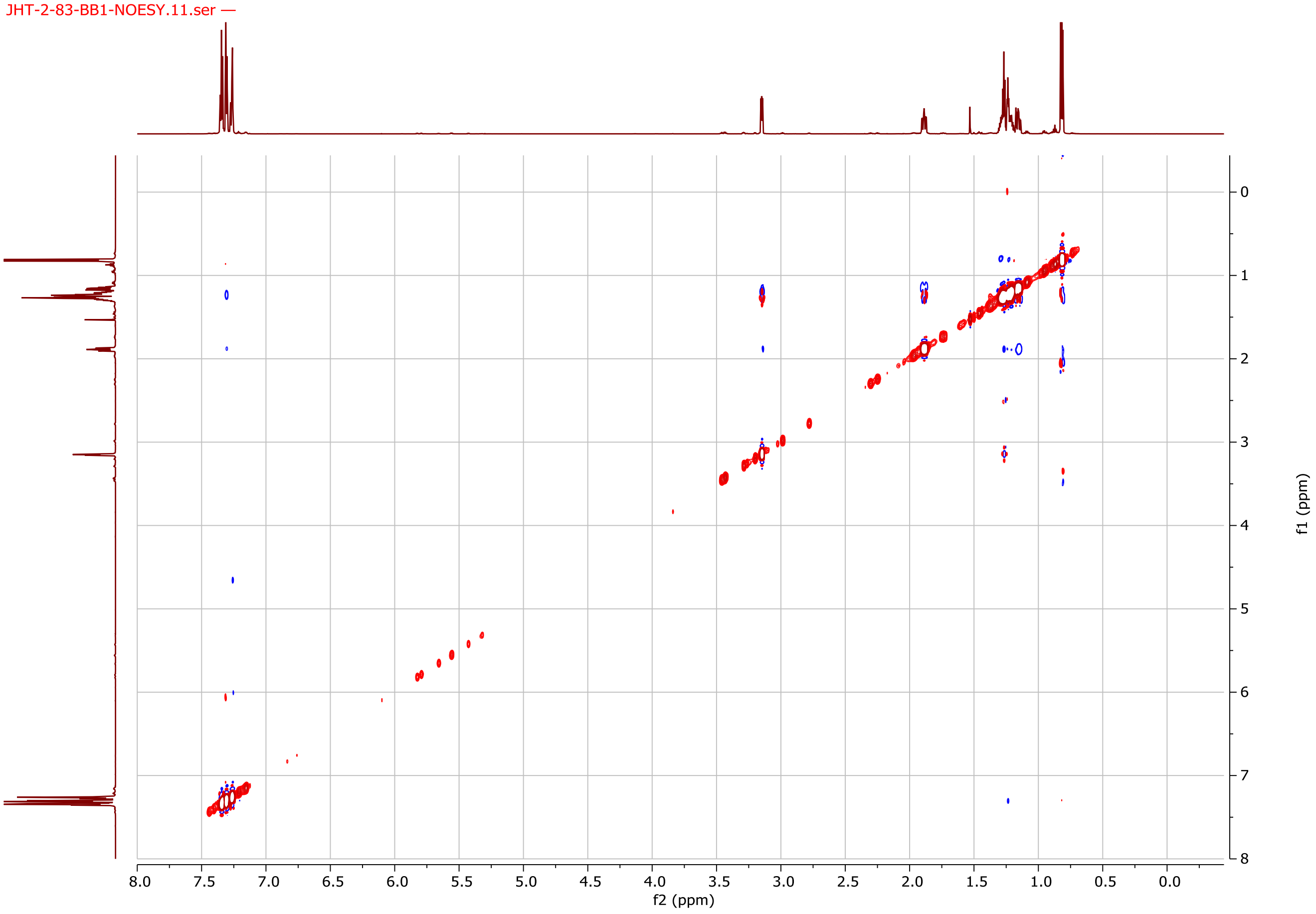

17 (*trans*): ^{1}H NMR (800 MHz, $CDCl_3$)

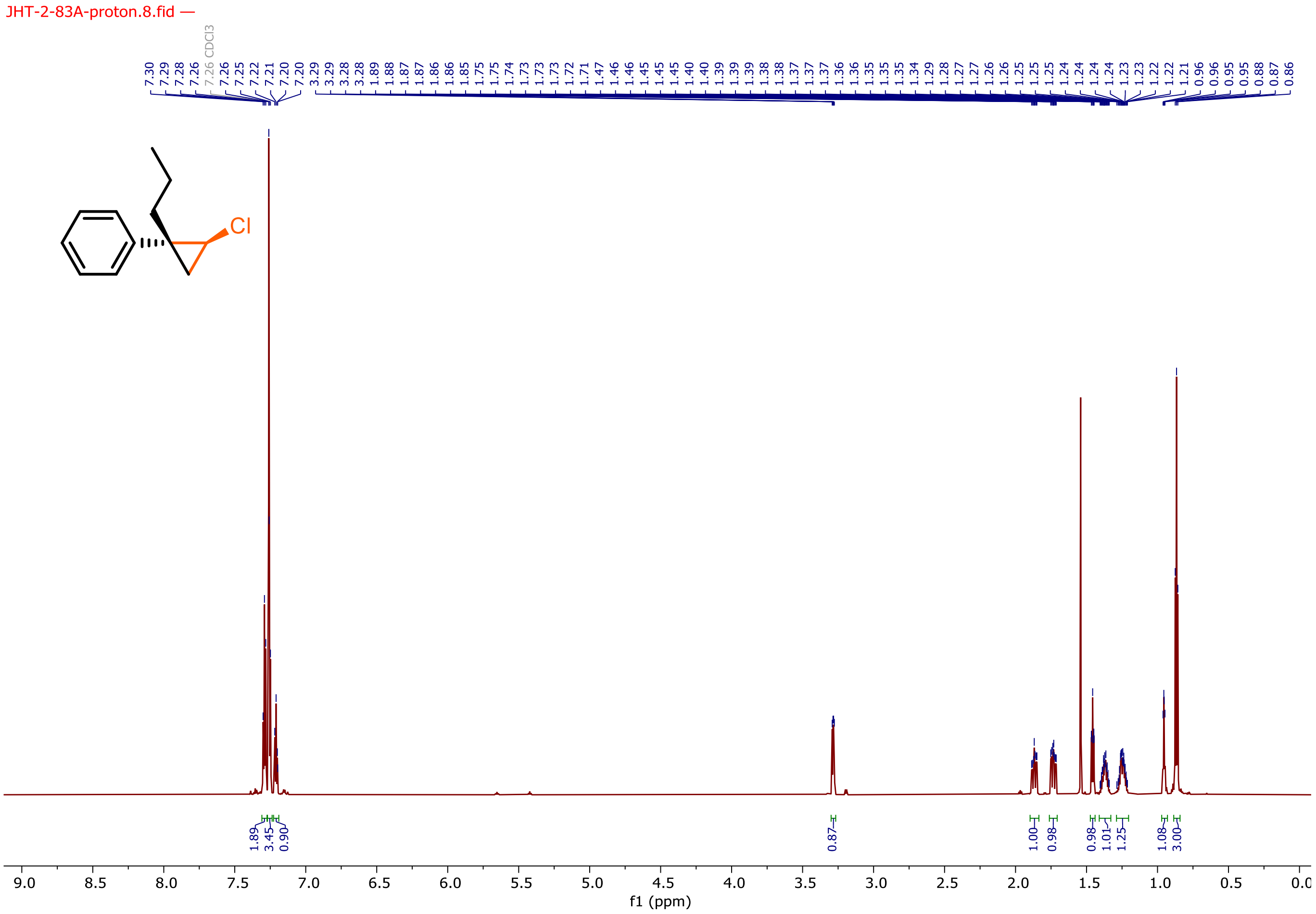

17 (*trans*): ^{13}C NMR (201 MHz, $CDCl_3$)

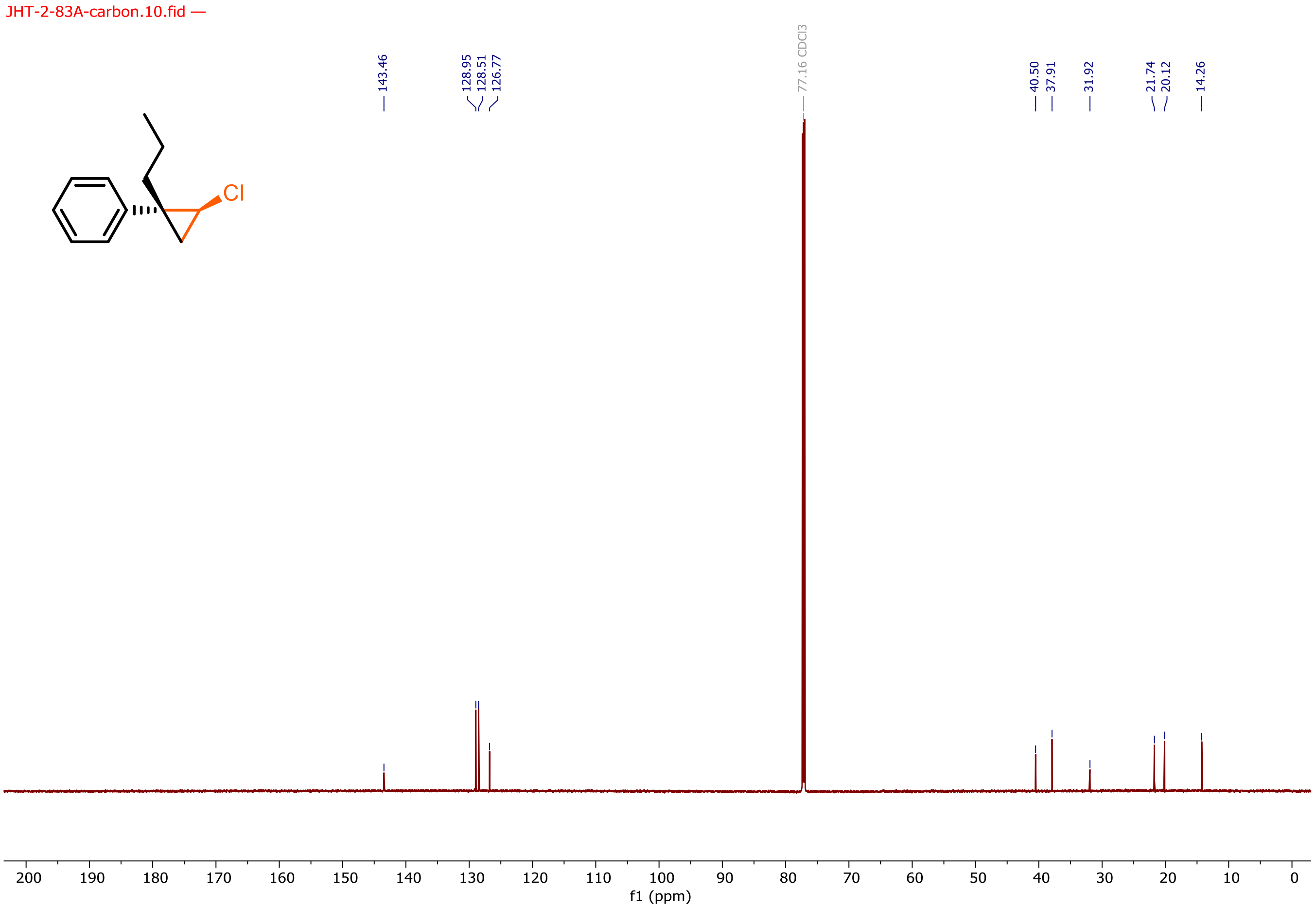

18: ¹H NMR (800 MHz, $CDCl_3$)

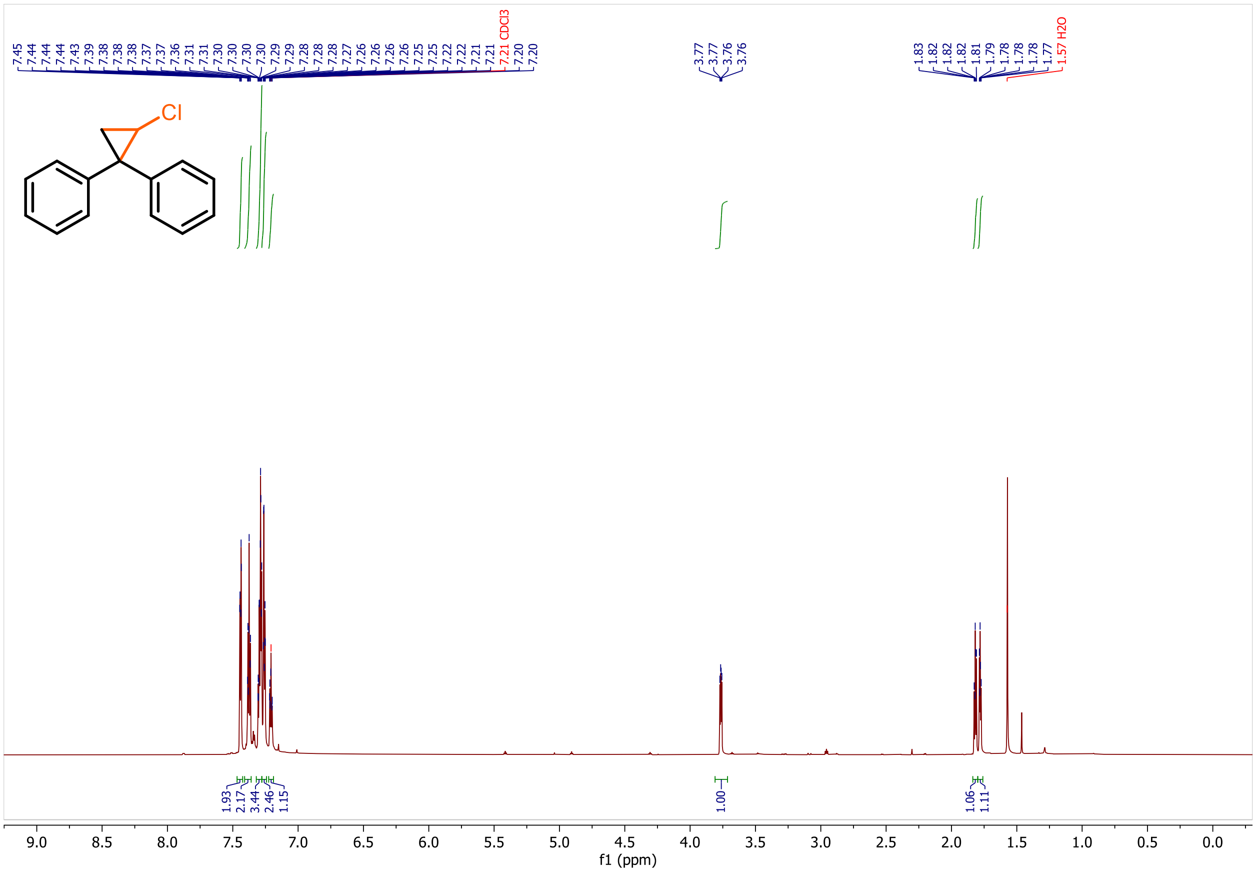

18: ^{13}C NMR (101 MHz, $CDCl_3$)

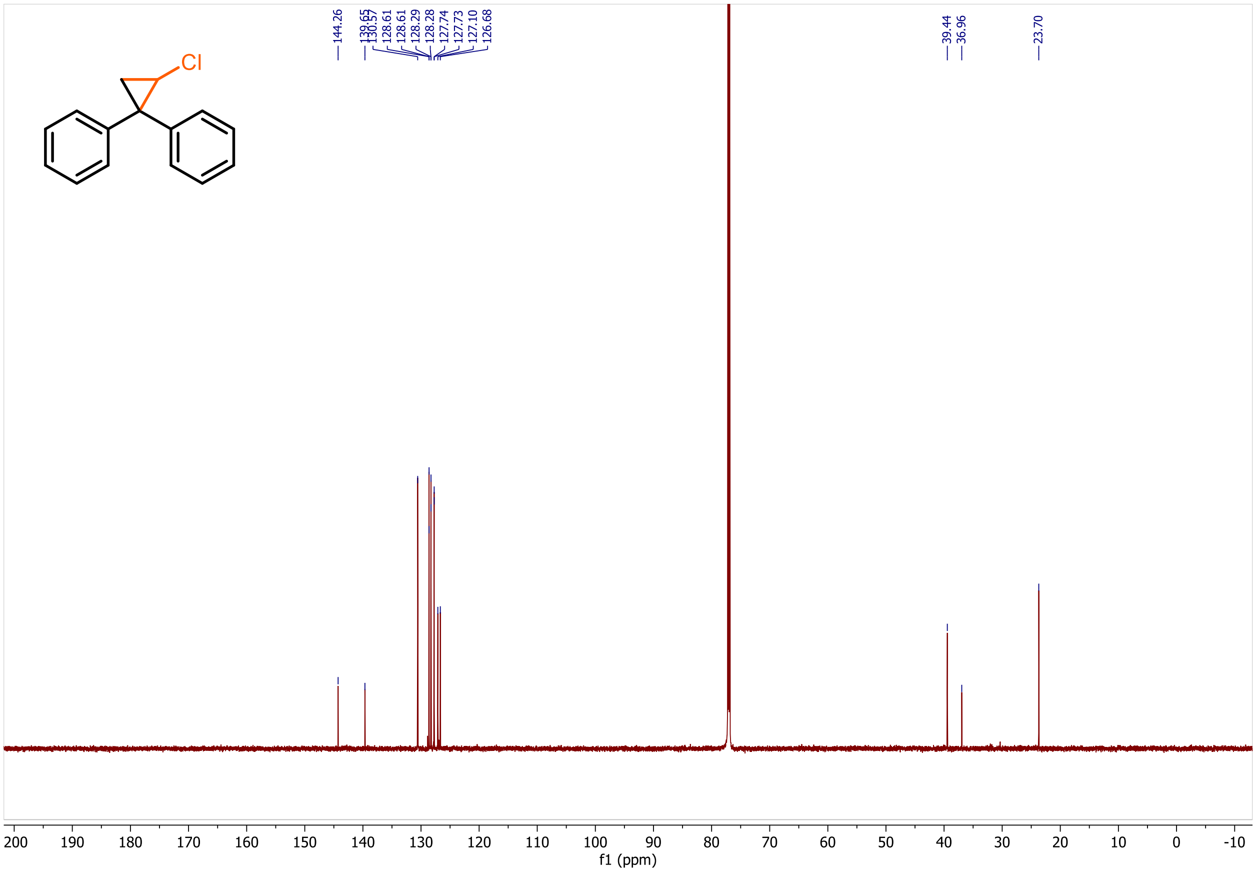

19: ¹H NMR (400 MHz, $CDCl_3$)

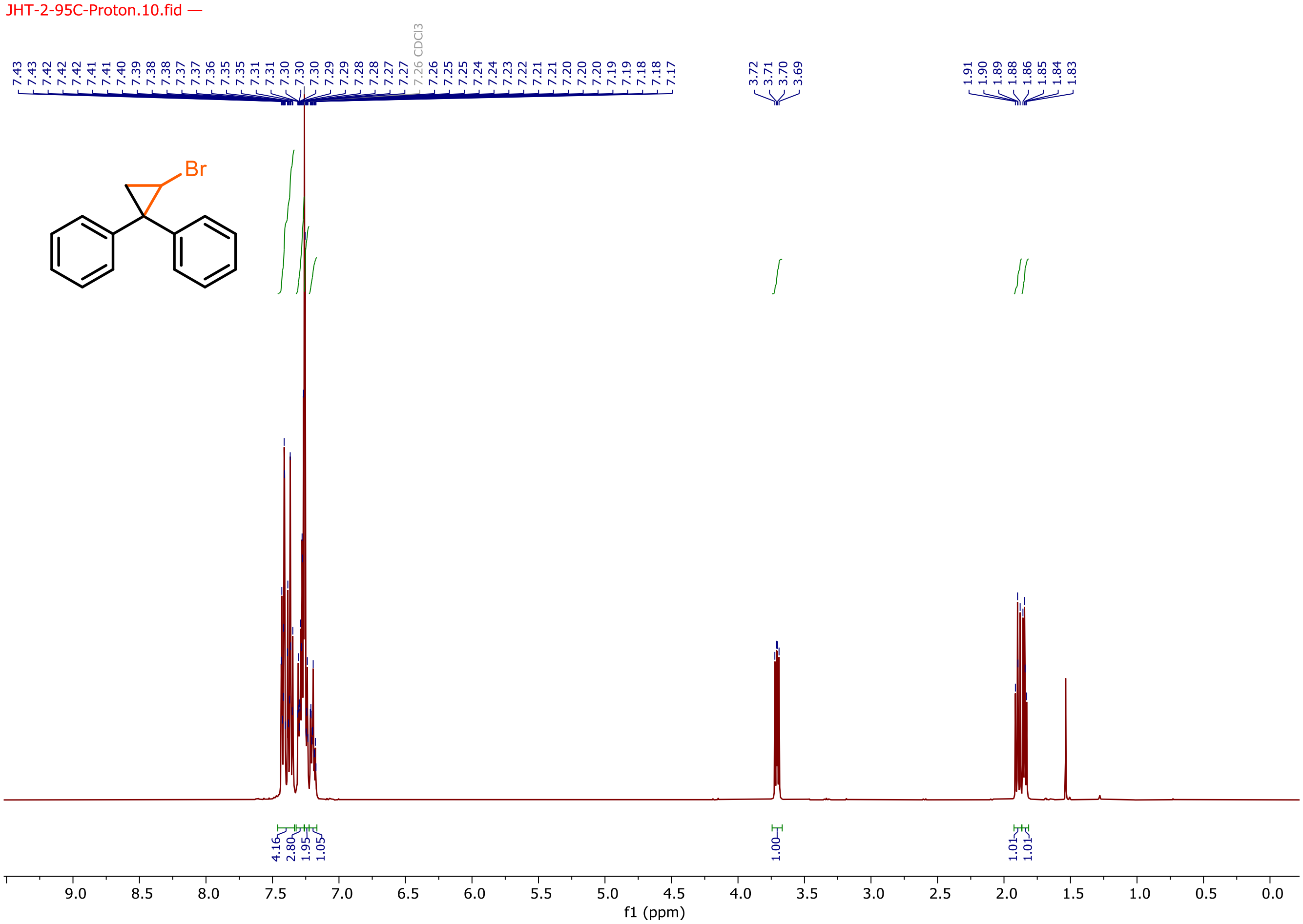

19: ^{13}C NMR (101 MHz, $CDCl_3$)

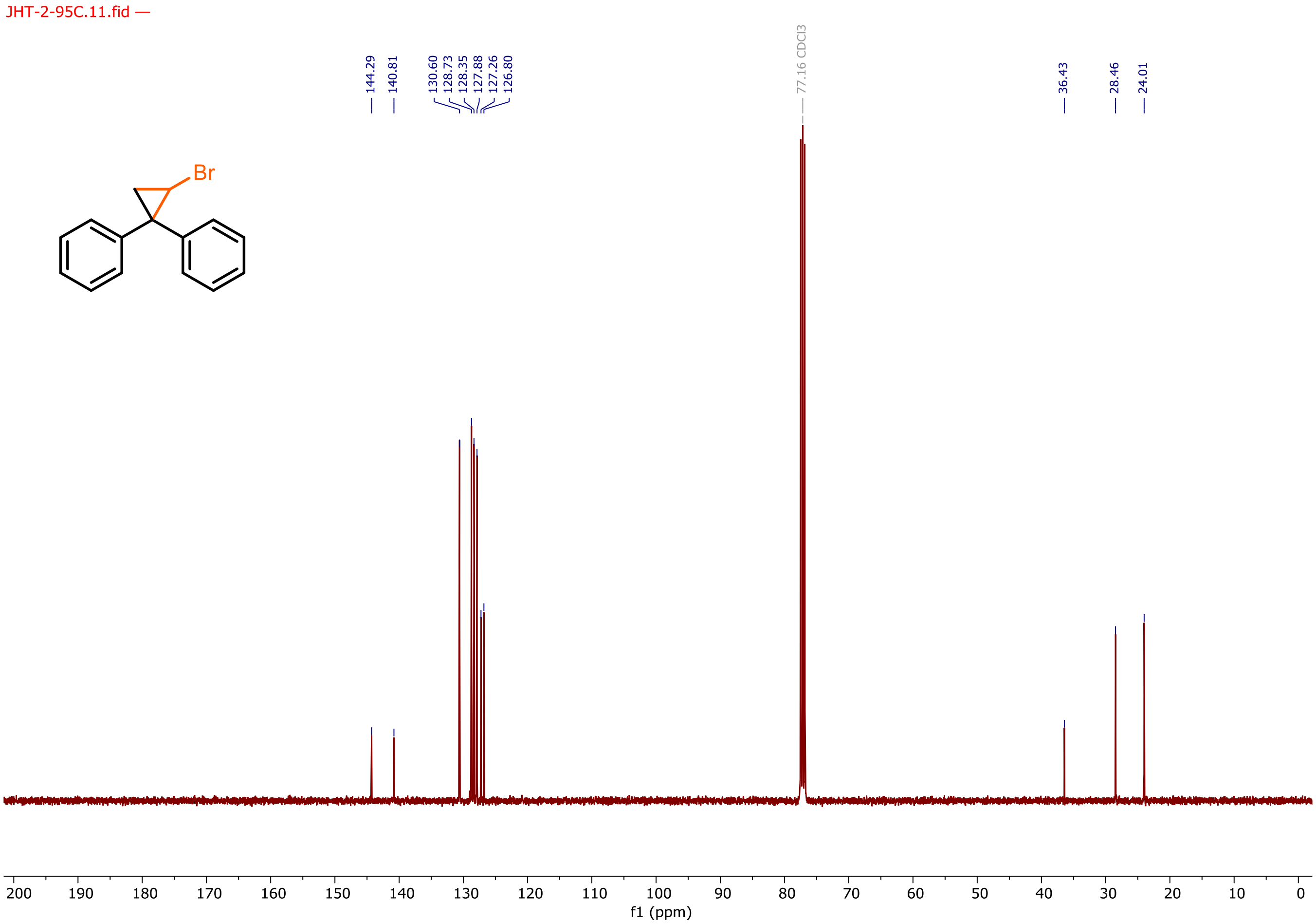

20:Crude ^{1}H NMR (400 MHz, $CDCl_3$)

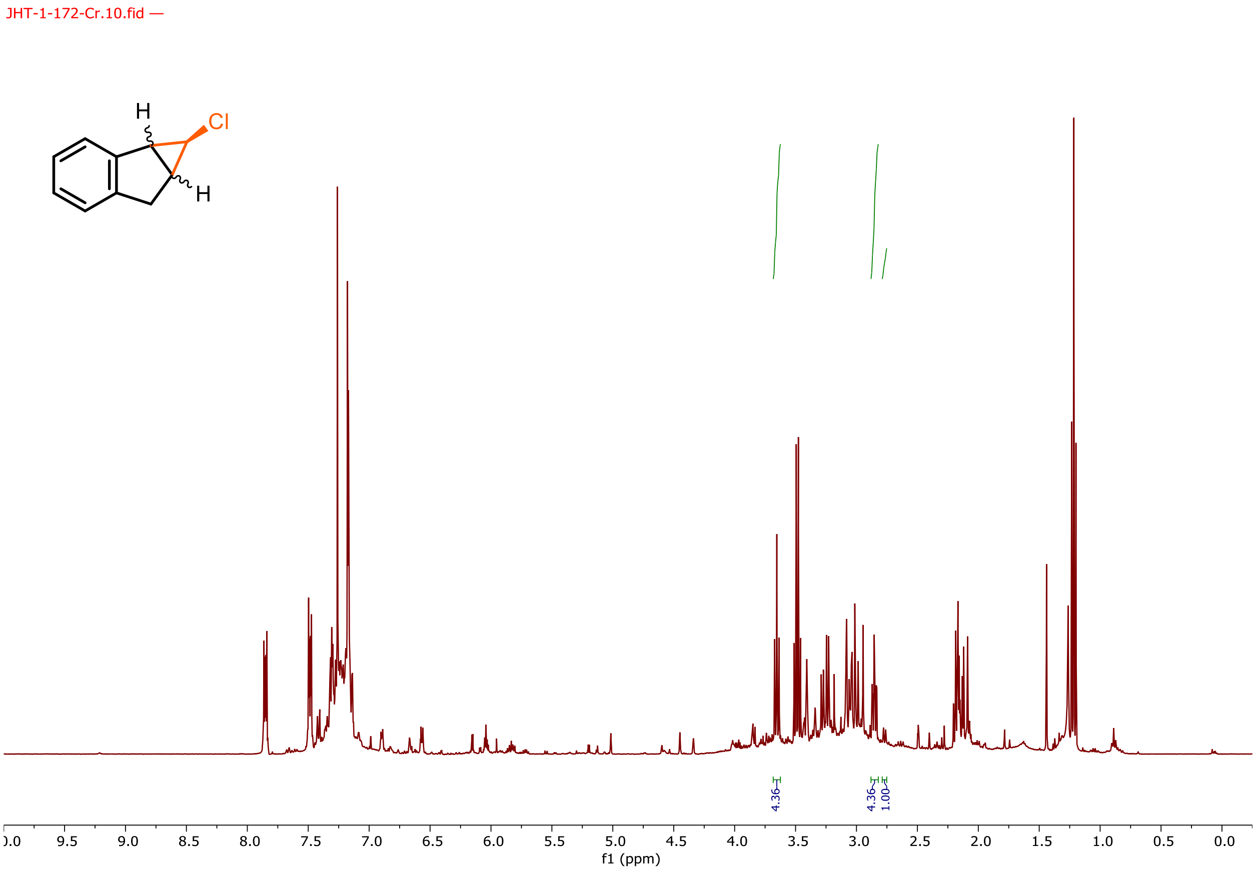

20: ^{1}H NMR (400 MHz, $CDCl_3$)

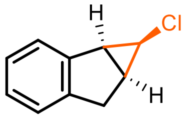

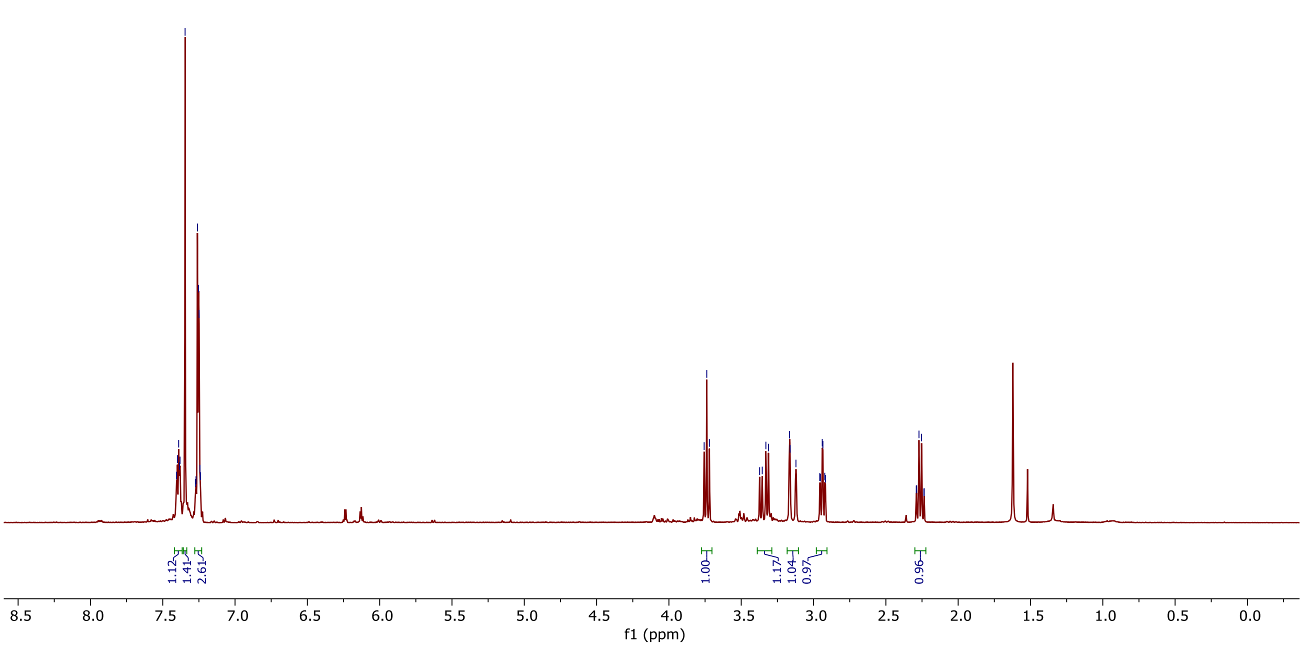

20: ^{13}C NMR (101 MHz, $CDCl_3$)

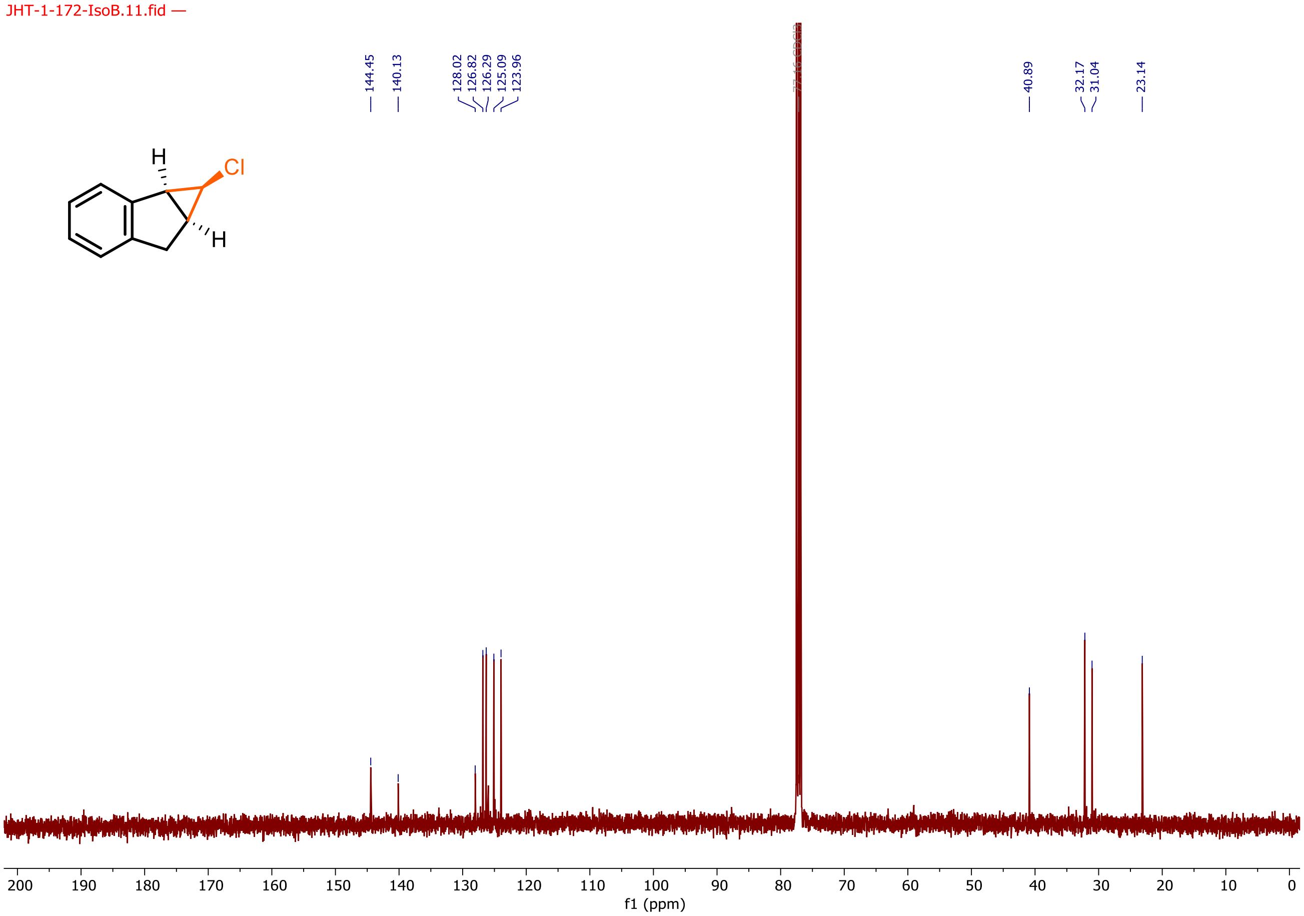

21: Crude ^{1}H NMR (400 MHz, $CDCl_3$)

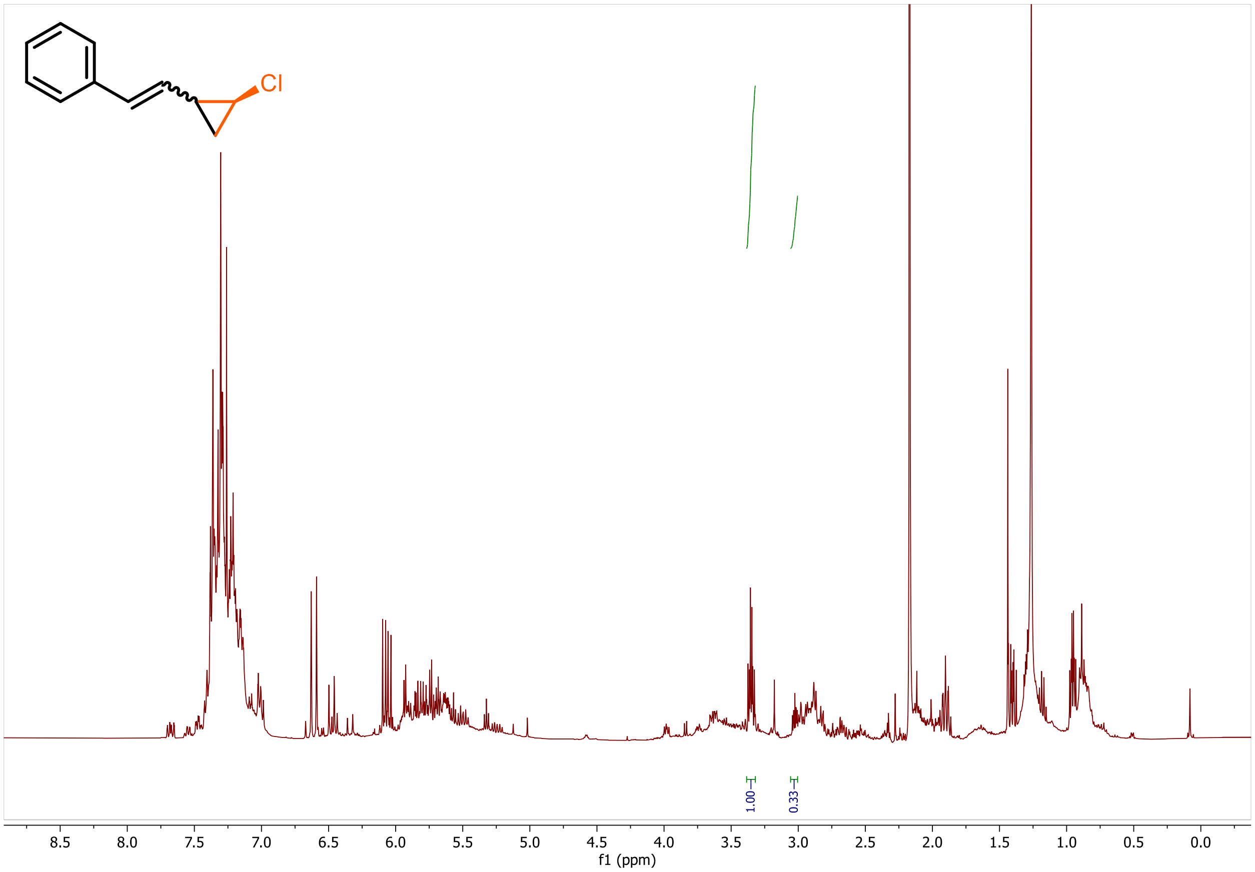

21: ^{1}H NMR (800 MHz, $CDCl_3$)

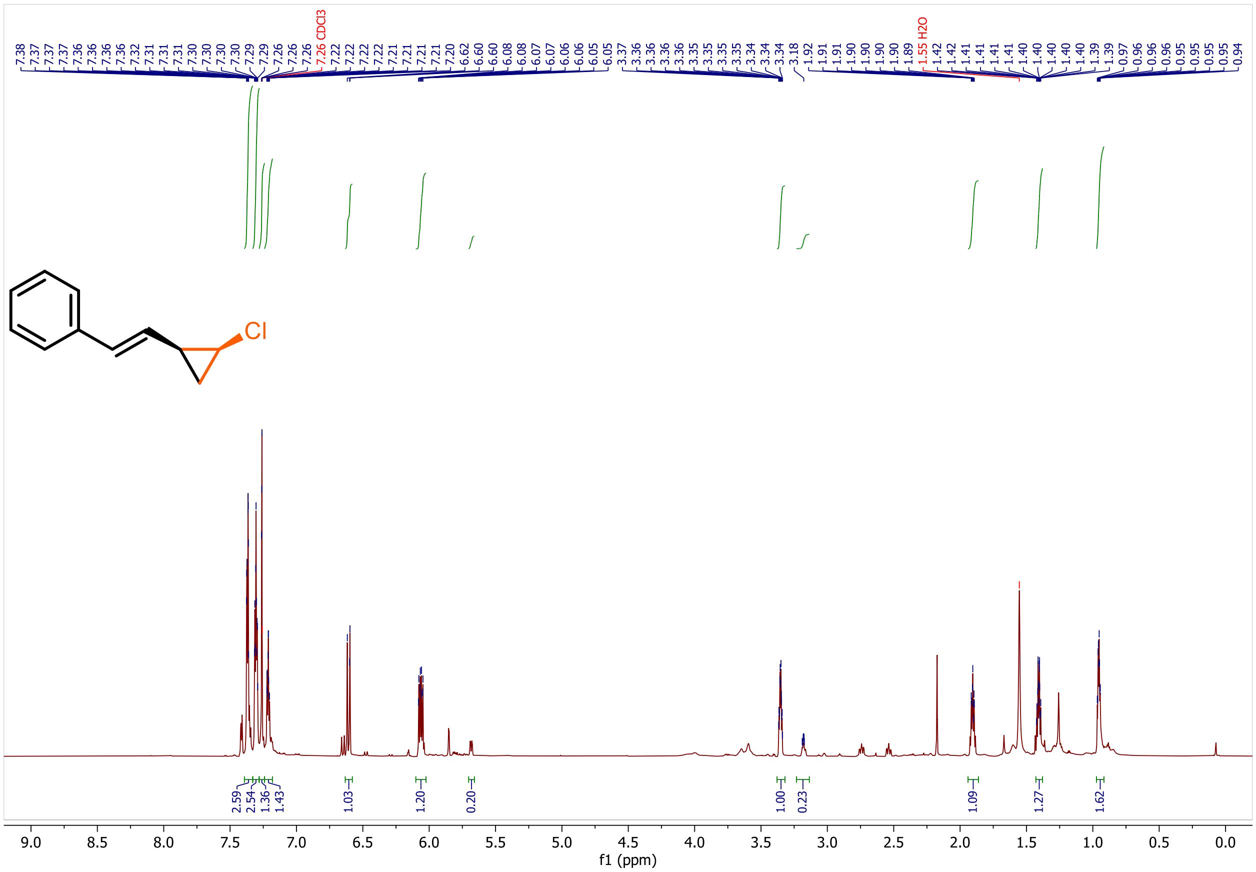

21: ^{13}C NMR (200 MHz, $CDCl_3$)

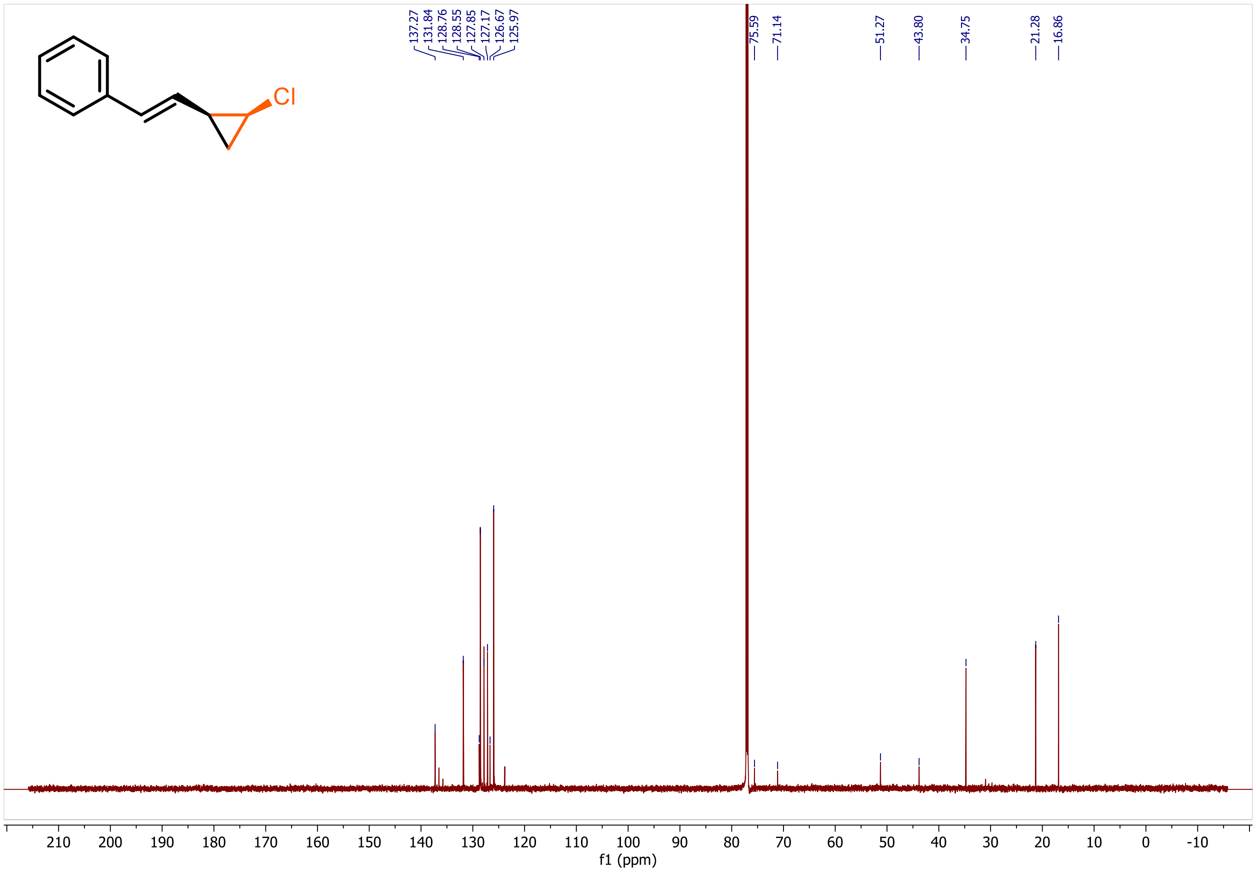

21: NOESY (400 MHz, $CDCl_3$)

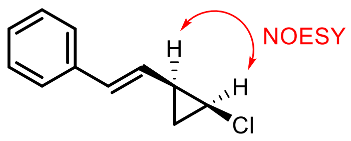

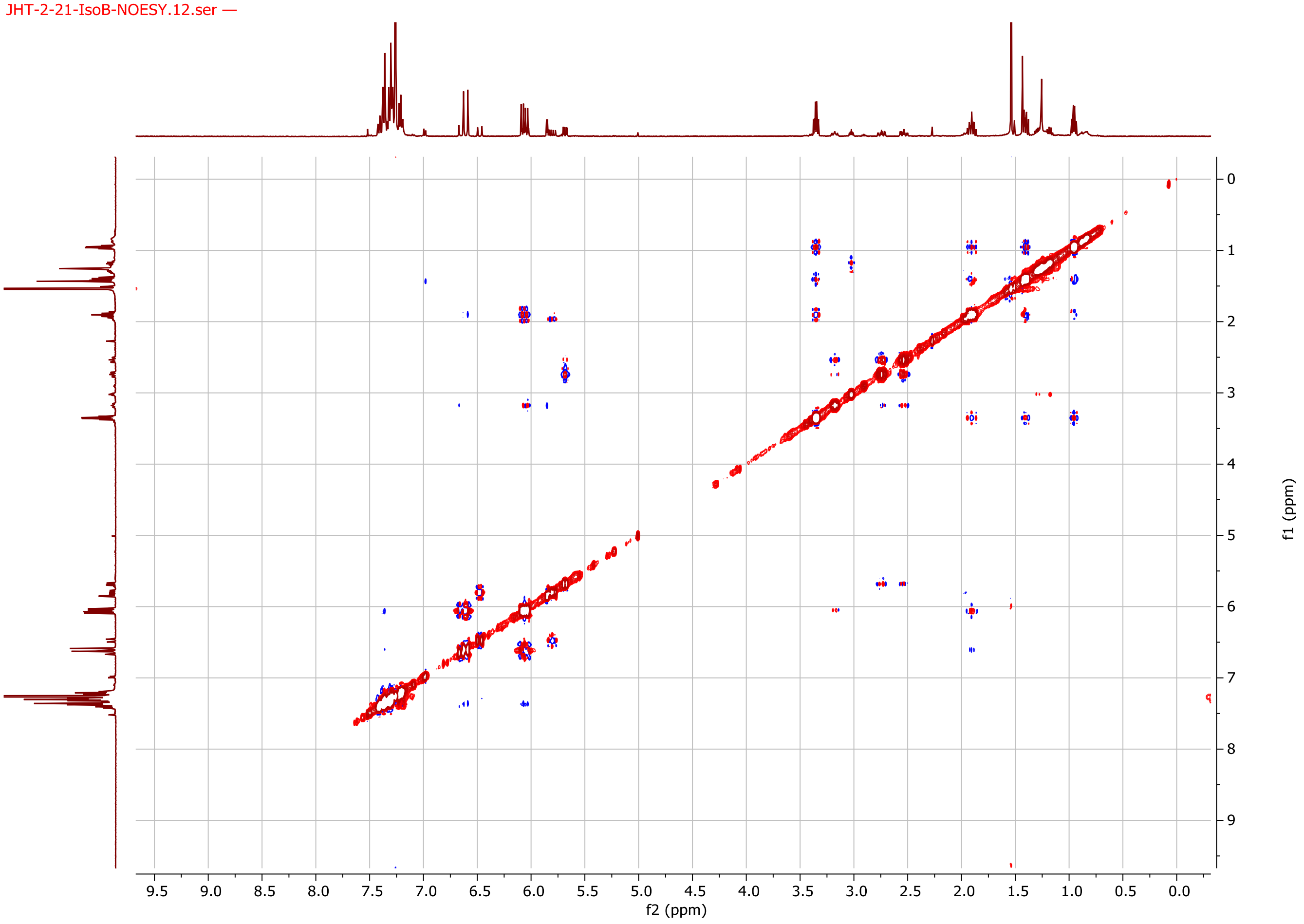

22: Crude ¹H NMR (400 MHz, $CDCl_3$)

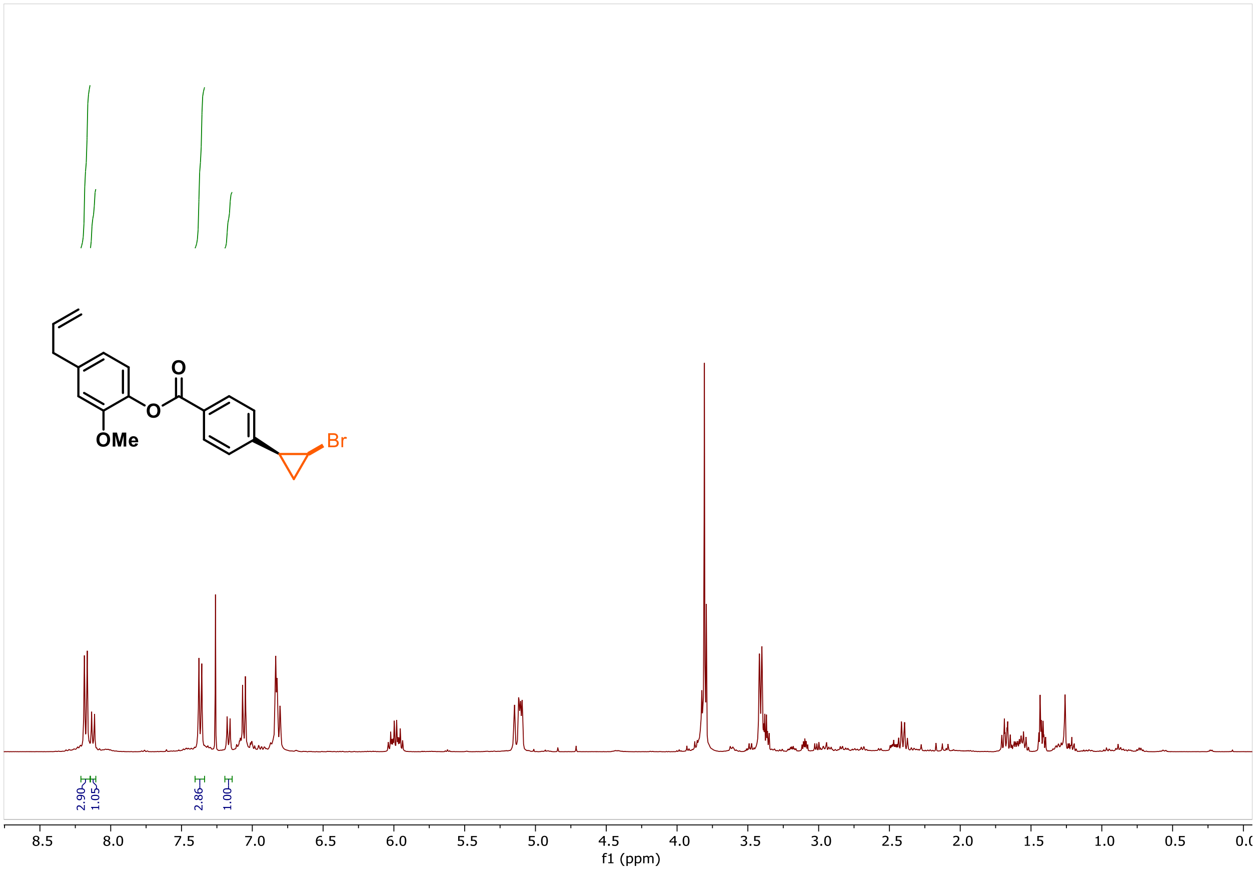

22: ^{1}H NMR (800 MHz, $CDCl_3$)

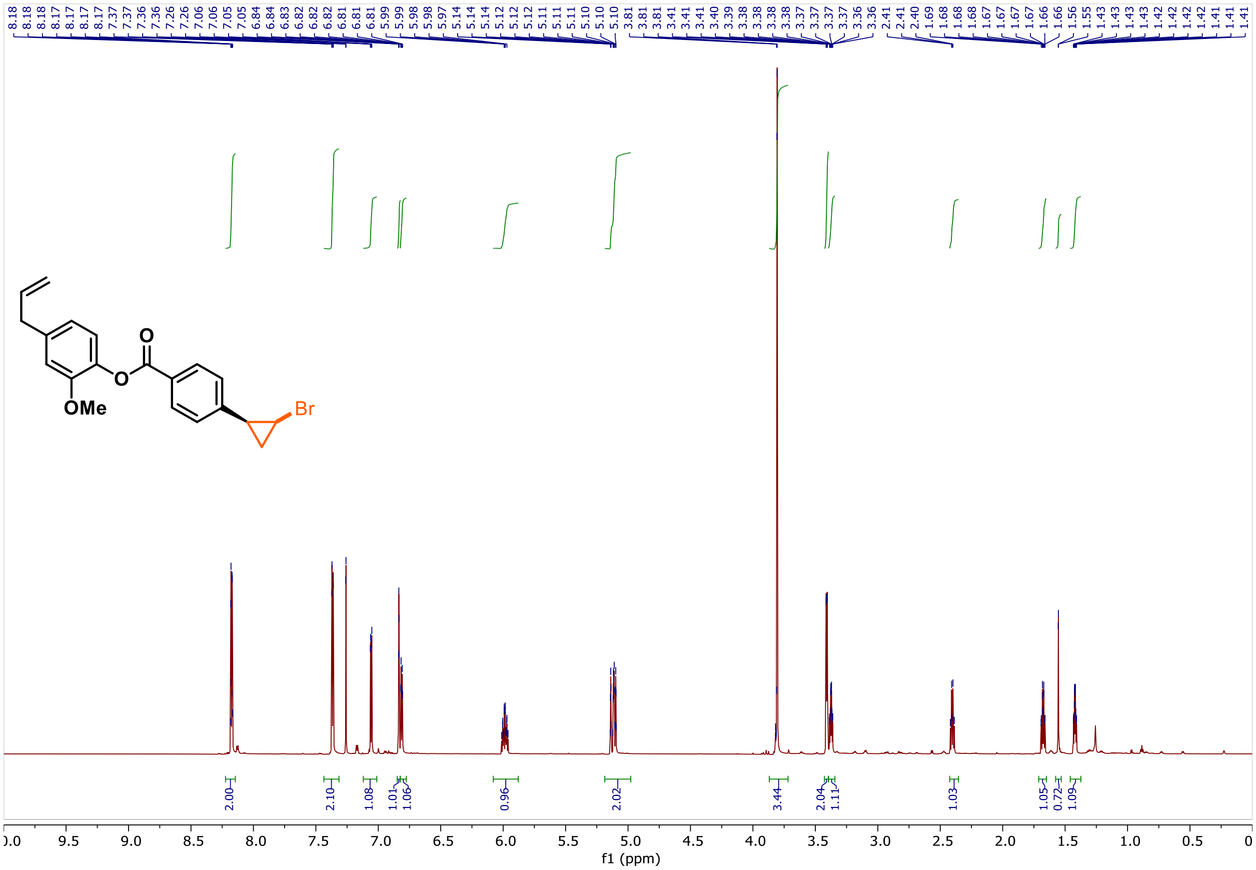

22: ^{13}C NMR (201 MHz, CDCl$_3$)

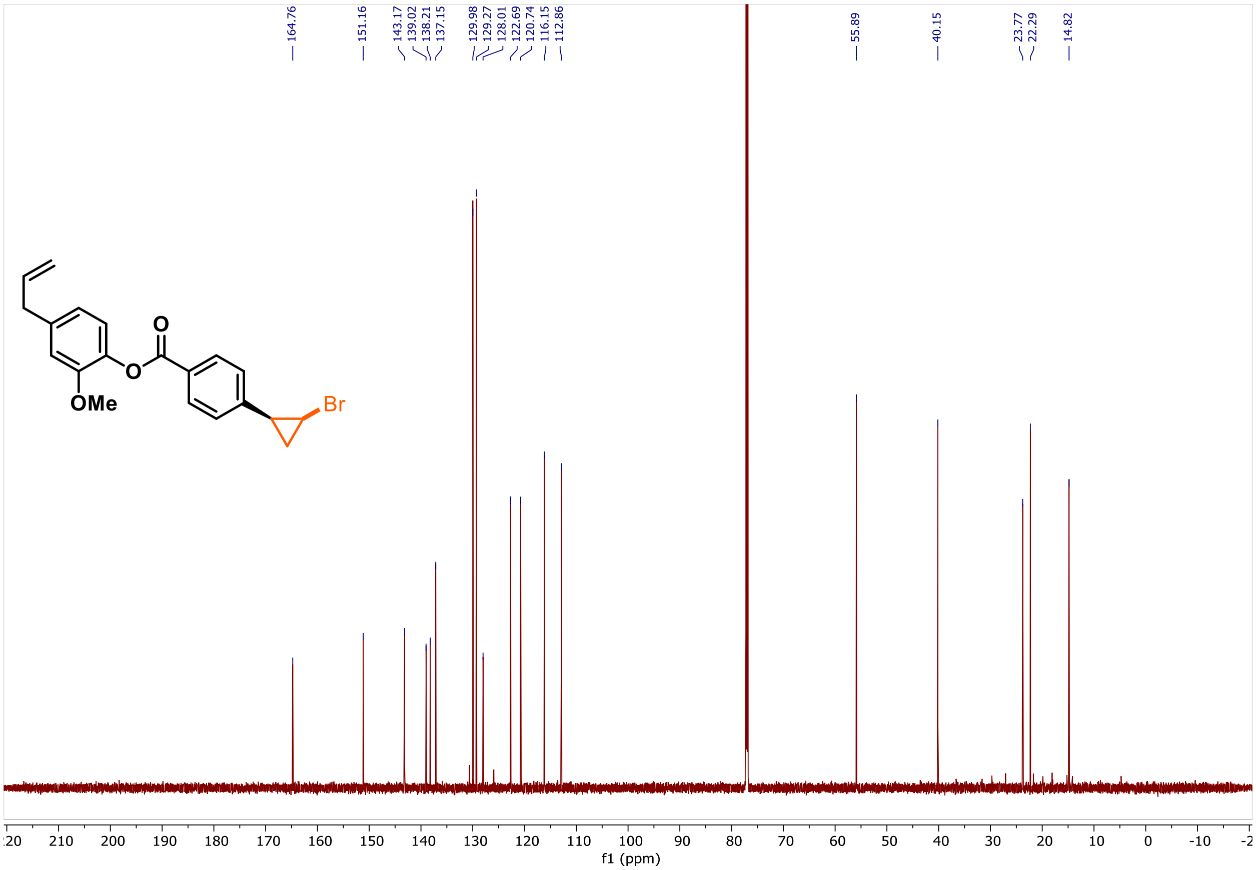

23: Crude ^{1}H NMR (400 MHz, $CDCl_3$)

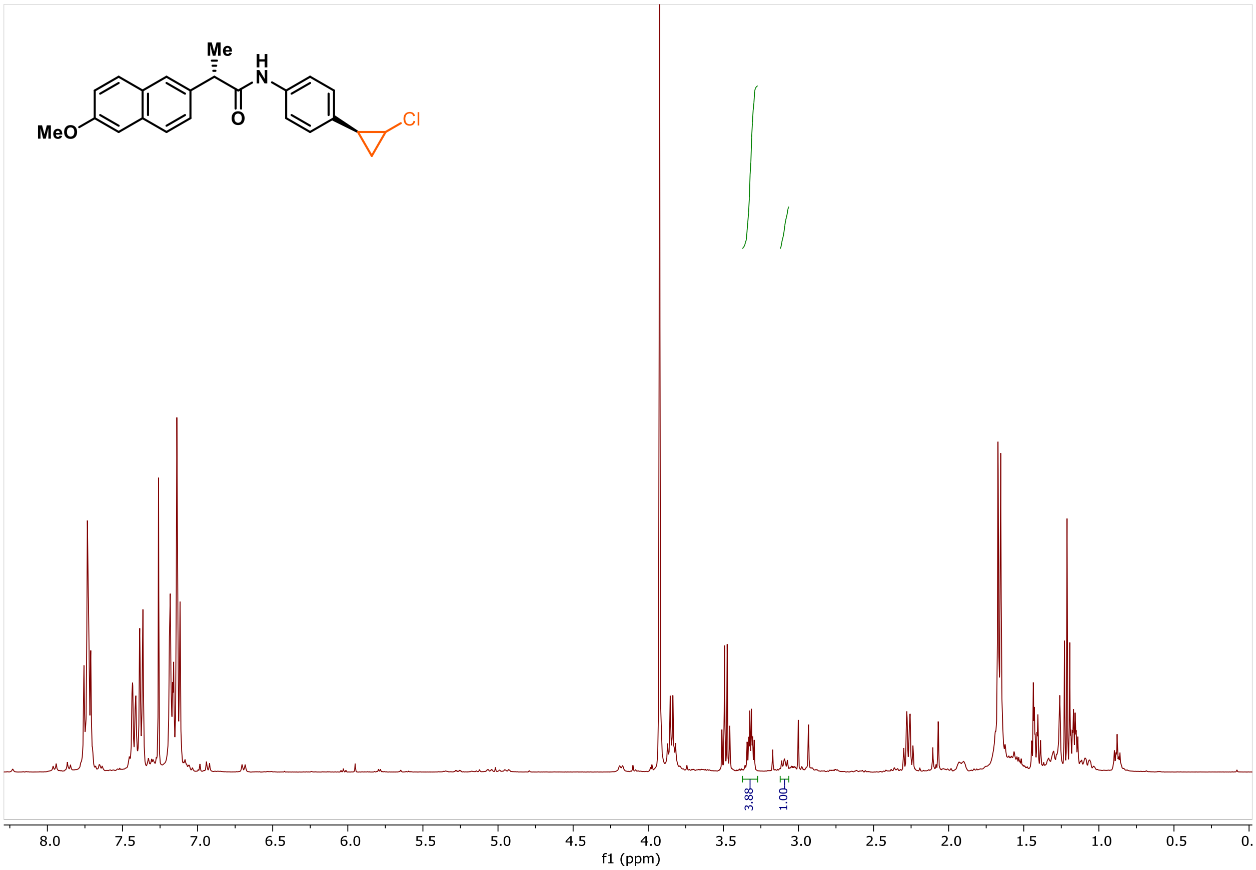

23: ^{1}H NMR (800 MHz, $CDCl_3$)

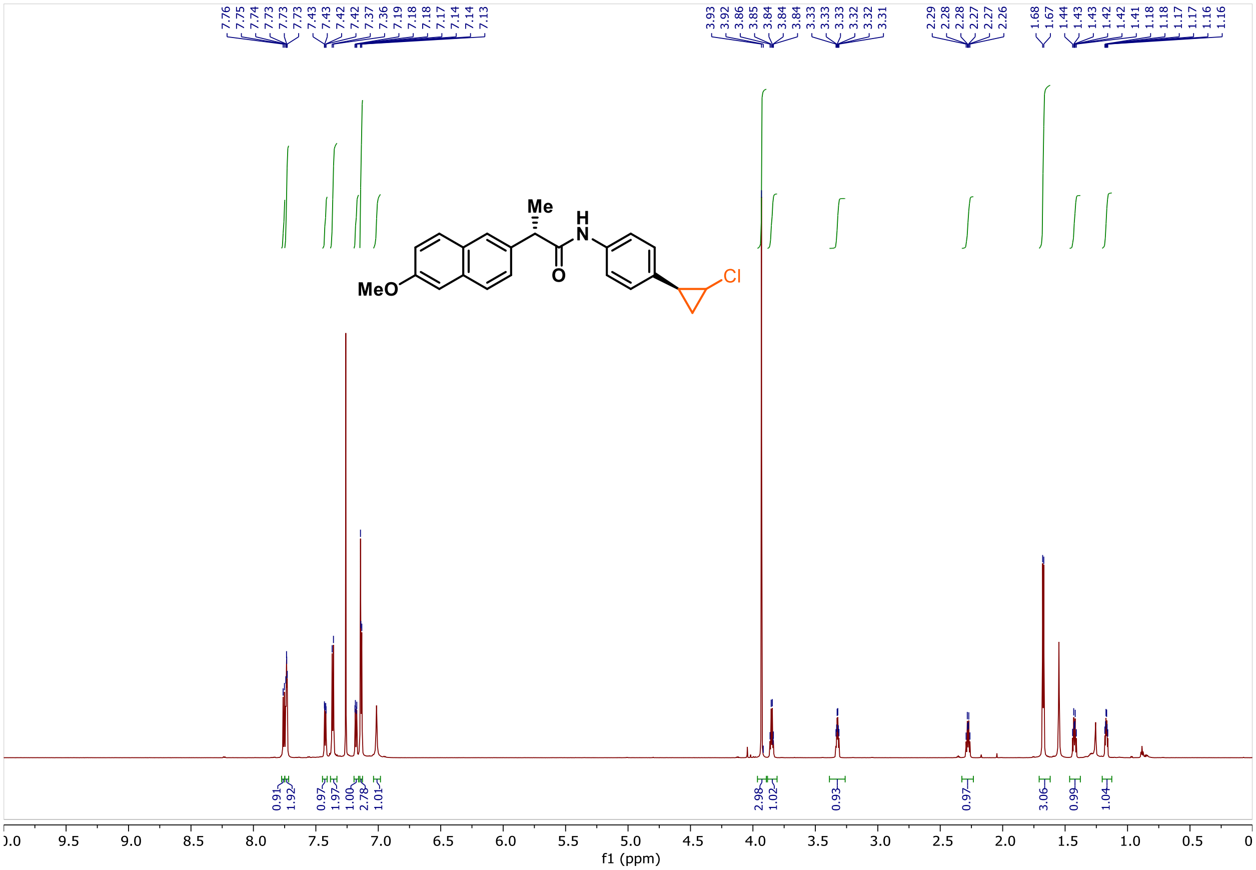

23: ^{13}C NMR (201 MHz, $CDCl_3$)

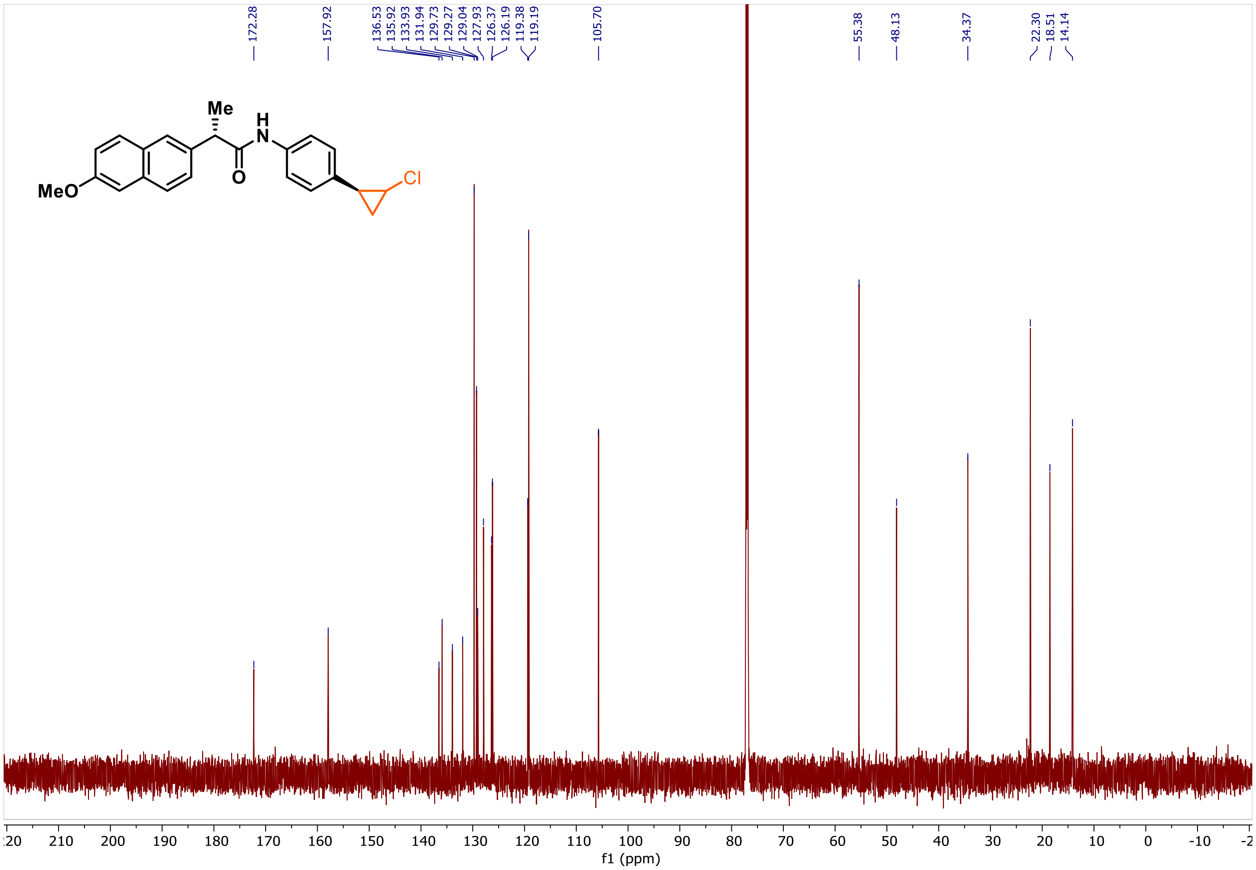

24: Crude ^{1}H NMR (400 MHz, $CDCl_3$)

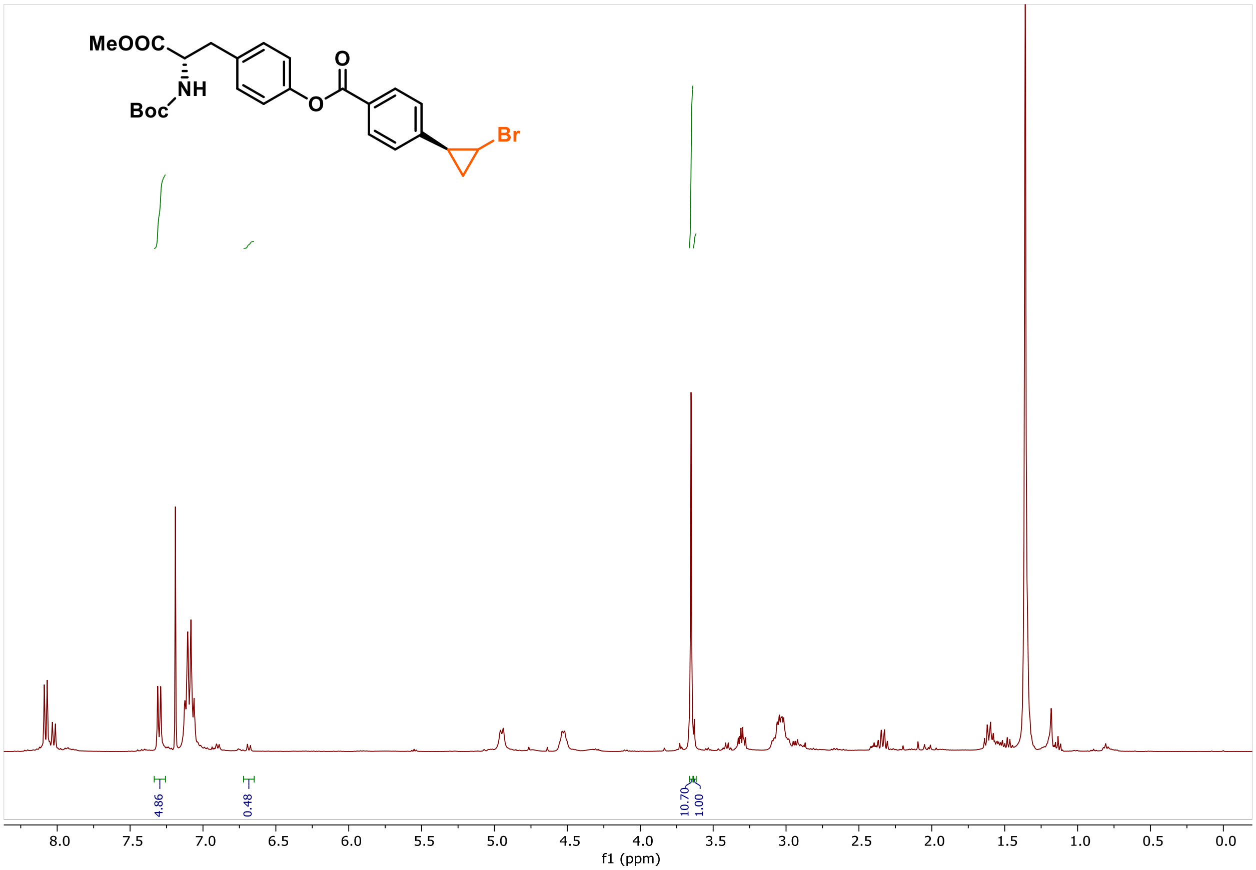

24: ^{1}H NMR (800 MHz, $CDCl_3$)

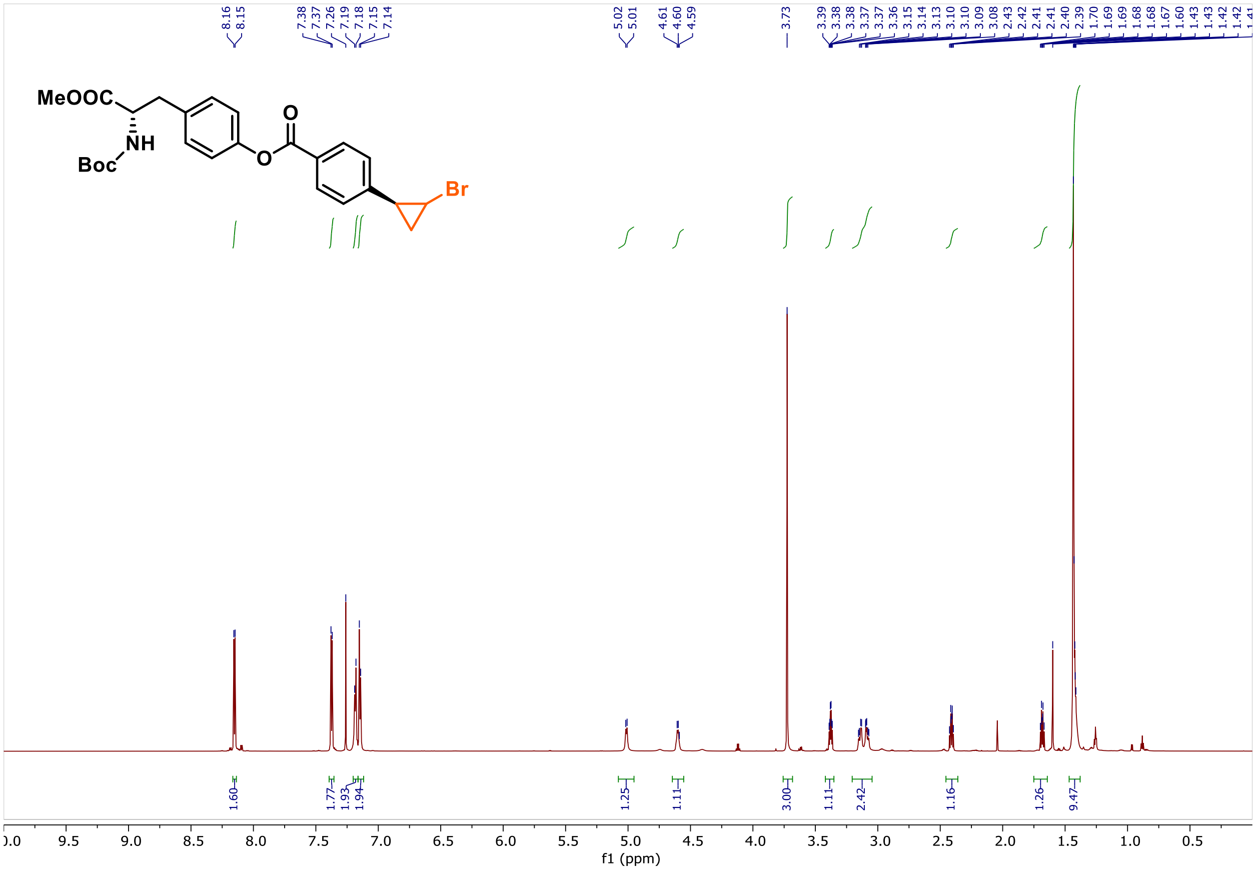

24: ^{13}C NMR (201 MHz, $CDCl_3$)

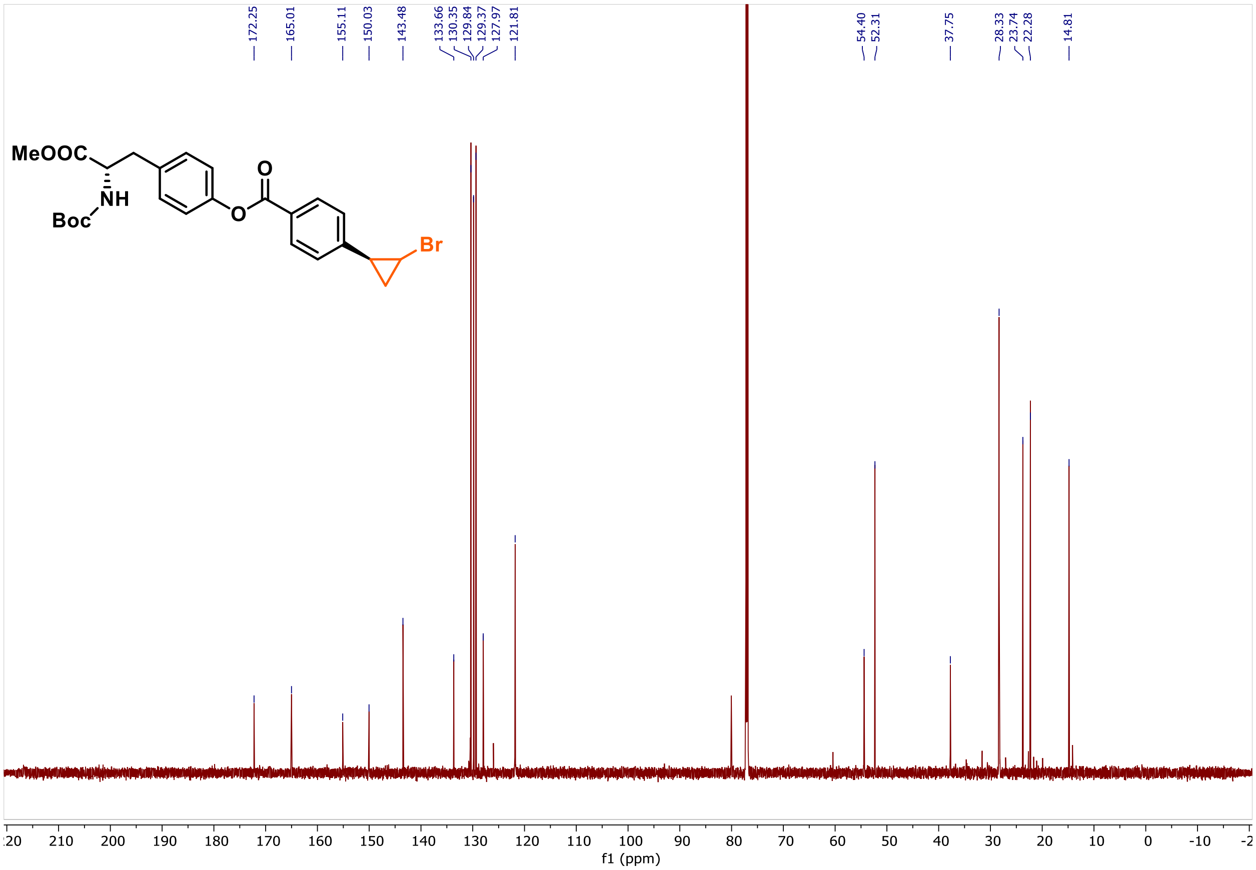

25: Crude ^{1}H NMR (400 MHz, $CDCl_3$)

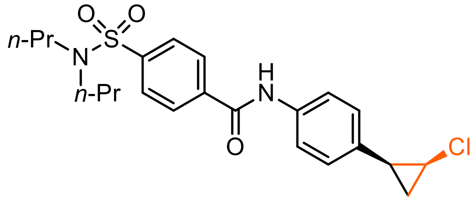

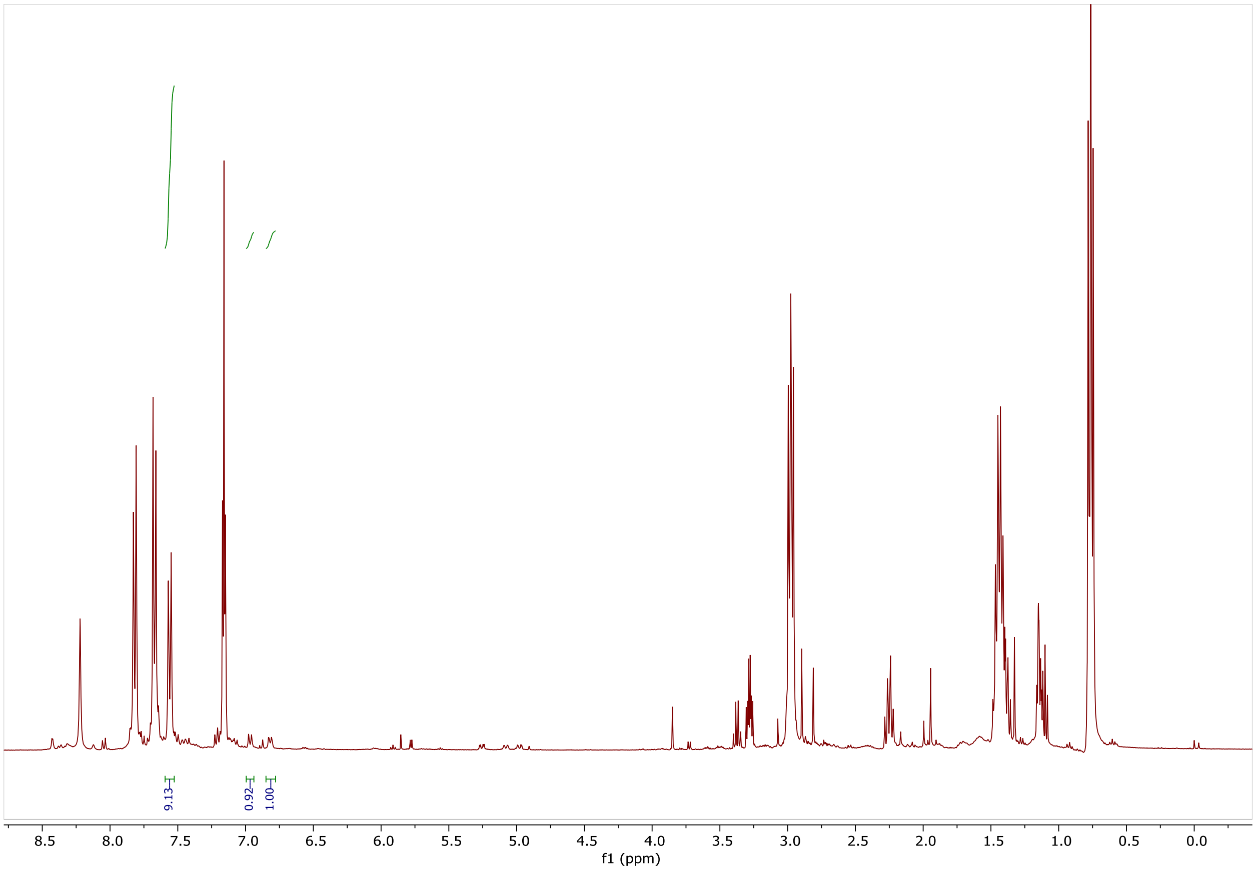

25: ^{1}H NMR (800 MHz, $CDCl_3$)

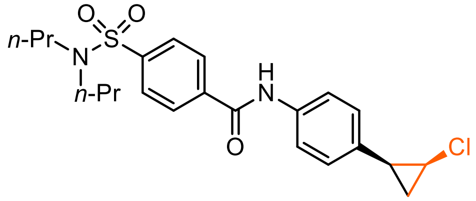

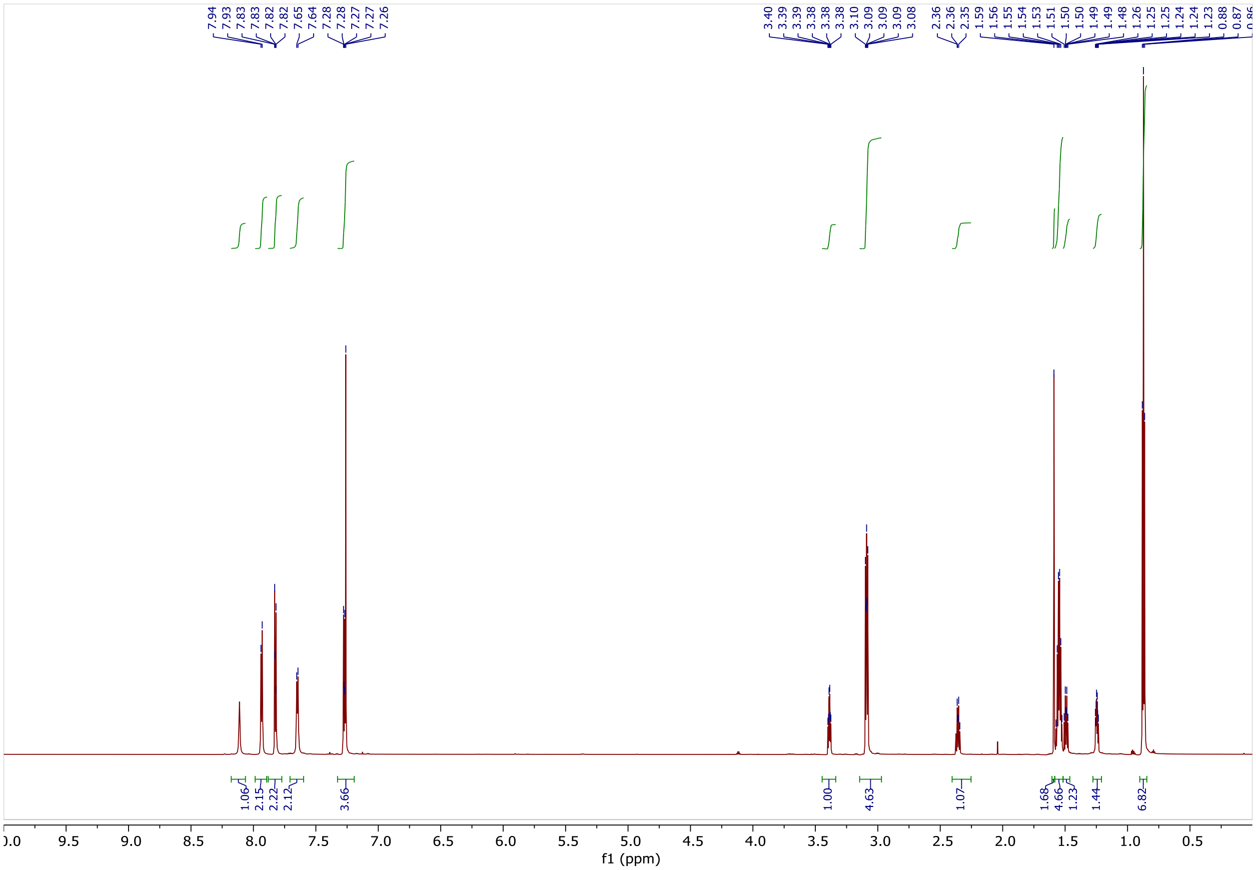

25: ^{13}**C NMR** (201 MHz, $CDCl_3$)

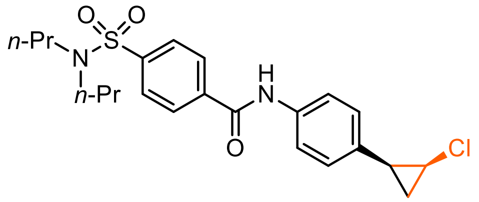

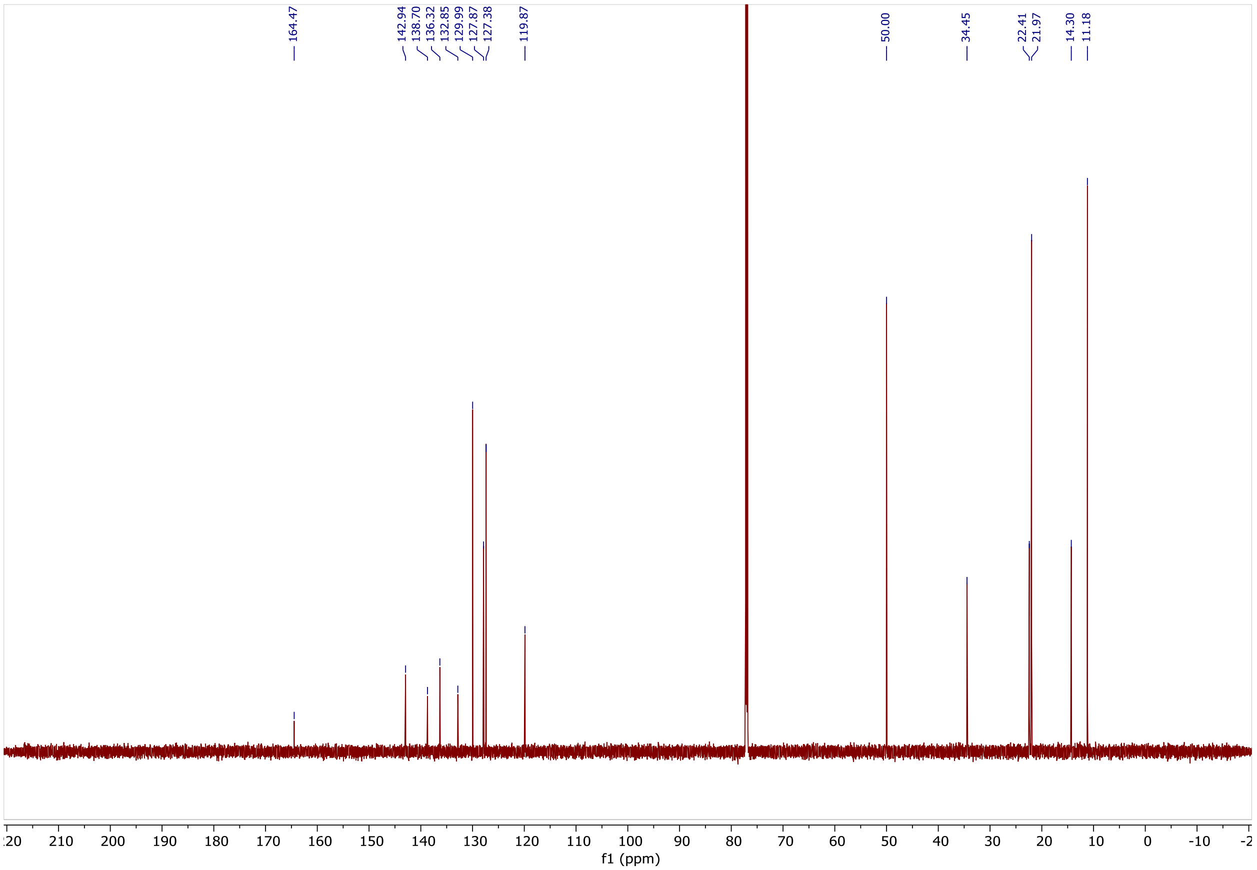

26: Crude ^{1}H NMR (400 MHz, $CDCl_3$)

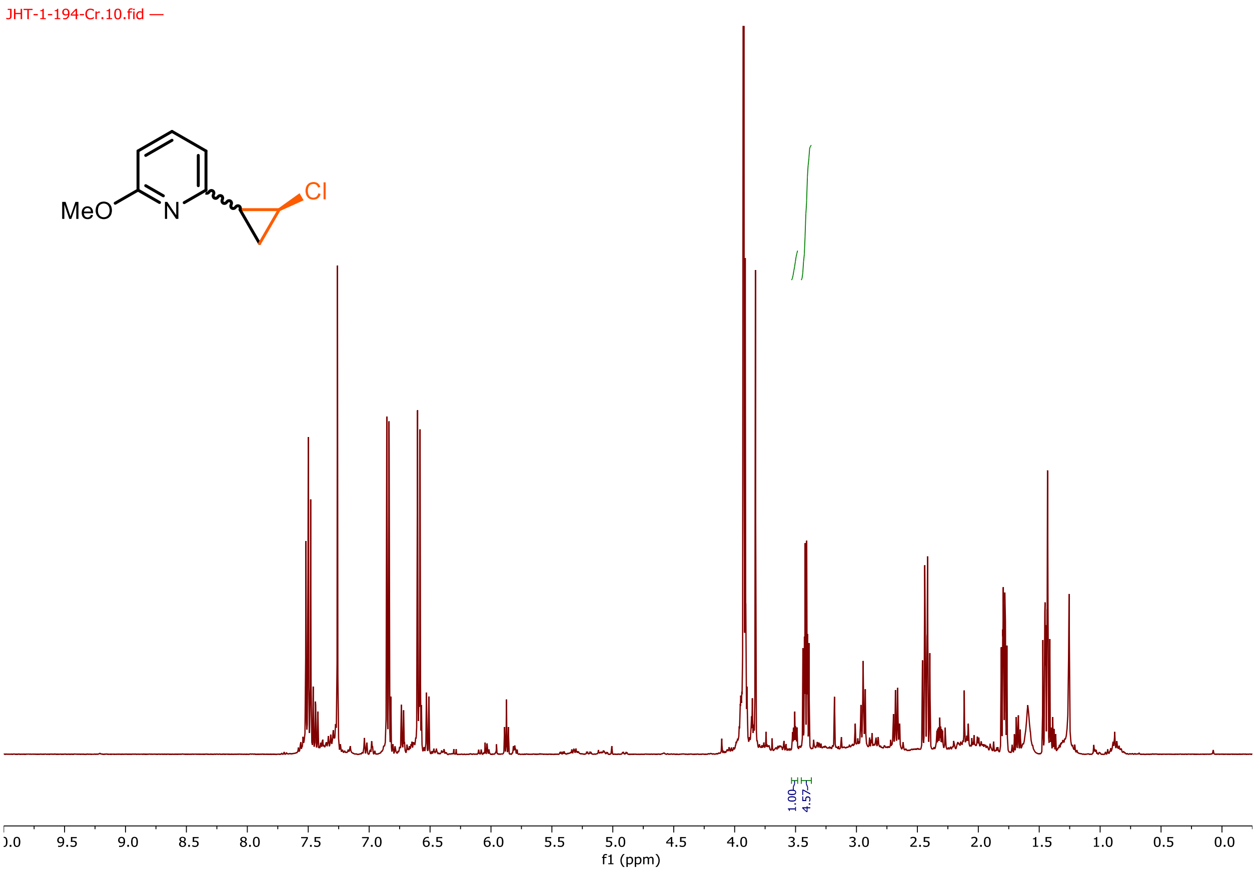

26: ^{1}H NMR (400 MHz, $CDCl_3$)

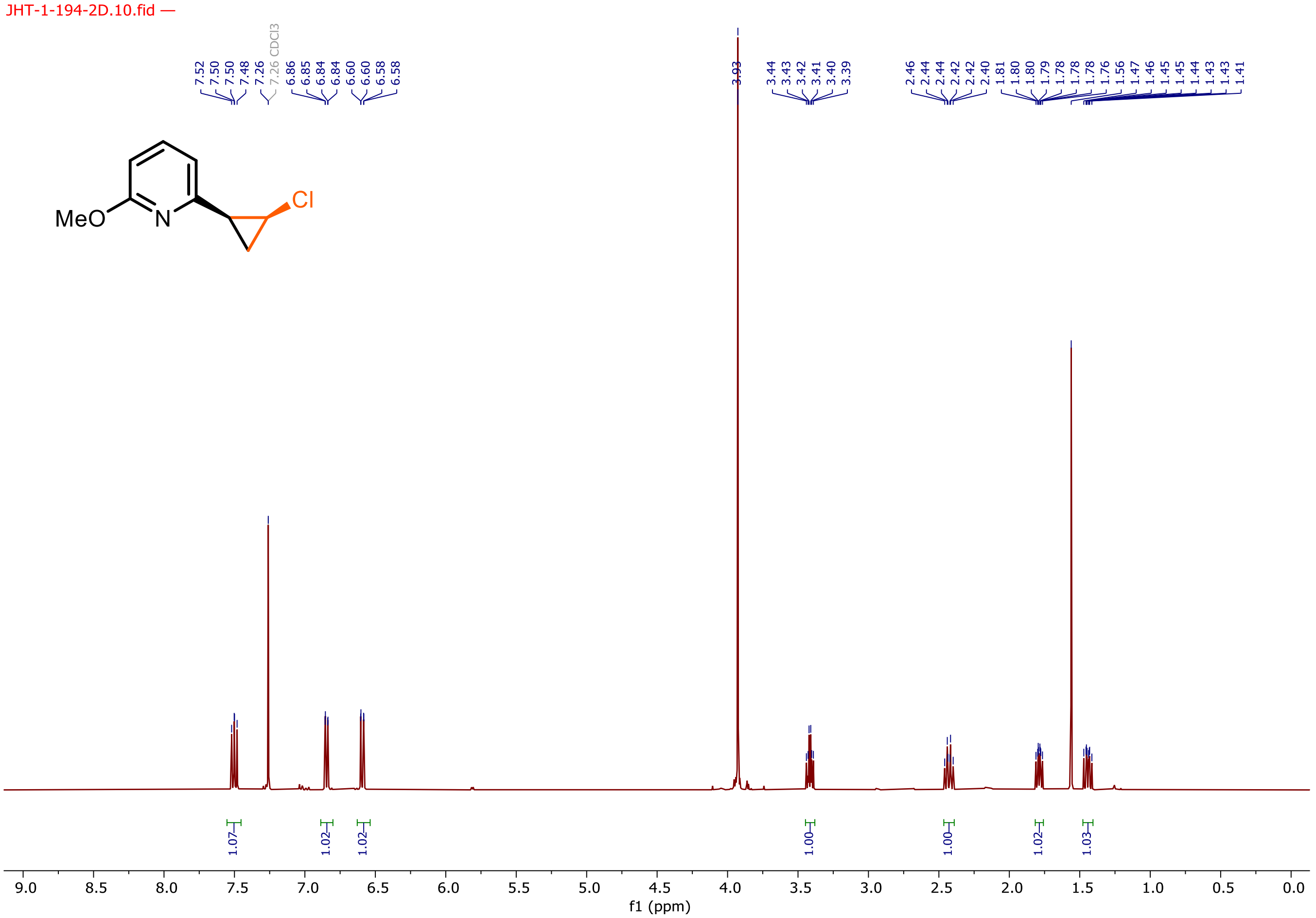

26: ^{13}C NMR (101 MHz, $CDCl_3$)

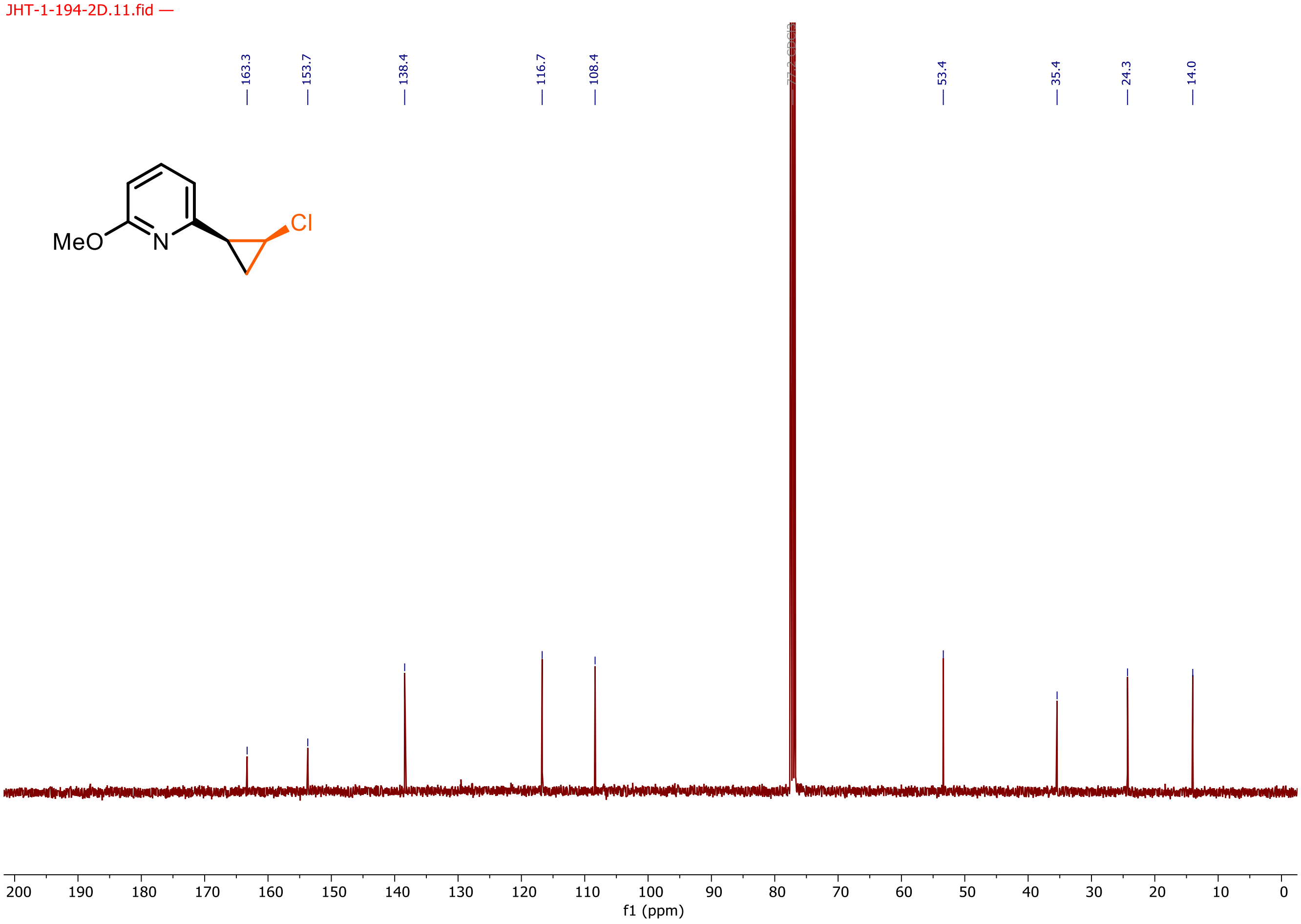

27: Crude ^{1}H NMR (400 MHz, $CDCl_3$)

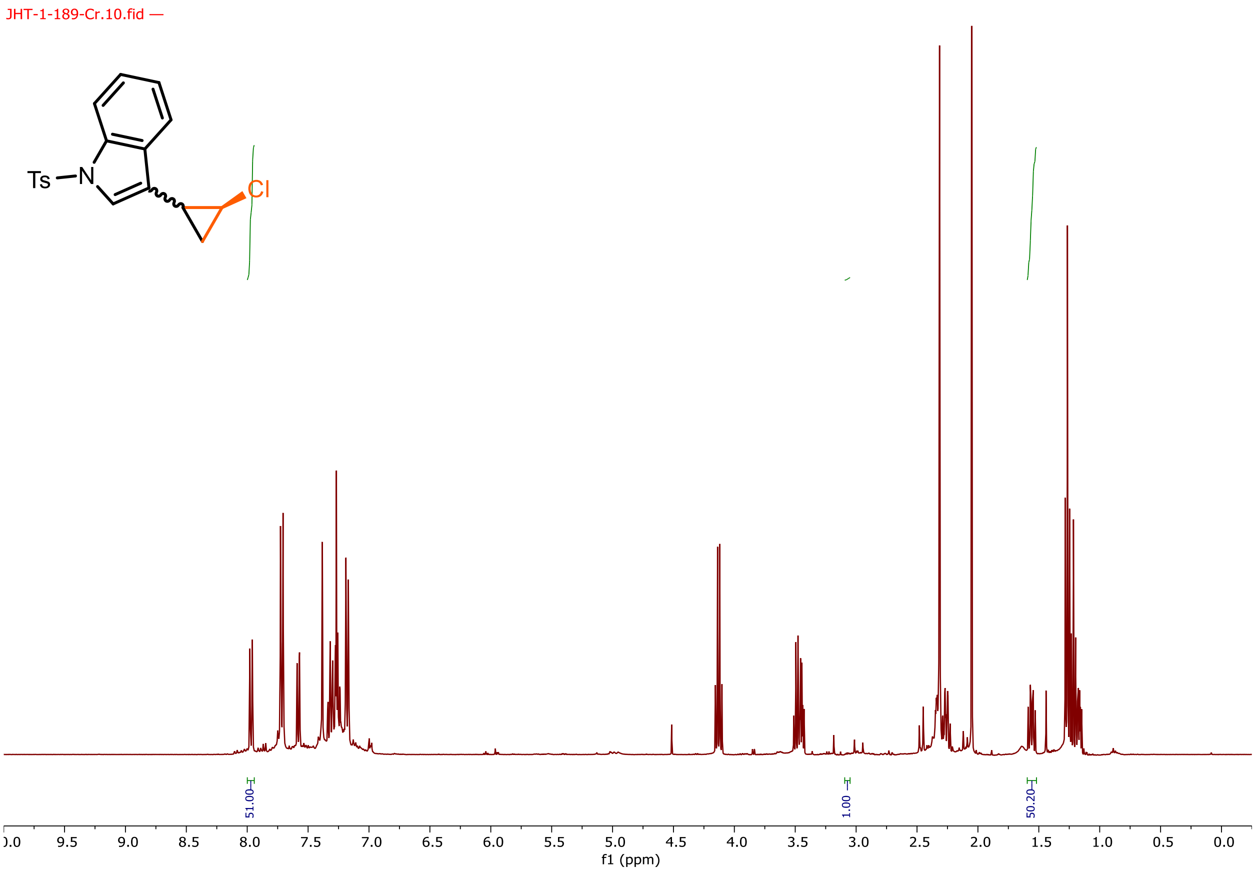

27: ^{1}H NMR (400 MHz, $CDCl_3$)

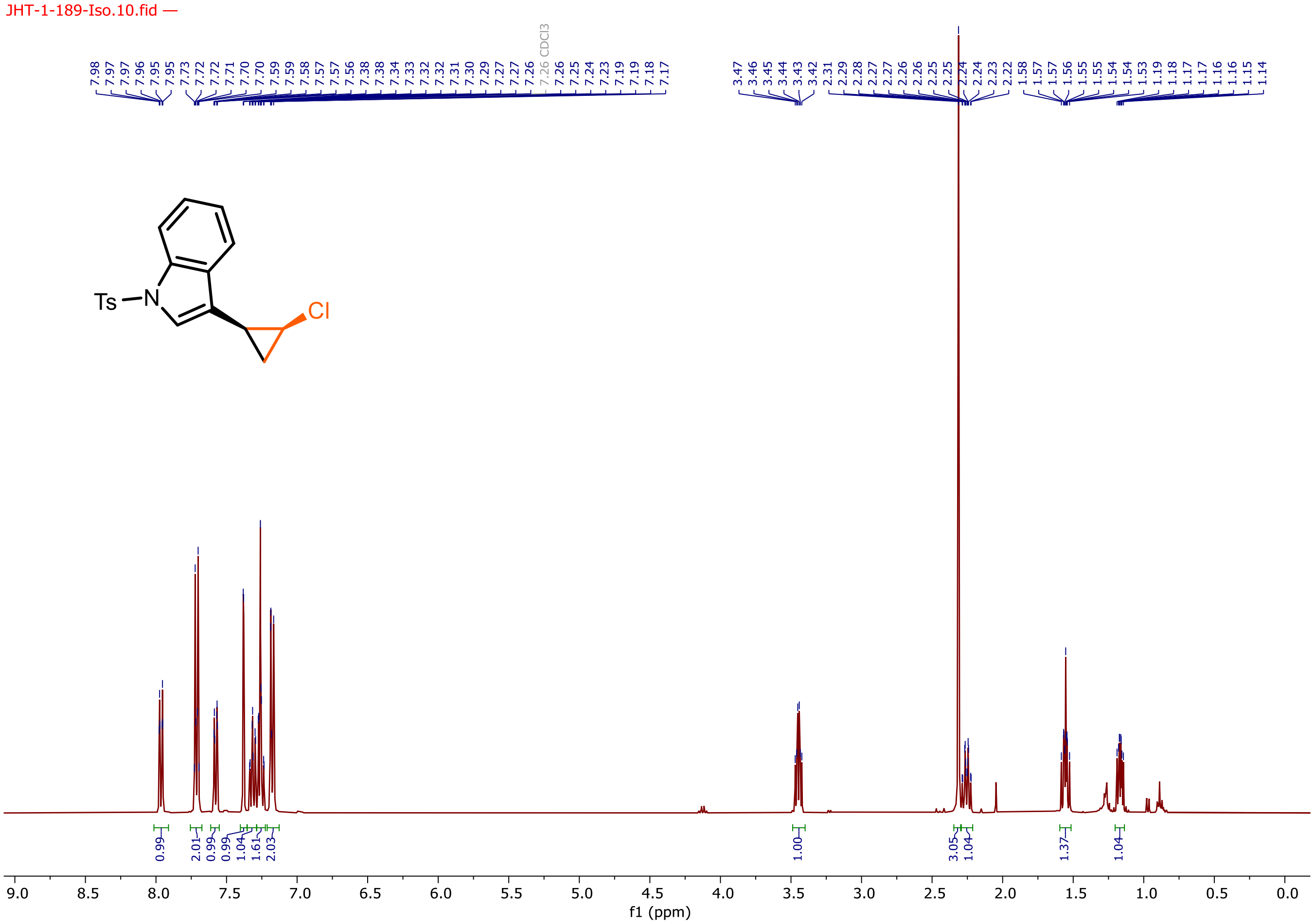

27: ^{13}C NMR (101 MHz, $CDCl_3$)

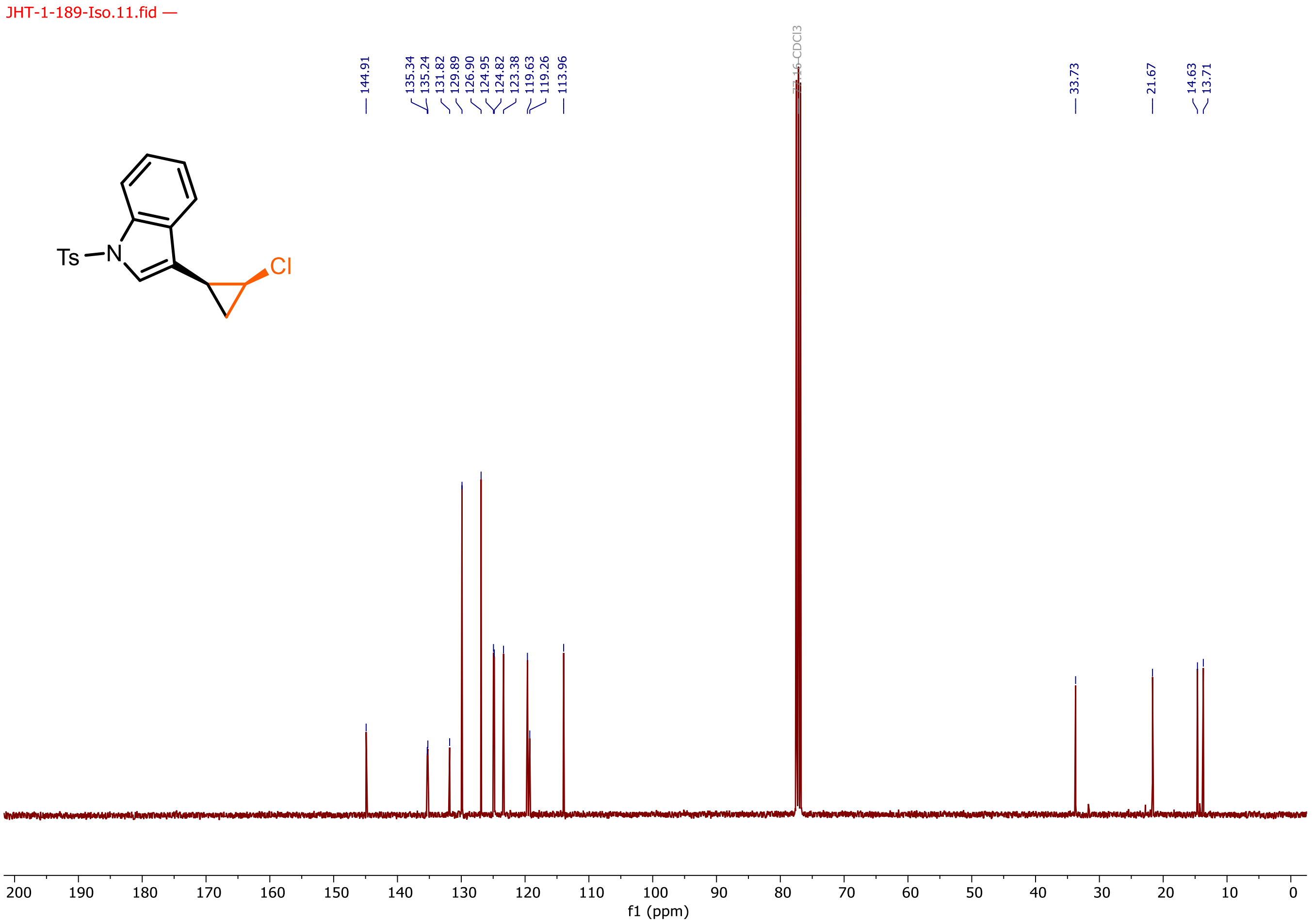

28: Crude ^{1}H NMR (400 MHz, $CDCl_3$)

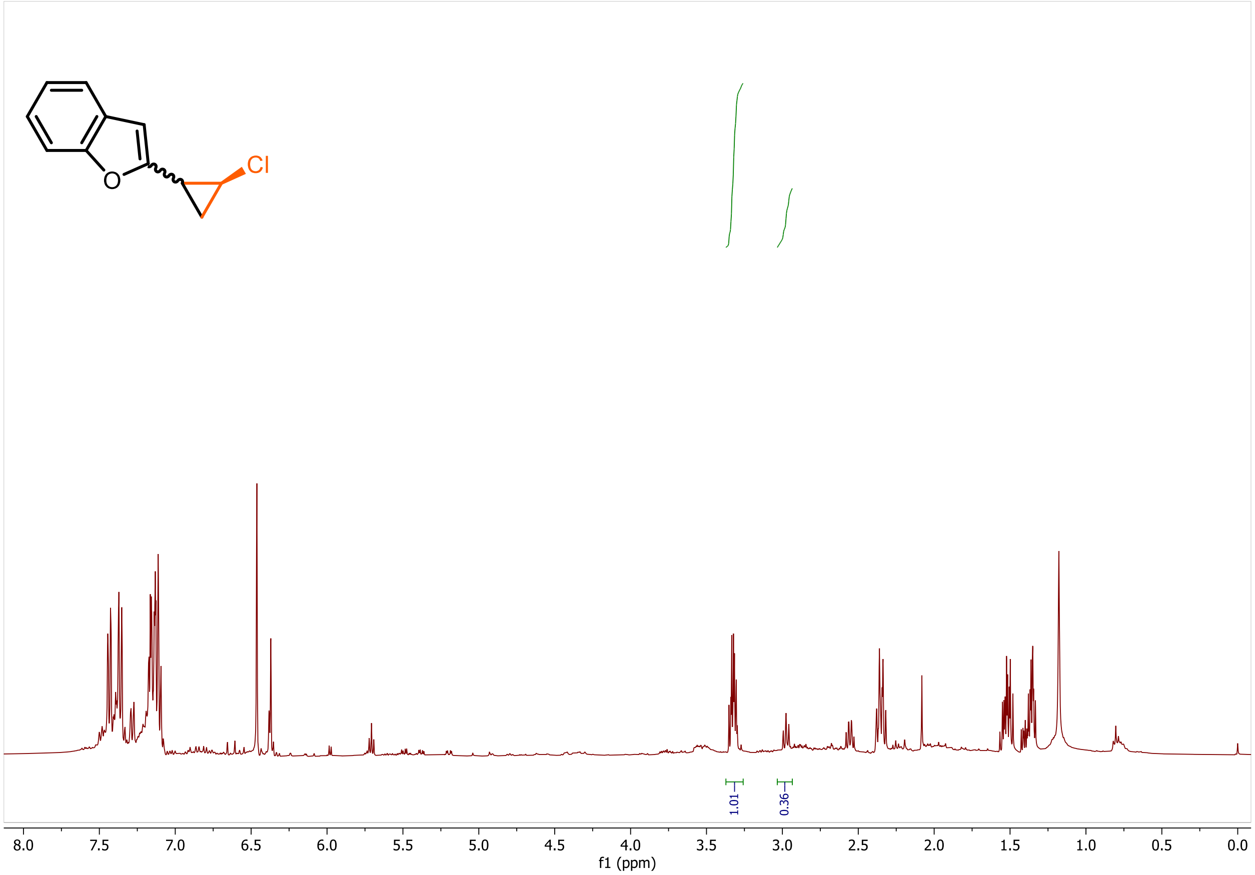

28: ¹H NMR (800 MHz, $CDCl_3$)

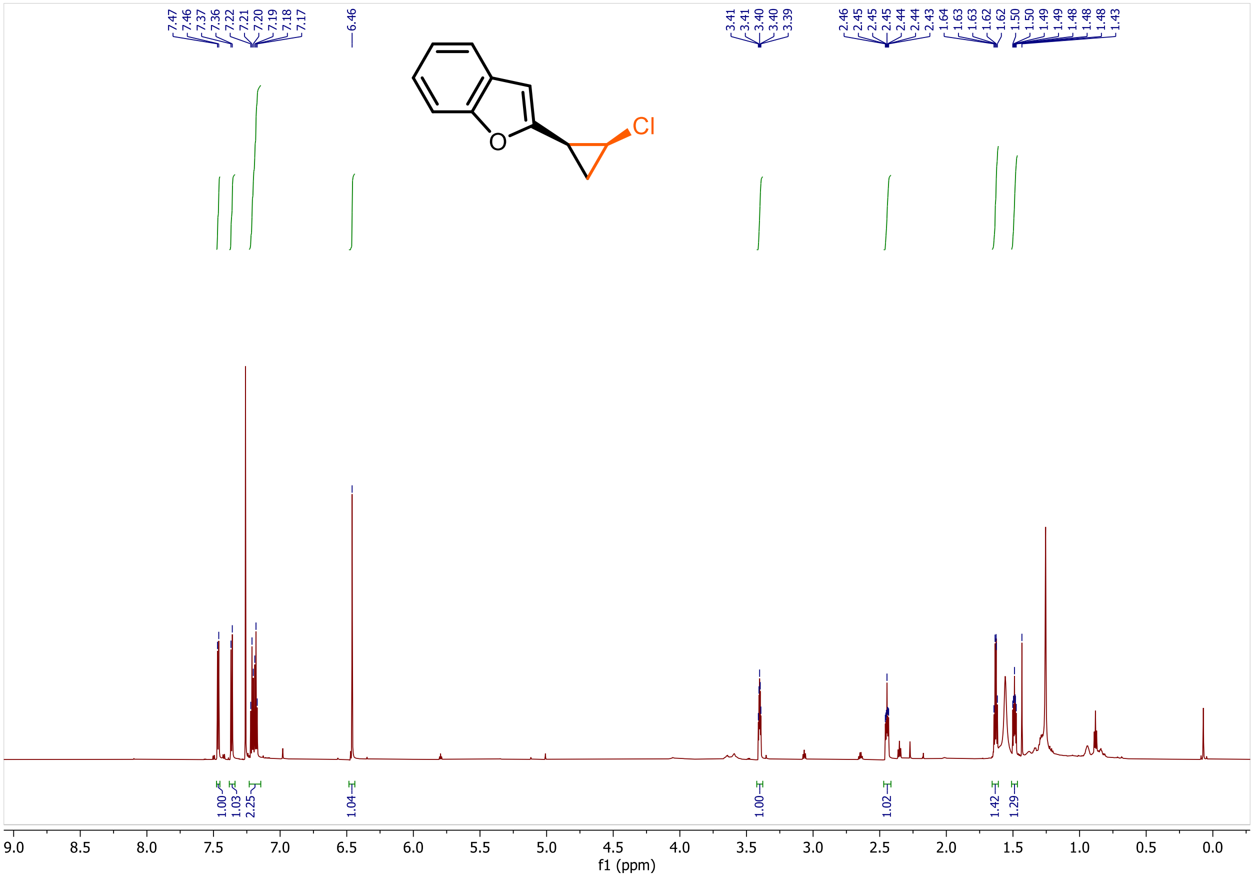

28: ^{13}C NMR (201 MHz, $CDCl_3$)

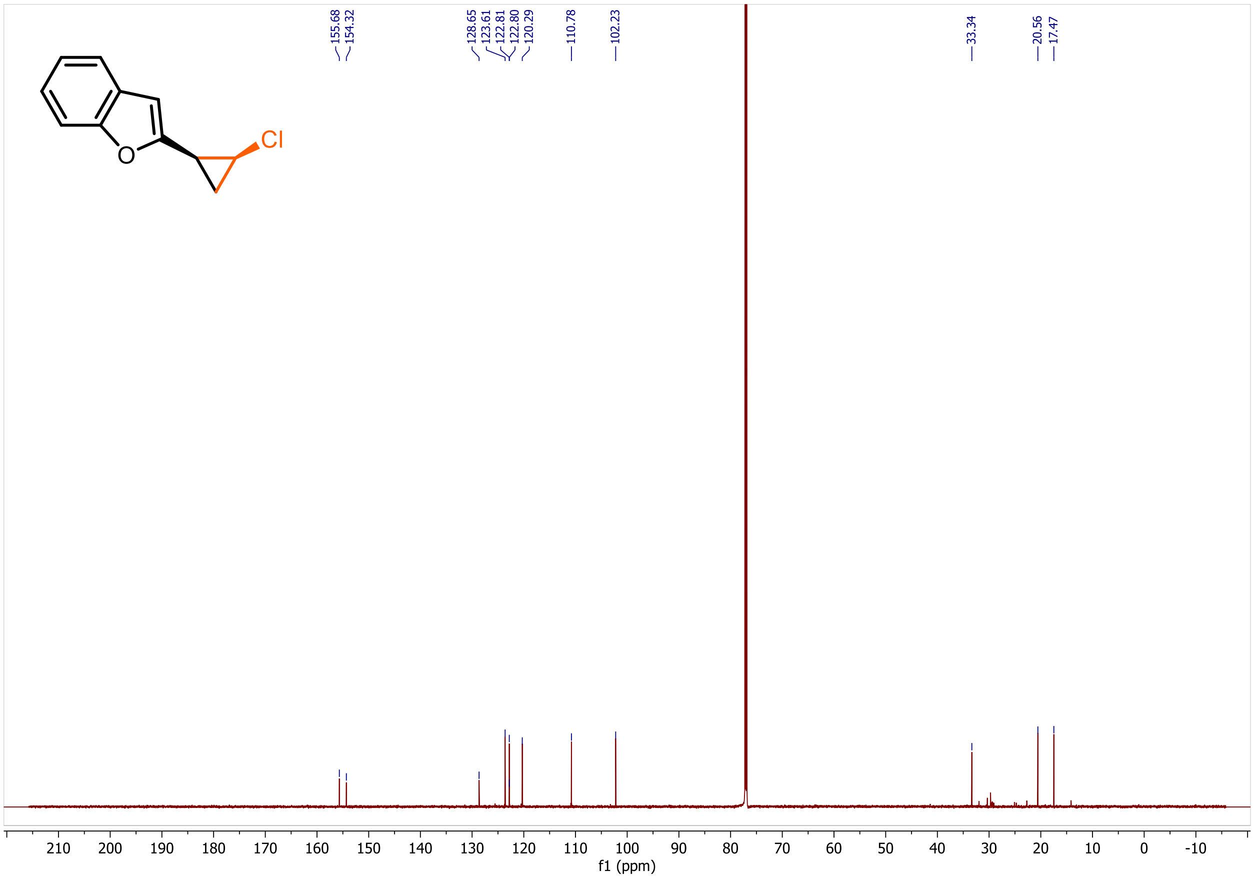

29: Crude ^{1}H NMR (400 MHz, $CDCl_3$)

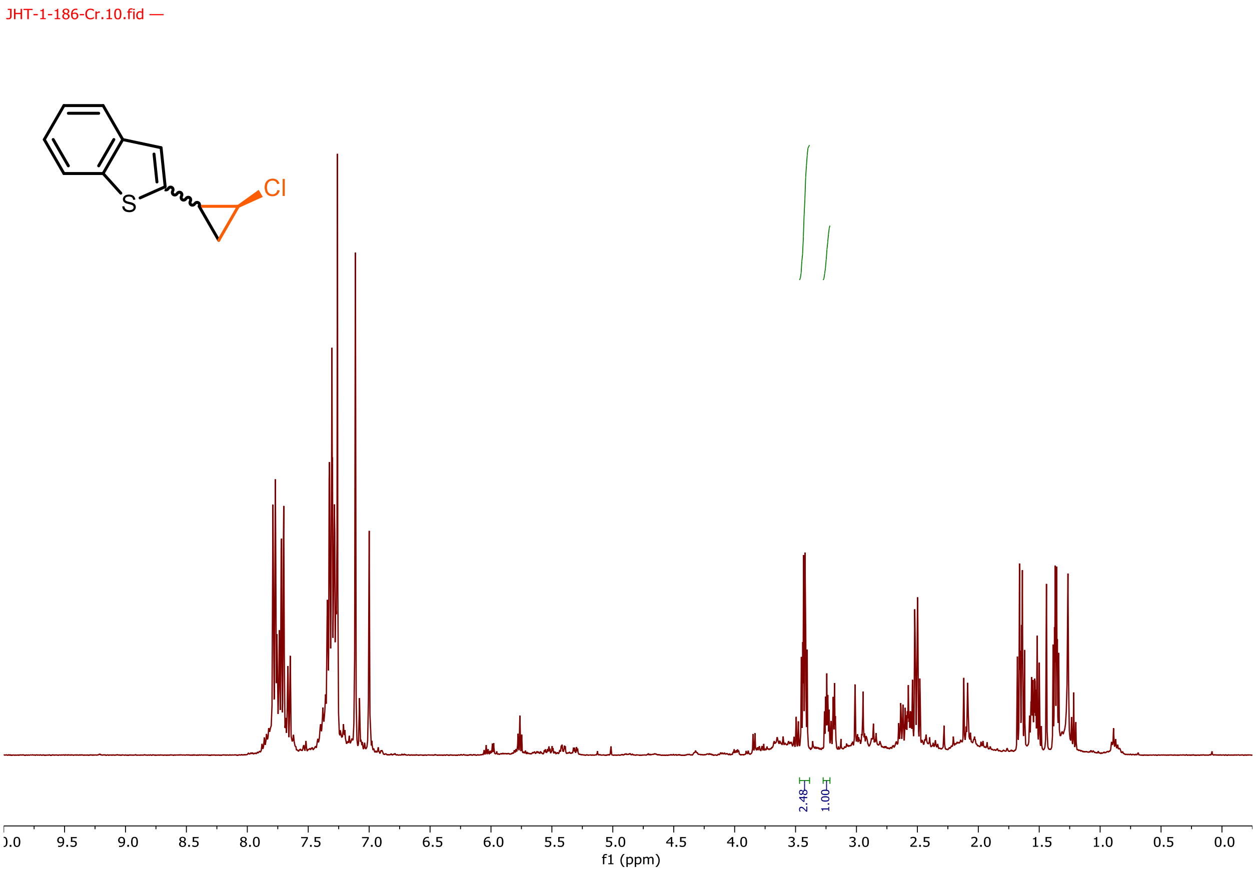

29: ^{1}H NMR (800 MHz, $CDCl_3$)

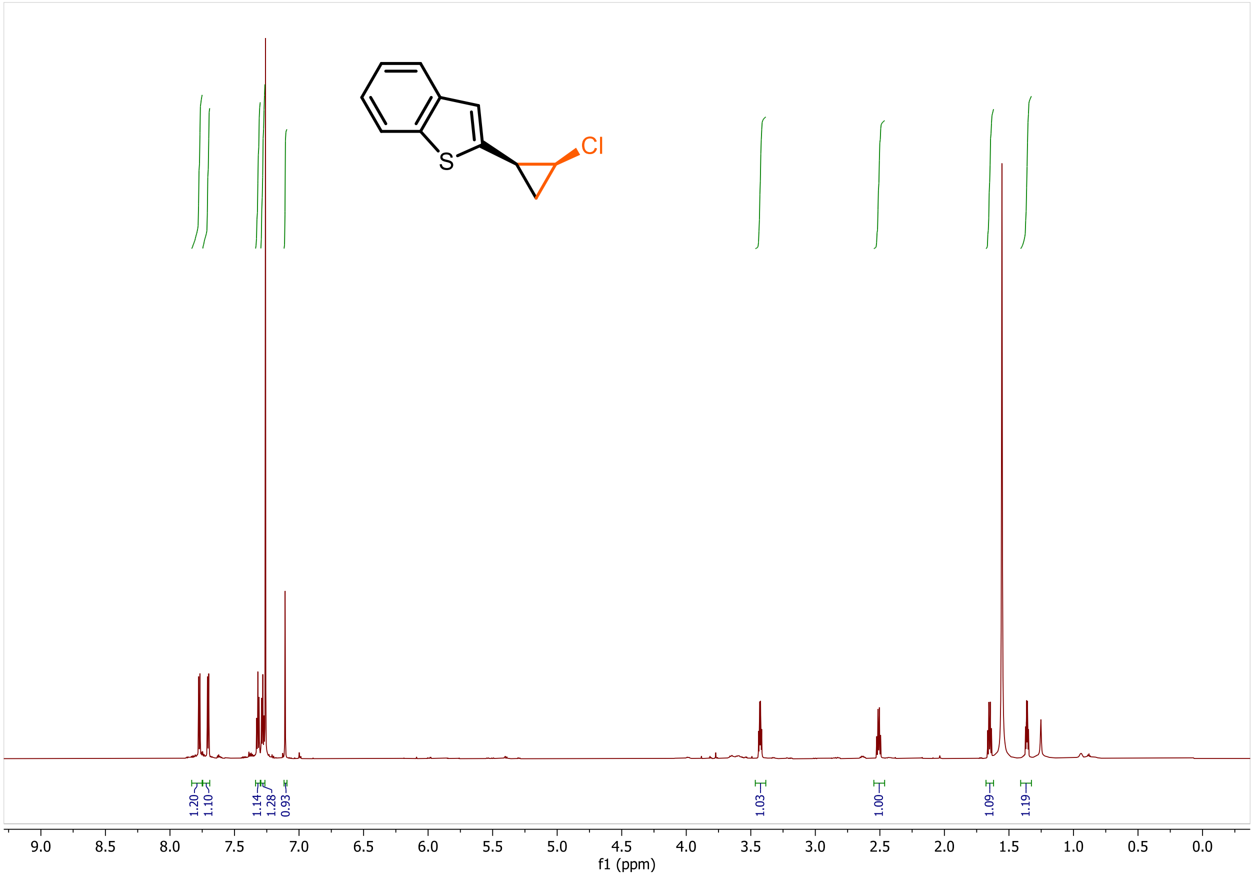

29: ^{13}C NMR (101 MHz, $CDCl_3$)

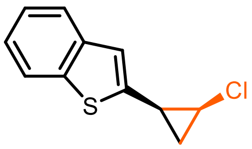

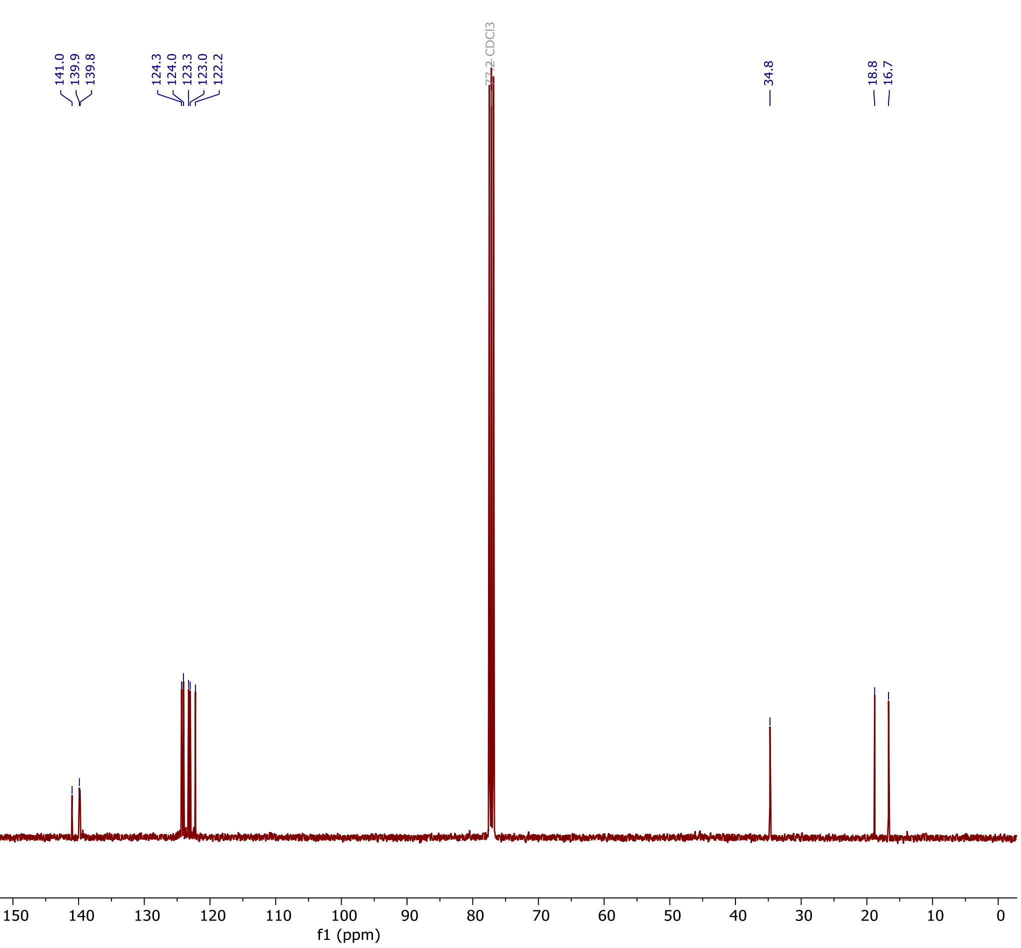

30: Crude ^{1}H NMR (400 MHz, $CDCl_3$)

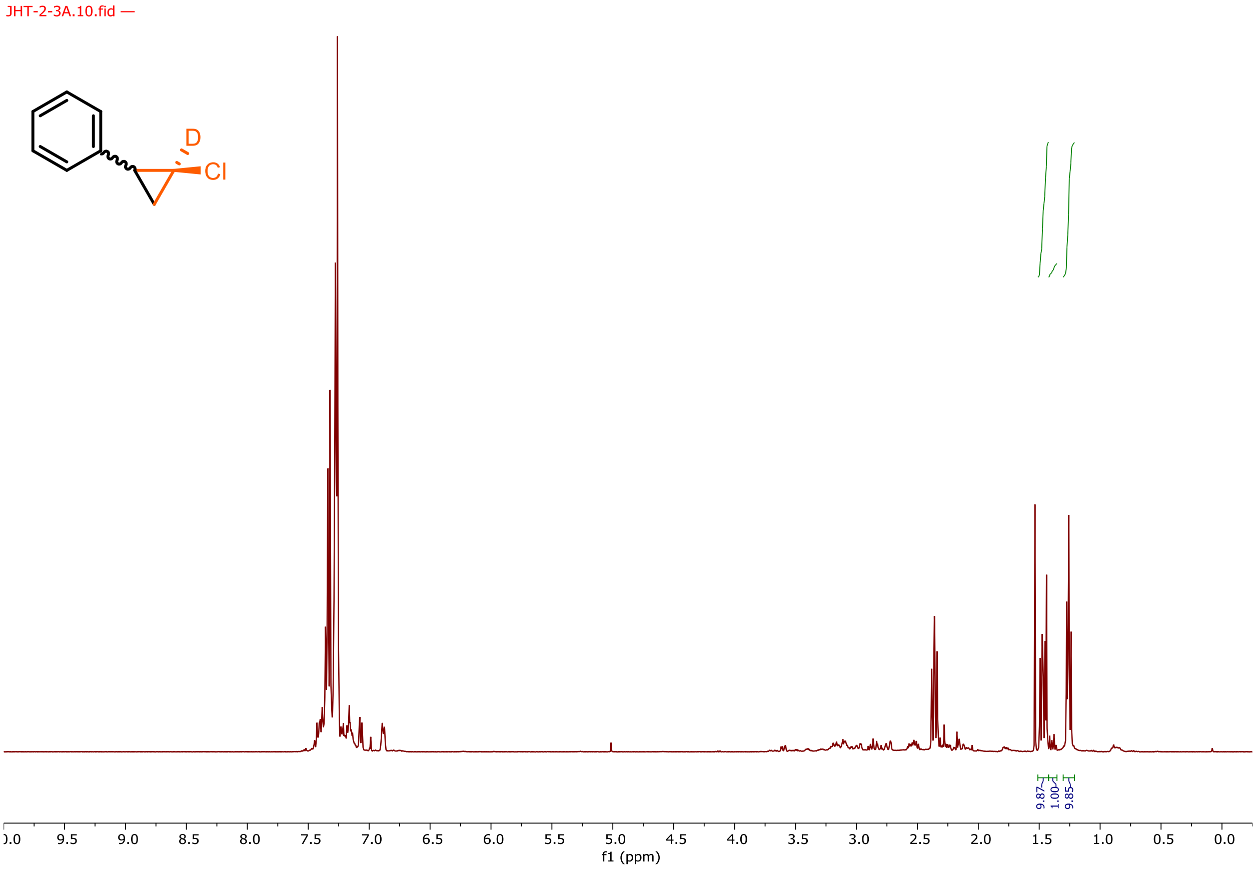

30: ^{1}H NMR (400 MHz, $CDCl_3$)

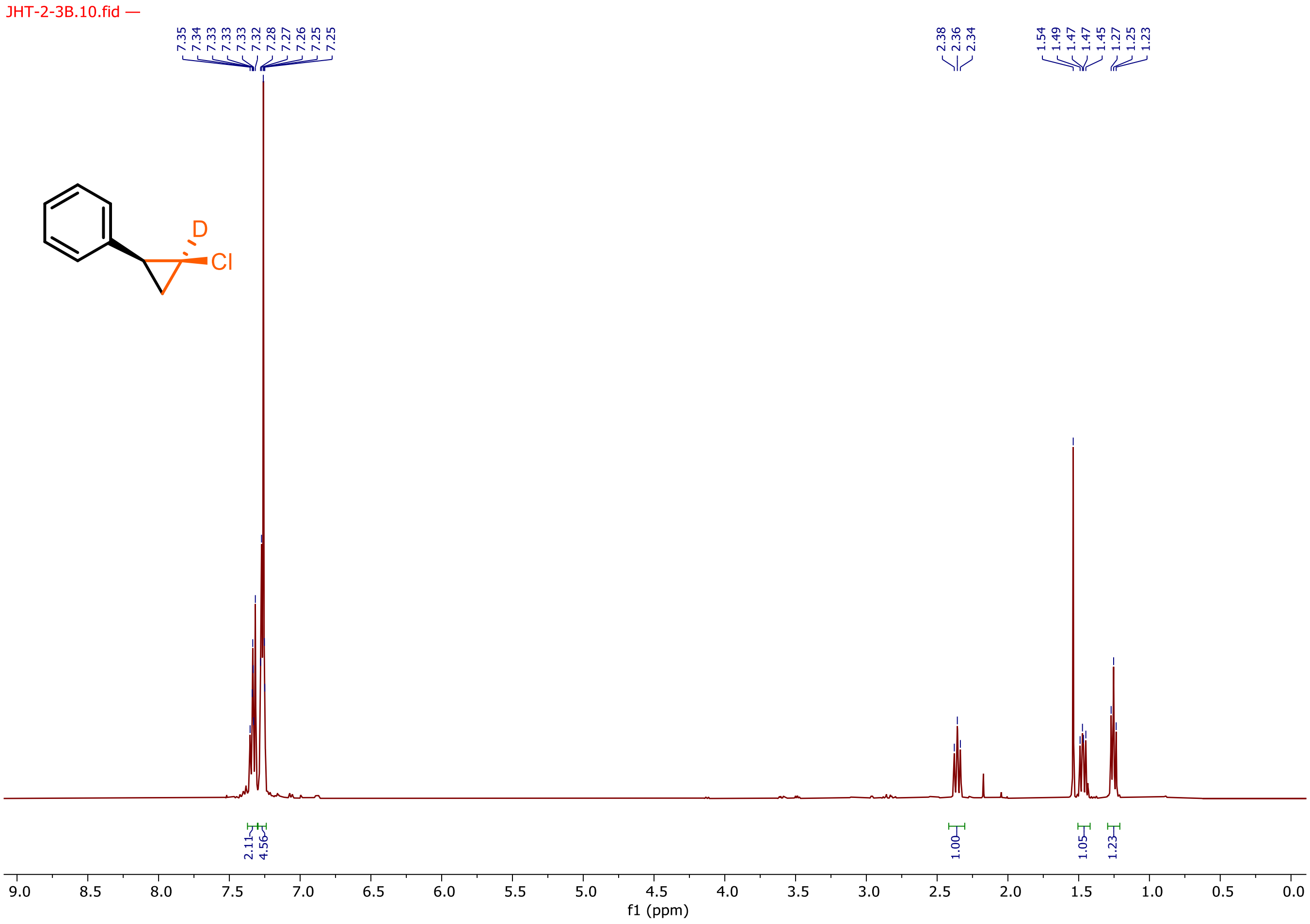

30: ^{13}C NMR (101 MHz, $CDCl_3$)

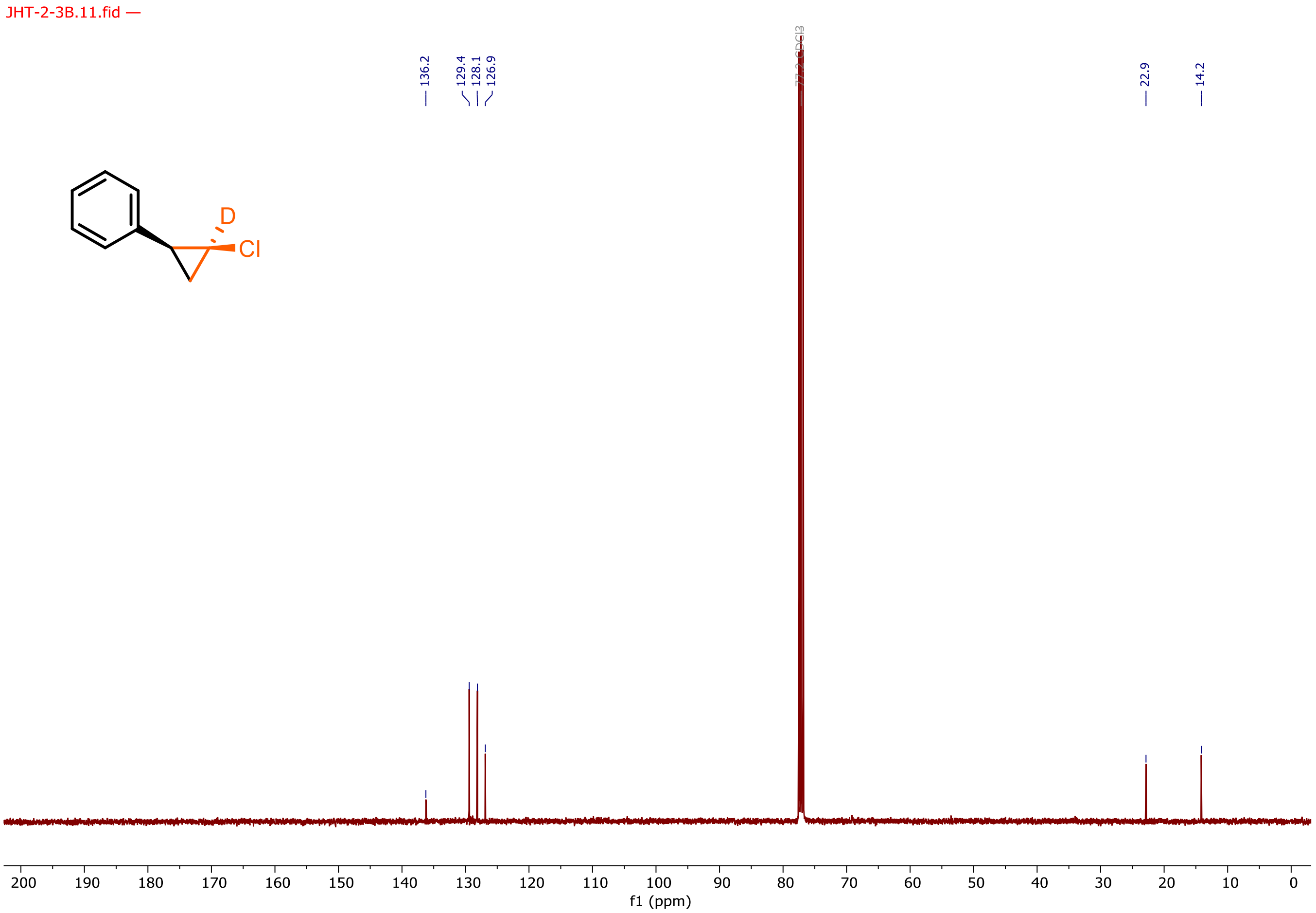

31: Crude ^{1}H NMR (400 MHz, $CDCl_3$)

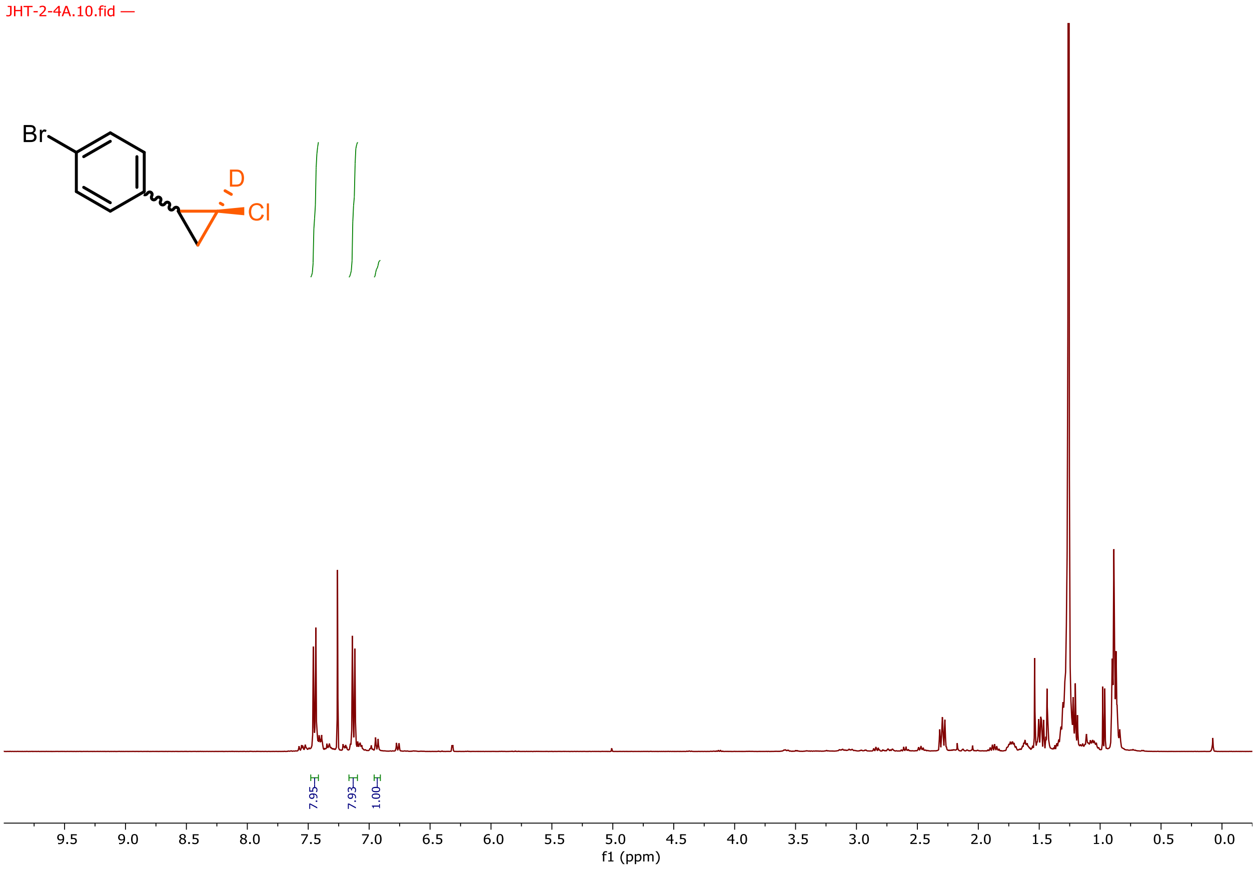

31: ^{1}H NMR (400 MHz, $CDCl_3$)

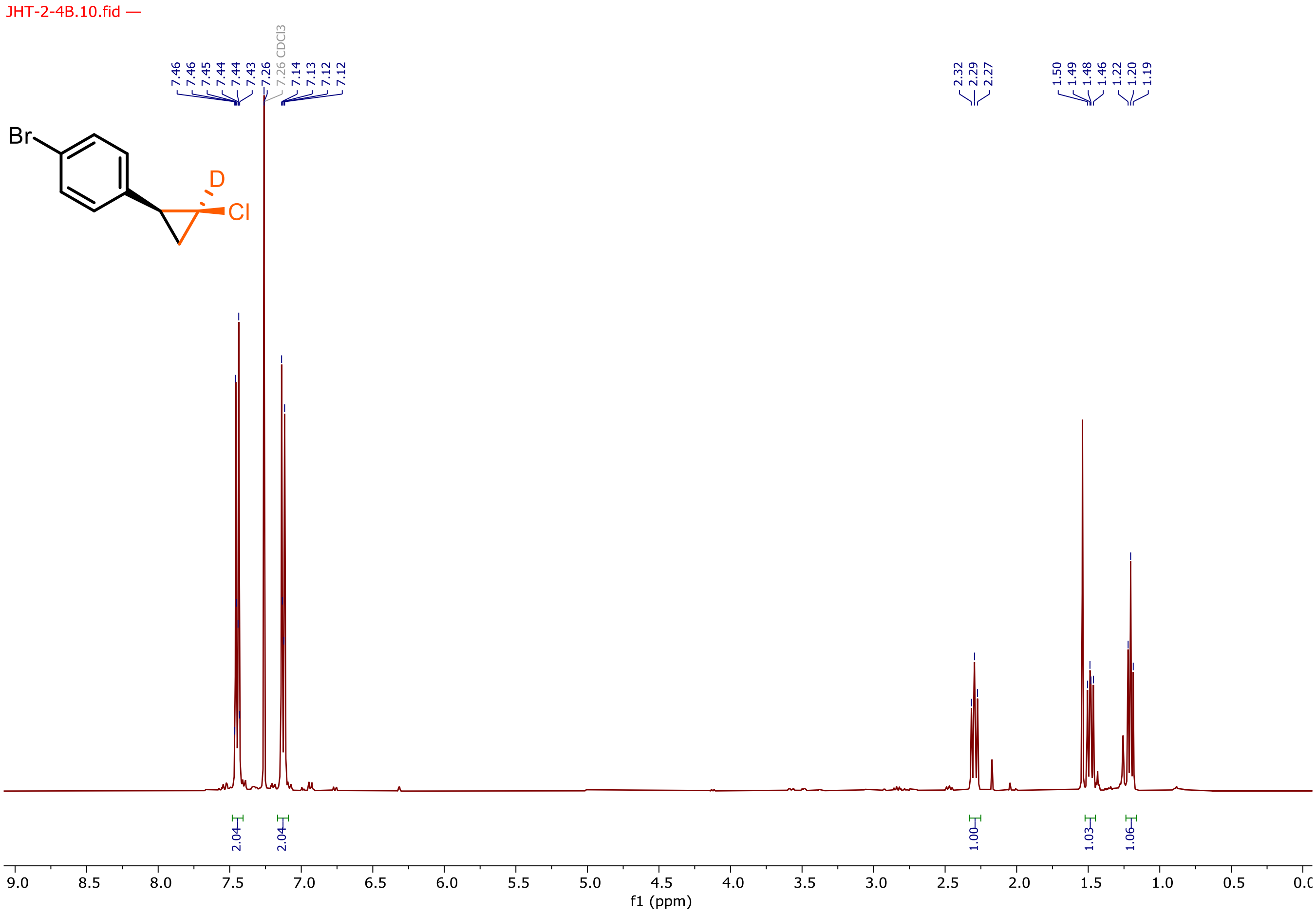

31: ^{13}C NMR (101 MHz, $CDCl_3$)

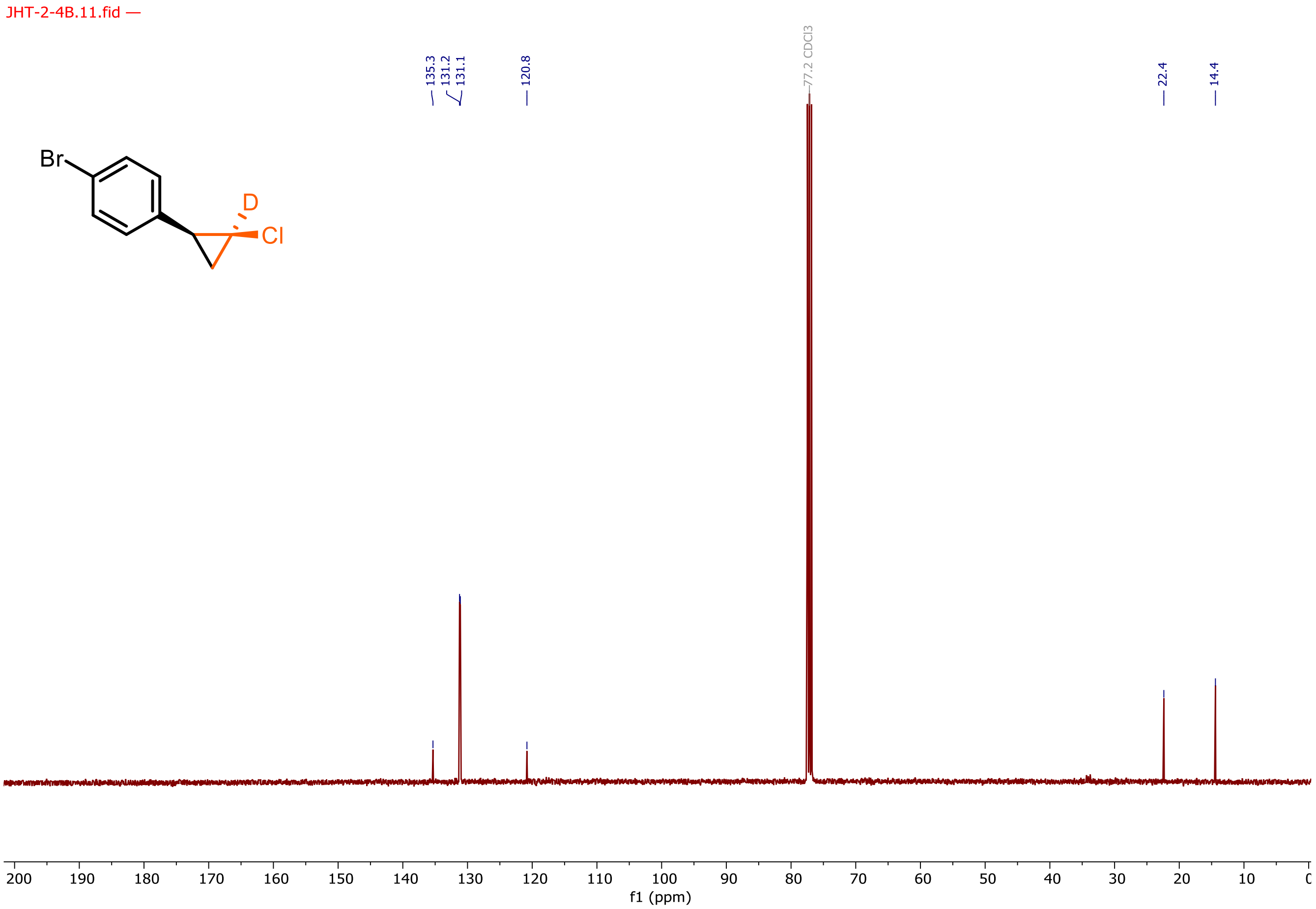

32: Crude ^{1}H NMR (400 MHz, $CDCl_3$)

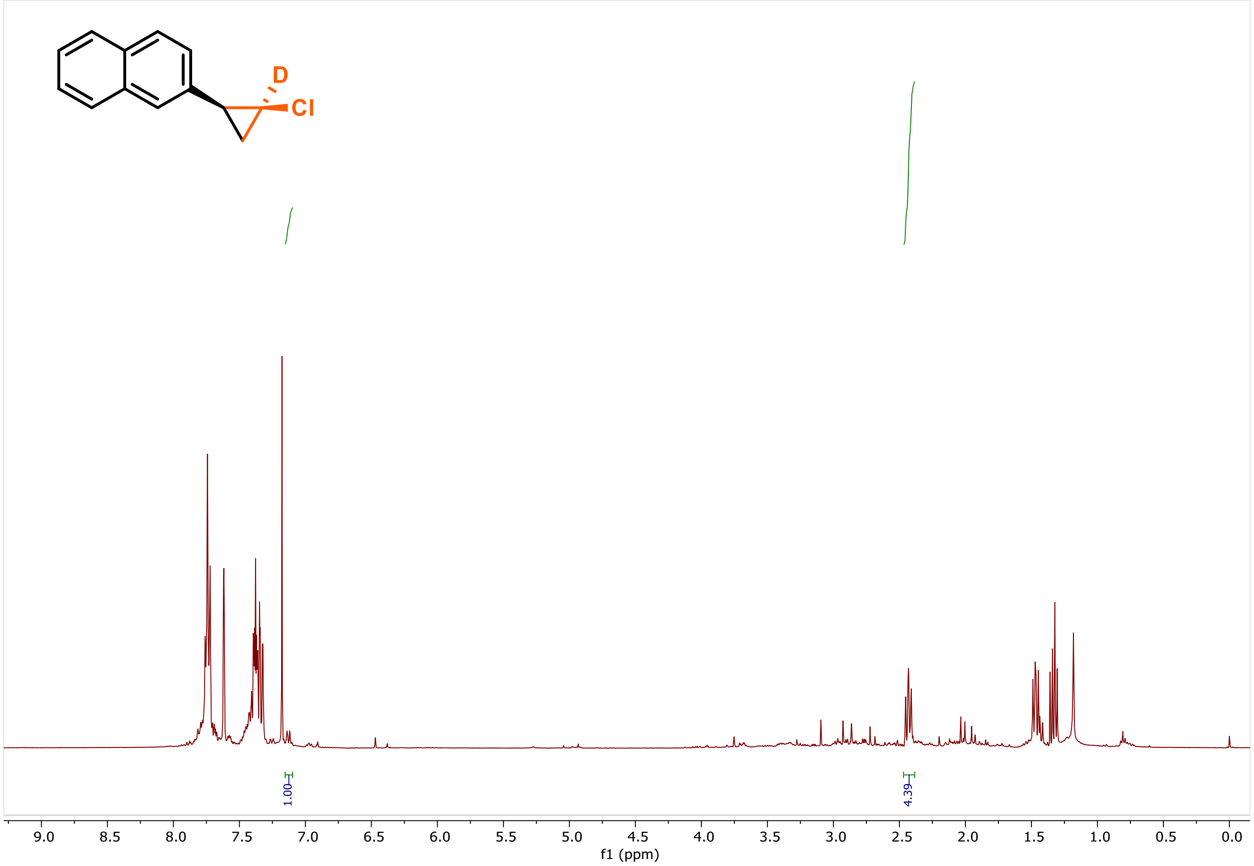

32: ^{1}H NMR (400 MHz, $CDCl_3$)

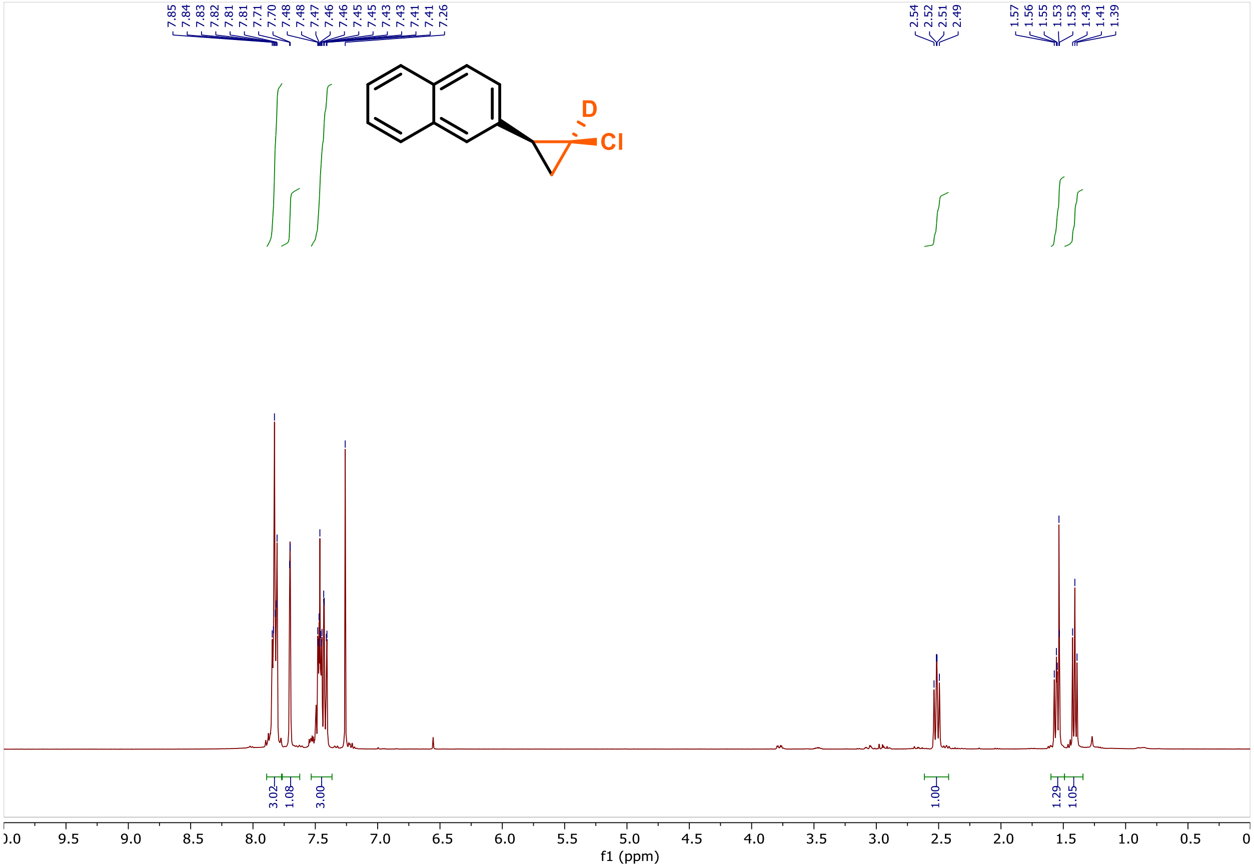

32: ^{13}C NMR (101 MHz, $CDCl_3$)

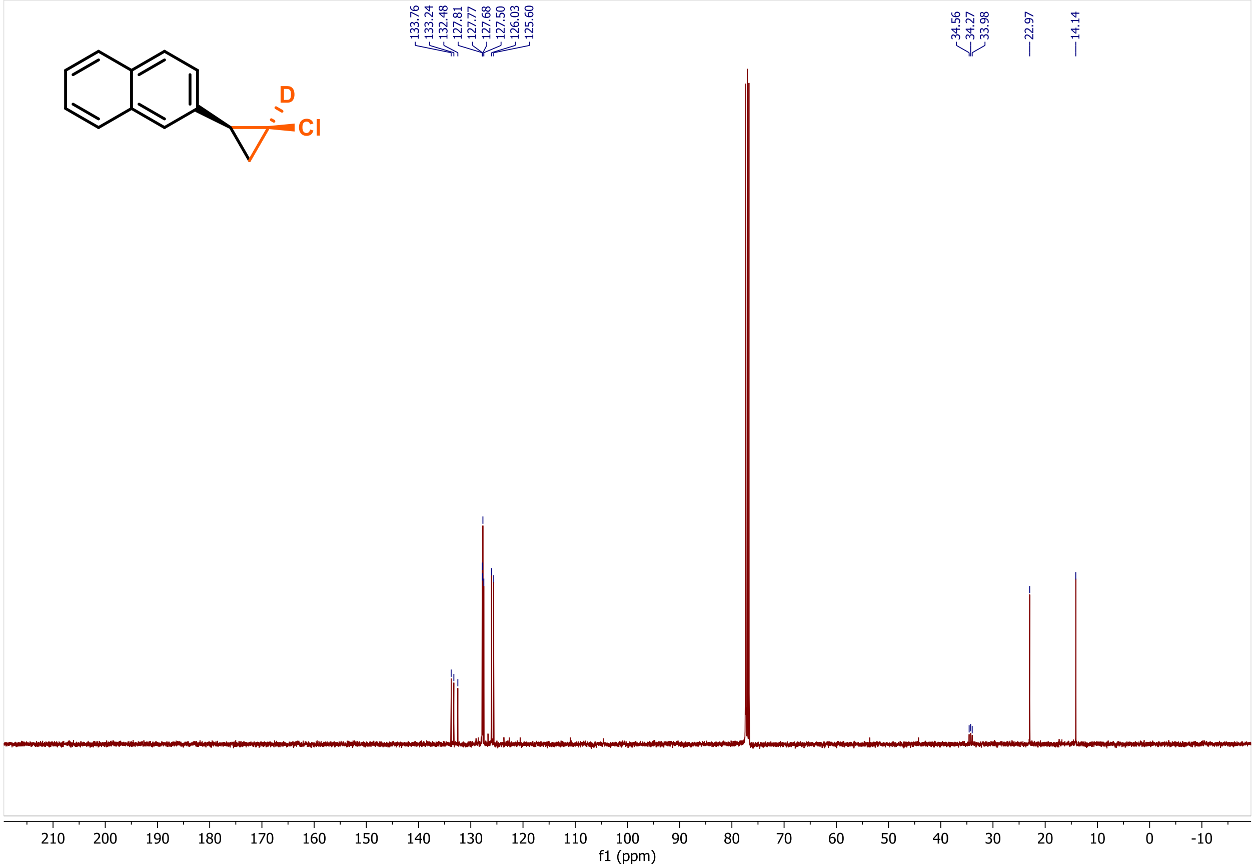

33: ^{1}H NMR (400 MHz, $CDCl_3$)

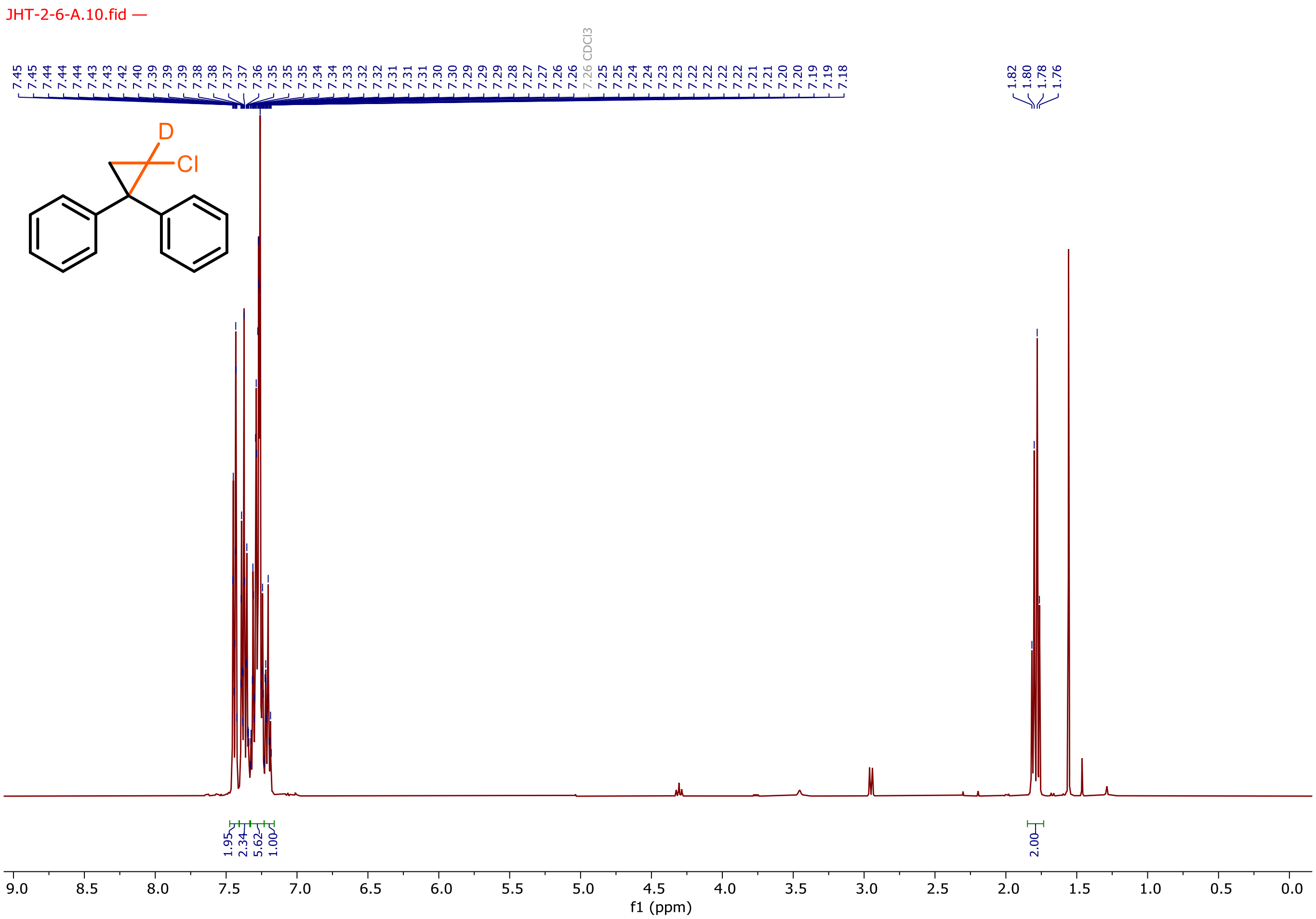

33: ^{13}C NMR (101 MHz, $CDCl_3$)

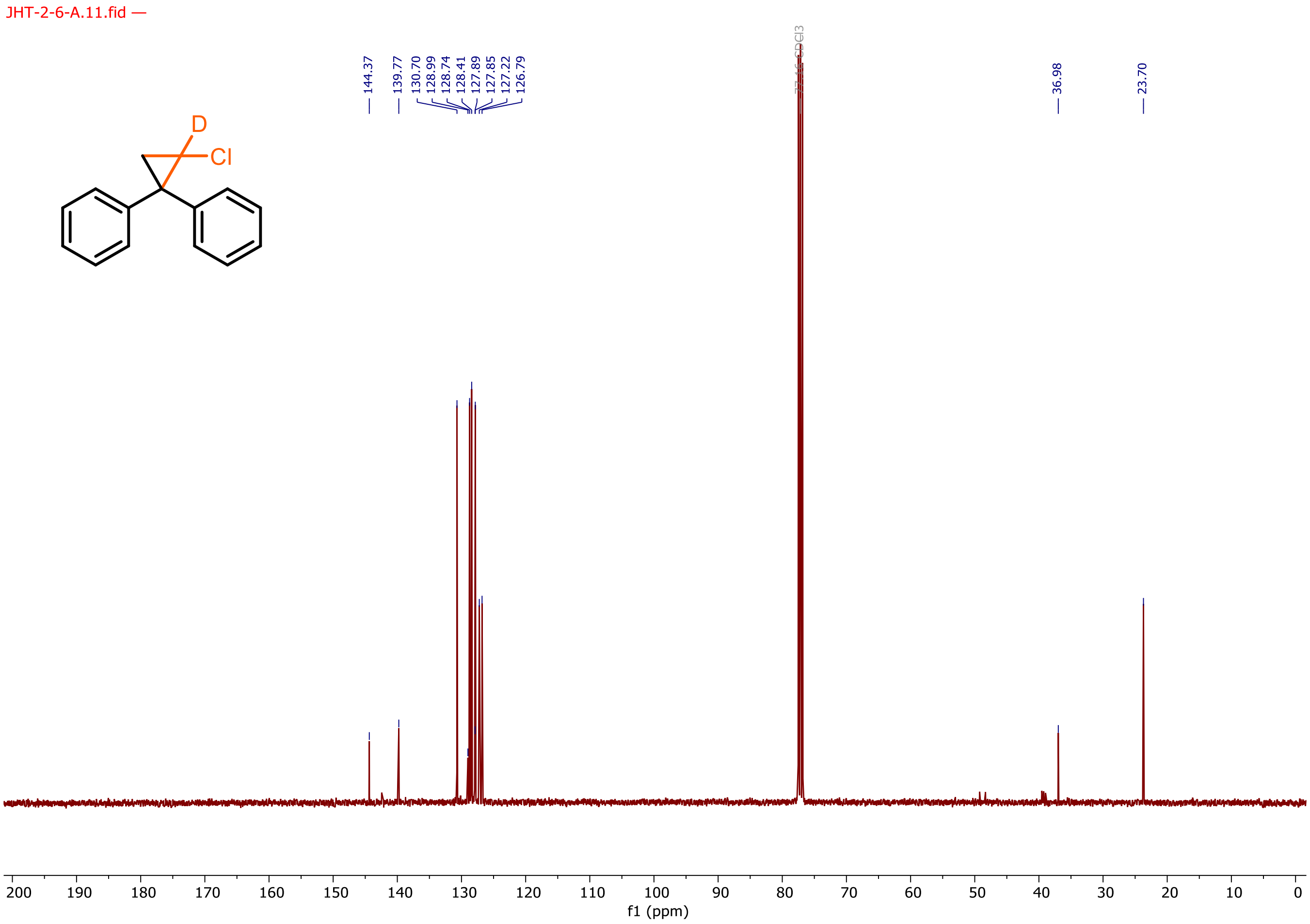

34: Crude ^{1}H NMR (400 MHz, $CDCl_3$)

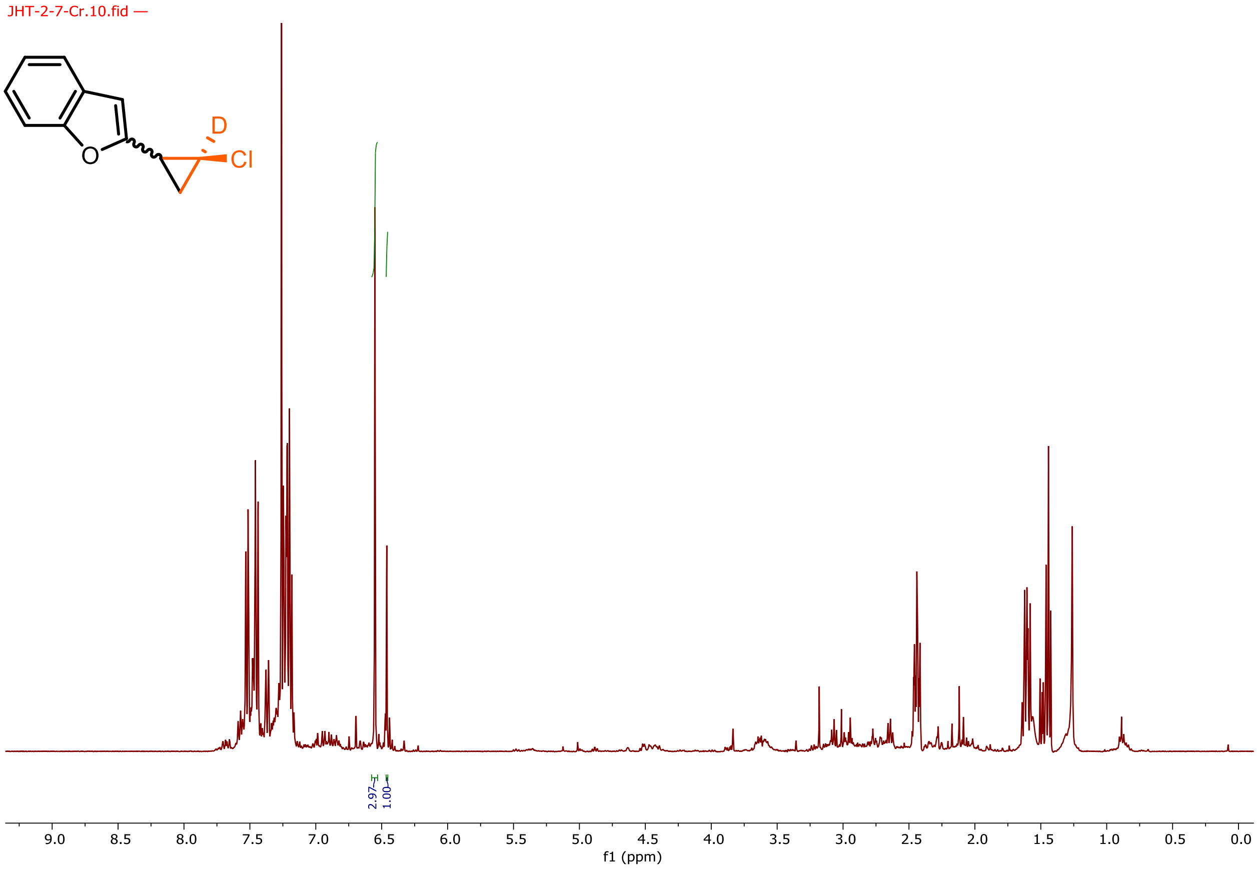

34: ^{1}H NMR (400 MHz, $CDCl_3$)

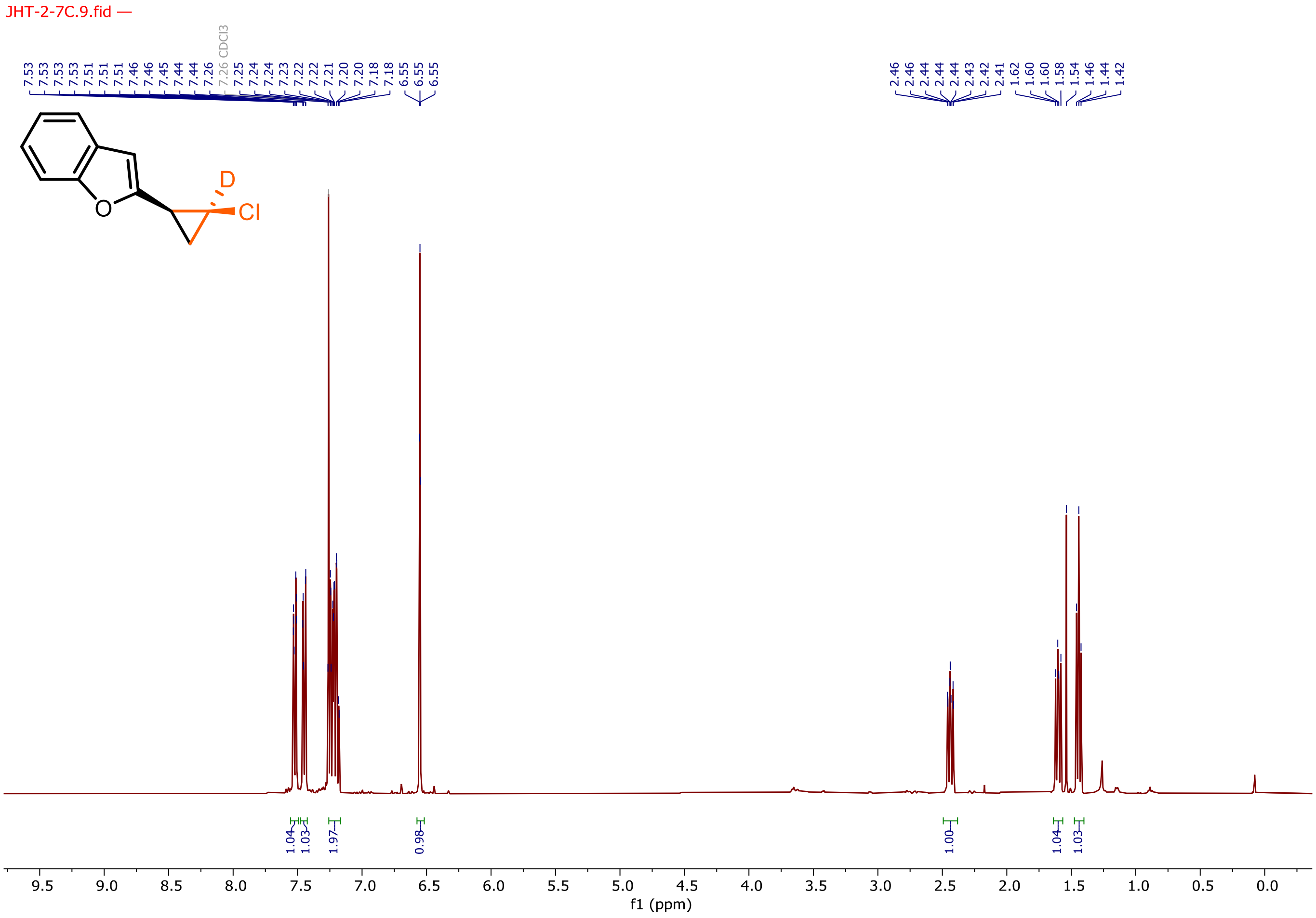

34: ^{13}C NMR (101 MHz, $CDCl_3$)

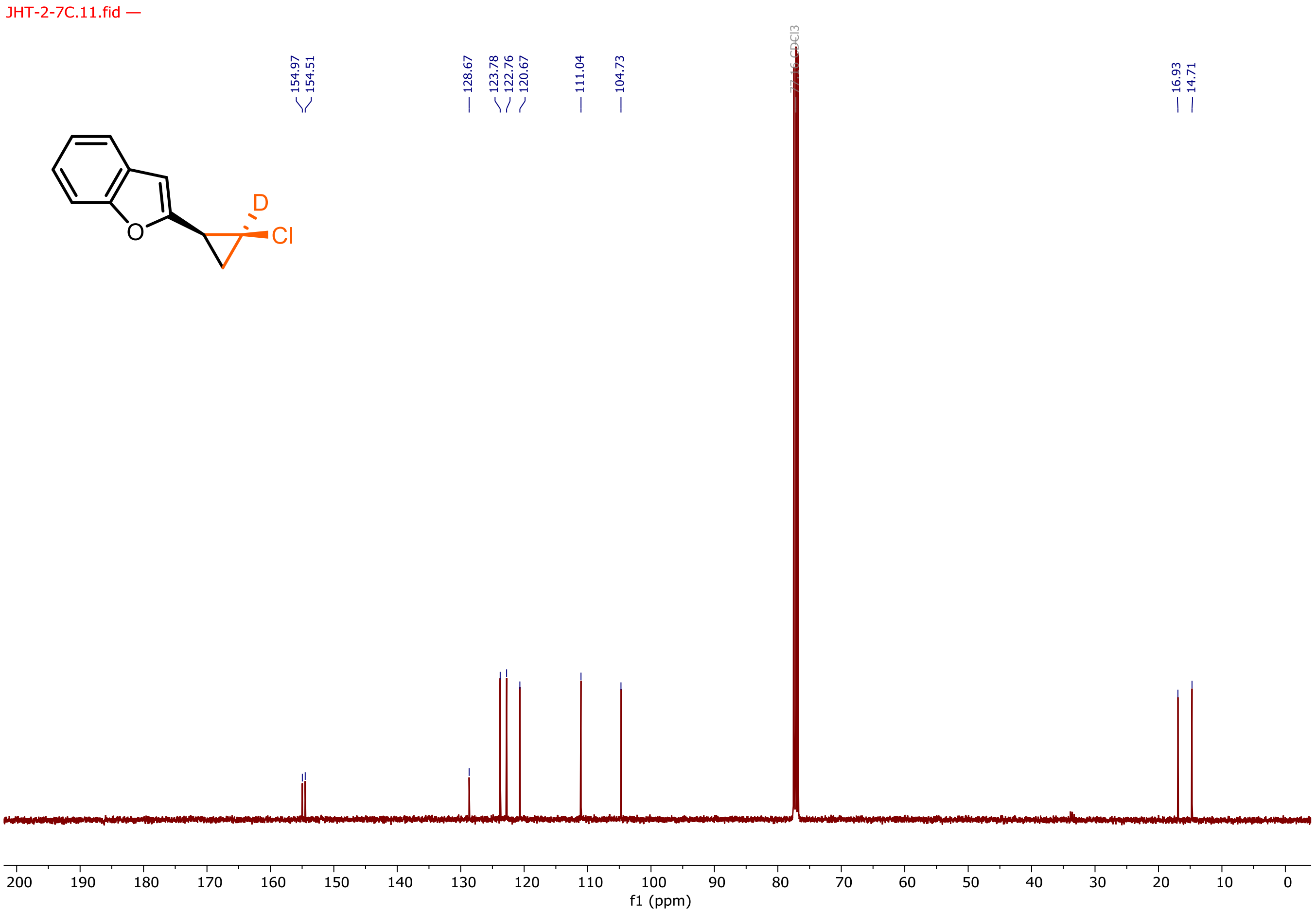

35: Crude ^{1}H NMR (400 MHz, $CDCl_3$)

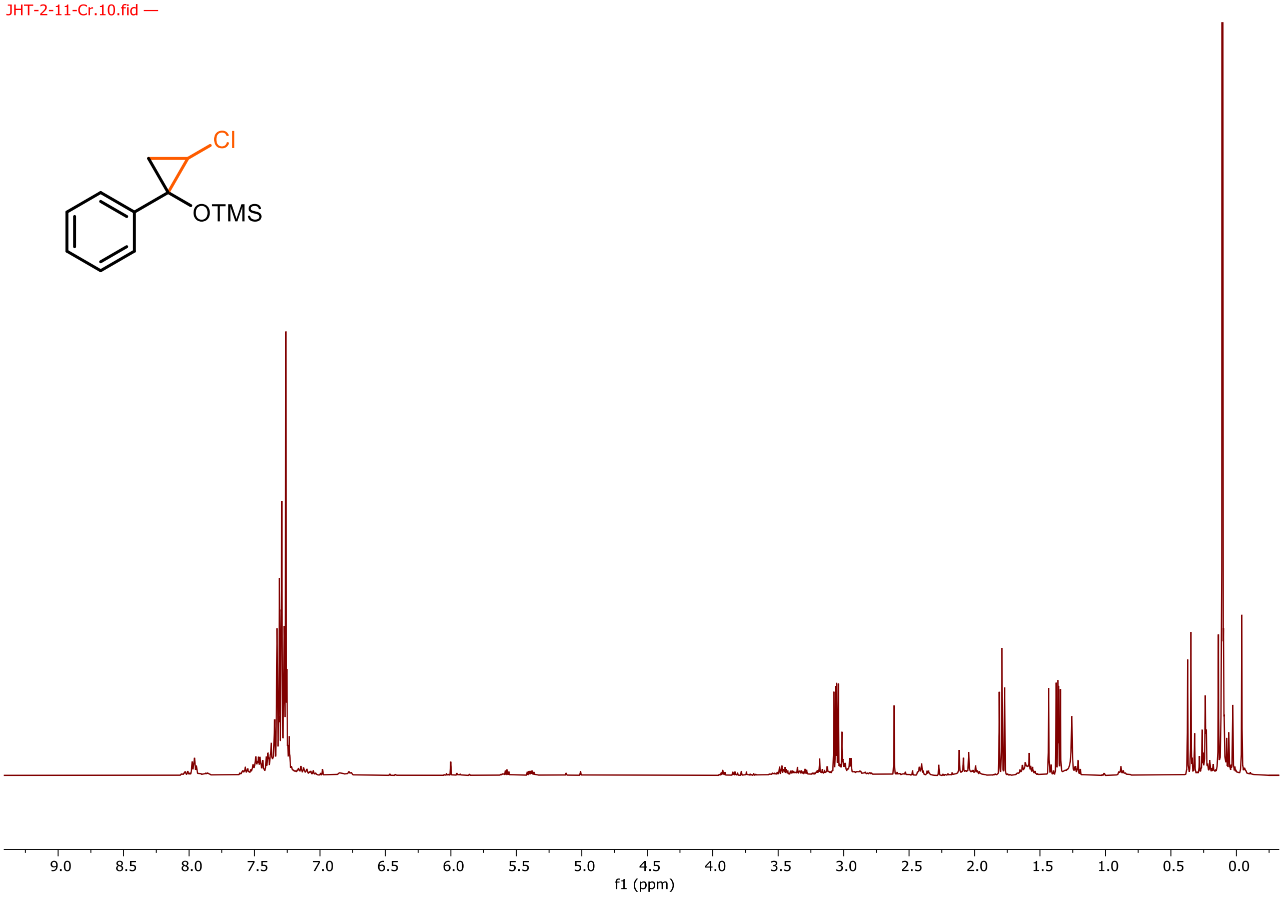

35: ^{1}H NMR (400 MHz, $CDCl_3$)

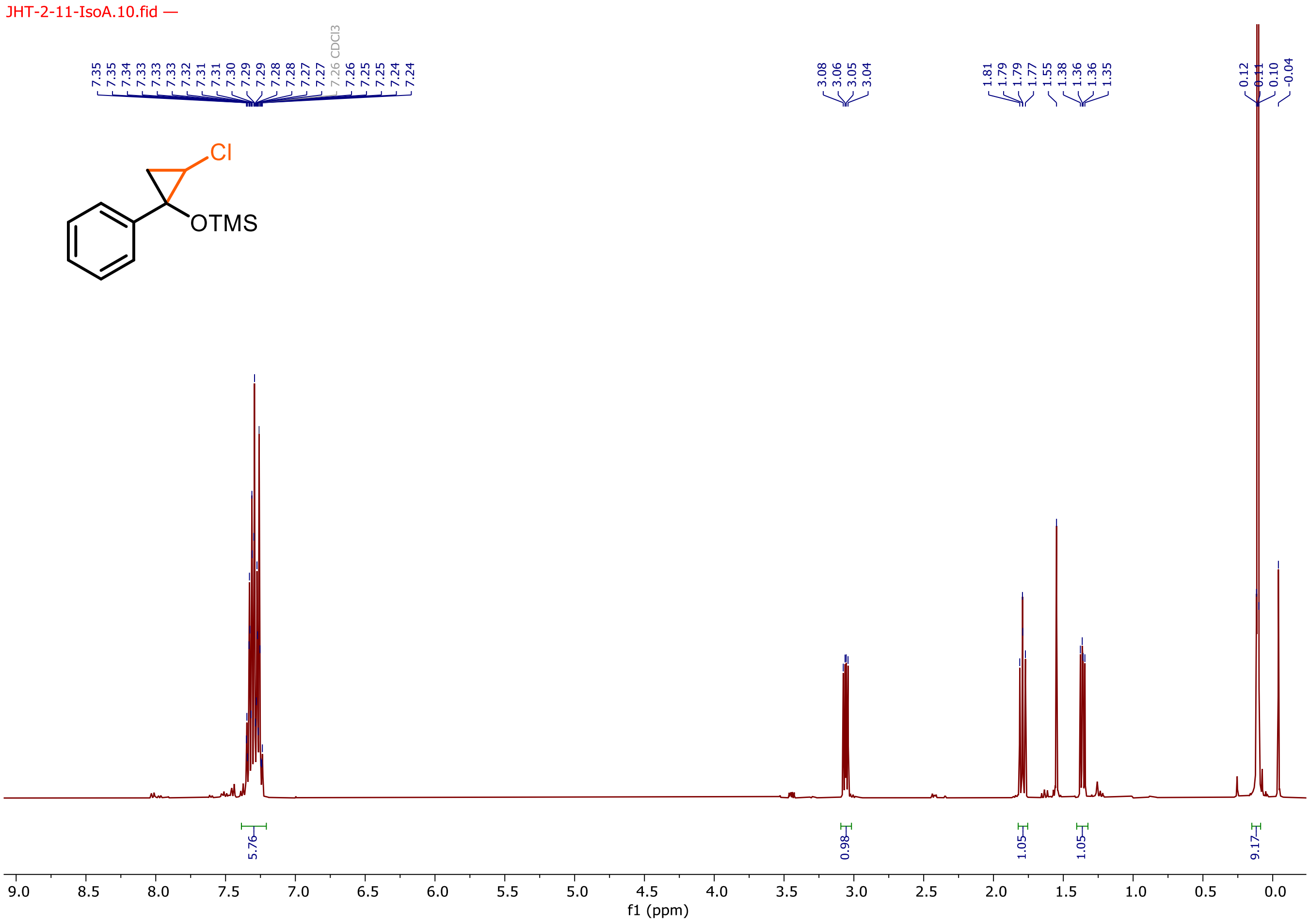

35: ^{13}C NMR (101 MHz, $CDCl_3$)

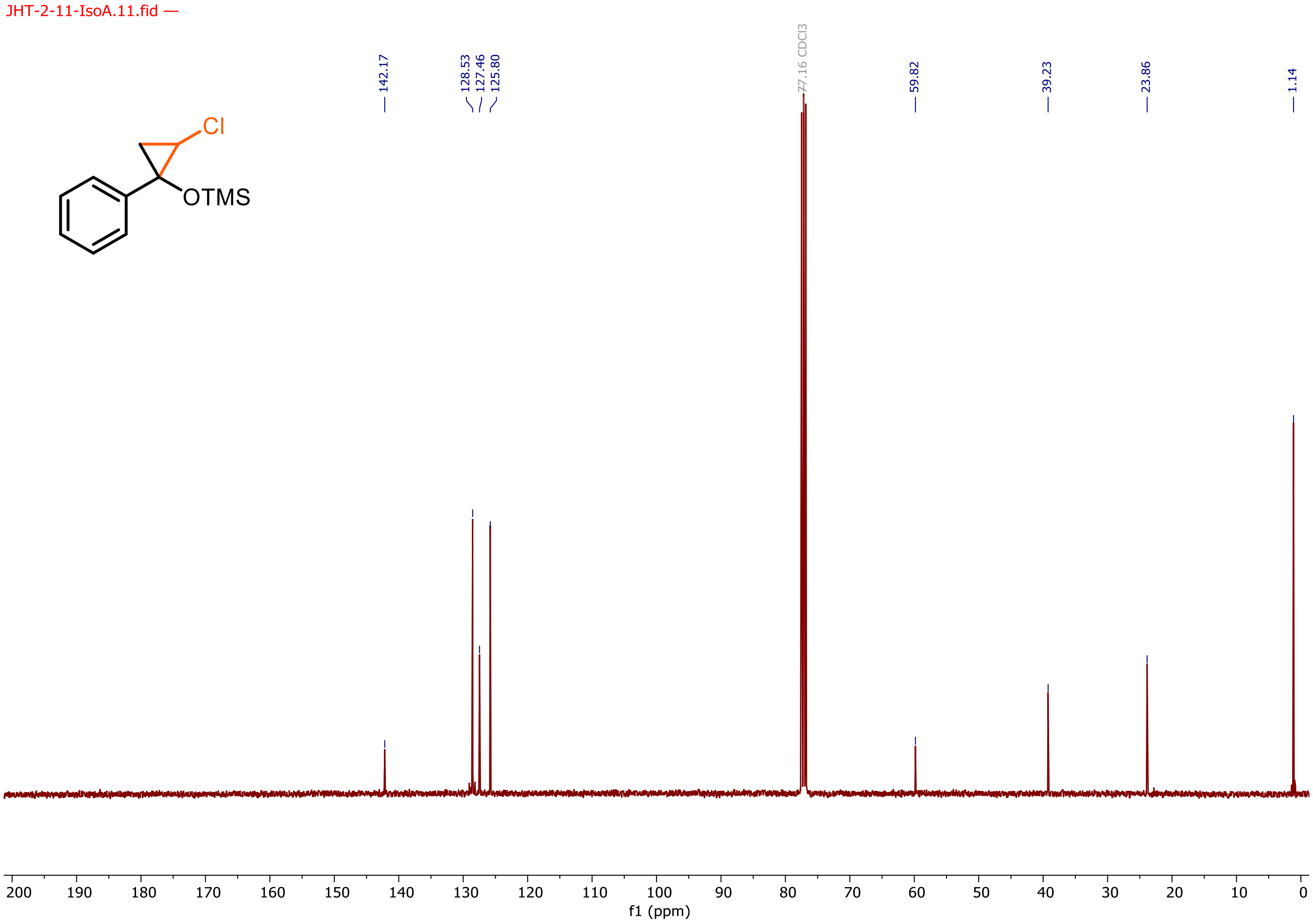

36: Crude ^{1}H NMR (400 MHz, $CDCl_3$)

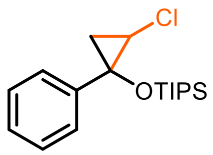

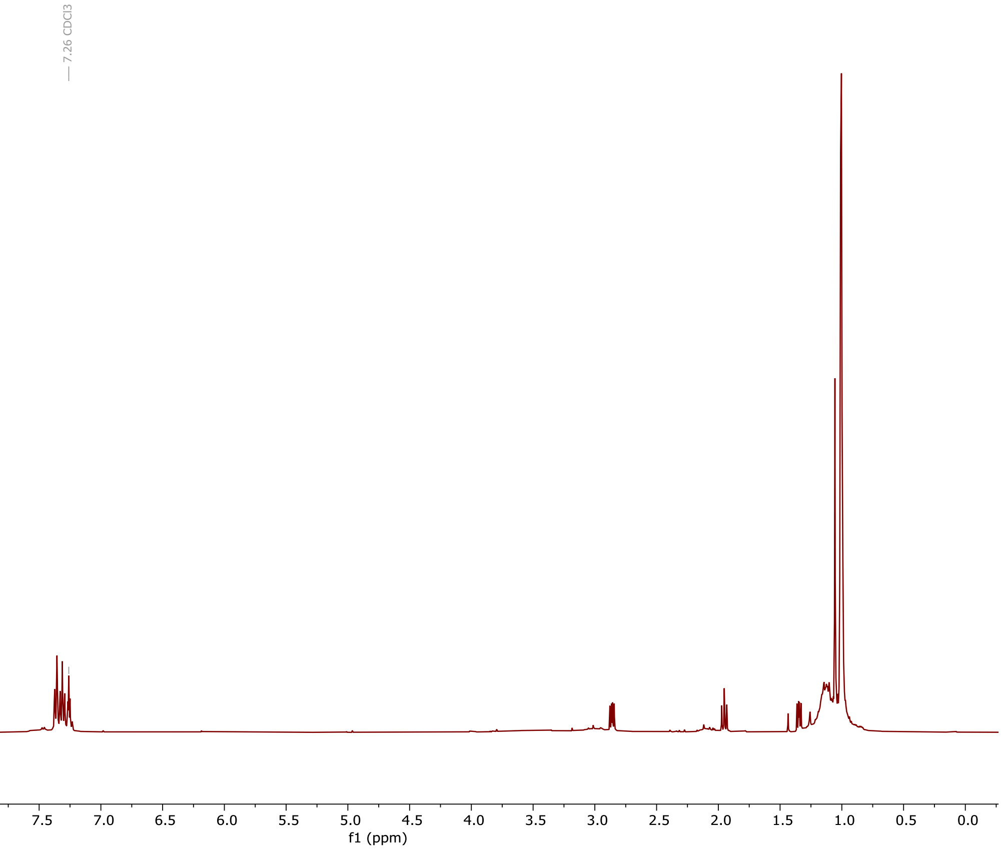

36: ^{1}H NMR (400 MHz, $CDCl_3$)

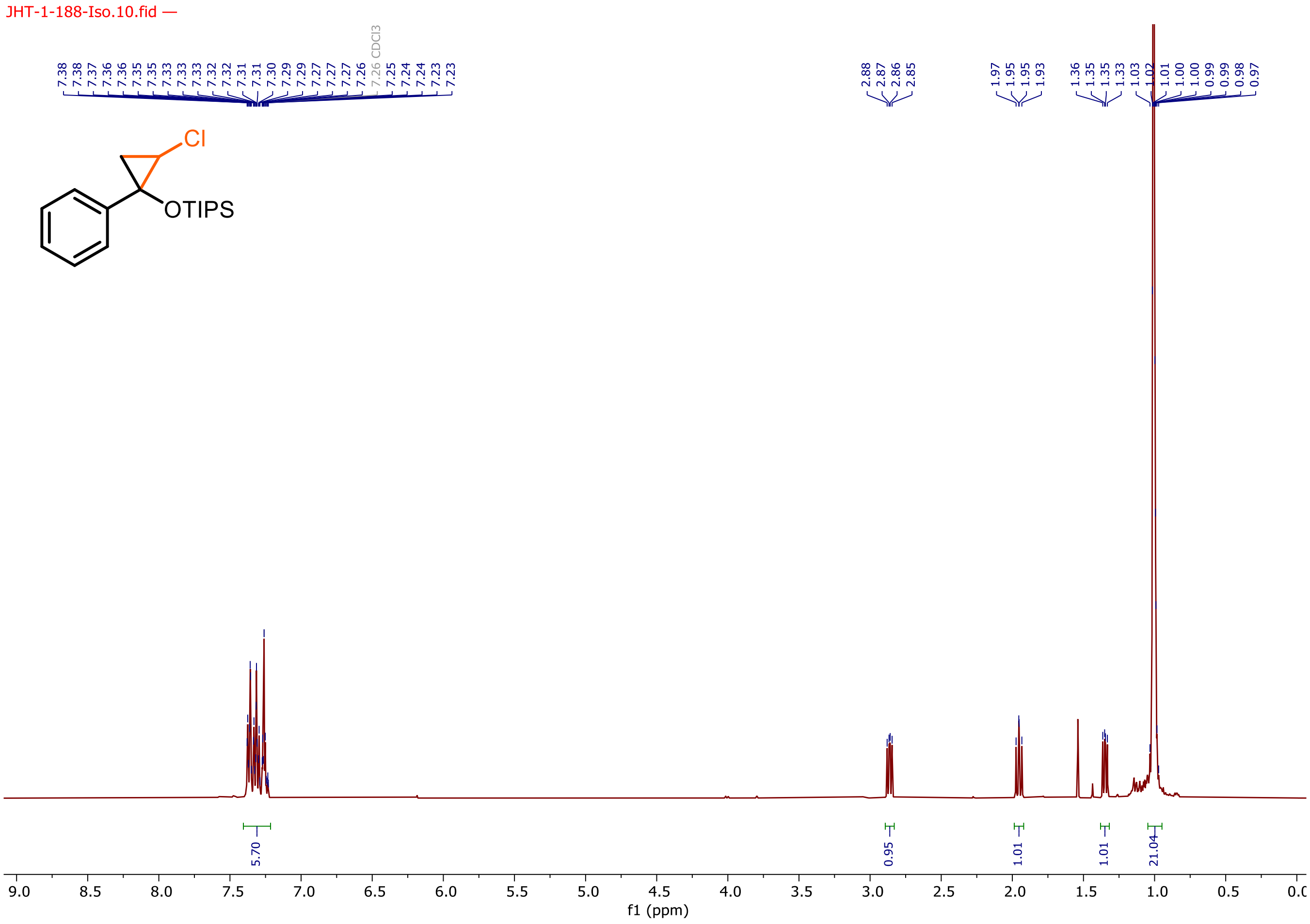

36: ^{13}C NMR (101 MHz, $CDCl_3$)

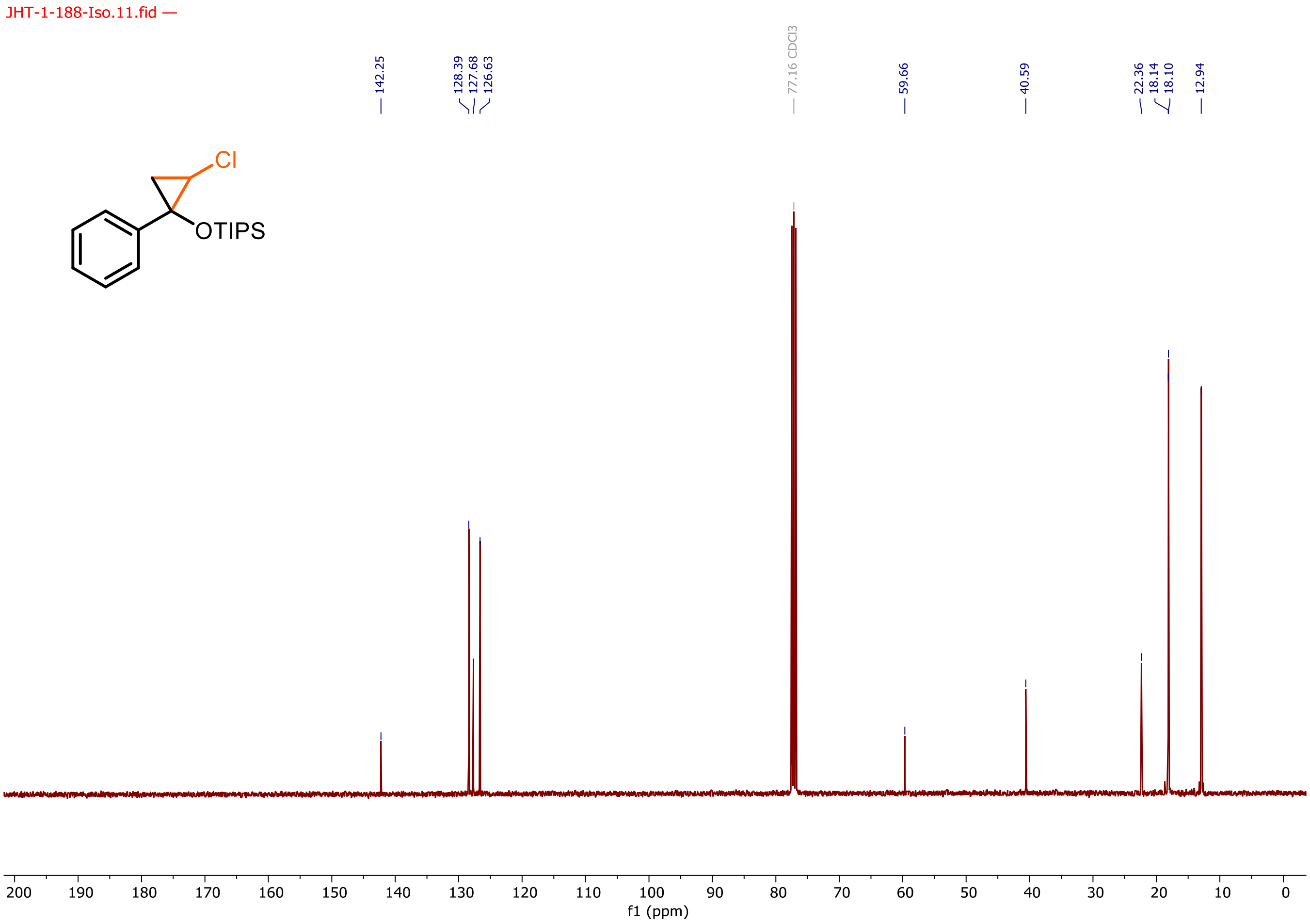

37: Crude ^{1}H NMR (400 MHz, $CDCl_3$)

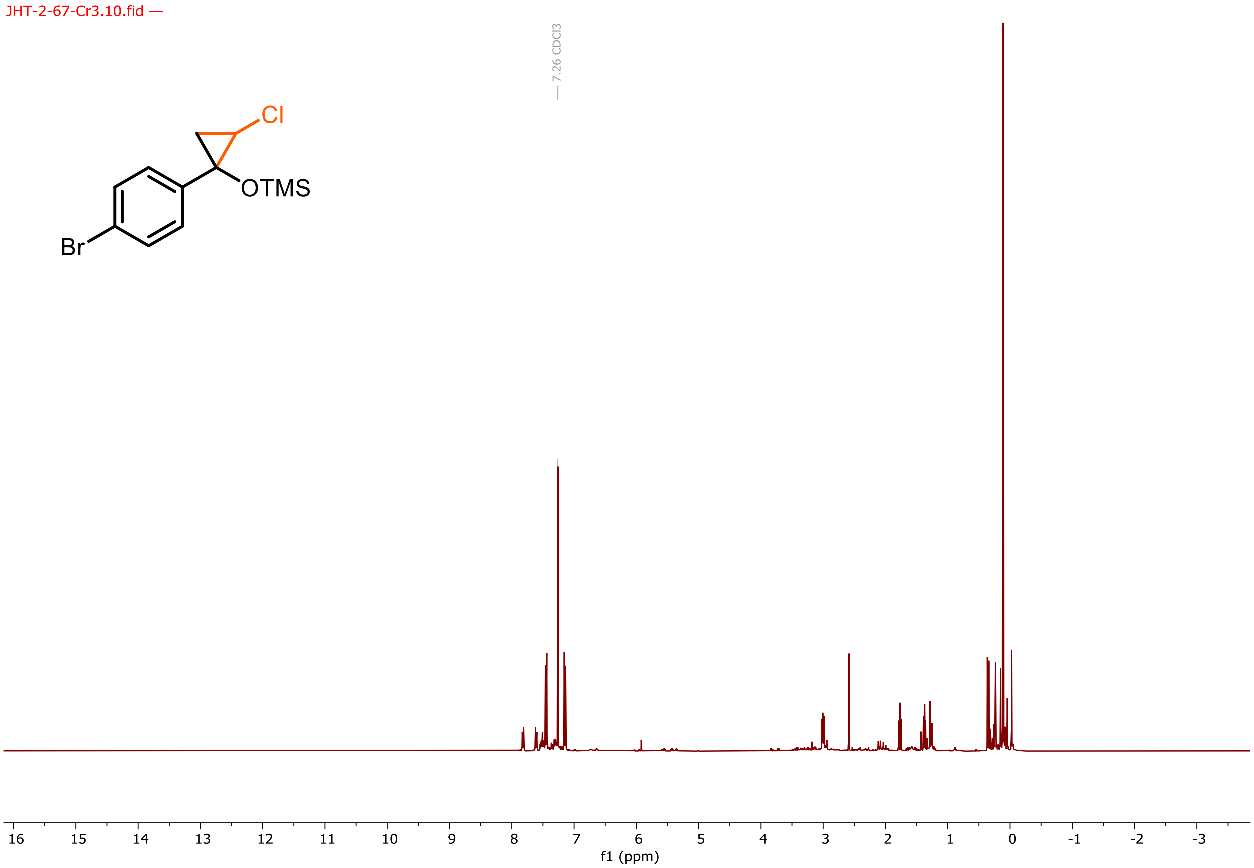

37: ^{1}H NMR (400 MHz, $CDCl_3$)

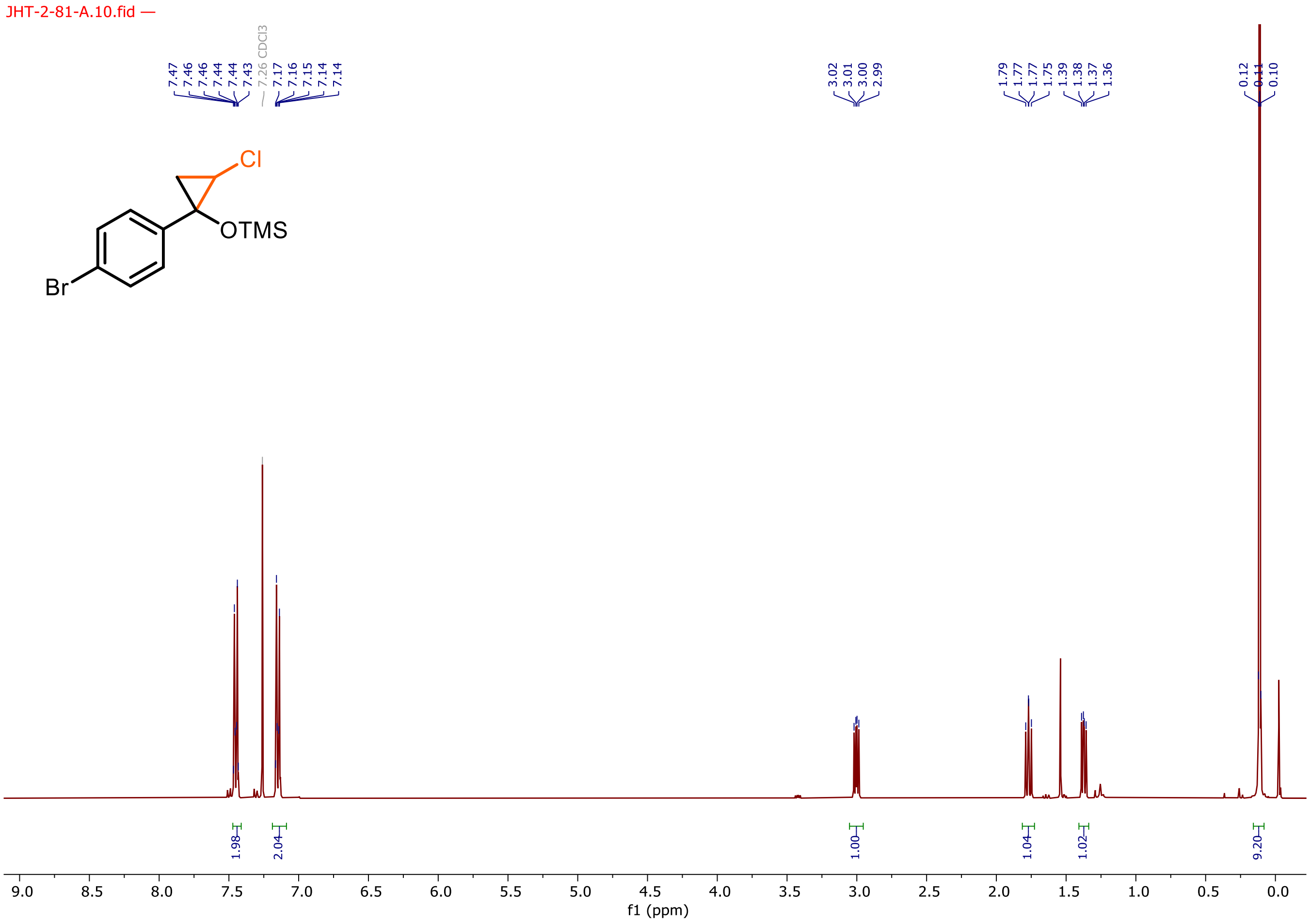

37: ^{13}C NMR (101 MHz, $CDCl_3$)

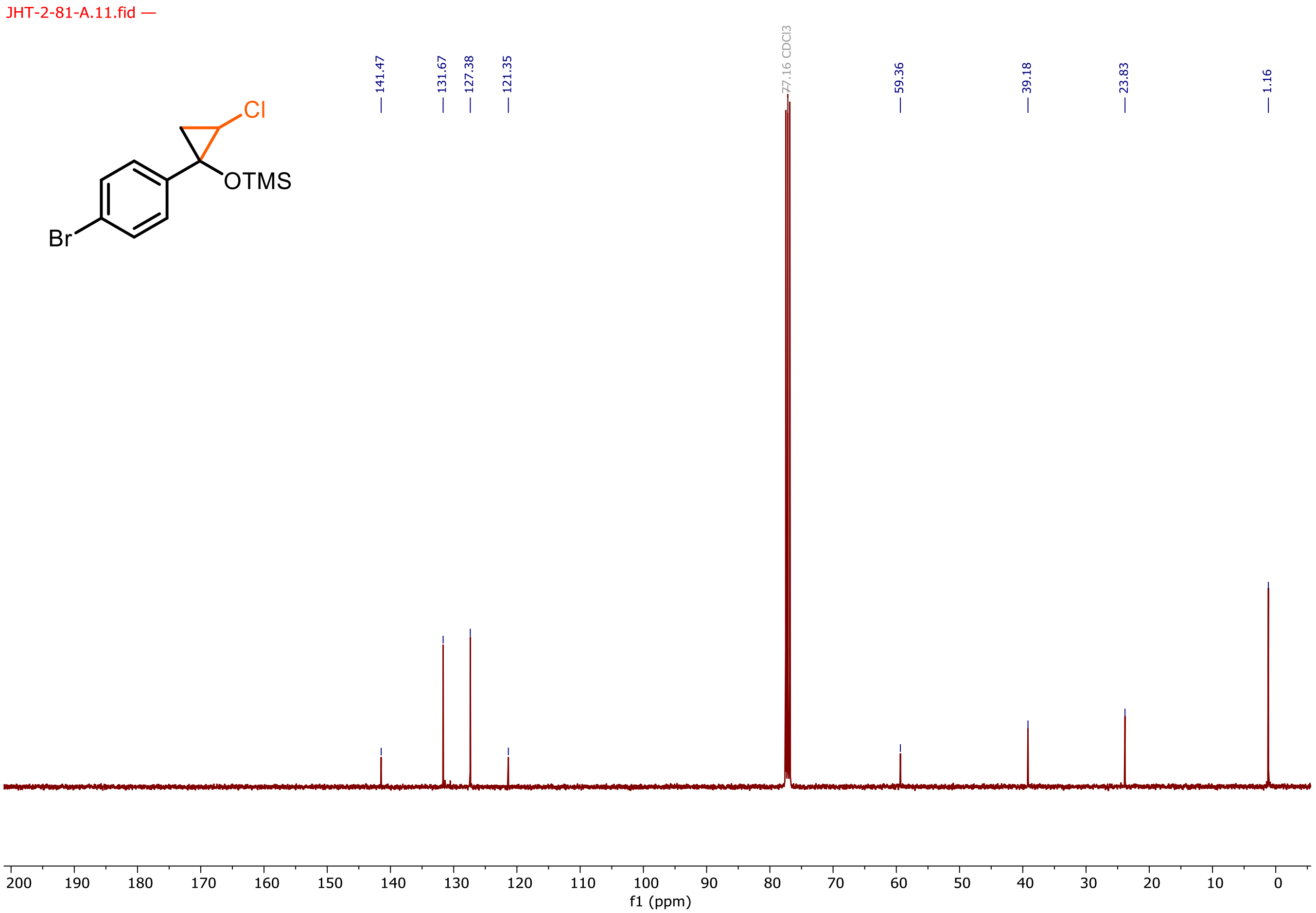

38: Crude ¹H NMR (400 MHz, $CDCl_3$)

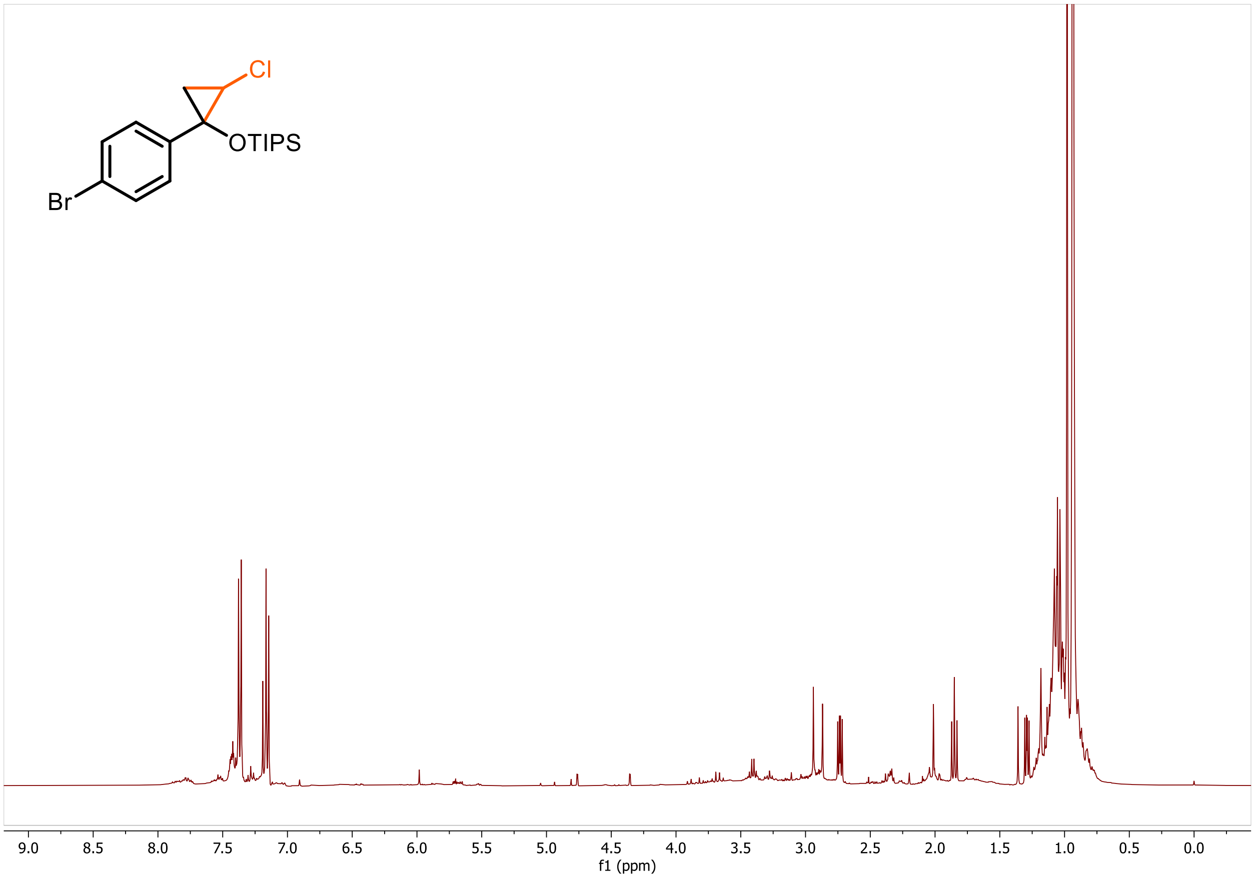

38: ^{1}H NMR (800 MHz, $CDCl_3$)

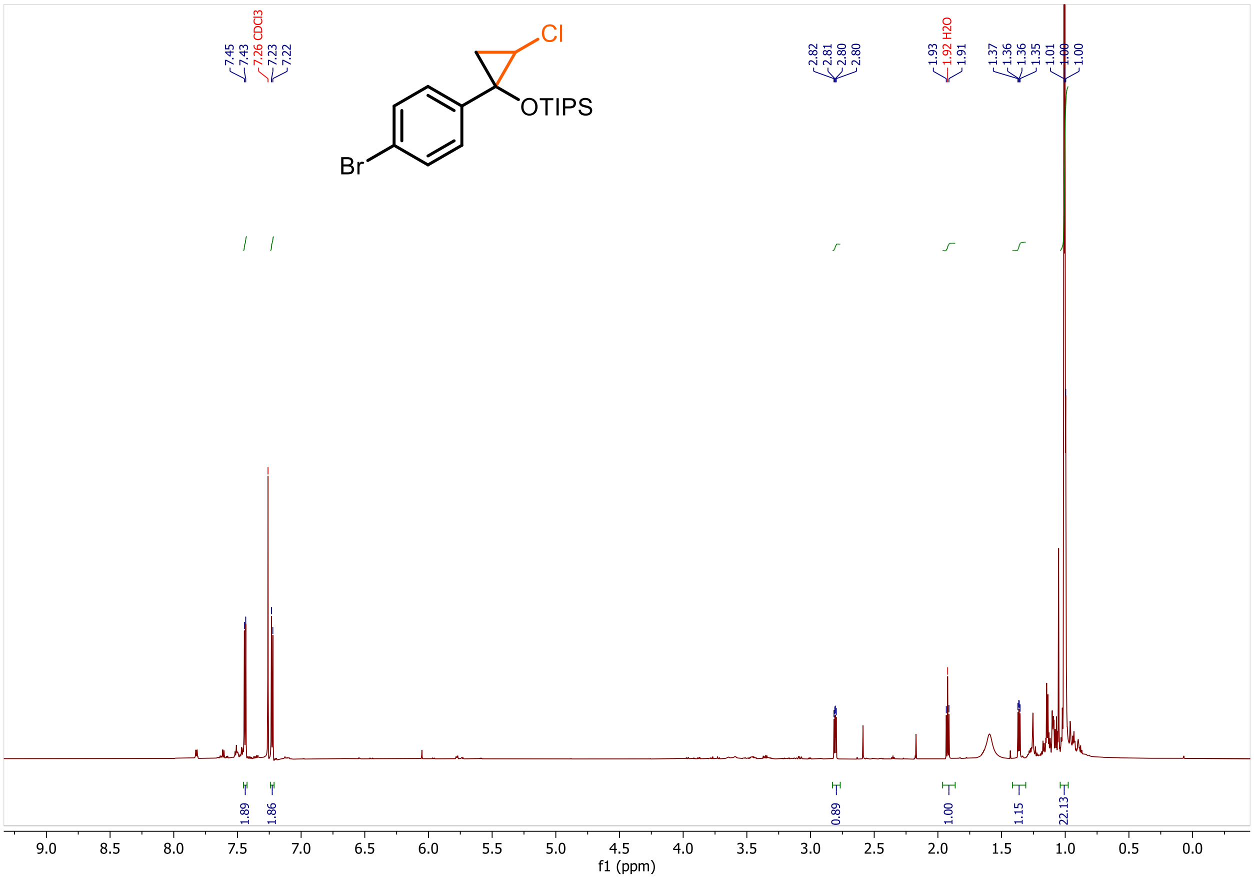

38: ^{13}C NMR (200 MHz, $CDCl_3$)

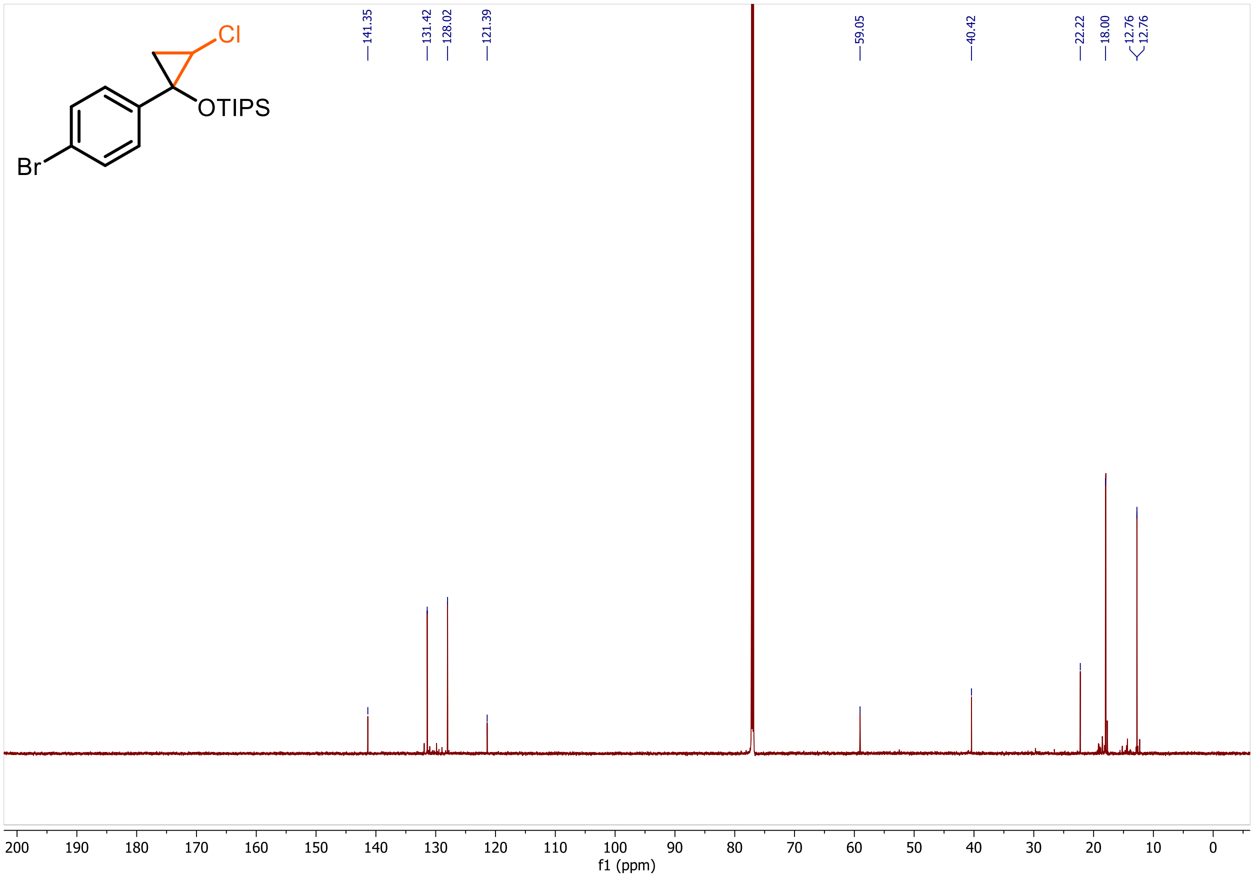

39: Crude ^{1}H NMR (400 MHz, $CDCl_3$)

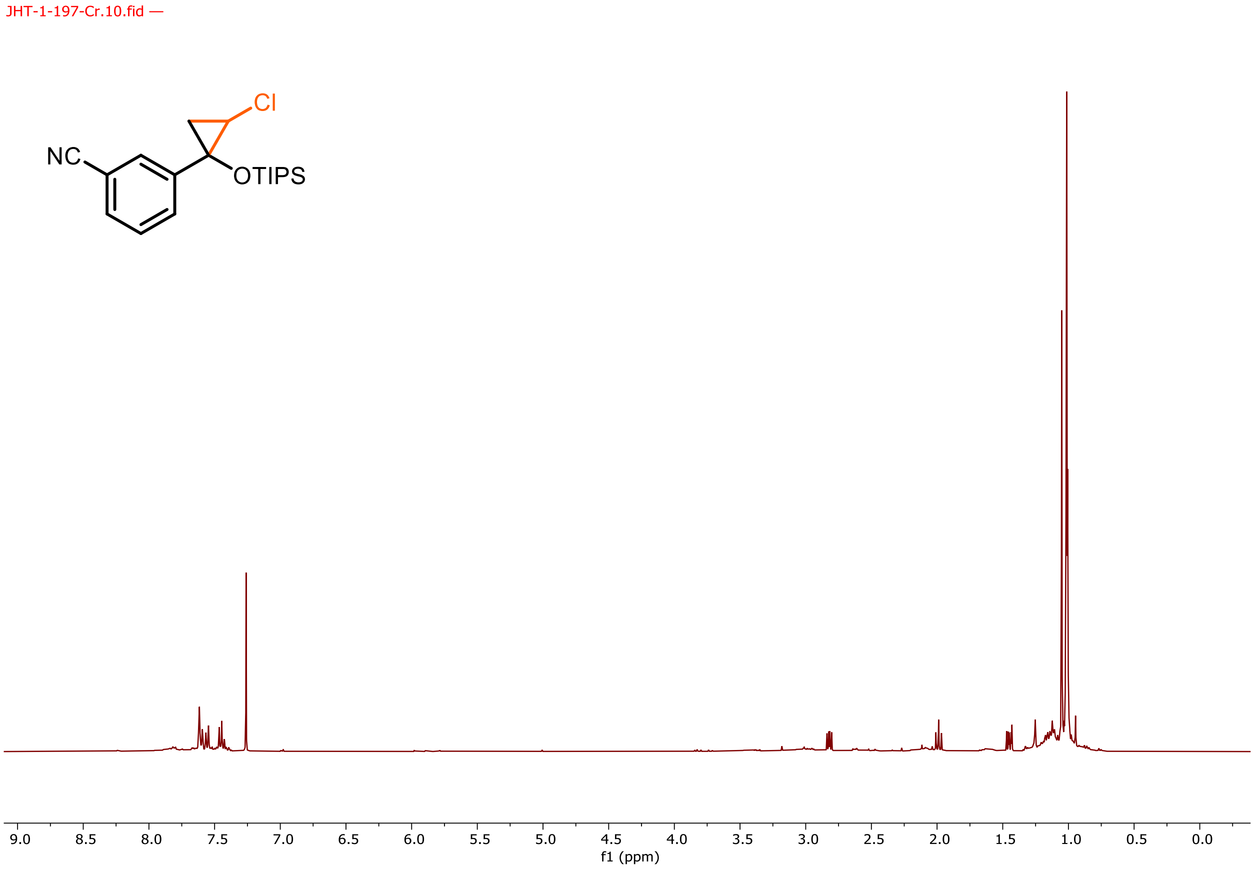

39: ^{1}H NMR (400 MHz, $CDCl_3$)

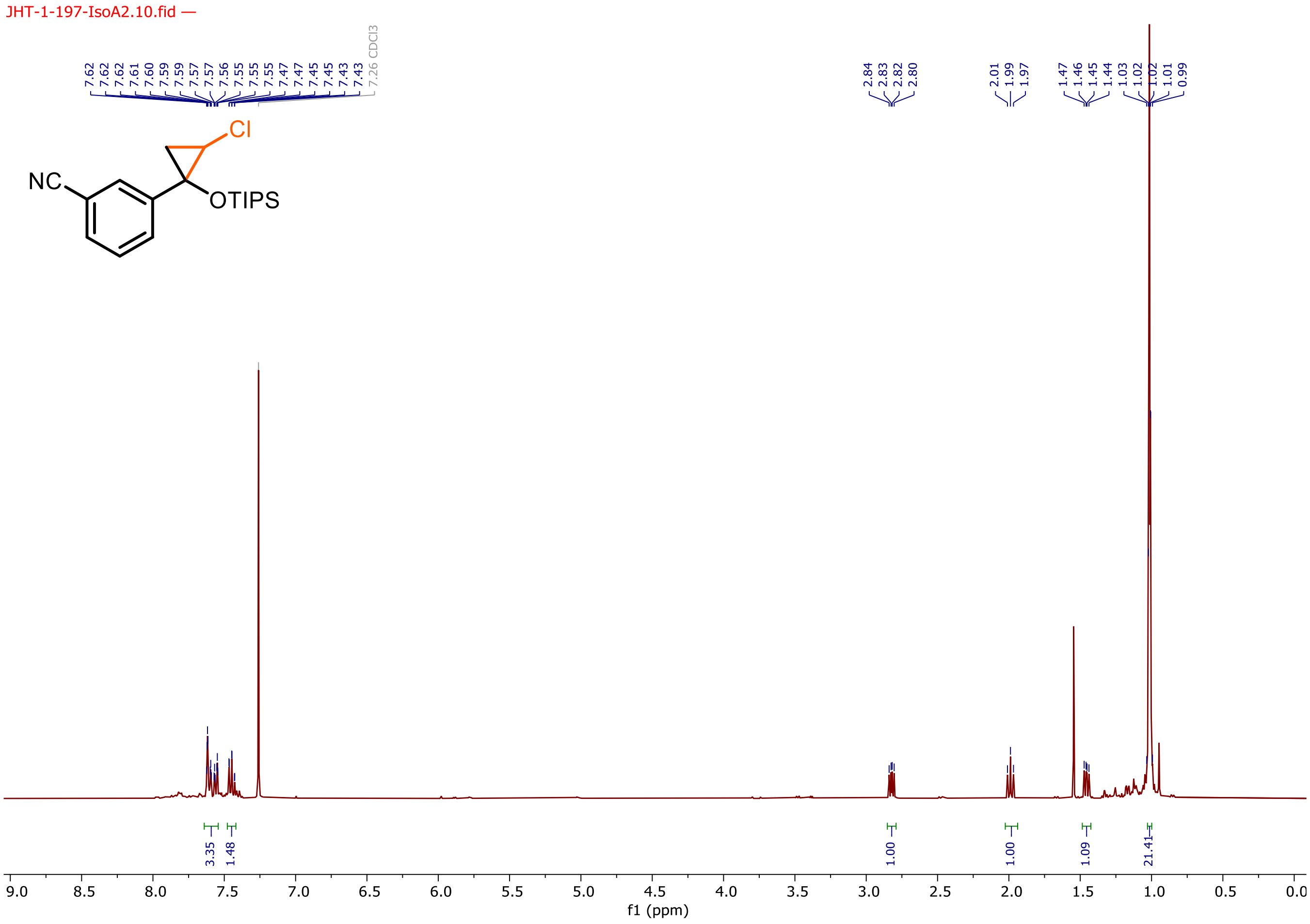

39: ^{13}C NMR (101 MHz, $CDCl_3$)

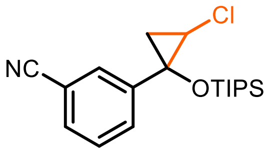

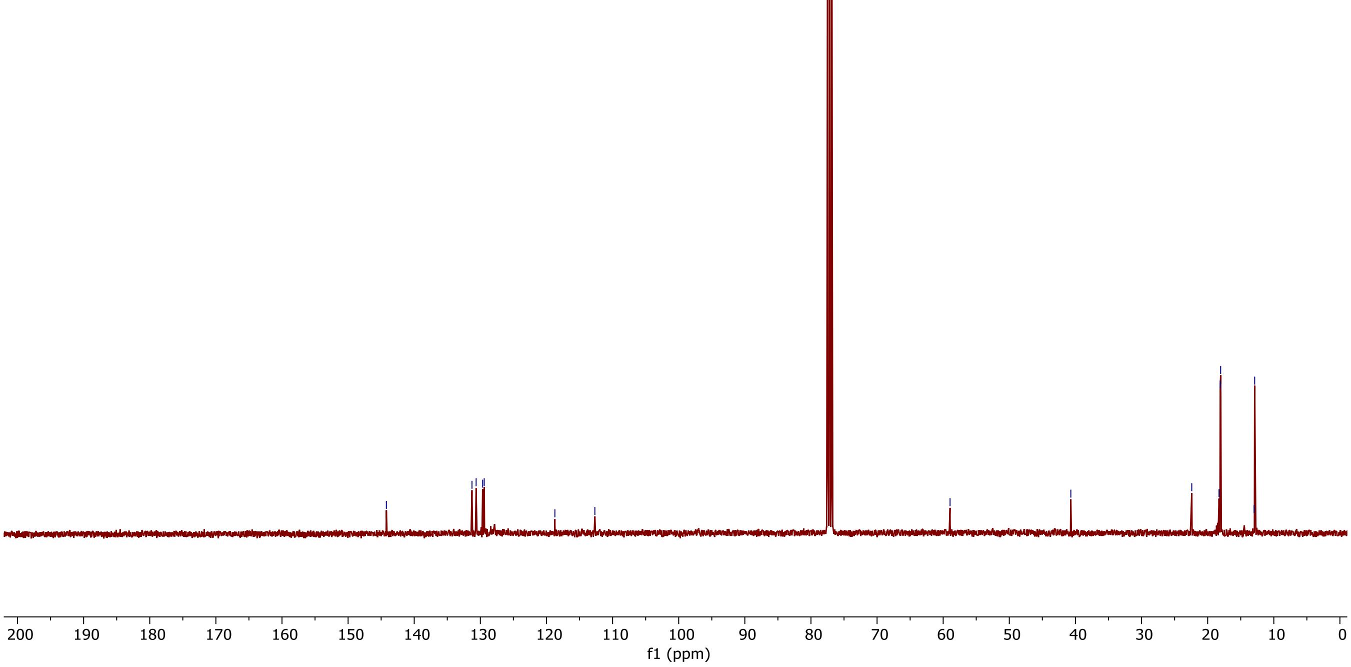

40: Crude ^{1}H NMR (400 MHz, $CDCl_3$)

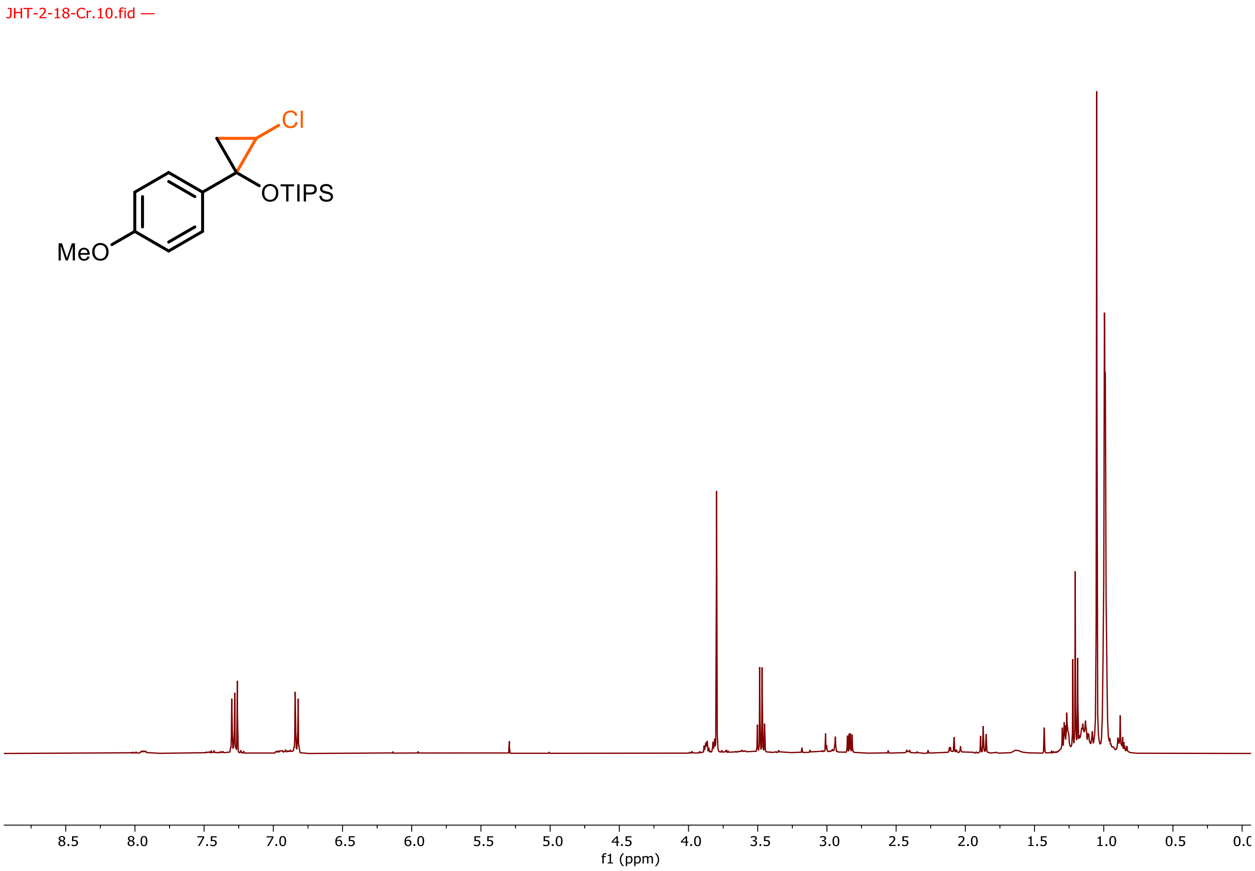

40: ^{1}H NMR (400 MHz, $CDCl_3$)

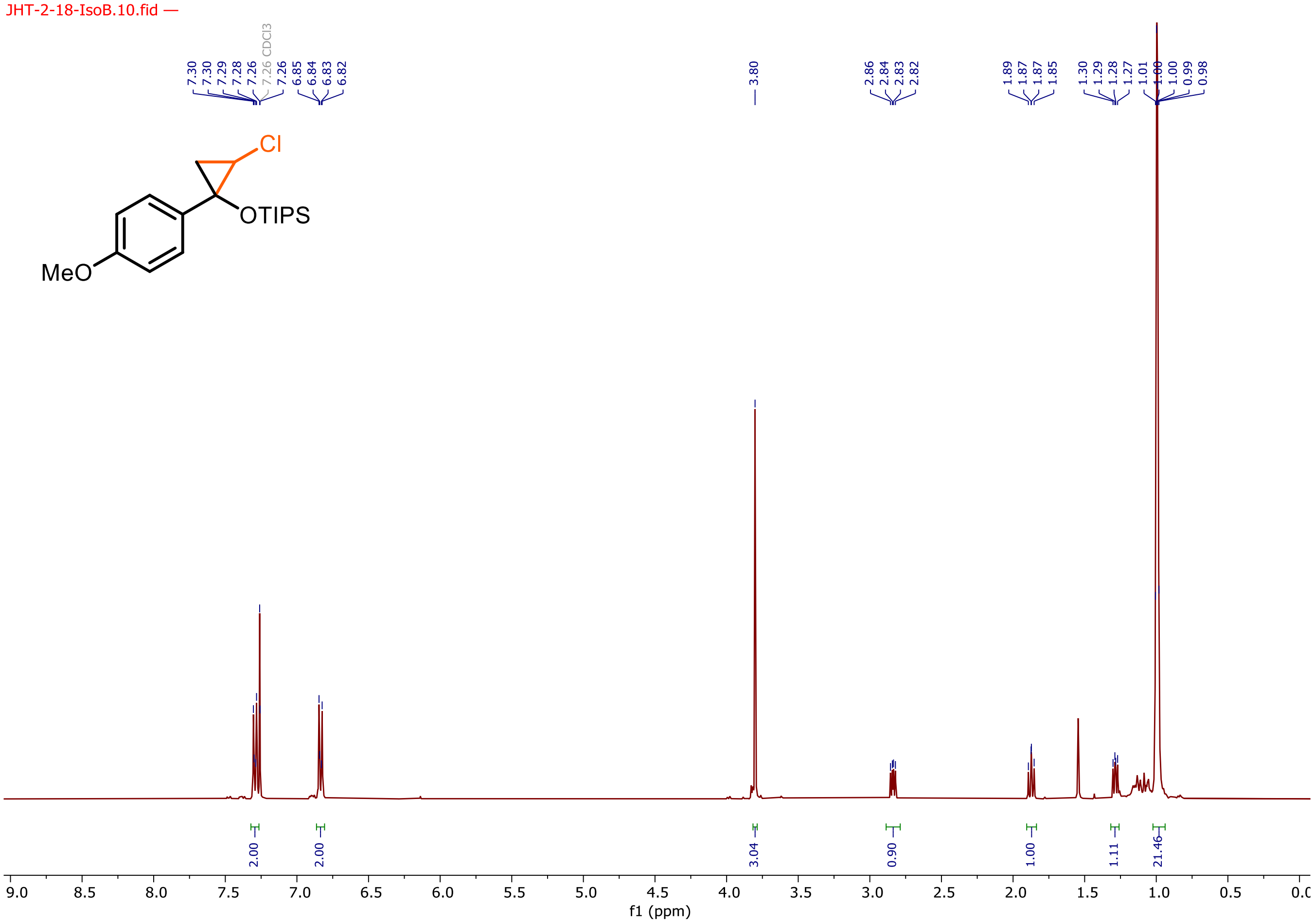

40: ^{13}C NMR (101 MHz, $CDCl_3$)

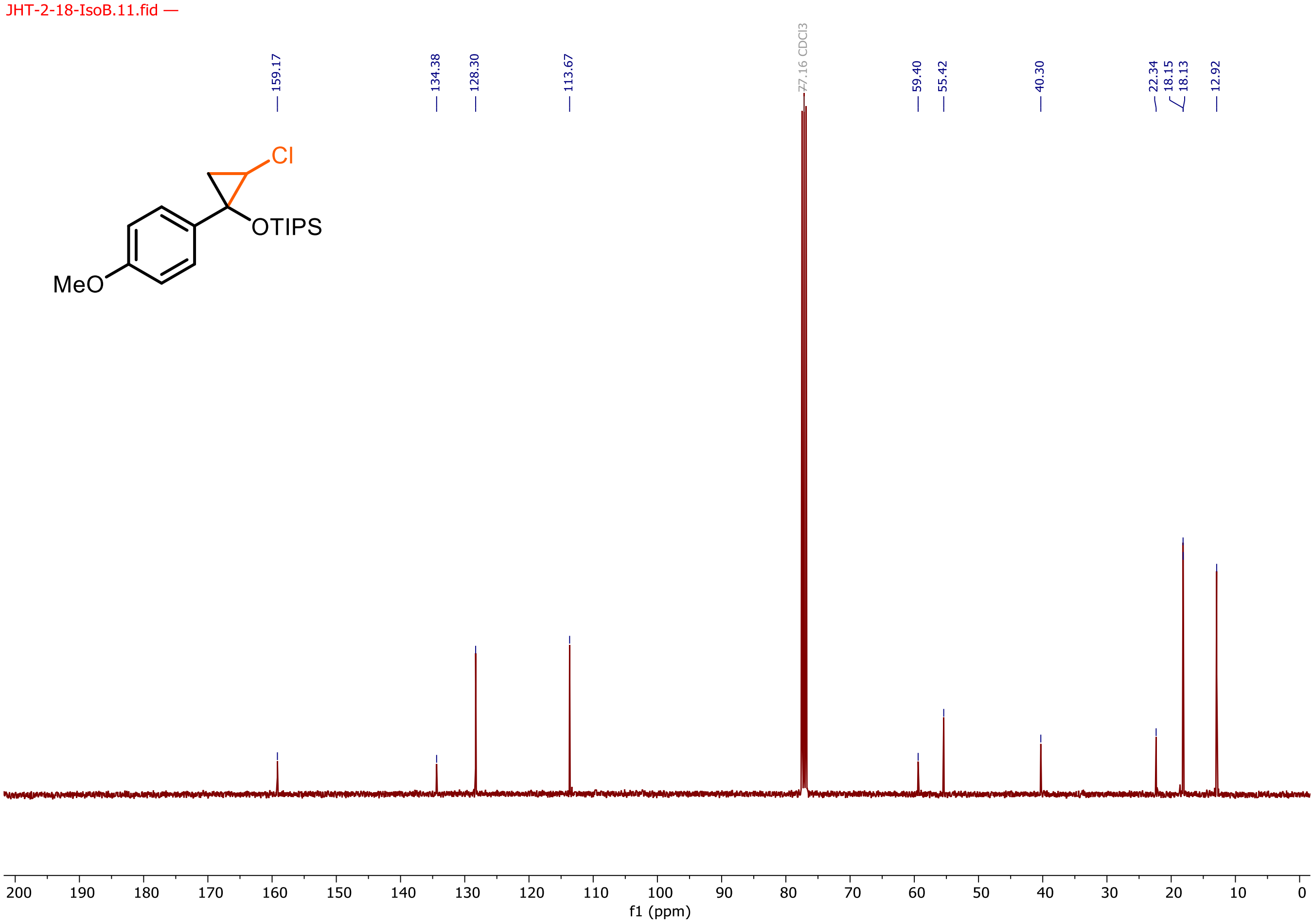

41: ¹H NMR (400 MHz, $CDCl_3$)

<u>Note:</u> This compound is highly unstable and is prone to decompose to the β-chloroketone.

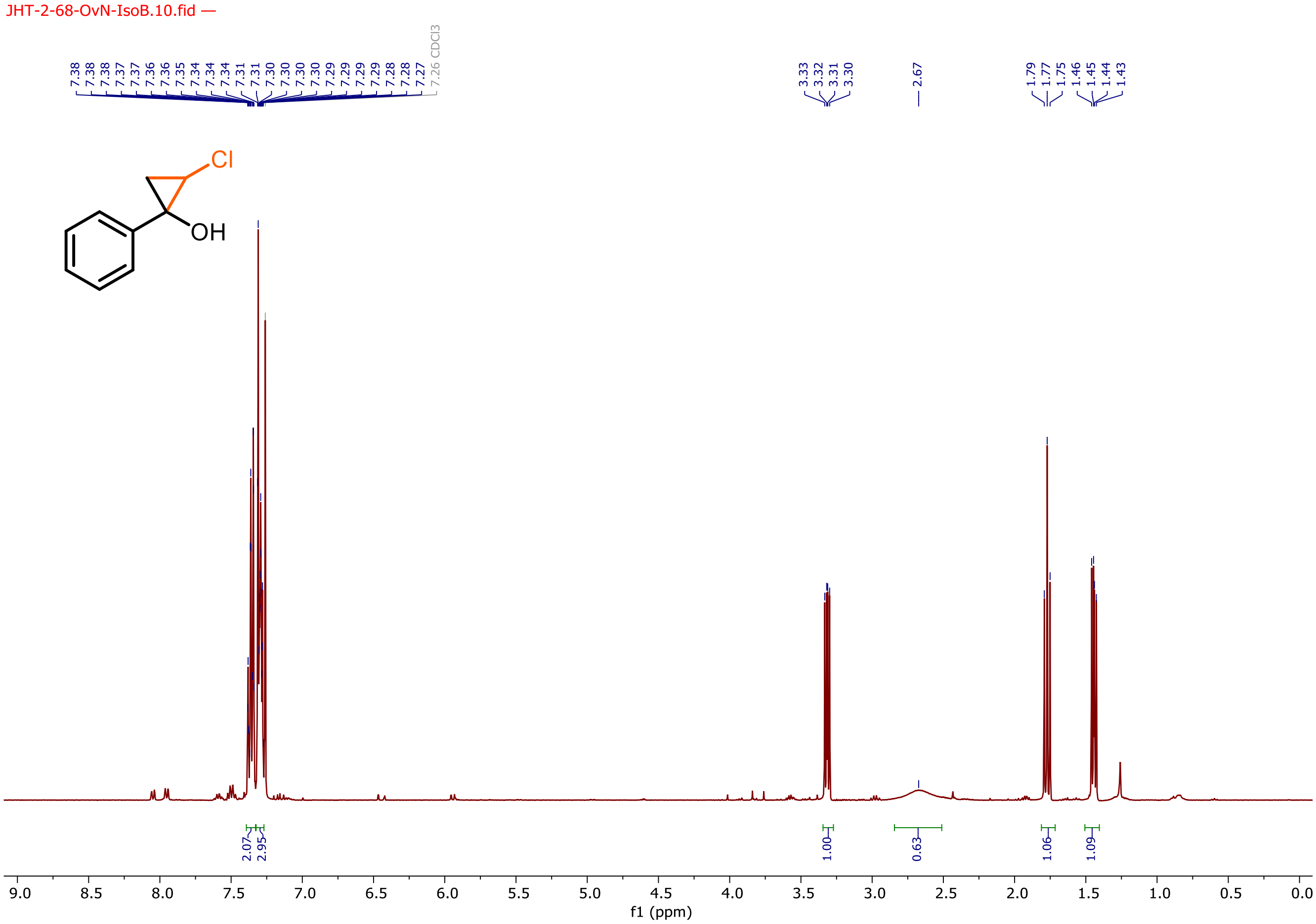

41: ^{13}C NMR (101 MHz, $CDCl_3$)

Note: This compound is highly unstable and is prone to decompose to the β-chloroketone.

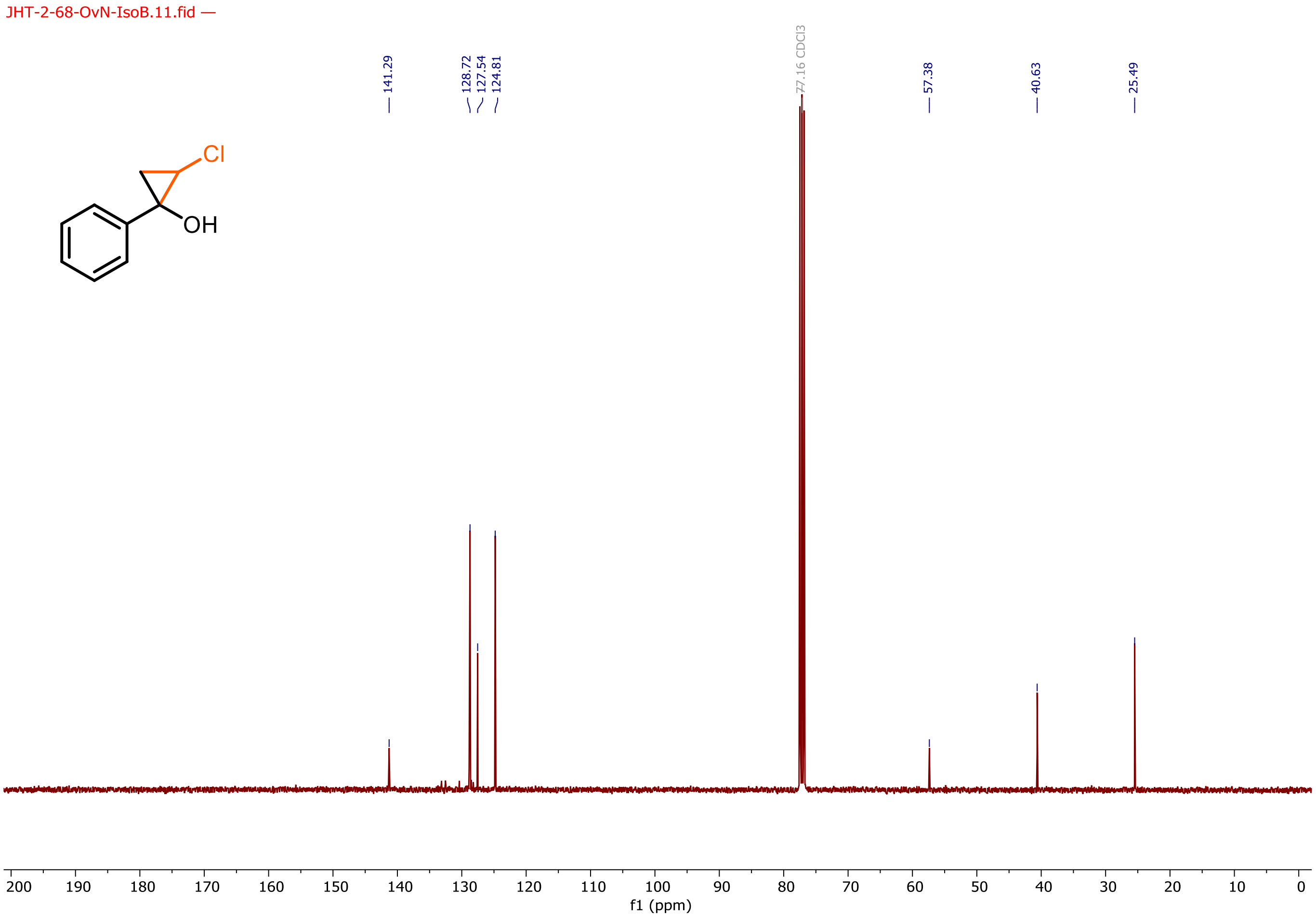

42: ^{1}H NMR (400 MHz, $CDCl_3$)

<u>Note:</u> This compound is highly unstable and is prone to decompose to the β-chloroketone.

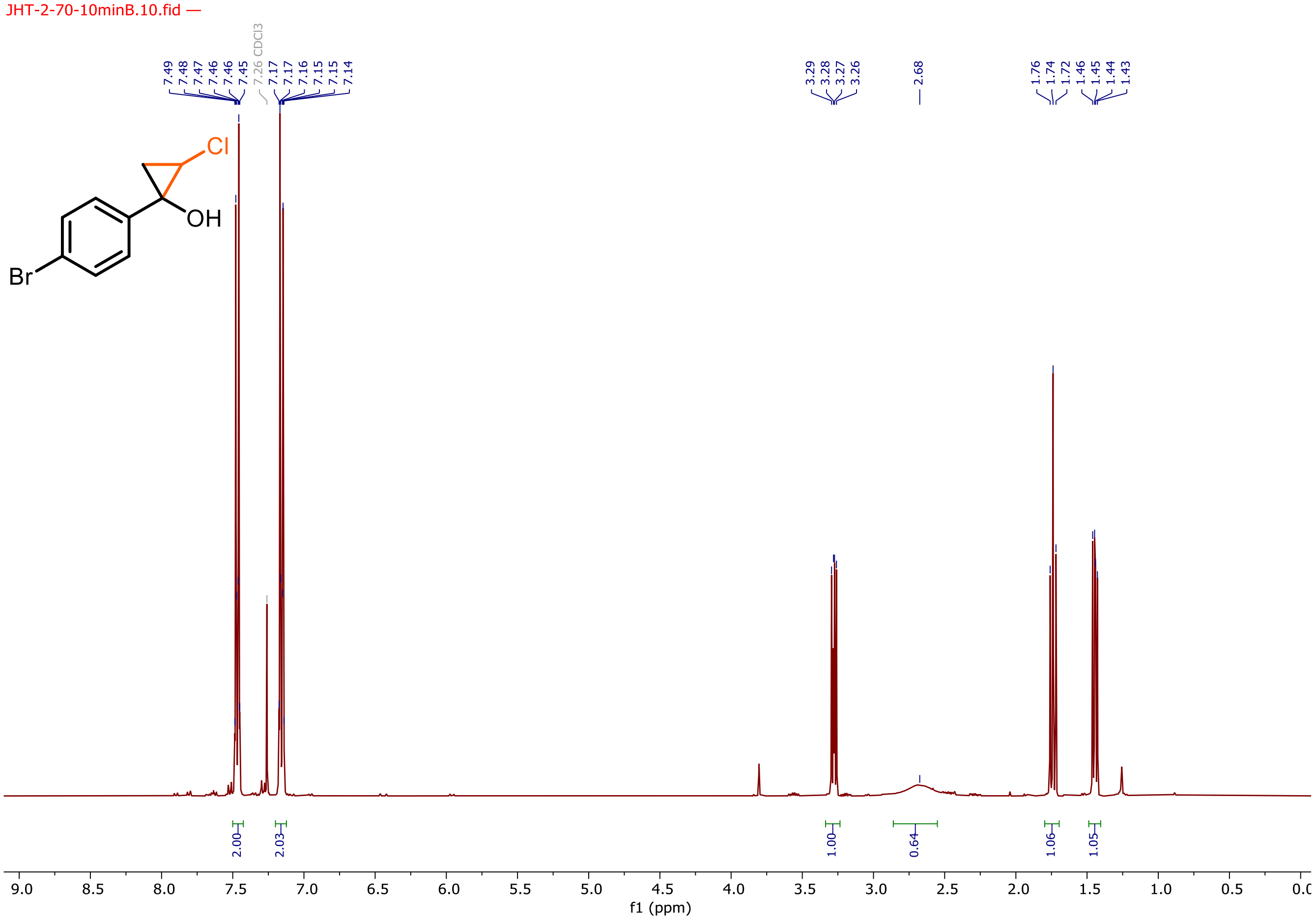

42: ^{13}C NMR (101 MHz, $CDCl_3$)

<u>Note:</u> This compound is highly unstable and is prone to decompose to the β-chloroketone.

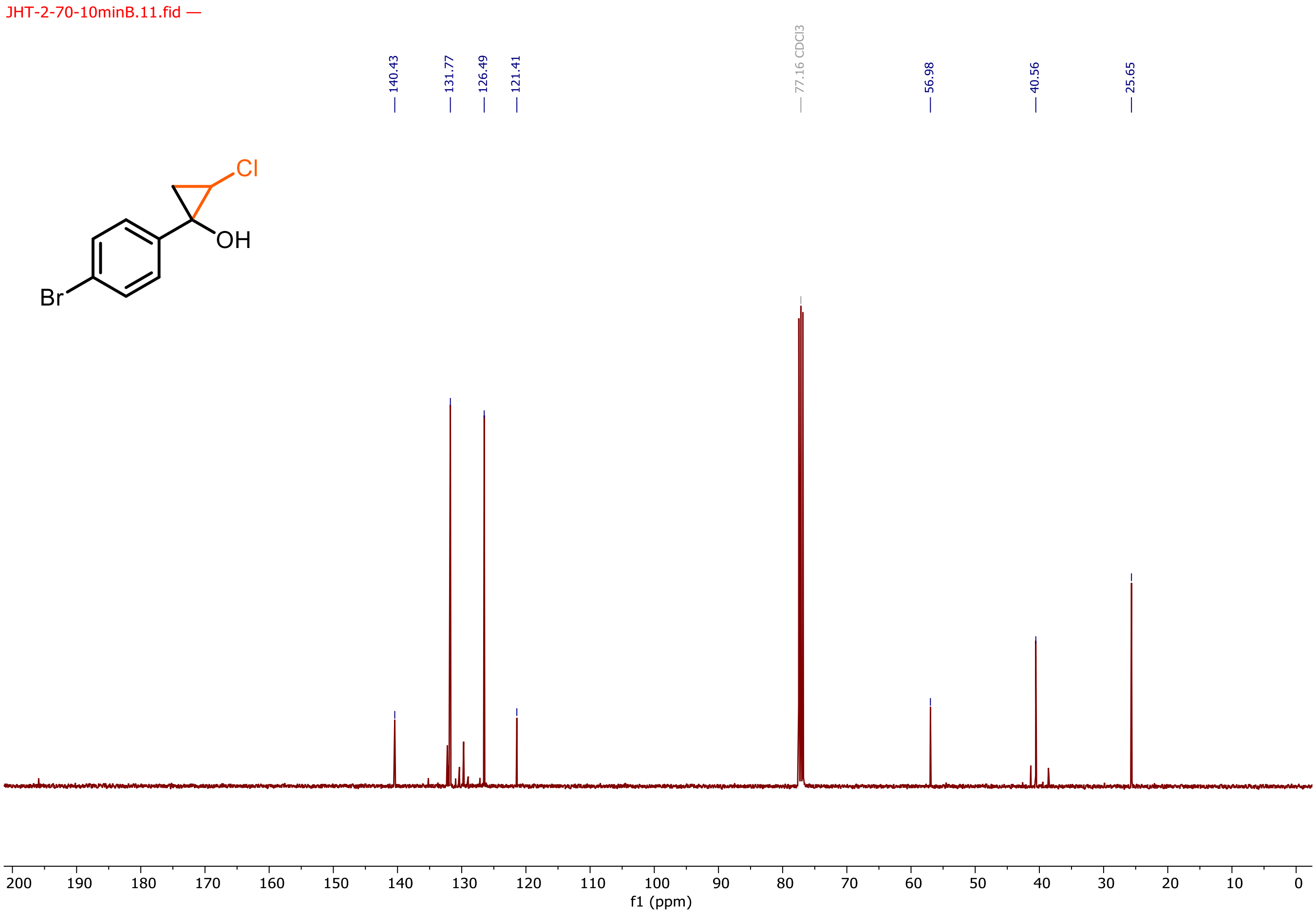

42: NOESY (800 MHz, C_6D_6)

<u>Note:</u> This compound is highly unstable and is prone to decompose to the β-chloroketone.

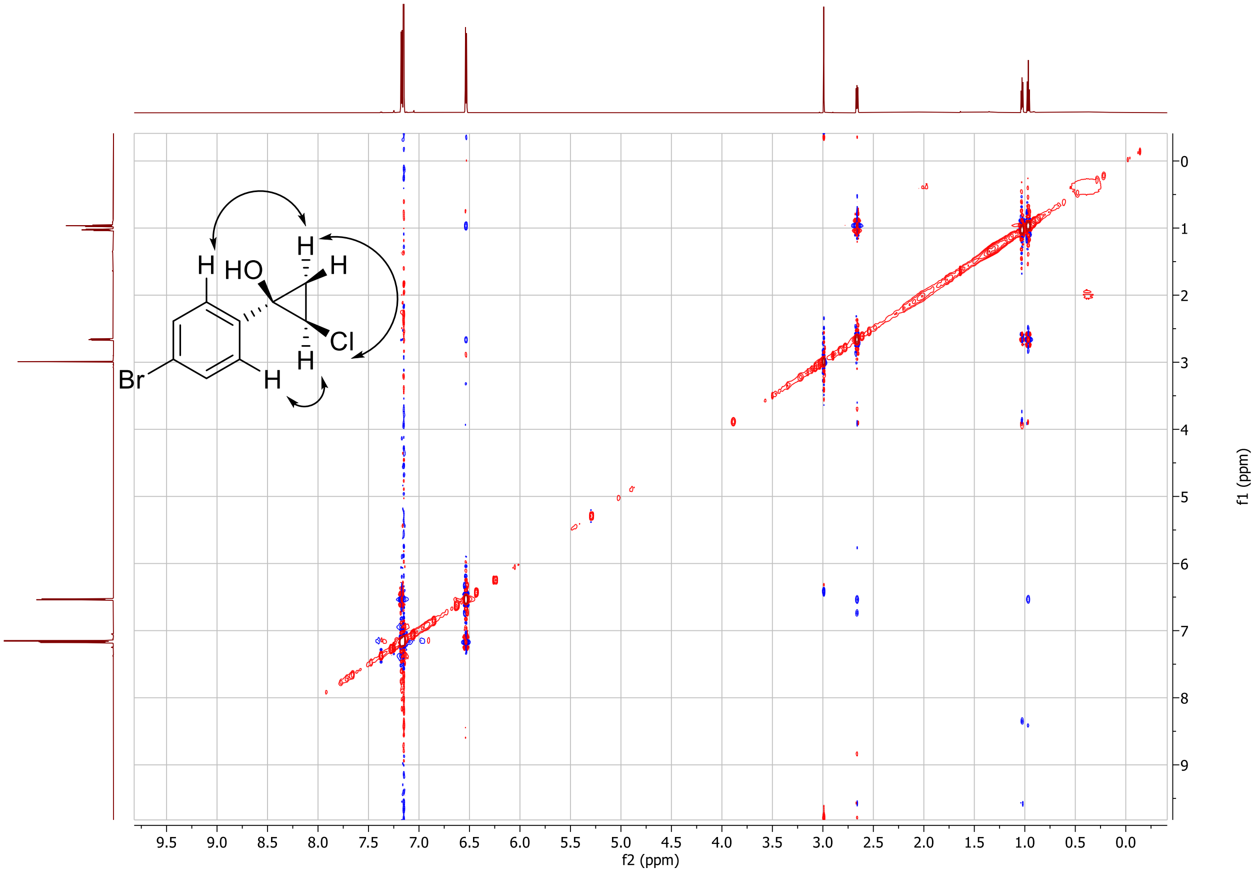

43: ^{1}H NMR (800 MHz, $CDCl_3$)

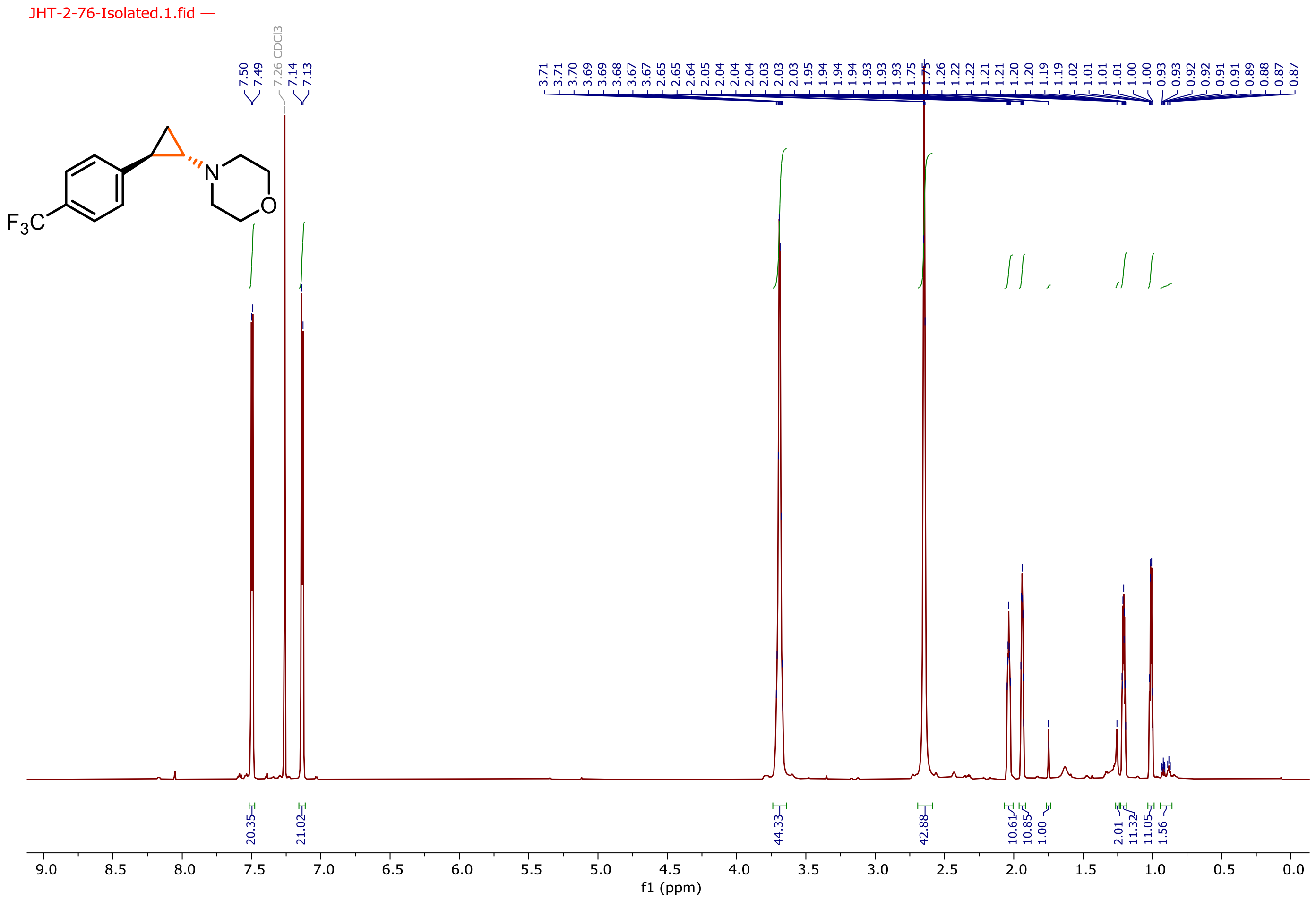

43: ^{13}C NMR (201 MHz, $CDCl_3$)

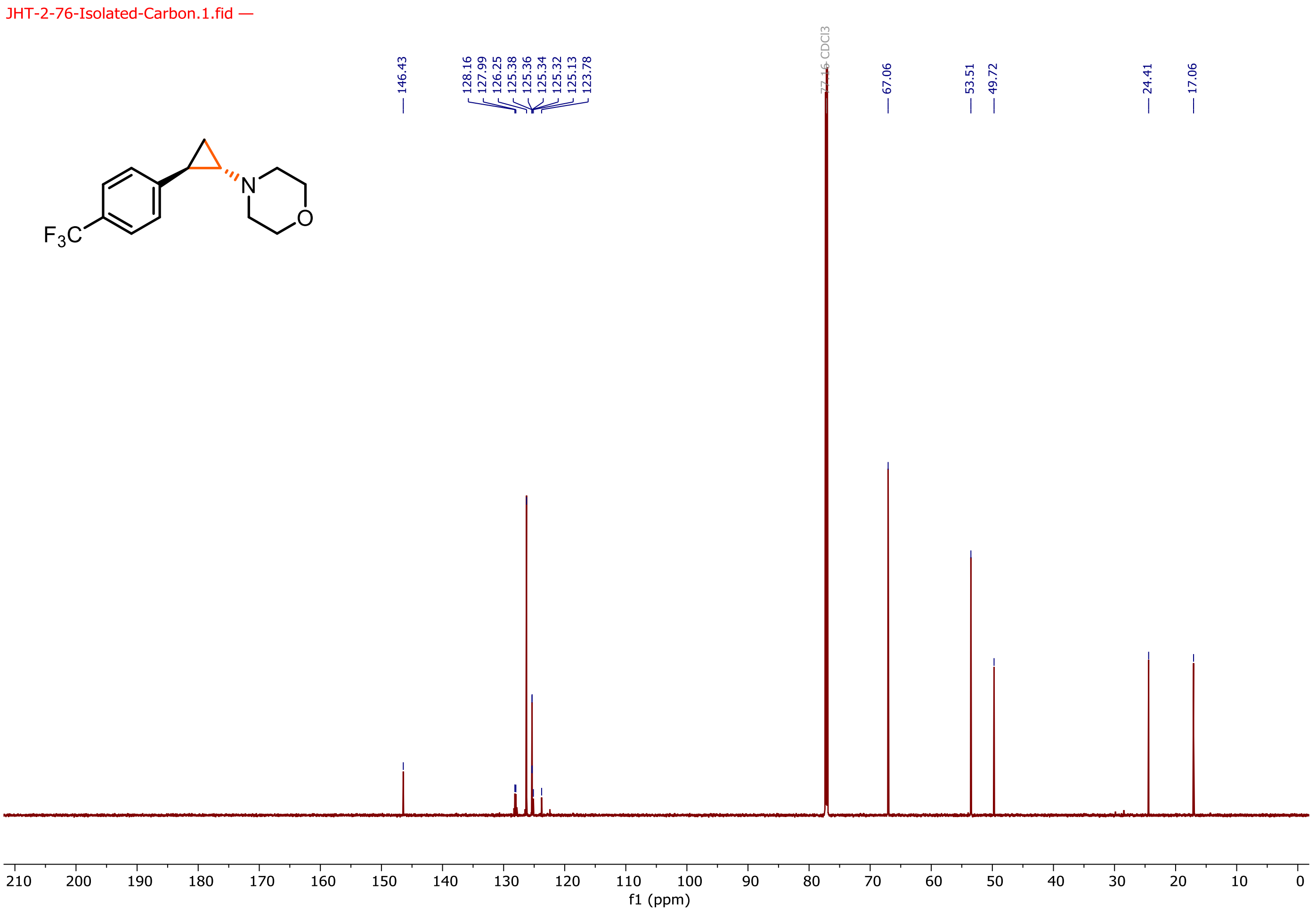

43: NOESY (800 MHz, $CDCl_3$)

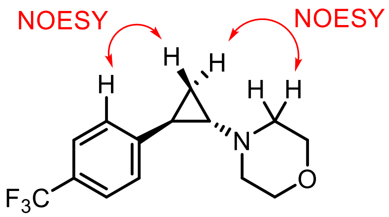

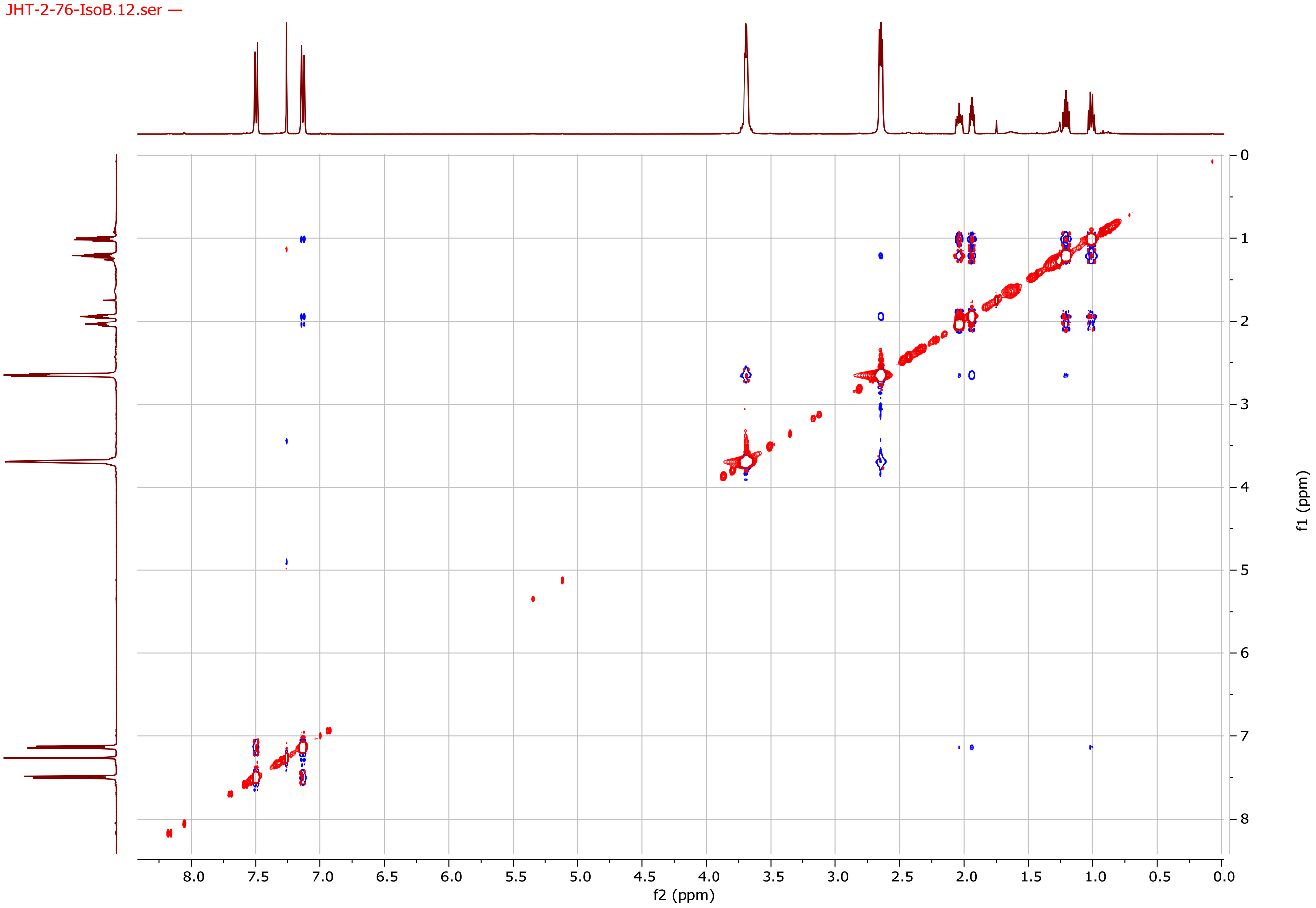

43: ^{19}F NMR (376 MHz, CDCl$_3$)

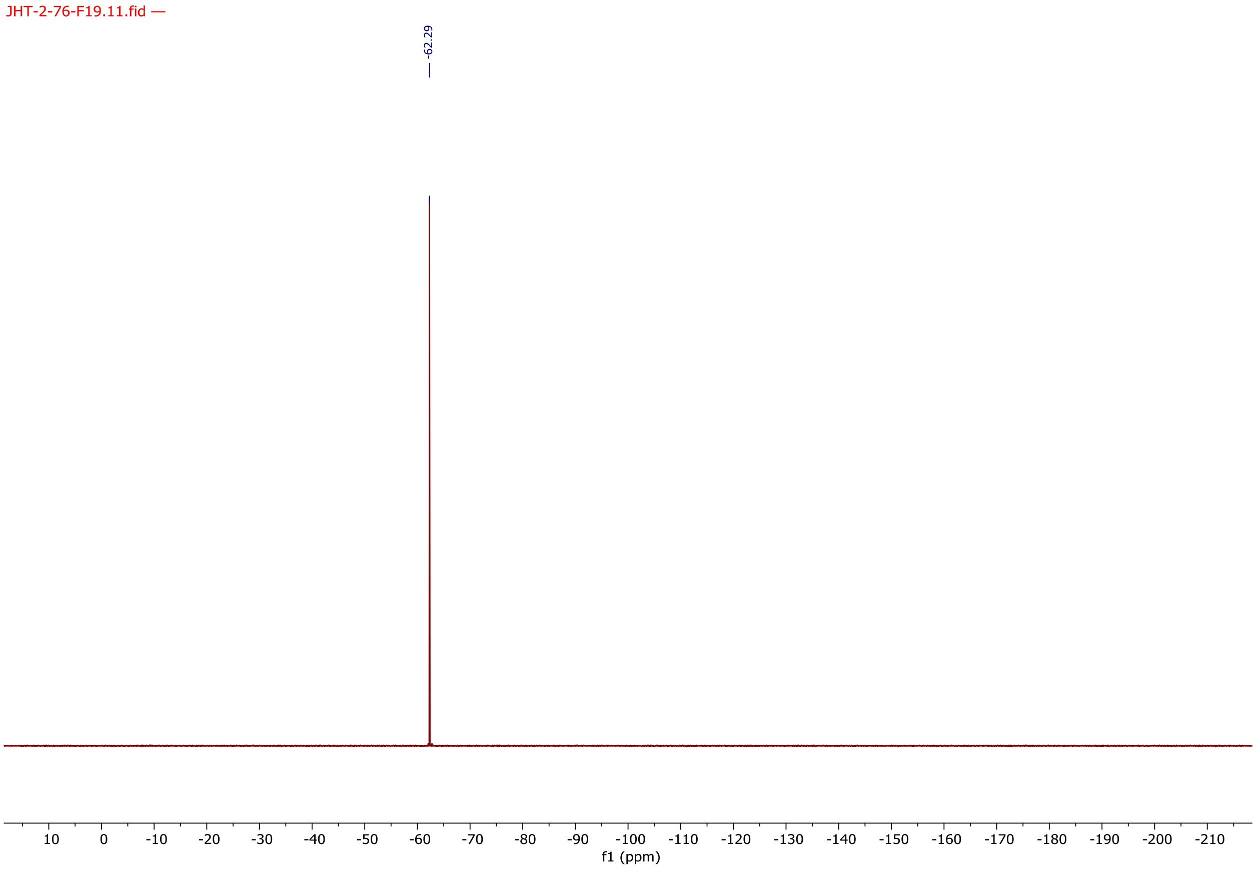

44: ^{1}H NMR (800 MHz, $CDCl_3$)

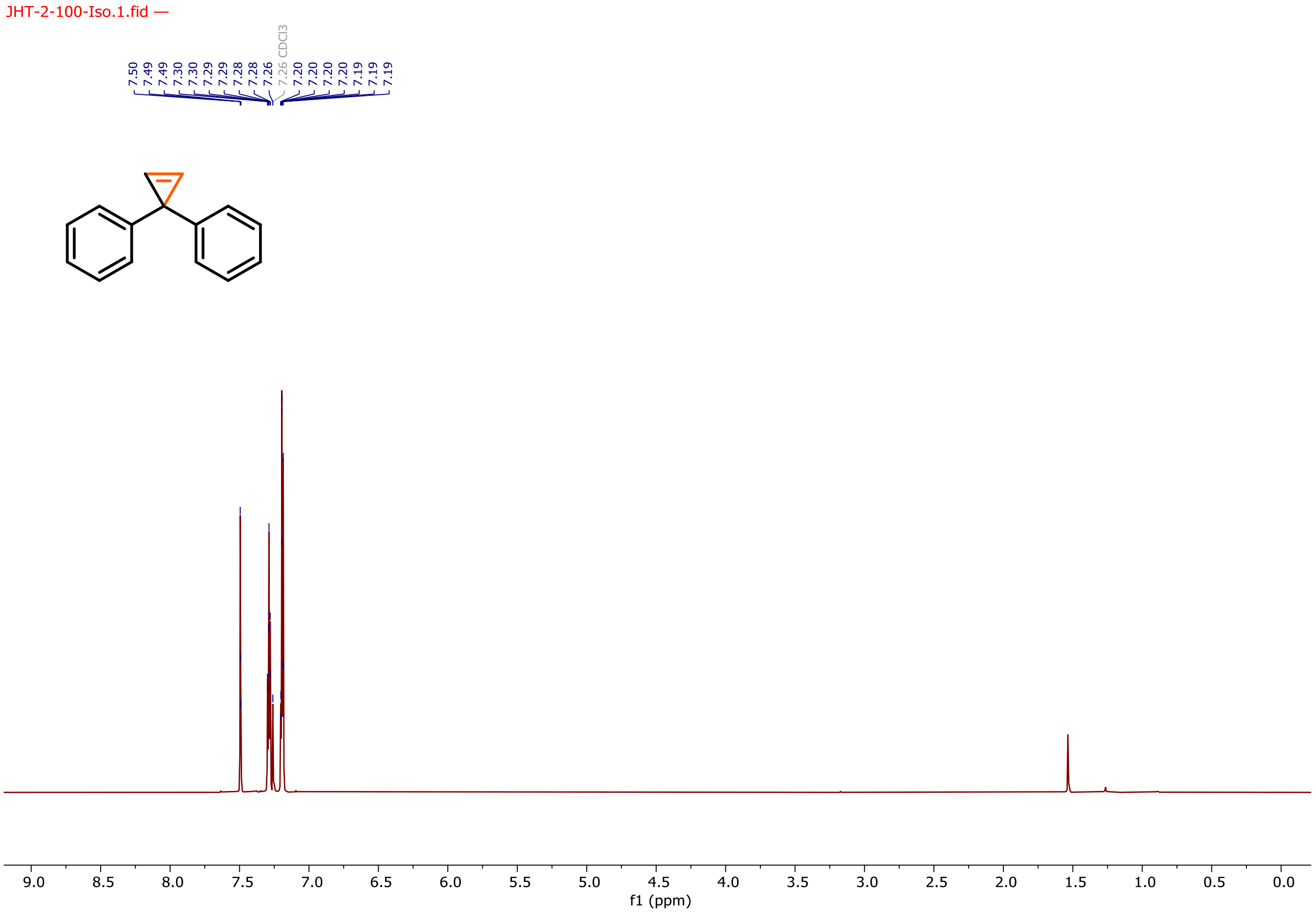

44: ^{13}C NMR (201 MHz, $CDCl_3$)

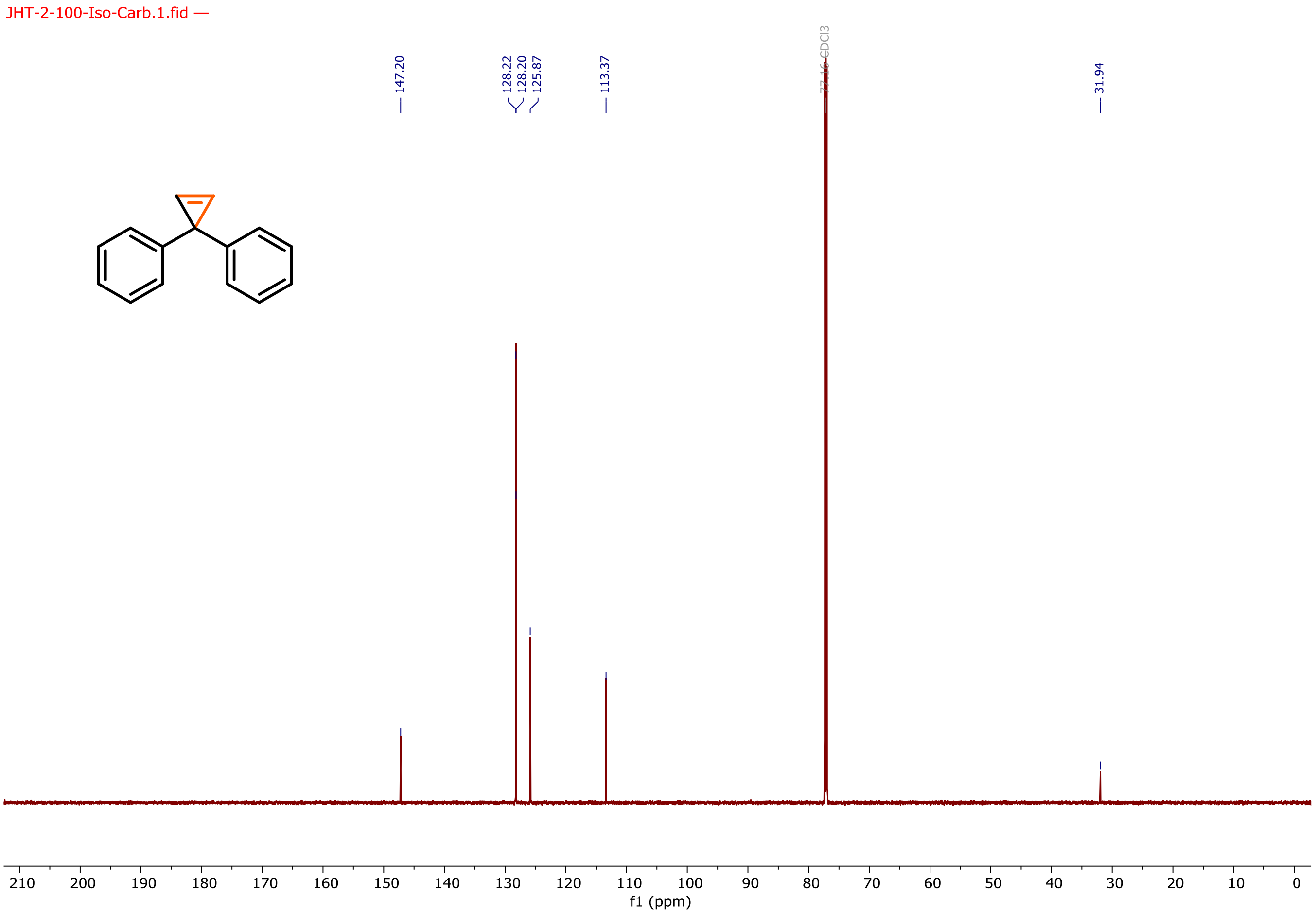

46: ^{1}H NMR (800 MHz, $CDCl_3$)

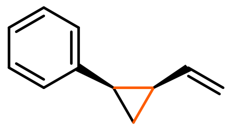

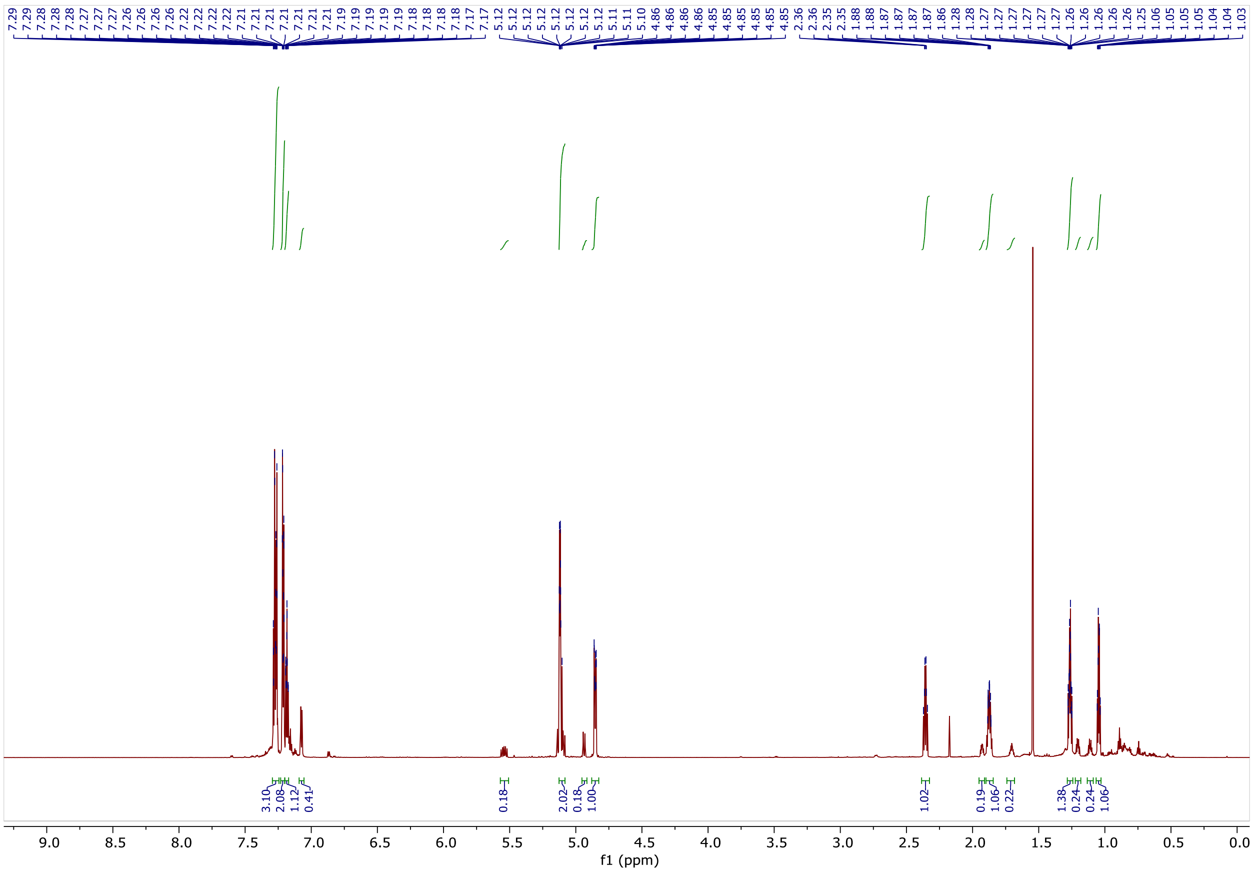

46: ^{13}C NMR (201 MHz, $CDCl_3$)

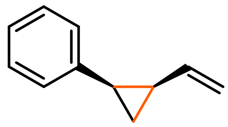

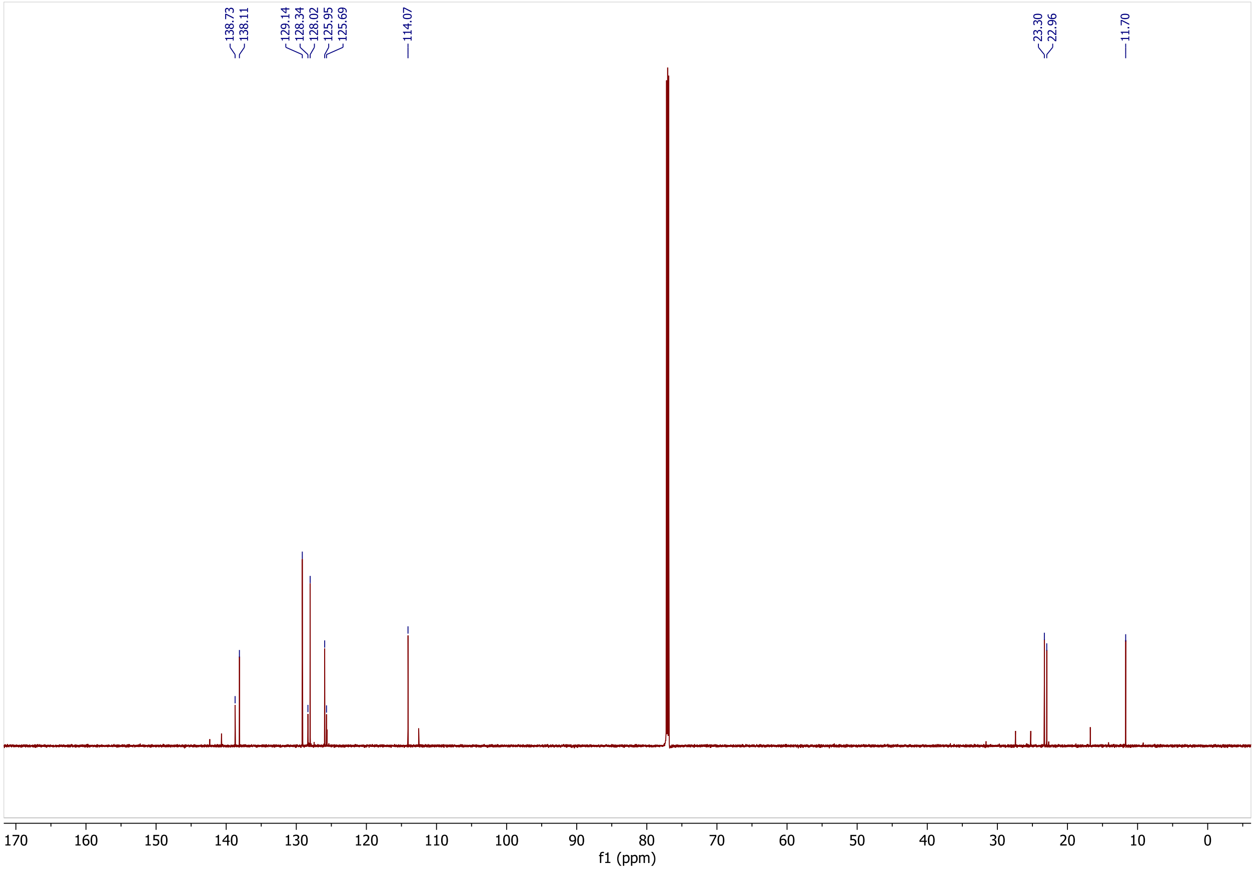

47: ^{1}H NMR (400 MHz, $CDCl_3$)

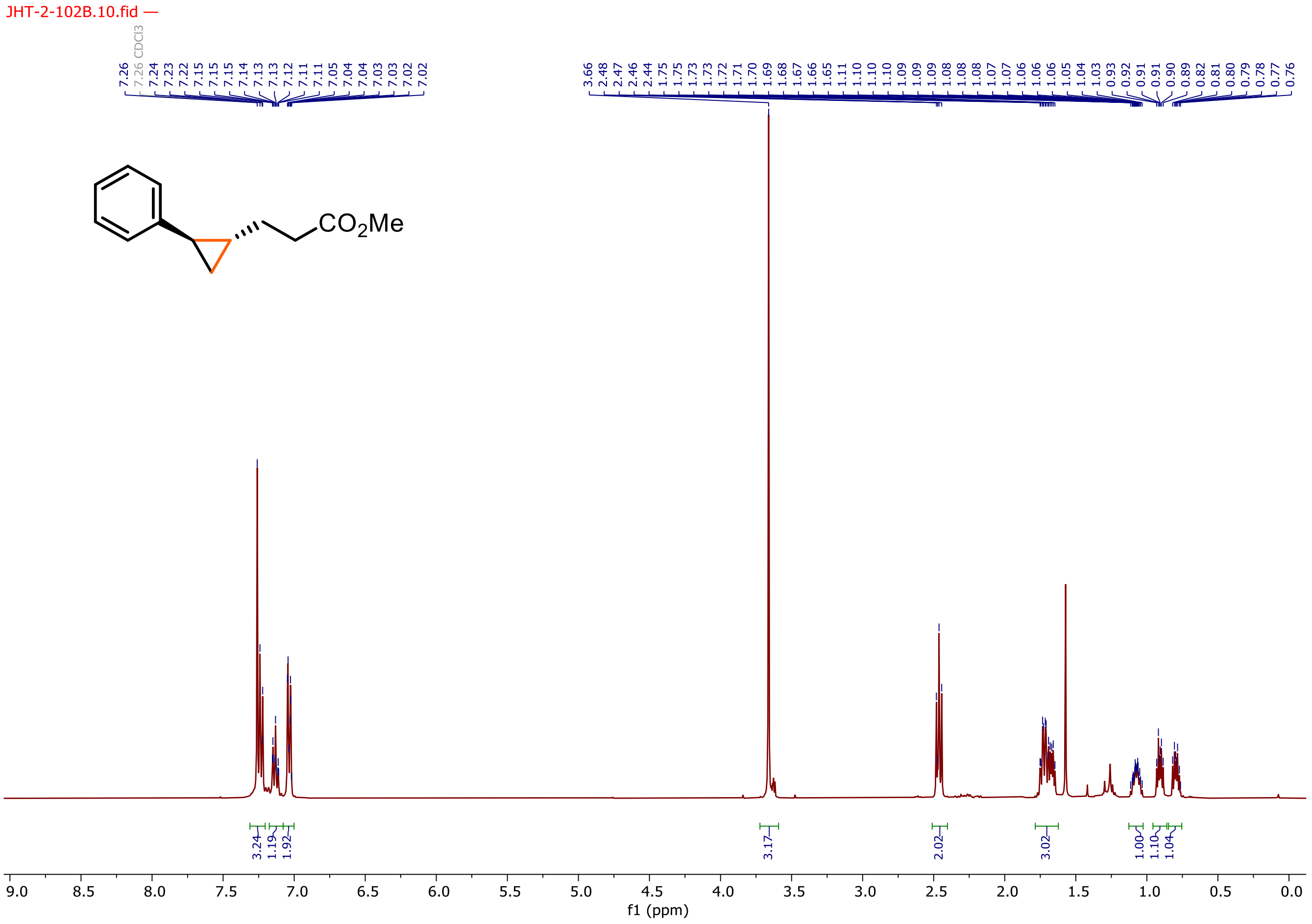

47: ^{13}C NMR (101 MHz, $CDCl_3$)

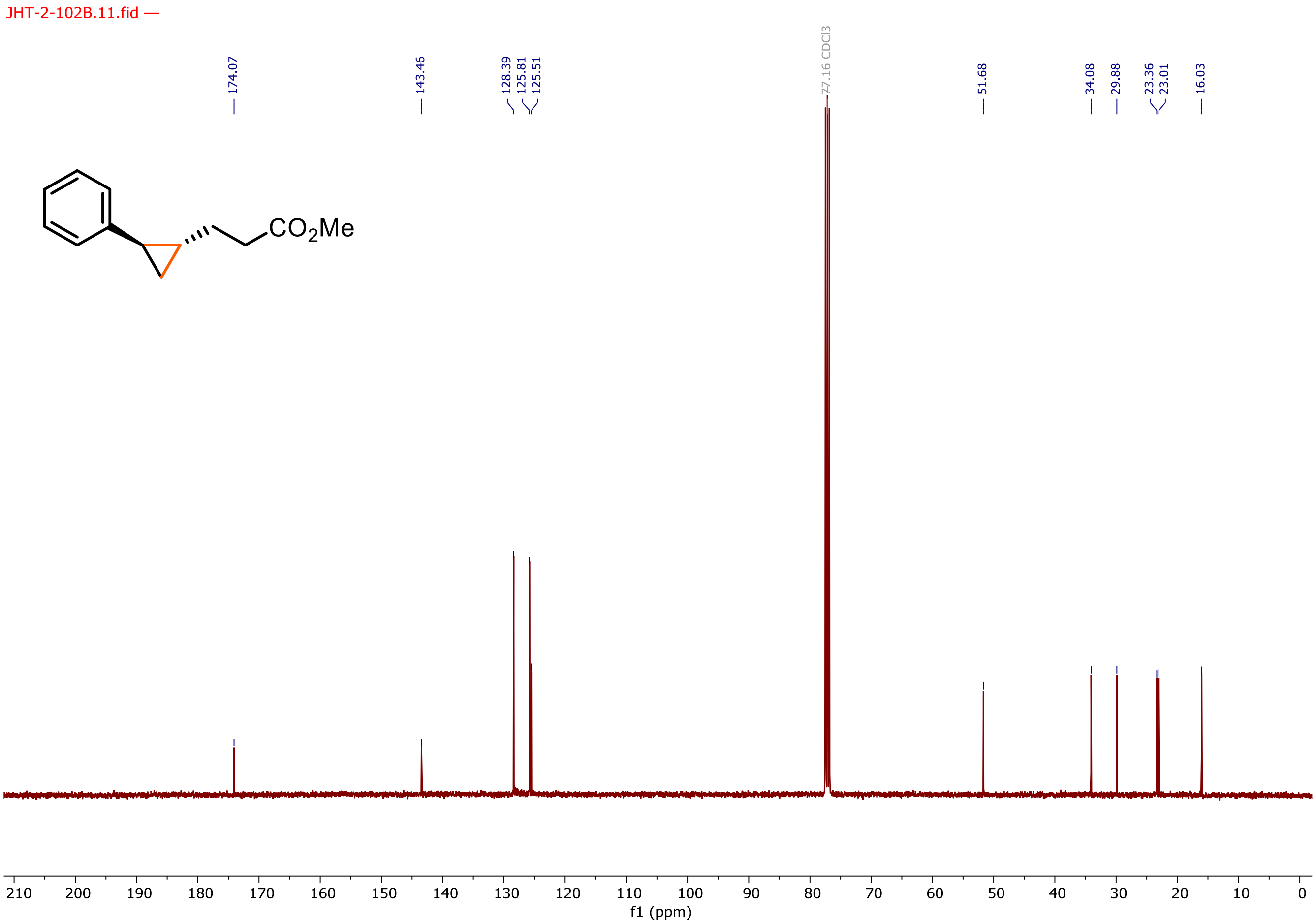

Supplement: Supplementary file 1 [file au6c00381_si_001.pdf]
